# Hyperpolarization of Pyridine ^15^ *N*‐Oxide Molecular Probes Enabled by Parahydrogen

**DOI:** 10.1002/anie.202525716

**Published:** 2026-07-09

**Authors:** Ruhuai Mei, Lisa Maria Fries, Gonzalo Gabriel Rodriguez, Charlotte von Petersdorff‐Campen, Leander Konstantin May, Julius Frederik Matz, Sergey Korchak, Grysette Daher, Stefan Glöggler

**Affiliations:** ^1^ NMR Signal Enhancement Group Max Planck Institute for Multidisciplinary Sciences Göttingen Germany; ^2^ Center For Biostructural Imaging of Neurodegeneration University Medical Center Göttingen Göttingen Germany; ^3^ Advanced Imaging Research Center University of Texas Southwestern Medical Center Dallas Texas USA; ^4^ Department of Biomedical Engineering University of Texas Southwestern Medical Center Dallas Texas USA

**Keywords:** molecular probe, NMR, parahydrogen, pyridine N‐oxide, spectroscopy

## Abstract

Magnetic resonance (MR) is a powerful non‐invasive technique for probing structural, functional, and metabolic processes with high spatial and temporal resolution. However, its inherently low sensitivity restricts broader applications. The use of hyperpolarized contrast agents has thus, emerged as an attractive approach to overcome this limitation and expand the capabilities. Among the available hyperpolarization techniques, parahydrogen‐induced polarization (PHIP) provides a rapid and cost‐efficient means to enhance magnetic resonance signals substantially. Yet, direct hyperpolarization of biomolecules, metabolites, or pharmaceuticals in vivo remains challenging, necessitating the development of versatile molecular tags and probes for hyperpolarized magnetic resonance (HP‐MR). In particular, imparting specific sensing functions—such as pH responsiveness and enzyme activity detection—to these HP molecular tags is of growing importance. Herein, we introduce pyridine *N*‐oxides as hyperpolarizable molecular tags and present [^1^ ^5^N, D]‐labeled 2‐alkenylpyridine *N*‐oxides as highly efficient candidates for HP‐MR with up to 47% ^15^N spin polarization. This performance opens pathways for broad potential in biomedical and preclinical HP‐MR applications. The systems feature long ^1^ ^5^N spin–lattice relaxation times (up to T_1_ = 477 s), broad functional‐group compatibility, and excellent structural tunability. Their practical utility is exemplified by pH and H_2_O_2_ sensing and monitoring enzymatic reactions in water.

## Graphical abstract

Pyridine ^15^ *N*‐oxides are introduced as motifs for hyperpolarization studies and biochemical applications are demonstrated.

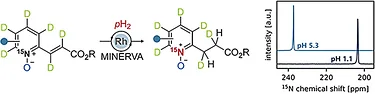

## 1 Introduction

Hyperpolarized magnetic resonance (HP‐MR) is a technique that can overcome the low sensitivity of conventional MR and enhance nuclear magnetic resonance (NMR) signals by several orders of magnitude [1]. This improved sensitivity significantly expands the utility of magnetic resonance (MR), enabling the visualization of metabolic flux in low concentrations in real time [2]. Furthermore, hyperpolarization has essential applications across various areas, including structure characterization, reaction monitoring [3, 4], elucidation of reaction mechanisms [5, 6], biomedical in vitro/cell studies [7], and preclinical research [8, 9, 10]. This considerable potential and range of applications have triggered a rapid development of hyperpolarization methods in recent years. Dissolution dynamic nuclear polarization (d‐DNP) is one of the clinically most developed and widely used hyperpolarization technologies today [11]. Using electron spin as the polarization source, nuclear spins can be hyperpolarized in the solid state at cryogenic temperatures (1–2 K) and in high magnetic fields (3–7 T). A wide range of metabolites and bioactive substances, including ^13^C‐pyruvate [12], have been effectively hyperpolarized with d‐DNP.

An alternative chemically based technique, parahydrogen‐induced polarization (PHIP), uses cost‐efficient, portable, and storable parahydrogen (pH_2_) as the polarization source [13]. pH_2_ is the NMR‐silent spin isomer of molecular hydrogen, characterized by antiparallel nuclear spins of the two protons, resulting in a pure singlet state with effectively 100% spin order. PHIP is attracting increasing attention due to its ability to produce hyperpolarized substances rapidly and affordably [14, 15]. The combination of PHIP with low‐field magnetic resonance imaging (MRI) enables MR sensitization using low‐cost, portable hyperpolarization devices, thereby making MRI more accessible in a broader range of scenarios where expensive devices are unavailable [10].

In non‐hydrogenative PHIP, polarization transfer occurs to substrate molecules via reversible interaction with a metal catalyst that simultaneously binds hydride ligands derived from pH_2_. This process is the Signal Amplification by Reversible Exchange (SABRE) [16]. Hydrogenative PHIP (h‐PHIP) requires an unsaturated bond in the substrate, which can be hydrogenated with pH_2_, and the resulting spin order can then be transferred to nuclei of interest via for example, *J*‐couplings [17, 18]. The commencement of PHIP with side‐arm hydrogenation (PHIP‐SAH) [19] and rapid hyperpolarization/purification techniques [20] made h‐PHIP a more practical technique in a diverse range of biomedical applications. Notably, in a recent study on the hyperpolarization of [1‐^13^C]pyruvate, PHIP‐SAH was shown to yield equivalent results as d‐DNP, rendering PHIP a viable alternative hyperpolarization (HP) technique [18].

**FIGURE 1:**
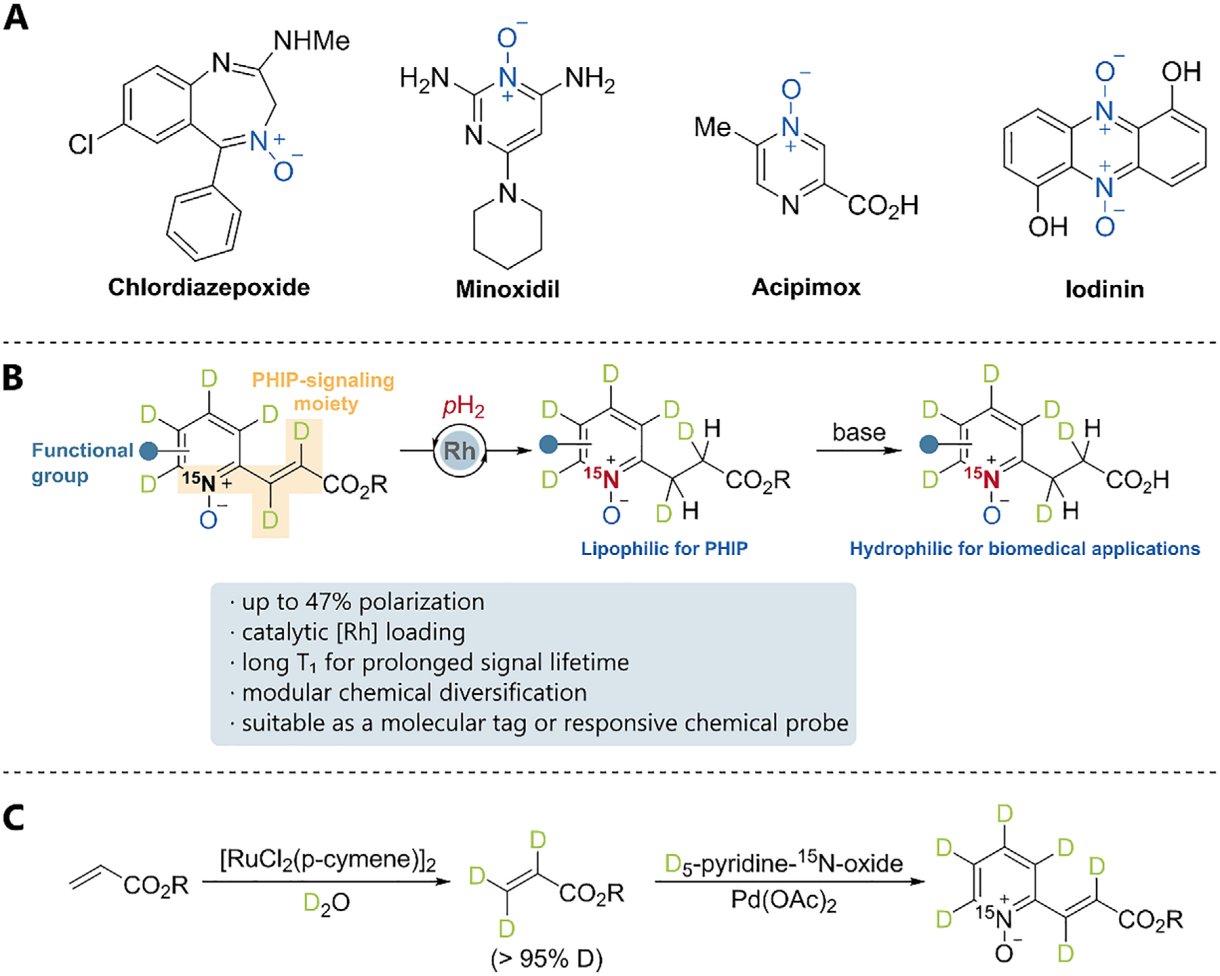
(A) Heterocyclic *N*‐oxide scaffolds in drugs and natural products, (B) Pyridine ^15^ *N*‐oxide as a practical and effective molecular tag for PHIP‐NMR. Note that a lipophilic *N*‐oxide can be used to obtain optimized polarization and workup with base can yield a hydrophilic molecule with increased solubility for biomedical applications. (C) versatile and concise synthesis of isotopically labelled pyridine‐^15^ *N*‐oxide acrylate via transition metal catalyzed H/D exchange and C–D activation.

In parallel with the rapid development of hyperpolarization methods themselves, the development of new MRI molecular probes and contrast agents [21] is also critical to meet the new demands of various MRI research areas. In the past few decades, most HP‐MR contrast agents have been based on ^13^C nuclei due to their longer T_1_ values of 1‐2 min compared to those of ^1^H. Although less sensitive than ^13^C and also less explored in HP, ^15^N nuclei have the following characteristics that make it an attractive candidates for HP‐MR contrast agents: [3, 22, 23, 24] (a) ^15^N containing molecules usually exhibit relative long T_1_ engendering long traceability in vivo; (b) nitrogen containing species play an essential role in various metabolic pathways due to their roles as one of the main elements composing life; (c) ^15^N possesses a wide chemical shift range up to 1350 ppm, making it sensible for minor changes in its chemical environment; (d) the broad availability of ^15^NH_4_Cl and Na^15^NO_3_ facilitates the synthesis of ^15^N isotopically labeled HP‐contrast agents. In our previous work, we reported a ^15^N‐labelled choline derivative with a T_1_ above 20 min that exhibits a detectable HP signal [25]. Following these advancements in ^15^N hyperpolarization, we reported on a ^15^N pyridinium species that exhibits excellent h‐PHIP efficiency and traceability [26], enabling chemo selective, rapid, and responsive detection of reactive oxygen species (ROS) in vitro [27] and at low field [28]. Recently, Wang and Warren et al. successfully leveraged ^15^N_2_‐diazirines as a versatile and long‐lived HP‐MRI tag [23, 29, 30, 31], and realized the biorthogonal cycloaddition with cyclooctyne [32]. Despite various ^15^N‐heterocycles proving to be viable HP contrast agents, pyridine *N*‐oxides, which are low toxic [33] and widely found in natural products [34] and pharmaceuticals (Figure 1A) [35, 36, 37], are rarely reported to be hyperpolarized by any HP technique. In this context, we envisioned that 2‐alkenylpyridine 1‐oxide may be applied as an efficient and valuable h‐PHIP molecular tag or probe (Figure 1B). As an HP contrast agent, the *N*‐oxide moiety could increase the molecule's solubility in H_2_O, an essential property for in vivo application. Additionally, the double bond in acrylate, as the receptor of pH_2_, tends to be more reactive compared to a non‐functionalized C = C, because of the electron‐withdrawing effect of the nearby carbonyl group. Furthermore, the pyridine skeleton facilitates addition of multiple biologically sensitive chemical moieties [38], either on the pyridine ring or on the carboxylic group, which may serve as sensing units for responsive applications.

Herein, we present the first demonstration of hyperpolarized pyridine ^15^ *N*‐oxides, a set of compounds found in natural products and medicines that have never been hyperpolarized by any HP method. Compared with reported h‐PHIP substrates, the current hyperpolarization delivered a high ^15^N HP efficiency with a catalytic amount of rhodium catalyst. A set of isotopically labelled 2‐alkenylpyridine ^15^ *N*‐oxides or 2‐alkenylpyridines with different acrylate side arms or functional groups on the pyridine ring were synthesized and successfully hyperpolarized with h‐PHIP and we showcase several potential applications of the developed molecules.

## 2 Results and Discussion

**FIGURE 2:**
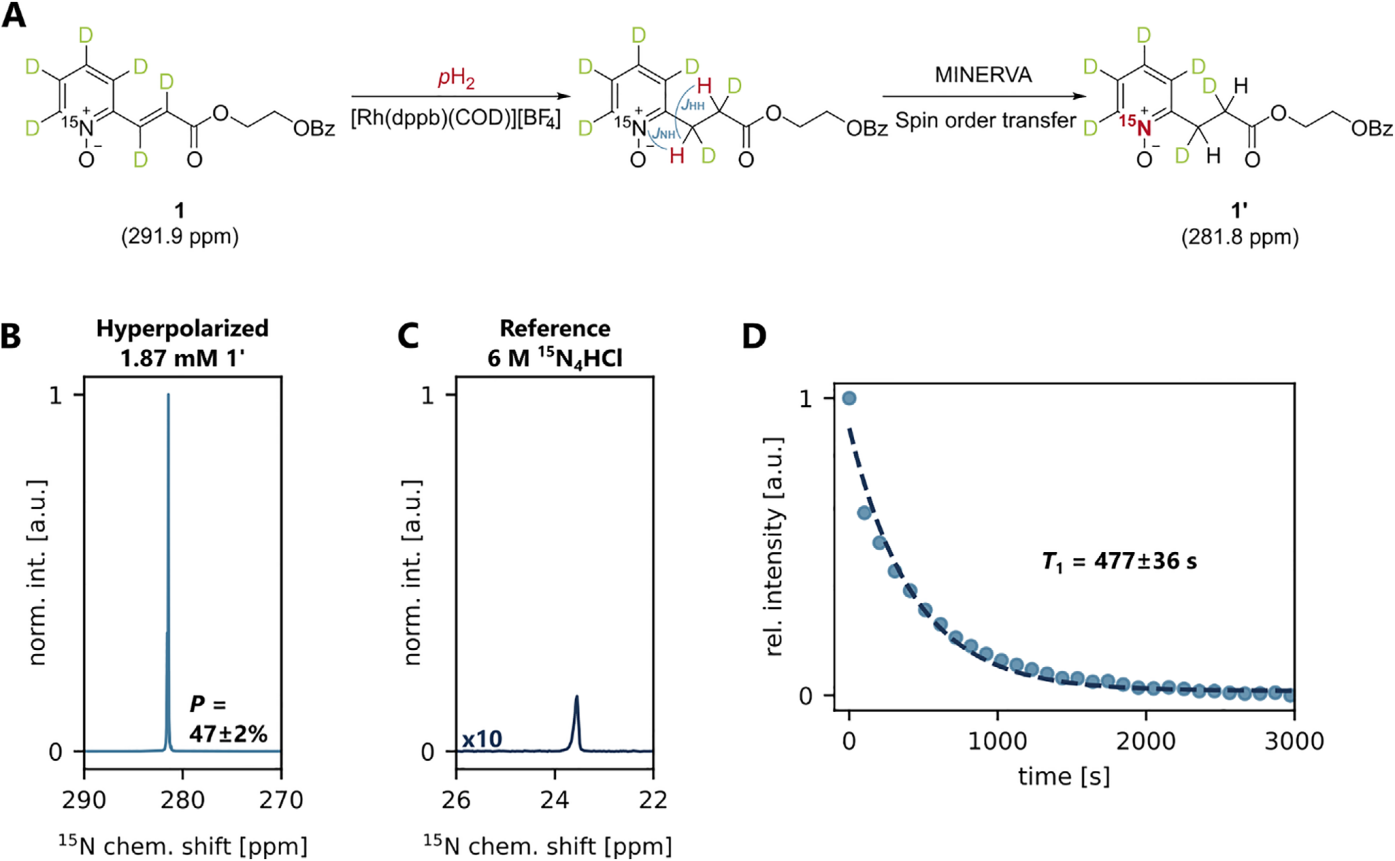
(A) Hydrogenative parahydrogen‐induced hyperpolarization of 2‐alkenylpyridine *N*‐oxide 1. (B) single 90° pulse ^15^N scan of hyperpolarized **1′** (1.87 mM) and (C) single scan thermal spectrum of ^15^NH_4_Cl (6.0 M) for comparison. (D) T_1_ of **1′**, measured with 10° low flip angle pulses in MeOD‐*d*_4_ at 0.1 T.

We initiated our study by focusing on ^15^N/D‐labelled 2‐alkenylpyridine ^15^ *N*‐oxide to investigate its potential as a molecular tag for h‐PHIP. By employing a ruthenium‐catalyzed deuteration strategy developed by Ackermann [39], D_3_‐labeled substituted acrylates were readily prepared. Then, the palladium‐catalyzed C–D activation [40], disclosed by Chang [41], delivered the desired isotopically labelled 2‐alkenyl pyridine ^15^ *N*‐oxides (Figure 1C). We use 2‐alkenylpyridine ^15^ *N*‐oxide **1** as the model substrate to perform hydrogenation reactions with pH_2_ and to explore subsequent spin‐order transfer to the heteronuclear ^15^N (Figure 2 and Table S1). As in previous studies, a homogeneous rhodium complex [Rh(dppb)(COD)][BF_4_] (dppb: diphenylphosphino butane, COD: cyclooctadiene) was used as the catalyst, and MeOD‐*d*_4_ was employed as the reaction medium. Using the MINERVA (maximizing insensitive nuclei enhancement reached via parahydrogen amplification) [42, 43, 44] sequence, ^15^N polarization was achieved with good efficiency, with 1.0 equivalent of rhodium catalyst utilized (entry 1 in Table S1). Next, we observed that the ^15^N polarization efficiency was significantly improved as the rhodium catalyst loading decreased (entries **2**–**6**). A decent ^15^N polarization efficiency was still achieved even at low catalyst loading of 0.5 mol%, demonstrating the excellent reactivity of 2‐alkenylpyridine ^15^ *N*‐oxide (entries **7**–**9**). It is worth mentioning that, in previous PHIP studies, most substrates required a stoichiometric to sub‐stoichiometric amount of rhodium catalyst [27]. After further experimentation with different temperatures (entries **11**–**12**), pH_2_ bubbling time (entries **13**–**14**), and pulse sequence delay (entries **15**–**16**), we finally determined the optimal h‐PHIP conditions (entry **10**), and 47% ± 2% ^15^N polarization efficiency was realized at 7 T as demonstrated by comparison to the thermal ^15^N‐NMR spectrum of ^15^NH_4_Cl in D_2_O (Figure 2). Note that reliable determination of the hydrogenation conversion was not possible under the experimental conditions due to the low precursor concentrations and insufficient signal‐to‐noise ratio in the thermal ^1^H NMR spectra which may be partly due to occurring hydrogen/deuterium exchange. The polarization calculations therefore, assume full conversion of the reactants, and the reported signal enhancement levels represent conservative estimates.

With the hyperpolarization of 2‐alkenylpyridine ^15^ *N*‐oxide **1** established, we subsequently investigated the versatility of this protocol (Table 1). Therefore, 2‐alkenylpyridine ^15^ *N*‐oxide **2**, bearing a 2‐(2‐ethoxyethoxy)ethan‐1‐ol hydrophilic tail, was synthesized and tested under optimal PHIP conditions. A good ^15^N polarization efficiency of 18% ± 2% was observed. Furthermore, the hydrophobic phenyl‐ethyl‐substituted 2‐alkenylpyridine ^15^ *N*‐oxide **3** was also a viable substrate for h‐PHIP, delivering a ^15^N polarization efficiency of up to 28% ± 2%. The fully deuterated, smaller 2‐alkenylpyridine *N*‐oxide **4** exhibited excellent reactivity, affording a ^15^N polarization efficiency of up to 24% ± 2%. It is noteworthy that the challenging carboxylic acid or hydroxy‐substituted 2‐alkenylpyridine *N*‐oxide (S‐5, S‐6, see Supporting Information) is also amenable to this PHIP protocol, although the ^15^N polarization efficiency was slightly lower than that of its analogs. The marginally lower efficiency may be due to the rhodium catalyst being partially deactivated by coordination to the excess carboxylic acid or hydroxy group.

**TABLE 1:** Pyridine‐^15^ *N*‐oxide acrylate exhibits excellent PHIP efficiency and functional group comparability.

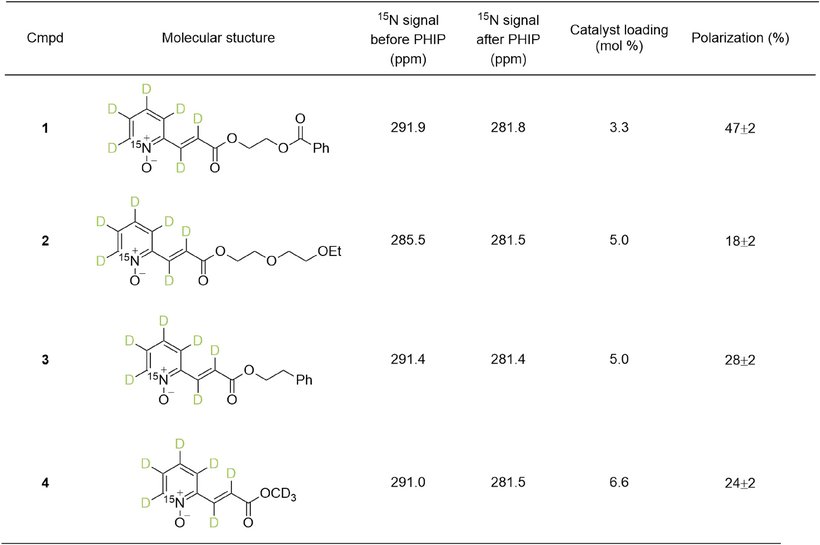

Standard hyperpolarization conditions: Pyridine‐^15^ *N*‐oxide (2.5 mM) and [Rh(dppb)(COD)][BF_4_] (3.3–6.6 mol%) in MeOD‐*d*_4_ (0.2 mL). pH_2_ bubbling time = 9 s, 335 K, *J*_HH _= 7.1 Hz, *J*_NH _= 3.75 Hz.

**FIGURE 3:**
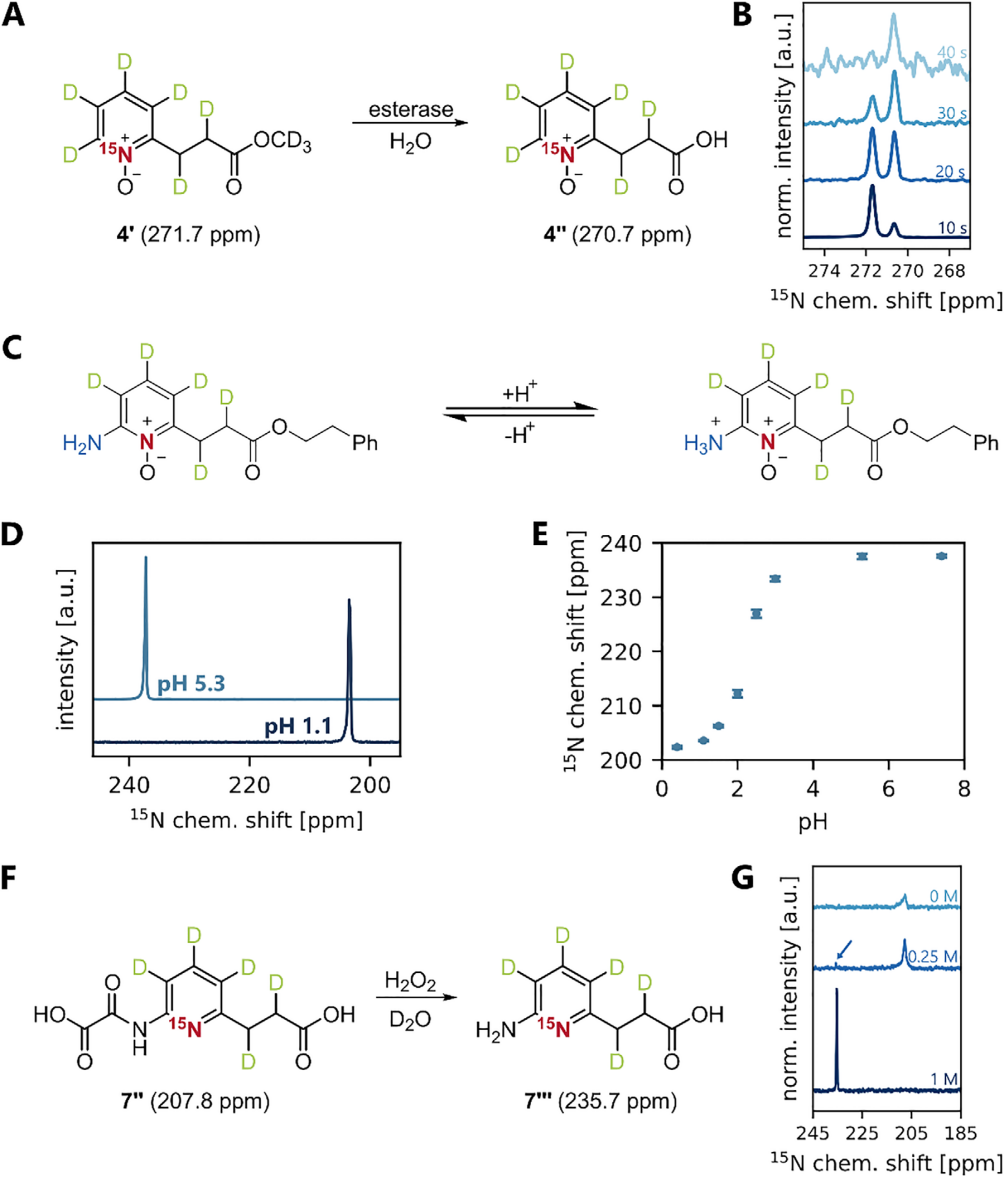
(A) Hydrolysis of hyperpolarized **4′** via esterase. (B) Series of ^15^N‐NMR spectra acquired upon mixing the esterase containing solution (300 U) with the aqueous solution of hyperpolarized **4′**. Spectra were acquired using flip angle pulse of 30 and 10 s delays between successive scans. (C‐E) Hyperpolarized *N*‐oxide **6′** is employed in pH measurement. (C) Mechanism of the pH sensitive probe, (D) Spectra of the chemical shifts at pH 1.1. and pH 5.3, (E) ^15^N chemical shift distribution in various aqueous pH environments. (F) H_2_O_2_ promoted oxidative cleavage of hyperpolarized **7′′** in neutral aqueous media, (G) Cleavage of the ROS reporter moiety leads to a change in chemical shift in water.

To further illustrate the potential of 2‐alkenylpyridine ^15^ *N*‐oxides as h‐PHIP chemical probes, a series of structural derivatives was synthesized (Table 2), and a set of applications with relevance to diseases were investigated (Figure 3). Compounds **1–4** contain an ester moiety that has potential to be cleaved in enzymatic reactions with esterases, whereby esterases have been linked to metabolic diseases [45]. Compound **1** can be reduced to **5** and, together with compound **6**, forms a set of moieties of interest for pH sensing due to the possibility of proton exchange near the ^15^N nucleus. Capabilities to measure pH are of interest to study extracellular pH, which is a hallmark for many cancers due to a reverse pH gradient compared to healthy tissue [46]. Compound **7** was synthesized with a ketoacid ester moiety that we expect to be cleaved in the presence of reactive oxygen species (ROS), such as hydrogen peroxide. ROS is a relevant redox component to maintain cell homeostasis and elevated levels are another hallmark of cancer [47]. In addition, a series of *para*‐nitro‐substituted 2‐alkenylpyridine ^15^ *N*‐oxides (**8–10**) were synthesized. These compounds provide a structural basis for detecting nitroreductases and are theoretically capable of functioning as chemical‐shift‐dependent ^1^ ^5^N MRI probes. We envision that optimized molecules can be activated upon reduction of the nitro group to an amino group, thereby reporting nitroreductase (NTR) activity in vivo. Notably, NTR serves as a key biomarker for evaluating tumor hypoxia [48], suggesting that nitro‐substituted 2‐alkenylpyridine ^15^ *N*‐oxide 8‐10 could offer a promising platform for probing tumor microenvironments and monitoring hypoxic progression [49, 50]. The obtained levels of polarization for these compounds are presented in Table 2 together with the chemical shift differences of the ^15^N signal before and after hydrogenation. We note that for the nitro‐substituted compounds, the ^15^N chemical shift was located at 306 ppm in acetone‐*d*_6_, while the signal appeared at 300 ppm in MeOD‐*d*_4_. This behavior may be attributed to the reduction of the ^15^ *N*‐oxide moiety to free pyridine species in MeOD‐*d*_4_ under the hydrogenation conditions. While all of the compounds show hyperpolarization in organic solvents, we then investigated the transfer into water to pursue biological applications (see Supporting Information). Briefly, buffer is added to the hyperpolarized organic solution and the organic solvent removed by vacuum. The aqueous solution was filtered to remove residual catalyst. Likely due to solubility reasons, no signal could be detected for the nitro‐substituted compounds **8–10**. Compounds **1–4** showed water solubility whereas out of the investigated derivatives, Compound **2** appears to be most soluble in water due to the hydrophilic tail. Following transfer into aqueous solution, we observed notable differences in the achieved hyperpolarization levels among the investigated compounds. Compound 2′ showed the highest polarization (4.0% ± 0.5%), followed by 4′ (1.6% ± 0.3%) and 6′ (0.9% ± 0.1%) measured at concentrations in the range of 10–20 mM depending on the compound and estimated using the starting data point of the T_1_ experiments (see Supporting Information). The polarization values assume full conversion and therefore represent conservative estimates. Comparison with the corresponding T_1_ values (Table 3) reveals that these differences cannot be explained by relaxation alone. This suggests that additional dynamic effects during hydrogenation and polarization transfer, such as substrate–catalyst interactions, differences in temperature, pH or differences in spin dynamics, play a significant role. A detailed understanding of these processes will be the subject of future investigations. At concentrations above about 20 mM, compound **2**′ could be directly extracted into water, whereas the more lipophilic molecules **4′** and **6′** needed to be hydrolyzed with base to be sufficiently water soluble. Compounds **1** and **3** display similar solubility behavior as compound **4** due to the lipophilic chains and need to be cleaved to obtain higher concentrations in water resulting in essentially the same cleaved ester as for **4′**. Following the similar cleavage approach, the cleaved ester of compound **7** could be obtained in an aqueous medium. For potential applications in aqueous buffer, we focused on compound **4′**, as well as the cleaved esters of **6′** and **7′**. With hyperpolarized ^15^ *N*‐oxide (17.2 mM concentration) **4′**, we successfully observed ester hydrolysis catalyzed by esterase from porcine liver (150 or 300 U) in a biocompatible H_2_O environment by NMR spectroscopy (Figure 3A, B). Studies of the cleaved ester of Compound 6′ confirmed that hydrogenated pyridine‐^15^ *N*‐oxide functions as a pH sensor, exhibiting high sensitivity under acidic conditions (from pH ≈ 1 to 5). Prior to the pH measurements, methanol was removed almost completely, and the remaining methanol content of 400 ± 200 (about 1.2 wt%), (Figure S63) is considered negligible with respect to the pH measurements as reported in the literature [51]. A pronounced ^1^ ^5^N chemical shift variation of 33.8 ppm was observed as the pH changed from 1.1 to 5.3. The large range of chemical shifts compared to the pH suggests the agent could be used in contexts of low pH, like the assessment of gastric acidity. (Figure 3C–E). We note that during the pH measurements strong fluctuations of the signal intensity were observed which we attribute to the above described mechanism that also lead to strong losses in polarization when removing methanol from the solution. The amide derivative **7′′** indeed showed responsiveness to H_2_O_2_ in neutral aqueous media (Figure 3F, G) showing that the attached keto acid derivative can be made labile that CO_2_ is released upon interaction with reactive oxygen species (ROS) [52, 53, 54], such as hydrogen peroxide (H_2_O_2_).

**TABLE 2:** Structure diversification demonstrates the ^15^ *N*‐oxides potential for responsive molecular tags.

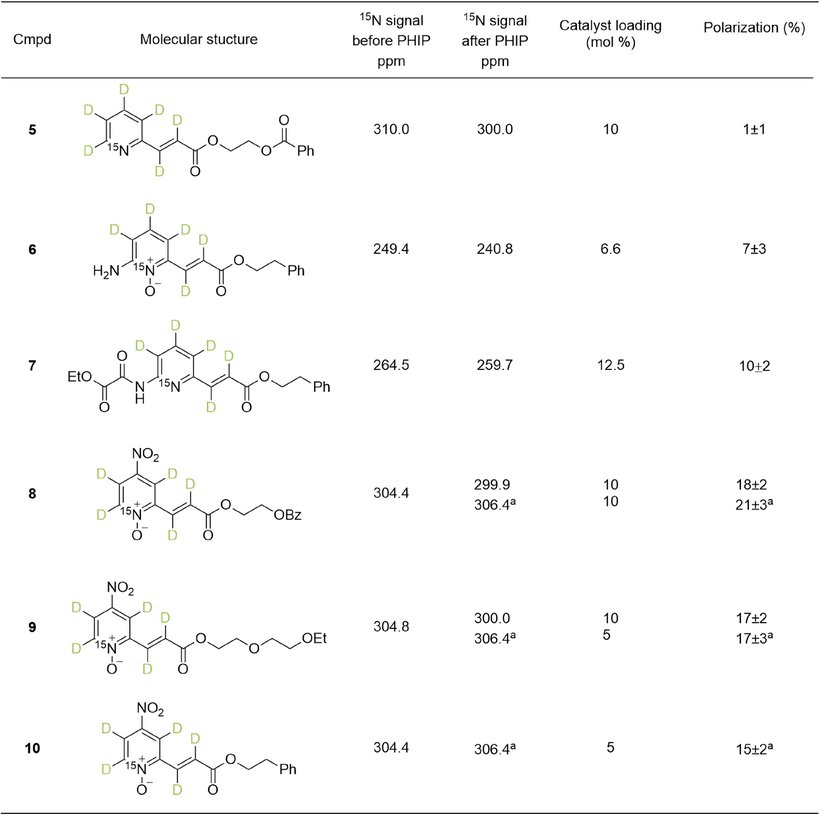

Standard hyperpolarization conditions: Pyridine‐^15^ *N*‐oxide (2.5 mM) and [Rh(dppb)(COD)][BF_4_] (5∼10 mol%) in MeOD‐*d*_4_ (0.2 mL). pH_2_ bubbling time = 9 s, 335 K, JHH = 7.1 Hz, JNH = 3.75 Hz. ^a^ in acetone‐*d*_6_ and pH_2_ bubbling time = 12 s.

**TABLE 3:** T_1_ in different magnetic fields and solvents.

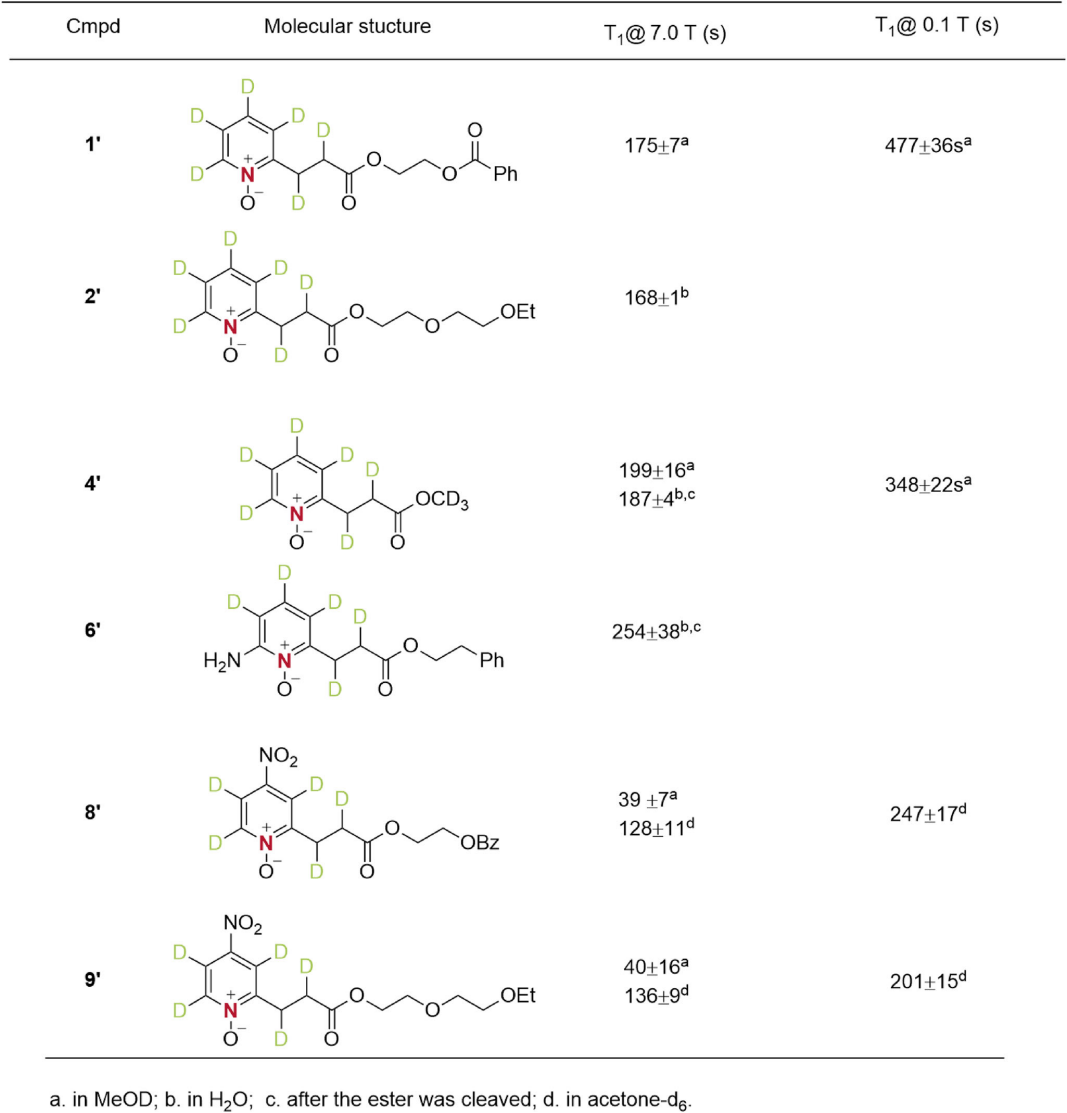

Encouraged by the robustness and diverse potential applications of 2‐alkenylpyridine *N*‐oxides as hydrogenative PHIP chemical probes or molecular tags, we next investigated their spin‐lattice relaxation time (T_1_), which reflects their detectability after hyperpolarization (Table 3). For *N*‐oxide **1′**, the T_1_ of ^15^N was measured to be 175 ± 7 s at 7.0 T in MeOD‐*d*_4_ at 335 K. Notably, the T_1_ increased with decreasing magnetic field strength, reaching 477 ± 36 s at 0.1 T. Comparing the T_1_ data of **1′** and **4′** revealed that, although **4′** exhibits a slightly longer T_1_ at 7.0 T (199 ± 16 s)—likely due to complete deuteration of the whole molecule and its smaller molecular size—its T_1_ at 0.1 T (348 ± 22 s) was shorter than that of **1′**. For the nitro‐substituted derivatives **8′** and **9′**, their short T_1_ values observed in MeOD‐*d*_4_ are likely attributable to the formation of free pyridine species in this solvent. This interpretation is consistent with previous reports indicating that free pyridine generally exhibits relatively rapid relaxation [55]. In contrast, longer T_1_ values were observed in acetone‐*d*_6_, where the distinct chemical shift and T_1_ differences (306 vs. 300 ppm) indicate that, the ^15^ *N*‐oxide functionality remains intact during hydrogenation in acetone‐*d*_6_. To evaluate the detectability of our HP molecules in H_2_O, which is the foundation for practical utility in biocompatible environments, we measured the T_1_ data of ^15^ *N*‐oxide **2′**, **4′**, and **6′**. All of them exhibit comparable T_1_ value (from 168 ± 1 to 254 ± 38 s) in H_2_O with a physiological or neutral pH, as in organic solvents. These findings provide a valuable design principle for future ^1^ ^5^N hyperpolarization probes: Protecting the ^1^ ^5^N site as a quaternary ammonium or ^15^ *N*‐oxide moiety can substantially prolong T_1_ compared to the corresponding free amine/pyridine analogs. The findings that the described probes possess a particular long T_1_ at lower field suggest that the described class of compounds is particularly suitable for benchtop and low field NMR and MRI applications that have generated a lot of interest even clinically in recent years [56]. Our recent demonstration that chemical reactions can be probed with ^15^N probes at fields even below 100 mT served as a proof of concept and supports the pursuit of designing novel ^15^N‐based molecular probes [28].

## 3 Conclusion

In conclusion, we have demonstrated the successful hyperpolarization of pyridine ^15^ *N*‐oxides, a class of compounds so far not explored with HP, as a new family of ^15^ *N*‐labeled tracers for hyperpolarized magnetic resonance (HP‐MR) via h‐PHIP. These probes exhibit remarkable robustness, long‐lived polarization, sensitive functional group tolerance, and high structural versatility. Their utility is further highlighted by the ease of structural modification, enabling diverse potential applications. Our experiments demonstrated that the ortho‐amine‐substituted pyridine ^15^ *N*‐oxide could serve as a practical pH indicator for monitoring, that is, gastric acidity, exhibiting high sensitivity under acidic conditions (pH ≈ 2). We demonstrated the use of the proposed compounds in detecting hydrogen peroxide as a reactive oxygen species and we demonstrate that, the *N*‐oxides can be used to monitor enzymatic reactions. Furthermore, we discuss para‐nitro‐substituted pyridine ^15^ *N*‐oxides, which are theoretically capable of serving as probes for nitroreductase (NTR) activity evaluation, a key biomarker of tumor hypoxia. While, further optimization is required to fully translate these systems into biological use, this work establishes 2‐alkenylpyridine ^15^ *N*‐oxides as promising molecular tags with excellent hyperpolarization properties and responsive chemical probes, with significant potential for diagnostic and preclinical HP‐MR studies in the near future.

## Conflicts of Interest

Stefan Glöggler is a co‐founder of MagniKeen all other authors declare no financial interest.

## Supporting information




**Supporting File 1**: anie73462‐sup‐0001‐SuppMat.pdf.

## Data Availability

The data that support the findings of this study are available in the Supporting Information of this article
