# Supplementary material for: Hyperpolarization of Pyridine 15 N‐Oxide Molecular Probes Enabled by Parahydrogen
Source: Angew Chem Int Ed Engl. 2026 Jul 9;65(37):e25716. doi: 10.1002/anie.202525716 (PMC13549037; doi:10.1002/anie.202525716)
Supplement: Supplementary file 1 [file ANIE-65-e25716-s001.pdf]

Supporting Information

Hyperpolarization of Pyridine ^{15}N-Oxide Molecular Probes Enabled by Parahydrogen

Ruhuai Mei, Lisa Maria Fries, Gonzalo Gabriel Rodriguez, Charlotte von Petersdorff-Campen, Leander Konstantin May, Julius Frederik Matz, Sergey Korchak, Grysette Daher, Stefan Glöggler*

stefan.gloeggler@utsouthwestern.edu.de

Contents

1. Synthetic procedures 3

1.1 General remark 3

1.2 Synthetic protocols and compounds characterization 3

2. Parahydrogen-induced polarization (PHIP) experiments 14

2.1 NMR instrumentation used 14

2.2 Sample & nuclear spin order preparation 14

2.3 General remark for ^{15}N hyperpolarization 15

2.4 The ^{15}N-NMR thermal spectrum of $^{15}NH_4Cl$ 15

2.5 ^{15}N-PHIP parameter optimization 18

2.6 ^{15}N-hyperpolarization spectra 19

2.7 T_1 field dependence for -pyridine ^{15}N-oxide derivatives 60

2.8 Hyperpolarized amine **6'** employed in pH measurement. 64

3. Hyperpolarization with aqueous workup 67

3.1 T_1 measurement in biocompatible aqueous media 66

3.2 Biochemically relevant transformation in aqueous media 68

3.3 Quantification of residual methanol concentration after workup 74

4. NMR & HR-MS spectra for compounds characterization 75

5. Reference 107

1 Synthetic procedures

1.1 General remarks

Chemicals were purchased from commercial suppliers and were used without further purification. pyridine ^{15}N-oxide-d$_5$,[1] were prepared analogously with the reported procedures. Thin-layer chromatography (TLC) was performed on aluminum-backed plates, and compounds were visualized under UV light. Silica gel (230-400 mesh, 60 Å) was purchased from Sigma Aldrich and used for flash chromatography. Esterase from porcine liver (18 units/mg solid, CAS: 9016-18-6) was purchased from Sigma Aldrich. ^{1}H, ^{2}H, ^{13}C and ^{15}N NMR spectra were recorded on Bruker Avance III HD spectrometers at 7 T. Further details on the instrumentation can be found below in the section "NMR Instrumentation used". The signals of the deuterated solvents were used as internal chemical shift standards. Residual proton signals from the deuterated solvents were used as references [D_2O (4.81 ppm), $CDCl_3$ (7.26 ppm), methanol-*d*$_4$ (3.31 ppm)] for ^{1}H spectra. The residual ^{13}C signals from the deuterated solvents being used as references [$CDCl_3$ (77.0 ppm), methanol-*d*$_4$ (49.05 ppm)] for ^{13}C spectra. All coupling constants were measured in Hertz. All moisture-sensitive reactions were carried out in oven-dried glassware using nitrogen industrial-grade cylinders. High resolution mass spectra were recorded on a Thermo LTQ Orbitrap XL (ESI$^+$) or a P-SIMS-Gly of Bruker Daltonics Inc (EI$^+$).

1.2 Synthetic protocols and compounds characterization

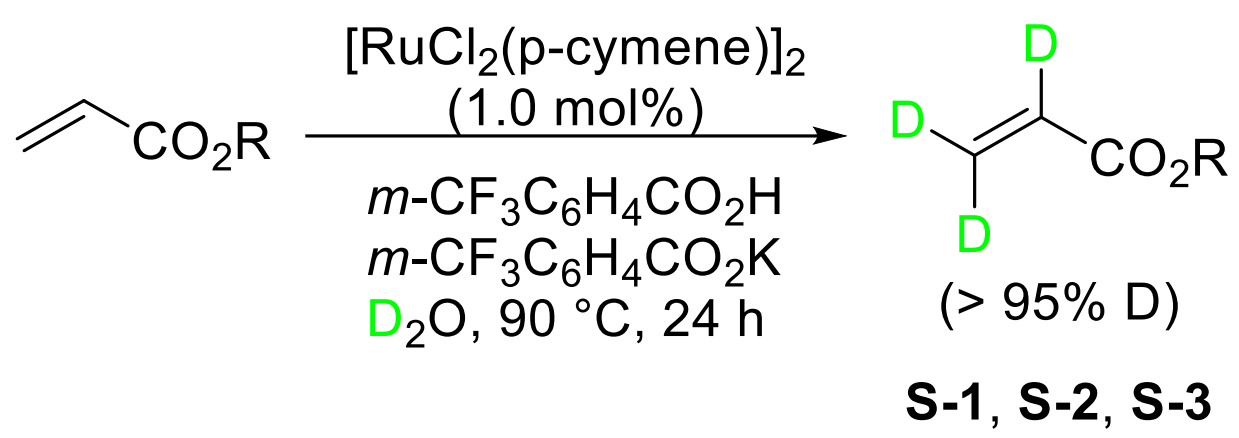

Scheme S-a. Versatile synthesis of [D$_3$]-acrylate *via* C−D exchange.

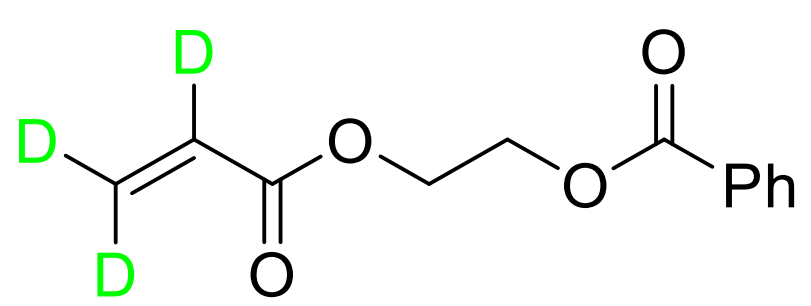

2-[(Acryloyl-d3)oxy]ethyl benzoate (S-1)

Following a reported procedure,[2] $[RuCl_2(p\text{-}cymene)]_2$ (255 mg, 0.41 mmol, 1.0 mol %), 3-CF_3-benzoic acid (198 mg, 1.04 mmol, 5.0 mol %) and potassium 3-CF_3-benzoate (238 mg, 1.04 mmol, 5.0 mol %) were placed in a predried 100 mL Schlenk tube equipped with a rubber septum. The tube was evacuated and backfilled with Ar three times. Acrylic ester (4.6 g, 20.9 mmol, 1.0 equiv) and D_2O (10.6 g, 25.3 equiv) were added, and the mixture was heated to 90 °C for 18 h. At ambient temperature, the aqueous mixture was extracted with CH_2Cl_2 (3 × 10 mL). The combined organic phase was dried with Na_2SO_4 and concentrated in vacuo. The crude product was further purified by column chromatography, delivering the desired product 2-[(Acryloyl-d_3)oxy]ethyl benzoate **S-1** (3.196 g, 68%) as a colorless oil. **^{1}H NMR** (300 MHz, $CDCl_3$) δ = 7.98 (d, J = 7.5 Hz, 2H), 7.48 (t, J = 7.4 Hz, 1H), 7.35 (t, J = 7.6 Hz, 2H), 4.64–4.33 (m, 4H). **^{2}H NMR** (46 MHz, $CDCl_3$) δ = 6.39 (s, 1D), 6.12 (s, 1D), 5.80 (s, 1D). **^{13}C NMR** (75 MHz, $CDCl_3$) δ = 165.90, 165.53, 132.80, 130.31, 129.52, 129.35, 128.08, 127.25, 62.37, 61.94. **HR-MS** (ESI) m/z calcd for $C_{12}H_{10}D_3O_4$ $[M + H]^+$ 224.0997, found 224.0991; $C_{12}H_9D_3O_4Na_1$ $[M + Na]^+$ 246.0816, found 246.0828.

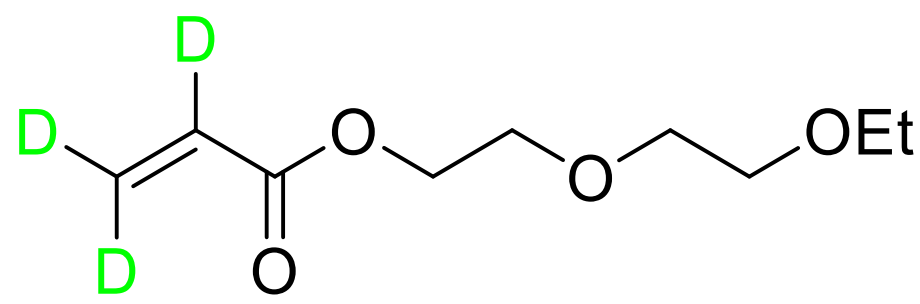

2-(2-Ethoxyethoxy)ethyl acrylate-d_3 (S-2)

Following a reported procedure,[2] $[RuCl_2(p\text{-}cymene)]_2$ (691 mg, 1.13 mmol, 1.0 mol %), 3-CF_3-benzoic acid (722.5 mg, 3.8 mmol, 5.0 mol %) and potassium 3-CF_3-benzoate (867 mg, 3.8 mmol, 5.0 mol %) were placed in a predried 100 mL Schlenk tube equipped with a rubber septum. The tube was evacuated and backfilled with Ar three times. 2-(2-Ethoxyethoxy)ethyl acrylate (14.3 g, 75.8 mmol, 1.0 equiv) and D_2O (38 g, 1881 mmol, 24.8 equiv) were added, and the mixture was heated to 90 °C for 18 h. At ambient temperature, the aqueous mixture was extracted with CH_2Cl_2 (3 × 40 mL). The combined organic phase was dried with Na_2SO_4 and concentrated in vacuo. The crude product was further purified by column chromatography, delivering the desired product 2-(2-Ethoxyethoxy)ethyl acrylate-d_3 **S-2** (10.87 g, 75%) as a colorless oil. **^{1}H NMR** (300 MHz, MeOD) δ = 4.30-4.27 (m, 2H), 3.81–3.39 (m, 8H), 1.17 (t, J = 7.1 Hz, 3H). **^{13}C NMR** (75 MHz, MeOD) δ = 168.07, 131.82, 129.75, 72.28, 71.59, 70.76, 68.22, 65.49, 16.34. **^{2}H NMR** (46 MHz, MeOD) δ = 6.44 (s, 1D), 6.22 (s, 1D), 5.94 (s, 1D). **HR-MS** (ESI) m/z calcd for $C_9H_{14}D_3O_4$ $[M + H]^+$ 192.1310, found 192.1317; $C_9H_{13}D_3O_4Na_1$ $[M + Na]^+$ 214.1129, found 214.1135.

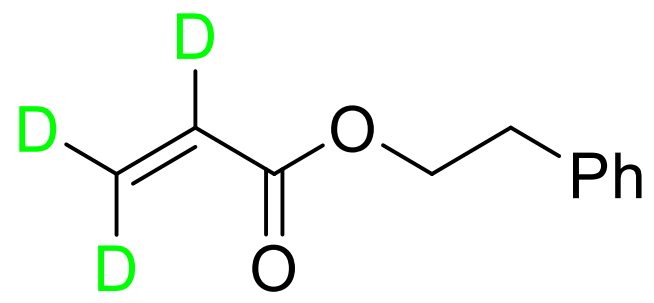

Phenethyl acrylate-d3 (S-3)

Following a reported procedure,[2] $[RuCl_2(p\text{-cymene})]_2$ (434 mg, 0.71 mmol, 1.0 mol %), 3-CF_3-benzoic acid (671 mg, 3.52 mmol, 5.0 mol %) and potassium 3-CF_3-benzoate (805 mg, 3.52 mmol, 5.0 mol %) were placed in a predried 100 mL Schlenk tube equipped with a rubber septum. The tube was evacuated and backfilled with Ar three times. Phenethyl acrylate (12.5 g, 70.6 mmol, 1.0 equiv) and D_2O (45 g, 2.24 mol, 31.8 equiv) were added, and the mixture was heated to 90 °C for 18 h. At ambient temperature, the aqueous mixture was extracted with CH_2Cl_2 (50 mL × 3). The combined organic phase was dried with Na_2SO_4 and concentrated in vacuo. The crude product was further purified by column chromatography, delivering the desired product phenethyl acrylate-d3 **S-3** (8.31 g, 46.3 mmol, 65%) as a colorless oil. **^{1}H NMR** (300 MHz, $CDCl_3$) δ = 7.49–7.10 (m, 5H), 4.41 (t, J = 7.1 Hz, 2H), 3.01 (t, J = 7.1 Hz, 2H). **^{2}H NMR** (46 MHz, $CDCl_3$) δ = 6.44 (s, 1D), 6.17 (s, 1D), 5.84 (s, 1D). **^{13}C NMR** (75 MHz, $CDCl_3$) δ = 165.70, 137.51, 129.66, 128.63, 128.22, 127.73, 126.29, 64.67, 34.81. **HR-MS** (ESI) m/z calcd for $C_{11}H_9D_3O_2Na_1$ $[M + Na]^+$ 202.0918, found 202.0926.

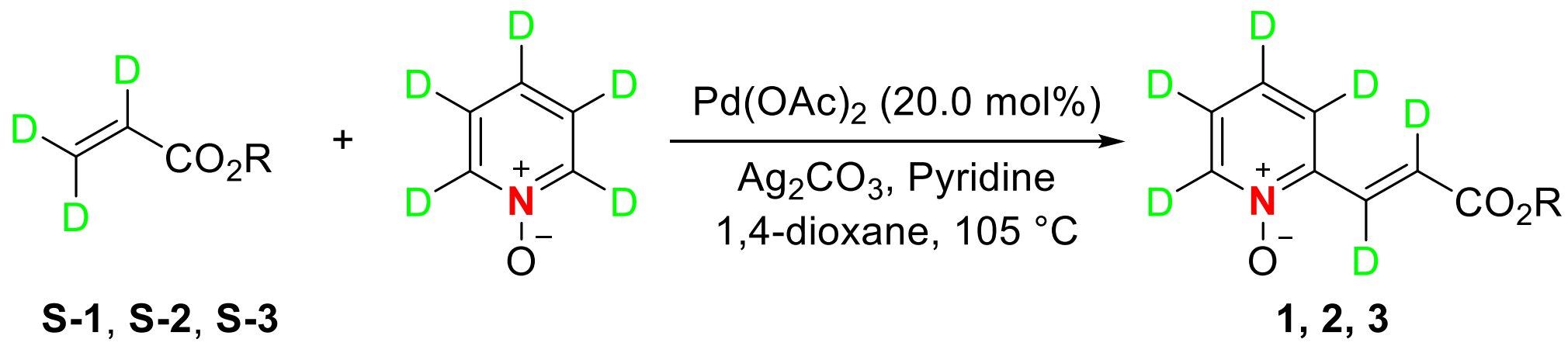

Scheme S-b. Versatile and concise synthesis of pyridine-^{15}N-oxide acrylate *via* C−D activation.[3]

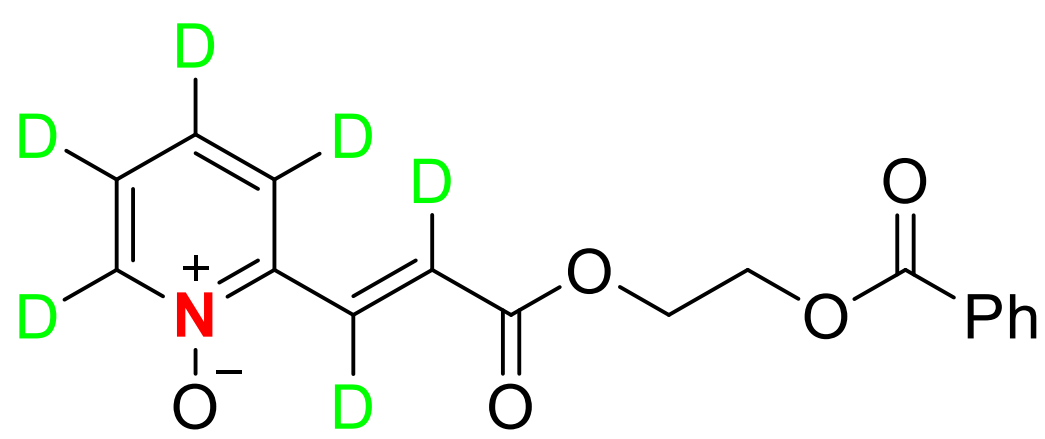

(E)-2-{3-[2-(Benzoyloxy)ethoxy]-3-oxoprop-1-en-1-yl-1,2-d2}pyridine 1-oxide-3,4,5,6-d4 (1)

A mixture of $Pd(OAc)_2$ (231 mg, 20 mol %), Ag_2O (2.98 g, 2.5 equiv) and pyridine N-oxide (1.446 g, 14.3 mmol, 2.77 equiv) was weighed into a 50 mL screw-capped vial equipped with a 10 x 5 mm spinvane triangular-shaped Teflon stirbar. 1,4-Dioxane (10 mL) and pyridine (406 mg, 5.15 mmol, 1.0 equiv) were added, followed by ethyl acrylate-d_3 **S-1** (1.15 g, 5.15 mmol, 1.0 equiv). The resulting mixture was sealed with a Teflon-lined cap and stirred at 105 °C for 48 h in an oil bath. The reaction mixture was cooled to room temperature, diluted with EtOAc (10 mL), and filtered through a Celite pad. The filtrate was concentrated and purified by column chromatography, delivering the desired pyridine-^{15}N-oxide acrylate **1** as a pale yellow solid (717 mg, 43%). **^{1}H NMR** (300 MHz, $CDCl_3$) δ = 7.89 (d, J = 7.5 Hz, 2H), 7.40 (t, J = 7.4 Hz, 1H), 7.28 (t, J = 7.6 Hz, 2H), 4.44 (s, 4H). **^{2}H NMR** (46 MHz, $CDCl_3$) δ = 8.10–7.98 (m), 7.60–6.91 (m). **^{13}C NMR** (75 MHz, $CDCl_3$) δ = 165.78, 165.59, 144.17 (d, $^1J_{C\text{-}N}$ = 15.0 Hz), 139.58 (d, $^1J_{C\text{-}N}$ = 15.6 Hz), 133.84, 132.71, 129.26, 129.19, 127.96, 125.40, 125.16, 124.18 (d, $^2J_{C\text{-}N}$ = 4.4 Hz), 123.08 (d, $^2J_{C\text{-}N}$ = 2.2 Hz), 62.24, 62.19. **^{15}N NMR** (30 MHz, $CDCl_3$) δ = 291.94 (s). **HR-MS** (ESI) *m/z* calcd for $C_{17}H_{10}D_6{}^{15}N_1O_5$ $[M + H]^+$ 321.1370, found 321.1375; $C_{17}H_9D_6{}^{15}N_1O_5Na_1$ $[M + Na]^+$ 343.1189, found 343.1204.

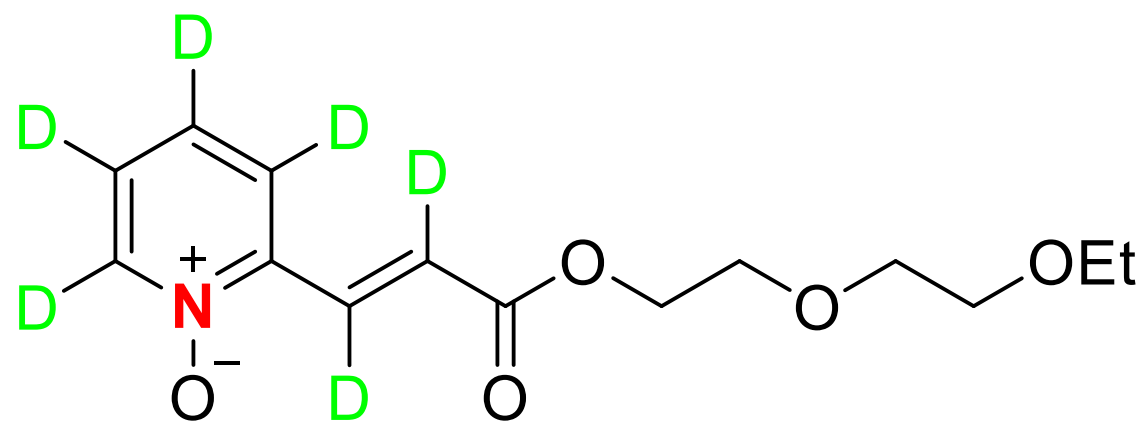

(E)-2-{3-[2-(2-Ethoxyethoxy)ethoxy]-3-oxoprop-1-en-1-yl-1,2-d_2}pyridine 1-oxide-3,4,5,6-d_4 (2)

A mixture of $Pd(OAc)_2$ (195 mg, 10 mol %), Ag_2CO_3 (4.78 g, 2.0 equiv) and pyridine N-oxide (877 mg, 8.7 mmol, 1.0 equiv) was weighed into a 100 mL screw-capped vial equipped with a 10 x 5 mm spinvane triangular-shaped Teflon stirbar. 1,4-Dioxane (17.4 mL, 0.5 M) and pyridine (690 mg, 8.7 mmol, 1.0 equiv) were added, followed by ethyl acrylate **S-2** (2.49 g, 13 mmol, 1.49 equiv). The resulting mixture was sealed with a Teflon-lined cap and stirred at 105 °C for 48 h in an oil bath. The reaction mixture was cooled to room temperature, diluted with EtOAc (10 mL), and filtered through a Celite pad. The filtrate was concentrated and purified by column chromatography, delivering the desired pyridine-^{15}N-oxide acrylate **2** as a pale yellow solid (821 mg, 37%). **^{1}H NMR** (300 MHz, MeOD) δ = 4.44–4.14 (m, 2H), 3.85–3.39 (m, 8H), 1.19–1.12 (q, J = 6.7 Hz, 3H). **^{2}H NMR** (46 MHz, MeOD) δ = 8.05–7.09 (m, 6D).

^{13}**C NMR** (75 MHz, MeOD) δ = 167.07, 145.75 (d, J = 15.1 Hz), 140.93 (d, J = 15.5 Hz), 134.31, 128.20 (d, J = 4.7 Hz), 127.55, 126.91, 125.50 (d, J = 2.4 Hz), 71.42, 70.71, 69.85, 67.31, 65.14, 15.53. ^{15}**N NMR** (30 MHz, MeOD) δ = 285.50 (s).**HR-MS** (ESI) m/z calcd for $C_{14}H_{14}D_6{}^{15}N_1O_5$ $[M + H]^+$ 289.1683, found 289.1687; $C_{14}H_{13}D_6{}^{15}N_1O_5Na_1$ $[M + Na]^+$ 311.1502, found 311.1515.

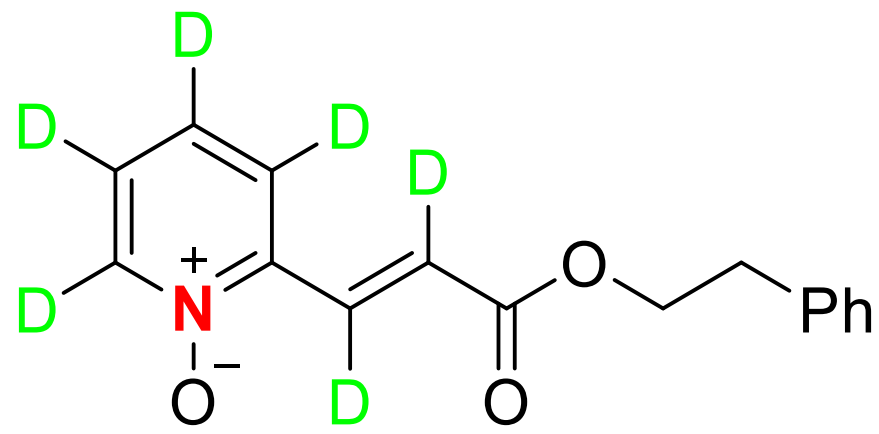

(E)-2-(3-Oxo-3-phenethoxyprop-1-en-1-yl-1,2-d$_2$)pyridine 1-oxide-3,4,5,6-d$_4$ (3)

A mixture of $Pd(OAc)_2$ (67 mg, 10 mol %), Ag_2CO_3 (2.47 g, 2.0 equiv) and pyridine N-oxide-d$_5$ (302 mg, 3.0 mmol, 1.0 equiv) was weighed into a 25 mL screw-capped vial equipped with a 10 x 5 mm spinvane triangular-shaped Teflon stirbar. 1,4-Dioxane (6.0 mL) and pyridine (240 mg, 3.0 mmol, 1.0 equiv) were added, followed by ethyl acrylate **S-3** (810.9 mg,4.5 mmol). The resulting mixture was sealed with a Teflon-lined cap and stirred at 105 °C for 48 h in an oil bath. The reaction mixture was cooled to room temperature, diluted with EtOAc (25 mL), and filtered through a celite pad. The filtrate was concentrated and purified by column chromatography, delivering the desired pyridine-^{15}N-oxide acrylate **3** as a pale yellow solid (231 mg, 28%). ^{1}**H NMR** (300 MHz, $CDCl_3$) δ = 7.34–7.18 (m, 5H), 4.44 (t, J = 7.0 Hz, 2H), 3.02 (t, J = 7.0 Hz, 2H). ^{2}**H NMR** (46 MHz, $CDCl_3$) δ = 8.21–8.03 (m), 7.60–6.91 (m). ^{13}**C NMR** (75 MHz, $CDCl_3$) δ = 165.85, 144.55 (d, $^1J_{C\text{-}N}$ = 15.1 Hz), 139.75 (d, $^1J_{C\text{-}N}$ = 15.5 Hz), 137.38, 133.44, 128.63, 128.25, 126.32, 125.36, 125.15, 124.44 (d, $^2J_{C\text{-}N}$ = 4.5 Hz), 123.88 (d, $^2J_{C\text{-}N}$ = 2.1 Hz), 65.14, 34.77. ^{15}**N NMR** (30 MHz, $CDCl_3$) δ = 291.42 (s).**HR-MS** (ESI) m/z calcd for $C_{16}H_{10}D_6{}^{15}N_1O_3$ $[M + H]^+$ 277.1472, found 277.1478; $C_{16}H_9D_6{}^{15}N_1O_3Na_1$ $[M + Na]^+$ 299.1291, found 299.1285.

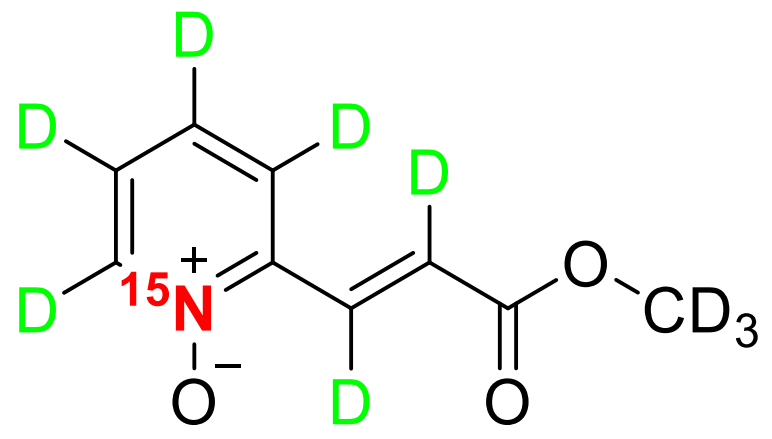

(E)-2-(3-(methoxy-d$_3$)-3-oxoprop-1-en-1-yl-1,2-d$_2$)pyridine 1-oxide-3,4,5,6-d$_4$-1-^{15}N (4)

To a solution of pyridine-^{15}N-oxide acrylate **3** (137.7 mg, 0.5 mmol, 1.0 equiv) in EtOH (2.0 mL) was added an aqueous solution of and NaOH (2.0 M, 2.0 mL) at ambient temperature. The mixture was stirred at 23 °C for 16 h. Then HCl (2.0 M, 2.05 mL) was added and the mixture was concentrated to afford the crude carboxylic acid, which was used for the next step without further purification. The crude carboxylic acid was suspended in CH_2Cl_2 (10 mL), and CD_3OD (90.2 mg, 2.5 mmol, 5.0 equiv) and DMAP (6.1 mg, 0.05 mmol, 0.1 equiv) were added. After the addition of a solution of EDCI (479.2 mg, 2.5 mmol, 5.0 equiv) in CH_2Cl_2 was added dropwise at 0 °C, the mixture was allowed to warm to ambient temperature gradually and stirred for 16 h. The reaction was quenched by adding H2O (10 mL), and the mixture was extracted with CH2Cl2 (15 mL × 3). The combined organic phase was dried over Na_2SO_4, concentrated, and purified by column chromatography (EtOAc/MeOH = 3:1), yielding the desired pyridine-15N-oxide acrylate 4 (64 mg, 67% over two steps) as a pale yellow solid. **^{13}C NMR** (75 MHz, $CDCl_3$) δ = 166.48, 144.69 (d, $^1J_{C\text{-}N}$ = 15.1 Hz), 139.88 (d, $^1J_{C\text{-}N}$ = 15.6 Hz), 133.52, 125.40, 125.28, 124.74 (d, $^2J_{C\text{-}N}$ = 4.5 Hz), 123.84 (d, $^2J_{C\text{-}N}$ = 2.2 Hz), 51.09. **^{2}H NMR** (46 MHz, $CDCl_3$) δ = 8.20 (s, 1D), 7.98 (s, 1D), 7.61–6.91 (m, 4D), 3.69 (s, 3D). **^{15}N NMR** (30 MHz, $CDCl_3$) δ = 290.97(s). **HR-MS** (ESI) *m/z* calcd for $C_9D_9H_1{}^{15}NO_3$ [M + H]$^+$ 190.1191, found 190.1190; $C_9D_9{}^{15}NO_3Na_1$ [M + Na]$^+$ 277.1472, found 277.1478.

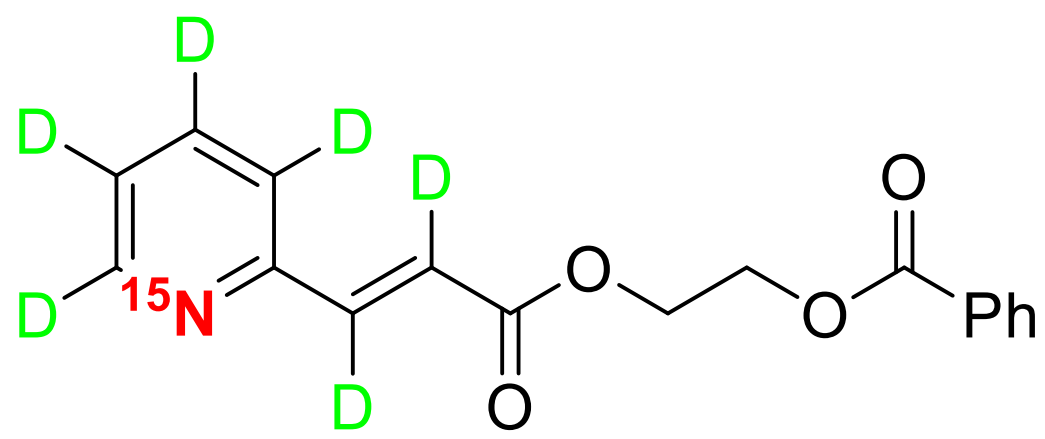

(E)-2-((3-(pyridin-2-yl-d4-1-15N)acryloyl-d2)oxy)ethyl benzoate (5)

To a stirred mixture of **1** (378 mg, 1.18 mmol) in toluene (5.0 mL) was added PCl3 (130 µL, 1.41 mmol, 1.2 equiv) dropwise at 0 °C. The reaction mixture was stirred for 15 min at room temperature. A saturated

solution of $NaHCO_3$ (5.0 mL) was added, and the mixture was stirred for an additional 5 min. The aqueous layer was then washed with CH_2Cl_2 (20 mL × 3). The combined organic layers were dried over MgSO4, filtered, and concentrated in vacuo. The crude was purified by flash column chromatography with EtOAc/hexane (1:4) to afford the corresponding deoxygenated product **5** as a pale oil (137 mg,38 %). **^{1}H NMR** (300 MHz, $CDCl_3$) δ = 7.93 (d, J = 6.9 Hz, 2H), 7.42–7.37 (m, 1H), 7.30–7.25 (m, 2H), 4.46 – 4.45 (m, 4H). **^{2}H NMR** (46 MHz, $CDCl_3$) δ = 8.52 (s, 1D), 7.61–6.91 (m, 5D). **^{15}N NMR** (30 MHz, $CDCl_3$) δ = 310.05. **^{13}C NMR** (75 MHz, $CDCl_3$) δ = 165.89, 165.77, 152.05 (d, $^3J_{C\text{-}N}$ = 1.6 Hz), 149.26, 143.19 (d, $^1J_{C\text{-}N}$ = 8.5 Hz), 135.80 (d, $^2J_{C\text{-}N}$ = 3.1 Hz), 132.67, 129.36, 129.23, 127.95, 123.45 (d, $^2J_{C\text{-}N}$ = 2.6 Hz), 123.36 (d, $^3J_{C\text{-}N}$ = 2.4 Hz), 120.79 (d, $^1J_{C\text{-}N}$ = 3.6 Hz), 62.32, 61.94. **HR-MS** (ESI) *m/z* calcd for $C_{17}H_9D_6{}^{15}NO^4Na_1$ $[M + Na]^+$ 327.1241, found 327.1240; $C_{17}H_{10}D_6{}^{15}NO^4Na_1$ $[M + H]^+$ 305.1421, found 305.1422.

Rmb57

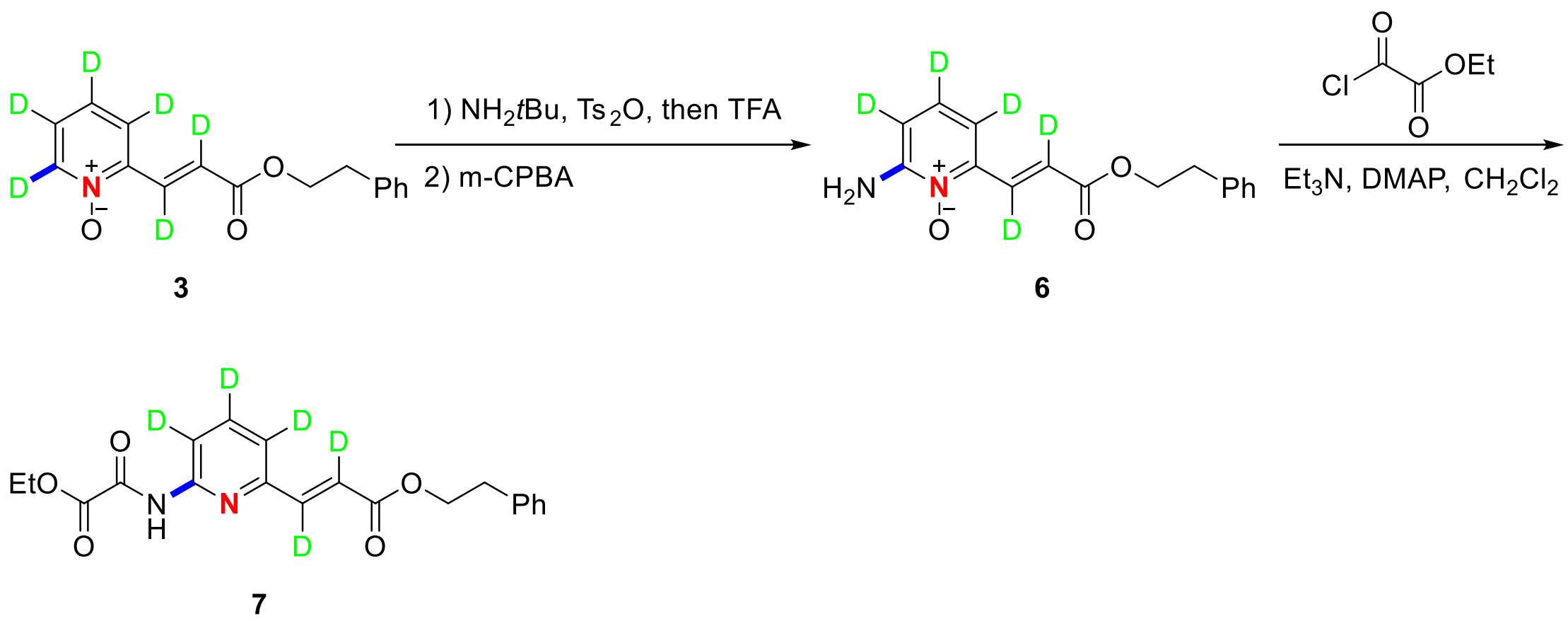

Scheme S-c. Synthesis of pyridine-^{15}N acrylate **6** and **7**.

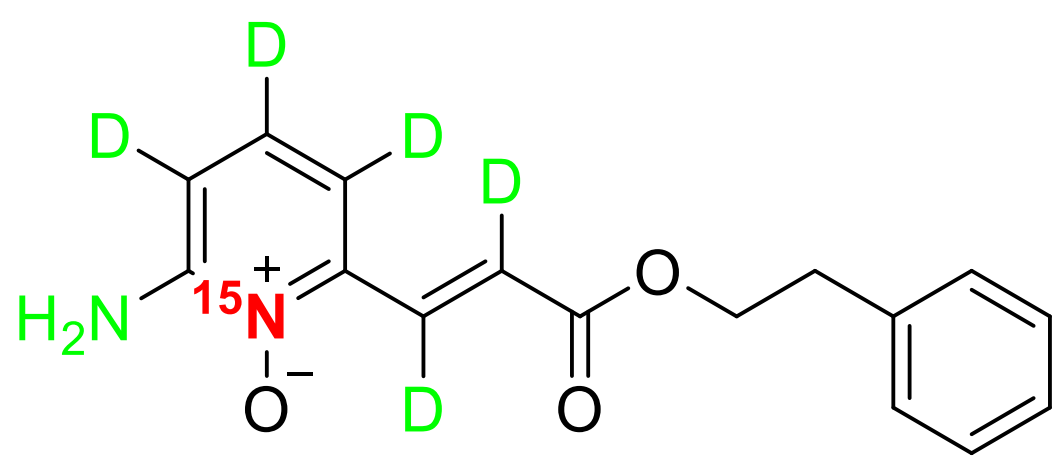

(E)-2-Amino-6-(3-oxo-3-phenethoxyprop-1-en-1-yl-1,2-d$_2$)pyridine 1-oxide-3,4,5-d$_3$-1-^{15}N (6)

Following a reported procedure,[4] To a solution of pyridine N-oxide **3** (464 mg, 1.68 mmol, 1.0 equiv) and tertbutylamine (2.14 mL, 20.2 mmol, 12 equiv) in $PhCF_3$ (10 mL) at 0 °C was added Ts_2O (2.75 g, 8.42 mmol, 5.0 equiv) as a solid in portions while maintaining the reaction temperature at <5 °C. After being stirred at 0 °C for 20 min, TFA (8.0 mL) was added to the reaction mixture, which was then aged at 80 °C for 5 h. The solution was concentrated to oil and diluted with water (5.0 mL) and CH_2Cl_2 (10 mL). The pH was carefully adjusted to ~10 with saturated aq $NaHCO_3$ (no NaOH, to avoid the Hydrolysis of the ester). The aqueous layer was extracted with CH_2Cl_2 (4 × 20 mL). The combined organic layers were concentrated and passed through a short silica pad to give a mixture of phenethyl (E)-3-(6-aminopyridin-2-yl-3,4,5-d$_3$-1-^{15}N)acrylate-d$_2$ and N-(tert-butyl)-4-methylbenzenesulfonamide in the ratio of 1:5.3 based on ^{1}H-NMR analysis. This mixture was directly oxidized with mCPBA without further purification.

To the solution of crude 2-amino pyridine from the last step in CH_2Cl_2 was added mCPBA at 0 °C in portions. The mixture was allowed to warm to 23 °C and stirred for 16 h. The mixture was concentrated in vacuo and purified with flash column chromatography (EtOAc/MeOH (4:1) to afford the corresponding 2-amino pyridine N-oxide **6** as a pale yellow solid (147 mg,30 % for 2 steps) .**^{1}H NMR** (300 MHz, $CDCl_3$) δ = 7.36–7.02 (m, 5H), 6.46 (s_{br}, 2H), 4.36 (t, J = 7.1 Hz, 2H), 2.94 (t, J = 7.1 Hz, 2H). **^{2}H NMR** (46 MHz, $CDCl_3$) δ = 8.11-6.91 (m, 5D). **^{13}C NMR** (75 MHz, $CDCl_3$) δ = 166.01, 150.83 (d, $^1J_{C\text{-}N}$ = 17.4 Hz), 142.64 (d, $^1J_{C\text{-}N}$ = 17.1 Hz), 137.38, 134.69, 128.60, 128.23, 126.61 (d, $^2J_{C\text{-}N}$ = 3.5 Hz), 126.29, 123.14 (d, $^2J_{C\text{-}N}$ = 2.0 Hz), 112.30, 109.50, 65.09, 34.74. **^{15}N NMR** (30 MHz, $CDCl_3$) δ = 249.39. **HR-MS** (ESI) *m/z* calcd for $C_{16}H_{12}D_5{}^{15}N_1O_3$ [M + H]$^+$ 291.1518, found 291.1515; $C_{16}H_{11}D_5{}^{15}N_1O_3Na_1$ [M + Na]$^+$ 313.1337, found 313.1340.

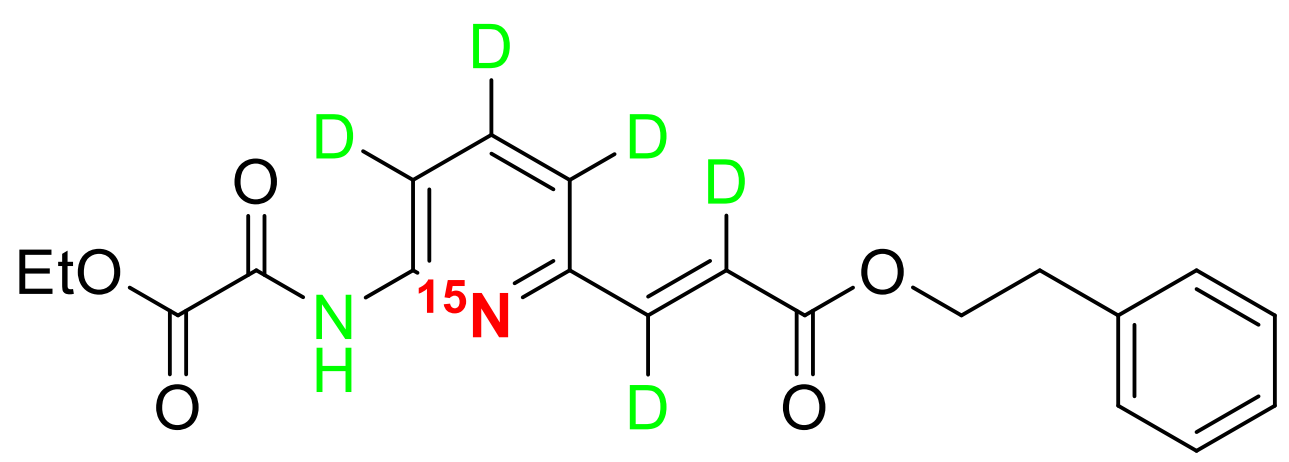

Phenethyl (E)-3-(6-(2-ethoxy-2-oxoacetamido)pyridin-2-yl-3,4,5-d$_3$-1-^{15}N)acrylate-d$_2$ (7)

To the solution of 2-amino pyridine N-oxide **6** (72 mg, 0.25 mmol) in CH_2Cl_2 (10 mL) were added Et_3N (101 mg, 1.0 mmol, 4.0 equiv) and DMAP (6.1 mg, 0.2 equiv). Ethyl 2-chloro-2-oxoacetate (102.4 mg, 0.75 mmol, 3.0 equiv) was added dropwise at 0 °C. The mixture was allowed to warm to 23 °C and stirred for 16 h. The reaction was stopped by adding H_2O (10 mL). After the organic phase was collected, the aqueous phase was extracted with CH_2Cl_2 (3 × 10 mL). The combined organic phase was dried over Na_2SO_4 and concentrated *in vacuo* and purified with flash column chromatography (EtOAc/nHexane (4:1) to afford the corresponding 2-amide pyridine **7** (35 mg, 37%) as a pale white solid. **^{1}H NMR** (300 MHz, $CDCl_3$) δ = 11.41 (s, 1H), 7.34–7.20 (m, 5H), 4.49-4.42 (m, 4H), 3.02 (t, J = 7.0 Hz, 2H), 1.44 (t, J = 7.1 Hz, 3H). **^{2}H NMR** (46 MHz, $CDCl_3$) δ = 8.46-6.91 (m, 5D). **^{13}C NMR** (75 MHz, $CDCl_3$) δ = 165.82, 158.64, 154.55, 154.53, 143.41 (d, $^1J_{C\text{-}N}$ = 17.7 Hz), 143.26 (d, $^1J_{C\text{-}N}$ = 17.2 Hz), 137.53, 133.35, 128.84, 128.48, 126.56, 126.06 (d, $^2J_{C\text{-}N}$ = 4.0 Hz), 125.25 (d, $^2J_{C\text{-}N}$ = 2.1 Hz), 119.71, 114.32, 65.51, 64.04, 35.00, 13.92. **^{15}N NMR** (30 MHz, $CDCl_3$) δ = 264.54. **HR-MS** (ESI) *m/z* calcd for $C_{20}H_{15}D_5N^{15}NO_5K$ $[M + K]^+$ 413.1288, found 413.1496.

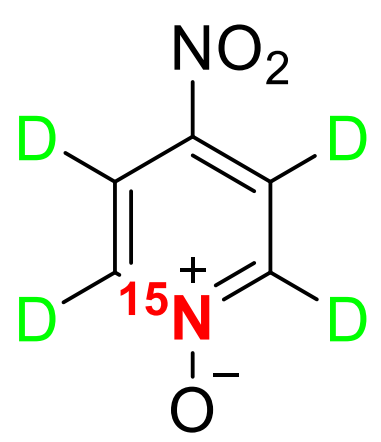

4-Nitropyridine 1-oxide-2,3,5,6-d$_4$-1-^{15}N (S-4)

At 0 °C, pyridine 1-oxide-d$_5$-1-^{15}N (1.7 g, 16.8 mmol) was carefully added into concentrated H_2SO_4 (99%, 4.0 mL) in a 25 mL round-bottom flask, which was equipped with a condenser. Then HNO_3 (65%, 2.0 mL) was added dropwise at 0 °C. The reaction mixture was heated up to 130 °C for 6.0 h. At ambient temperature, the acidic mixture was poured onto ice. The aqueous mixture was extracted with EtOAc (20 mL × 10). The combined organic phase was dried over Na_2SO_4, concentrated in vacuo and purified with

flash column chromatography (EtOAc/nHexane (1:1) to afford 4-Nitropyridine 1-oxide-2,3,5,6-d_4-1-^{15}N **S-4** (1.3 g, 53%) as a pale white solid. **^{2}H NMR** (46 MHz, DMSO-d_6) δ = 8.39 (s, 2D), 8.19 (s, 2D). **^{15}N NMR** (30 MHz, DMSO-d_6) δ = 308.18. **^{13}C NMR** (75 MHz, DMSO-d_6) δ = 143.37 (d, $^2J_{C\text{-}N}$ = 4.2 Hz), 141.33 (d, $^1J_{C\text{-}N}$ = 14.4 Hz), 122.40 (d, $^3J_{C\text{-}N}$ = 1.2 Hz). **HR-MS** (ESI) *m/z* calcd for $C_5H_1D_4N_1{}^{15}N_1O_3$ [M + H]$^+$ 146.0517, found 146.0515.

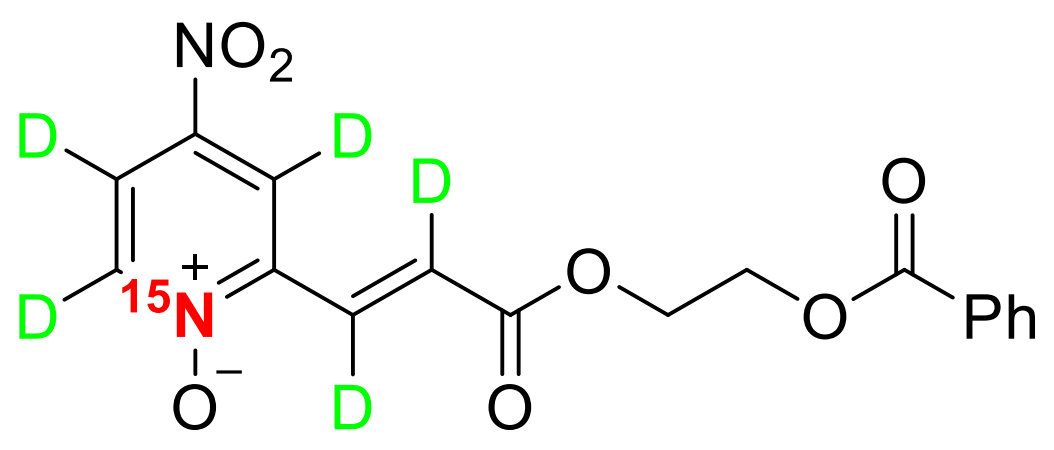

(E)-2-(3-(2-[Benzoyloxy]ethoxy)-3-oxoprop-1-en-1-yl-1,2-d2)-4-nitropyridine 1-oxide-3,5,6-d_3-1-^{15}N (8)

A mixture of $Pd(OAc)_2$ (89 mg, 20 mol %), Ag_2CO_3 (1.1 g, 2.0 equiv) and 4-nitropyridine 1-oxide-2,3,5,6-d_4-1-^{15}N **S-4** (290 mg, 2.0 mmol, 1.0 equiv) was weighed into a 25 mL screw-capped vial equipped with a 10 x 5 mm spinvane triangular-shaped Teflon stirbar. 1,4-Dioxane (5.0 mL) and pyridine (160 mg, 2.0 mmol, 1.0 equiv) were added, followed by ethyl acrylate **S-1** (669 mg, 6.0 mmol). The resulting mixture was sealed with a Teflon-lined cap and stirred at 105 °C for 48 h in an oil bath. The reaction mixture was cooled to room temperature, diluted with EtOAc (25 mL), and filtered through a celite pad. The filtrate was concentrated and purified by column chromatography, delivering the desired pyridine-^{15}N-oxide acrylate **8** as a pale yellow solid (179 mg, 24%). **^{1}H NMR** (300 MHz, $CDCl_3$) δ = 8.04 (d, J = 7.6 Hz, 2H), 7.56 (t, J = 7.3 Hz, 1H), 7.44 (t, J = 7.7 Hz, 2H), 4.60 (s, 4H). **^{2}H NMR** (46 MHz, $CDCl_3$) δ = 8.33-6.91 (m, 5D). **^{13}C NMR** (75 MHz, $CDCl_3$) δ = 166.25, 165.34, 145.64 (d, $^1J_{C\text{-}N}$ = 14.7 Hz), 141.44 (d, $^2J_{C\text{-}N}$ = 3.1 Hz), 141.00 (d, $^1J_{C\text{-}N}$ = 15.3 Hz), 133.17, 132.41, 129.65, 129.61, 128.38, 125.75 (d, $^2J_{C\text{-}N}$ = 2.1 Hz), 120.25, 119.15, 63.00, 62.48. **^{15}N NMR** (30 MHz, $CDCl_3$) δ = 304.41.**HR-MS** (ESI) *m/z* calcd for $C_{17}H_{10}D_5N_1{}^{15}N_1O_7$ [M+H]$^+$ 365.1158, found 365.1147; $C_{17}H_9D_5N_1{}^{15}N_1O_7Na_1$ [M + Na]$^+$ 387.0977, found 387.0974.

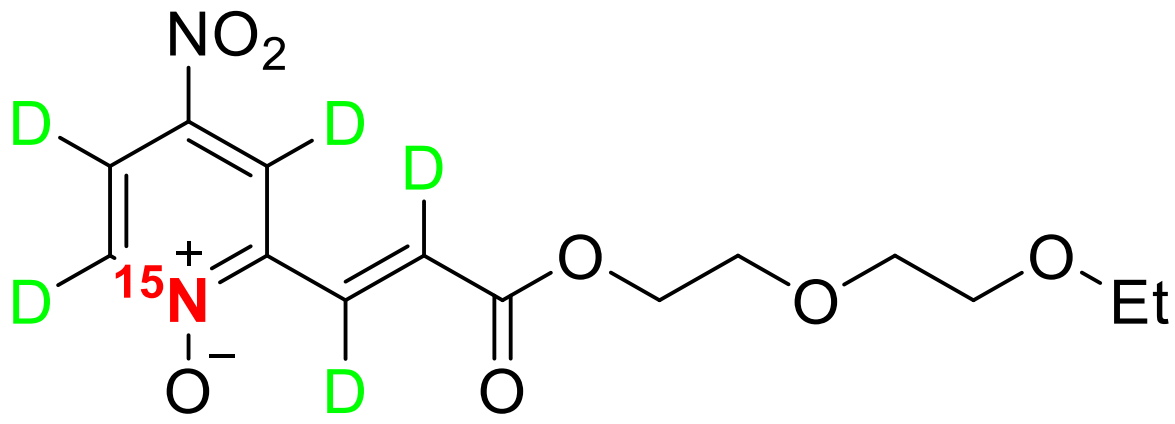

(E)-2-[3-(2-(2-ethoxyethoxy)ethoxy)-3-oxoprop-1-en-1-yl-1,2-d$_2$]-4-nitropyridine 1-oxide-3,5,6-d$_3$-1-^{15}N (9)

A mixture of $Pd(OAc)_2$ (89 mg, 20 mol %), Ag_2CO_3 (1.1 g, 2.0 equiv) and 4-nitropyridine 1-oxide-2,3,5,6-d$_4$-1-^{15}N **S-4** (290 mg, 2.0 mmol, 1.0 equiv) was weighed into a 25 mL screw-capped vial equipped with a 10 x 5 mm spinvane triangular-shaped Teflon stirbar. 1,4-Dioxane (5.0 mL) and pyridine (160 mg, 2.0 mmol, 1.0 equiv) were added followed by ethyl acrylate **S-2** (765 mg,4.0 mmol). The resulting mixture was sealed with a Teflon-lined cap and stirred at 105 °C for 48 h in an oil bath. The reaction mixture was cooled to room temperature, diluted with EtOAc (25 mL), and filtered through a Celite pad. The filtrate was concentrated and purified by column chromatography, delivering the desired pyridine-^{15}N-oxide acrylate **9** as a pale yellow solid (112 mg, 17%).**^{1}H NMR** (300 MHz, $CDCl_3$) δ = 4.36 (t, J = 4.7 Hz, 2H), 3.75 (t, J = 4.8 Hz, 2H), 3.64 (dd, J = 6.1, 3.5 Hz, 2H), 3.56 (dd, J = 6.1, 3.5 Hz, 2H), 3.49 (q, J = 7.0 Hz, 2H), 1.16 (t, J = 7.0 Hz, 3H). **^{2}H NMR** (46 MHz, $CDCl_3$) δ = 8.36–6.91 (m, 5D). **^{13}C NMR** (75 MHz, $CDCl_3$) δ = 165.40, 145.64 (d, $^1J_{C\text{-}N}$ = 14.7 Hz), 141.44 (d, $^2J_{C\text{-}N}$ = 4.0 Hz), 140.96 (d, $^1J_{C\text{-}N}$ = 15.3 Hz), 131.94, 126.00 (d, $^2J_{C\text{-}N}$ = 2.1 Hz), 120.06, 119.07, 70.53, 69.62, 68.81, 66.50, 64.26, 14.97. **^{15}N NMR** (30 MHz, $CDCl_3$) δ = 304.83. **HR-MS** (ESI) *m/z* calcd for $C_{14}H_{13}D_5N_1{}^{15}N_1O_7Na_1$ $[M + Na]^+$ 355.1291, found 355.1288.

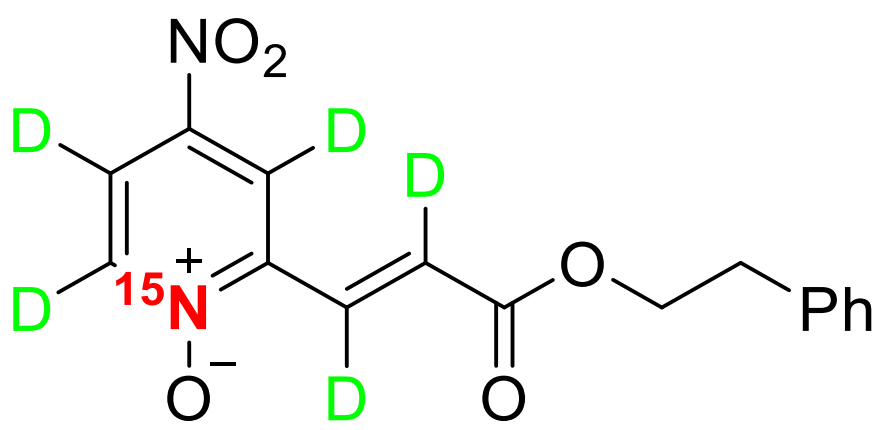

(E)-4-Nitro-2-(3-oxo-3-phenethoxyprop-1-en-1-yl-1,2-d2)pyridine 1-oxide-3,5,6-d3-1-^{15}N (10)

A mixture of $Pd(OAc)_2$ (89 mg, 10 mol %), Ag_2CO_3 (2.2 g, 2.0 equiv) and 4-nitropyridine 1-oxide-2,3,5,6-d$_4$-1-^{15}N **S-4** (580 mg, 4.0 mmol, 1.0 equiv) was weighed into a 100 mL screw-capped vial equipped with a 10 x 5 mm spinvane triangular-shaped Teflon stirbar. 1,4-Dioxane (20 mL) and pyridine

(320 mg, 4.0 mmol, 1.0 equiv) were added, followed by ethyl acrylate S-3 (2.7 g,12 mmol). The resulting mixture was sealed with a Teflon-lined cap and stirred at 105 °C for 48 h in an oil bath. The reaction mixture was cooled to room temperature, diluted with EtOAc (100 mL), and filtered through a celite pad. The filtrate was concentrated and purified by column chromatography, delivering the desired pyridine-^{15}N-oxide acrylate **10** as a pale yellow solid (167 mg, 13%). **^{1}H NMR** (300 MHz, $CDCl_3$) δ = 7.35–7.21 (m, 5H), 4.46 (t, J = 7.0 Hz, 2H), 3.03 (t, J = 7.0 Hz, 2H). **^{2}H NMR** (46 MHz, $CDCl_3$) δ = 8.37–6.91 (m, 5D). **^{13}C NMR** (75 MHz, $CDCl_3$) δ = 165.40, 145.78 (d, $^{1}J_{C\text{-}N}$ = 14.7 Hz), 141.44 (d, $^{2}J_{C\text{-}N}$ = 3.2 Hz) 140.94 (d, $^{1}J_{C\text{-}N}$ = 15.3 Hz), 137.43, 131.85, 128.83, 128.51, 126.62, 126.24 (d, $^{2}J_{C\text{-}N}$ = 2.1 Hz), 120.05, 119.06, 65.70, 34.95. **^{15}N NMR** (30 MHz, $CDCl_3$) δ = 304.35. **HR-MS** (ESI) *m/z* calcd for $C_{16}H_9D_5N_1{}^{15}N_1O_5Na_1$ [M + Na]$^+$ 343.1080, found 343.1077.

2 Parahydrogen-induced polarization (PHIP) experiments

2.1 NMR Instrumentation used

All experiments were performed on a Bruker Avance III HD NMR spectrometer with a 7 T ultrashield magnet (^{1}H: 300.13 MHz, ^{13}C: 75.47 MHz, ^{2}H: 46.07 MHz, ^{15}N: 30.41 MHz). The system was equipped with a room-temperature 5 mm broadband direct-observe double-resonance probe (BBFO-H-D). 90° Hard pulses used with this probe were 9.5µs for ^{1}H, 8 µs for ^{13}C, 17 µs for ^{15}N and 200 µs for ^{2}H. A BCU I was using for temperature regulation. For parahydrogen experiments, magnetic valves controlling pressurization and sample bubbling are driven by the spectrometers' TTL output signals. The parahydrogen fraction of dihydrogen gas was enriched to > 98% by cooling down to 20 K with a He-cooled parahydrogen generator provided by a cryocooler system (Sumimoto HC-4A helium compressor, Sumimoto Cold Head CH-204 with parahydrogen reaction chamber by ColdEdge Technologies, temperature controller Lake Shore Cryotronics, Inc.) and delivered by a home-built valve and tubing system.

2.2 Sample & nuclear spin order preparation

Hyperpolarization experiments with pH_2 were performed using the 7 T system, as described above. Experiments with the homogeneous catalyst [Rh(dppb)(COD)][BF_4] were performed in methanol-d_4 or acetone-d_6 with the indicated concentration at 335 K unless otherwise noted. Pressurized N_2 at 7.0 bar

was bubbled through the samples for 60 seconds to remove oxygen, then the N2 pressure was released and the samples were inserted into the magnet. After temperature equilibration, 7.0 bar $p\mathrm{H}_2$ pressure were applied and the samples were supplied with $p\mathrm{H}_2$ for indicated time followed by a 1 s delay for sample homogenization.

2.3 General remark for ^{15}N hyperpolarization

For ^{15}N hyperpolarization experiments, the MINERVA[5] sequence in Fig. 1 (2) of ref. [5b] was used without the final 90° pulse (and using ^{15}N instead of ^{13}C as the X-nucleus). For pyridine-^{15}N-oxide acrylates, the delays Δ_1 = 66.6 ms and Δ_2 = 35.2 ms were used for the heteronuclear and homonuclear coupling evolution periods, respectively. ^{15}N polarizations were computed according to protocols described in our previous papers.[6] ^{15}N Reference spectra were collected with a 6.0 M ^{15}N-amonium chloride ($^{15}NH_4Cl$) sample prepared in D_2O. Full relaxation (relaxation delay > 5 T_1) was ensured.

2.4 The ^{15}N-NMR thermal spectrum of $^{15}NH_4Cl$, used as a reference for hyperpolarization efficiency calculation.

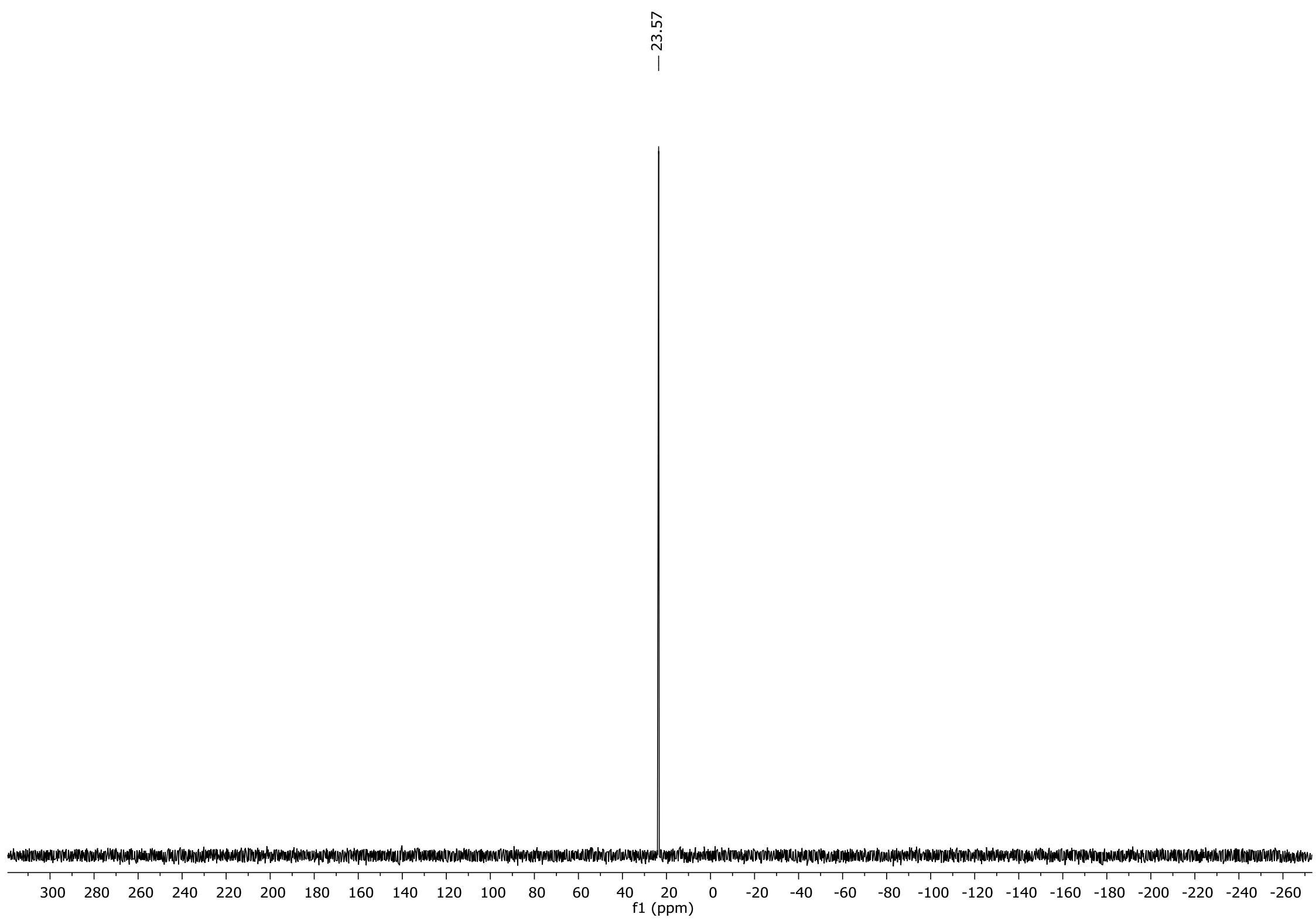

Object	Integral [abs]	Integral [rel]	Range (F1) from	Range (F1) to	ν(F1) [ppm]

Integral 1	77037.58	1.0	24.03	23.08	23.55

Figure S1. ^{15}N-NMR and corresponding integration of ^{15}N-Amoniachloride (6.00 M in D_2O) with a scanning number of 1 at 335K. (1st measurement).

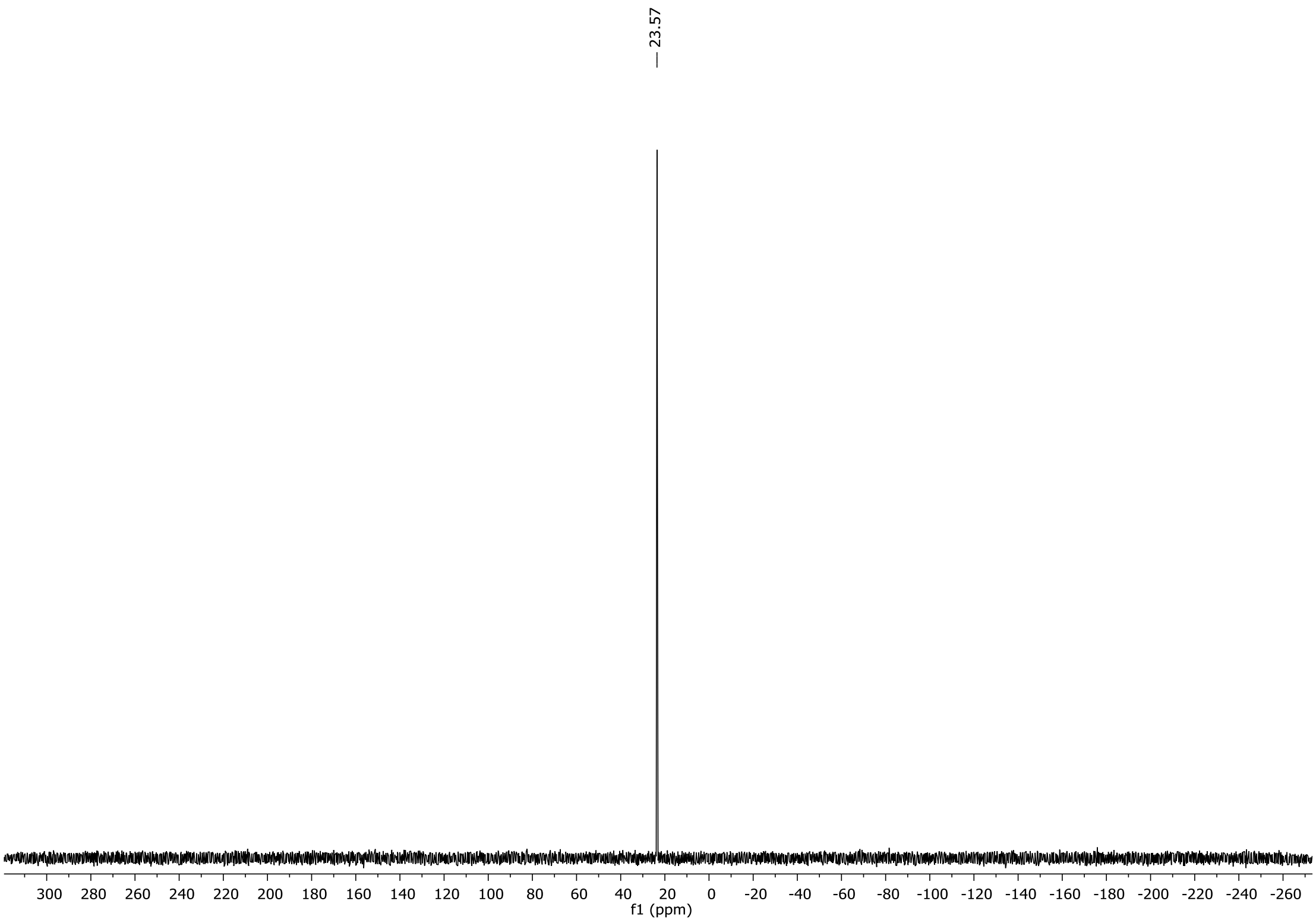

Figure S2. ^{15}N-NMR and corresponding integration of ^{15}N-Amoniachloride (6.00 M in D_2O) with a scanning number of 1 at 335K. (2nd measurement)

Object	Integral [abs]	Integral [rel]	Range (F1) from	Range (F1) to	ν(F1) [ppm]
Integral 1	76633.32	1.0	24.27	22.99	23.63

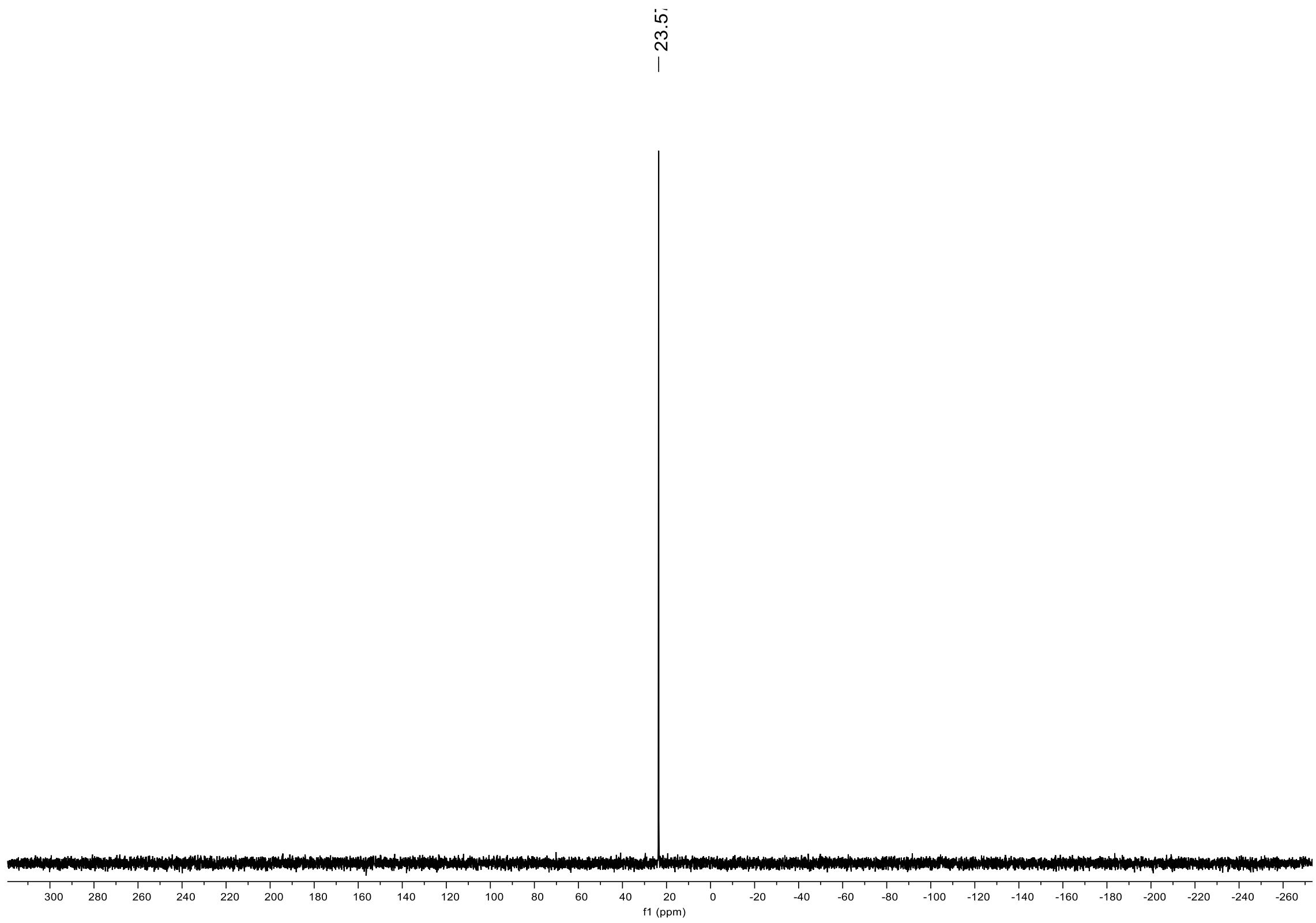

Figure S3. ^{15}N-NMR and corresponding integration of ^{15}N-Amoniachloride (6.00 M in D_2O) with a scanning number of 1 at 335K. (3rd measurement).

Object	Integral [abs]	Integral [rel]	Range (F1) from	Range (F1) to	ν(F1) [ppm]
Integral 1	73009.09	1.0	24.21	23.06	23.64

Object	Integral [abs]1	Integral [abs]2	Integral [abs]3	Average
Integral	77037.58	76633.32	73009.09	**75560±2219**

2.5 ^{15}N-PHIP parameter optimization

Table S1: Hydrogenative parahydrogen-induced hyperpolarization parameter optimization for 2-Alkenylpyridine 1-Oxide **1**.

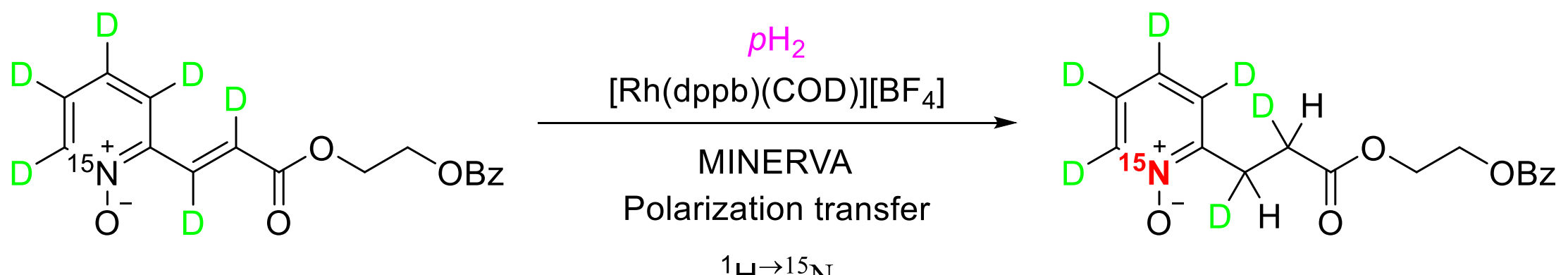

1
(291.9 ppm)

1'
(281.8 ppm)

entry	Deviation from standard conditions	^{15}N-HP efficiency (%)	entry	Deviation from standard conditions	^{15}N-HP efficiency (%)
1	J_{NH} = 4.0 Hz, [Rh]: 1.0 equiv.	1.6	9	J_{NH} = 4.0 Hz, [Rh]: 0.5 mol%	8.1
2	J_{NH} = 4.0 Hz, [Rh]: 50 mol%	8.4	10[a]	**none**	**47.9**
3	J_{NH} = 4.0 Hz, [Rh]: 20 mol%	10.8	11	J_{NH} = 4.0 Hz, T = 320 K	17.8
4	J_{NH} = 4.0 Hz, [Rh]: 10 mol%	25.7	12	J_{NH} = 4.0 Hz, T = 335 K	42.1
5	J_{NH} = 4.0 Hz, [Rh]: 5.0 mol%	32.9	13	J_{NH} = 4.0 Hz, pH_2 bubbling time: 11s	43.3
6	J_{NH} = 4.0 Hz, [Rh]: 3.3 mol%	46.0	14	J_{NH} = 4.0 Hz, pH_2 bubbling time: 7s	45.5
7	J_{NH} = 4.0 Hz, [Rh]: 2.0 mol%	33.4	15	J_{NH} = 3.5 Hz	44
8	J_{NH} = 4.0 Hz, [Rh]: 1.0 mol%	17.6	16	J_{HH} = 6.85 Hz	41.7

[a] Standard hyperpolarization conditions: **1** (2.5 mM) and [Rh(dppb)(COD)][BF4] (0.083 mM, 3.3 mol %) in MeOD-d4 (0.2 mL). pH_2 bubbling time = 9 s, 330 K, J_{HH}= 7.1 Hz, J_{NH}=3.75 Hz.

2.6 ^{15}N Hyperpolarization Spectra

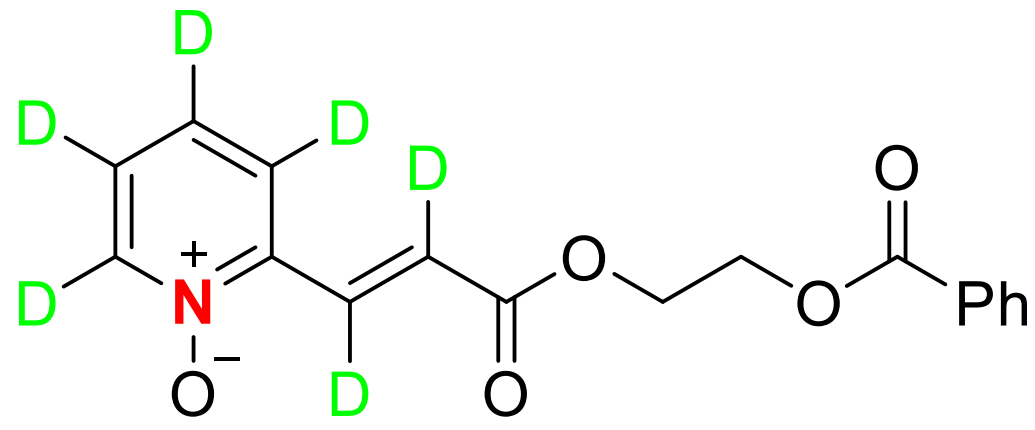

1

Hyperpolarization conditions: **1** / [Rh(dppb)(COD)][BF_4] 30:1, MeOD-d_4 (0.2 mL). pH_2 bubbling time = 9 s, 330 K, J_{HH}= 7.1 Hz, J_{NH}=3.75 Hz.

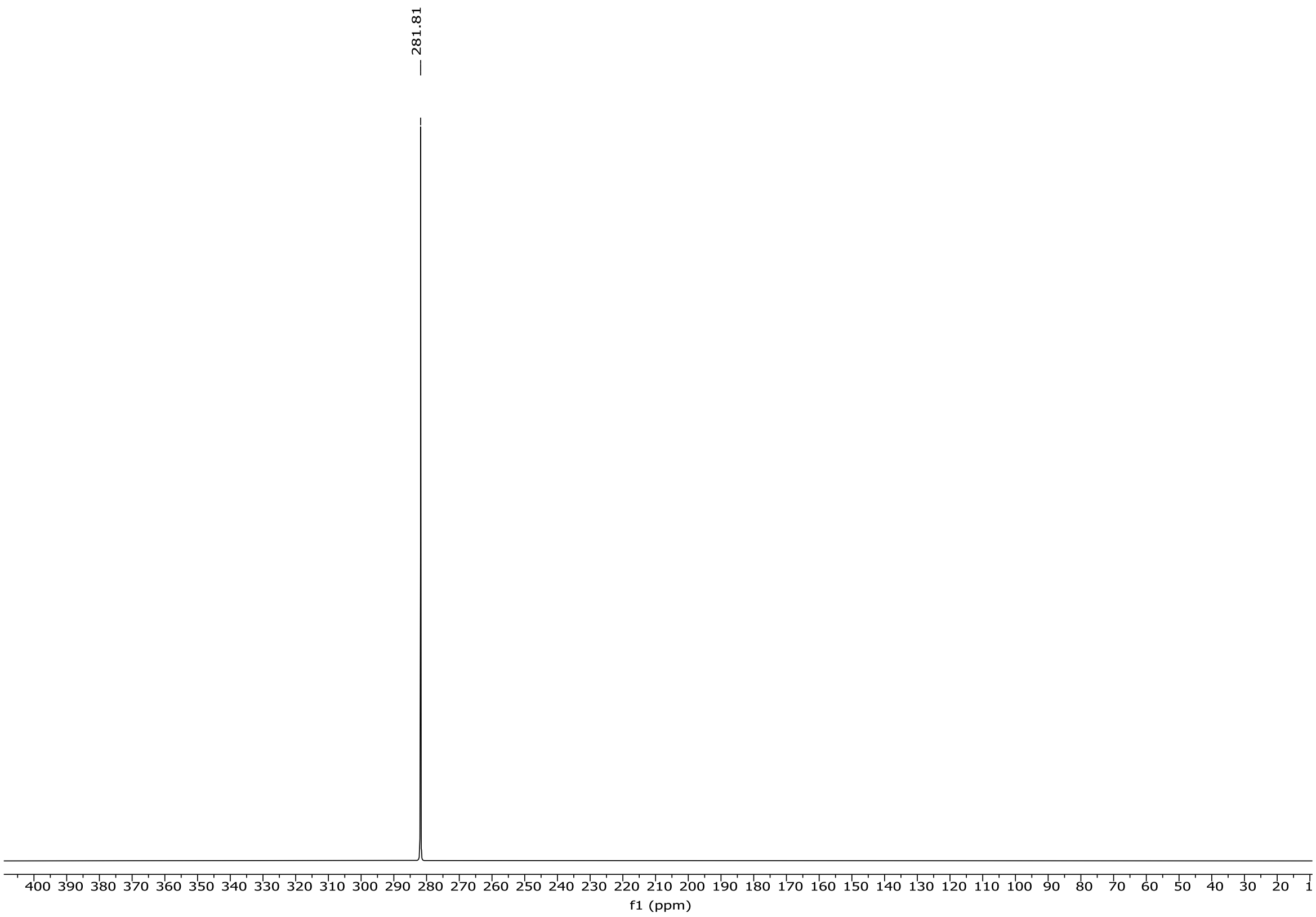

Object	Integral [abs]	Integral [rel]	Range (F1) from	Range (F1) to	ν(F1) [ppm]
Integral 1	5066310.69	1	281.92	281.21	281.81

Figure S4. ^{15}N-hyperpolarization of pyridine-N-oxide **1** (1.87 mM) and the corresponding integrations. A hyperpolarization efficiency of 47.5 % was achieved with this sample. (1st measurement).

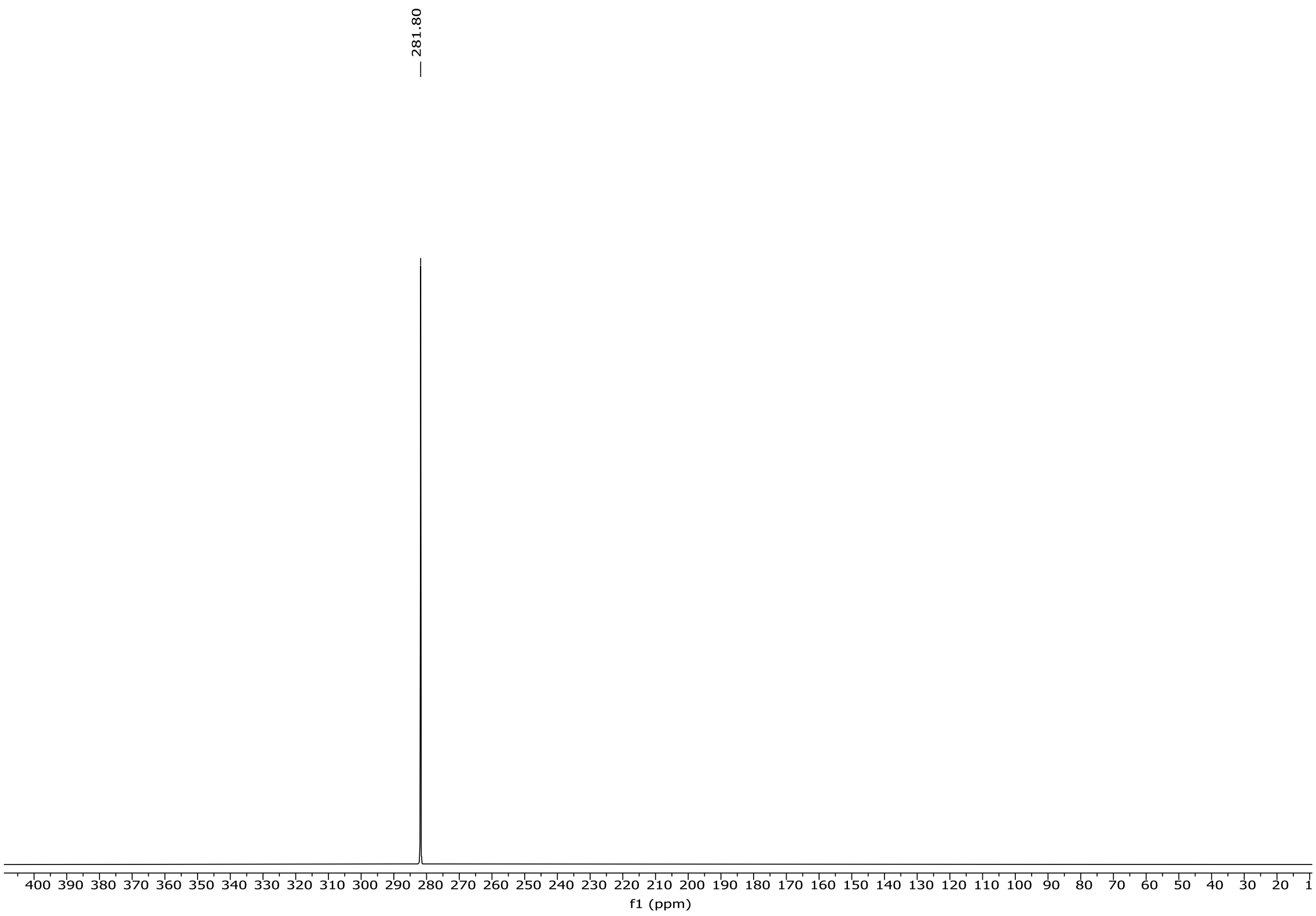

Object	Integral [abs]	Integral [rel]	Range (F1) from	Range (F1) to	ν(F1) [ppm]
Integral 1	5115951.00	1	281.92	281.21	281.80

Figure S5. ^{15}N-hyperpolarization of pyridine-N-oxide **1** (1.87 mM) and the corresponding integrations. A hyperpolarization efficiency of 47.9 % was achieved with this sample. (2nd measurement).

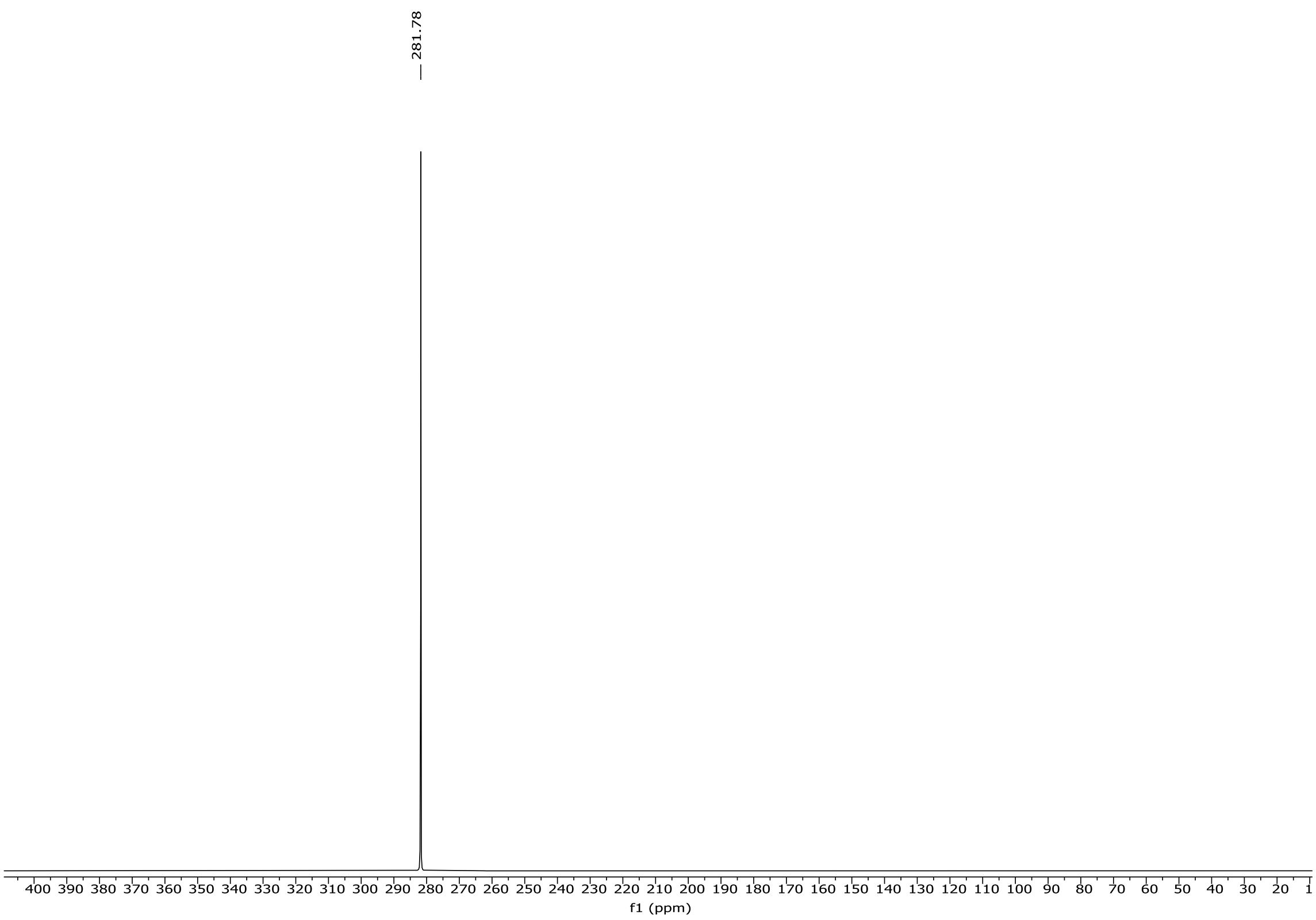

Object	Integral [abs]	Integral [rel]	Range (F1) from	Range (F1) to	ν(F1) [ppm]
Integral 1	4853460.04	1	281.99	281.12	281.78

Figure S6. ^{15}N-hyperpolarization of pyridine-N-oxide **1** (1.87 mM) and the corresponding integrations. A hyperpolarization efficiency of 45.6% was achieved with this sample. (3rd measurement).

Object	Effi (%)1	Effi (%)2	Effi (%)3	AVR Effi (%)1
Integral 1	47.5	47.9	45.6	**47±2**

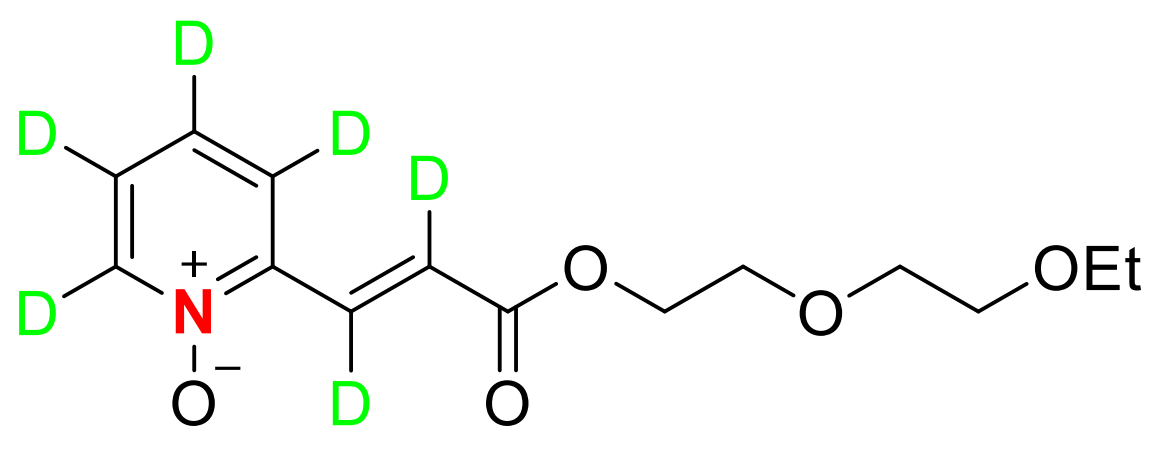

2

Hyperpolarization conditions: **2** / [Rh(dppb)(COD)][BF_4] 20:1, MeOD-d_4 (0.2 mL). pH_2 bubbling time = 9 s, 330 K, J_{HH}= 7.1 Hz, J_{NH}=3.75 Hz.

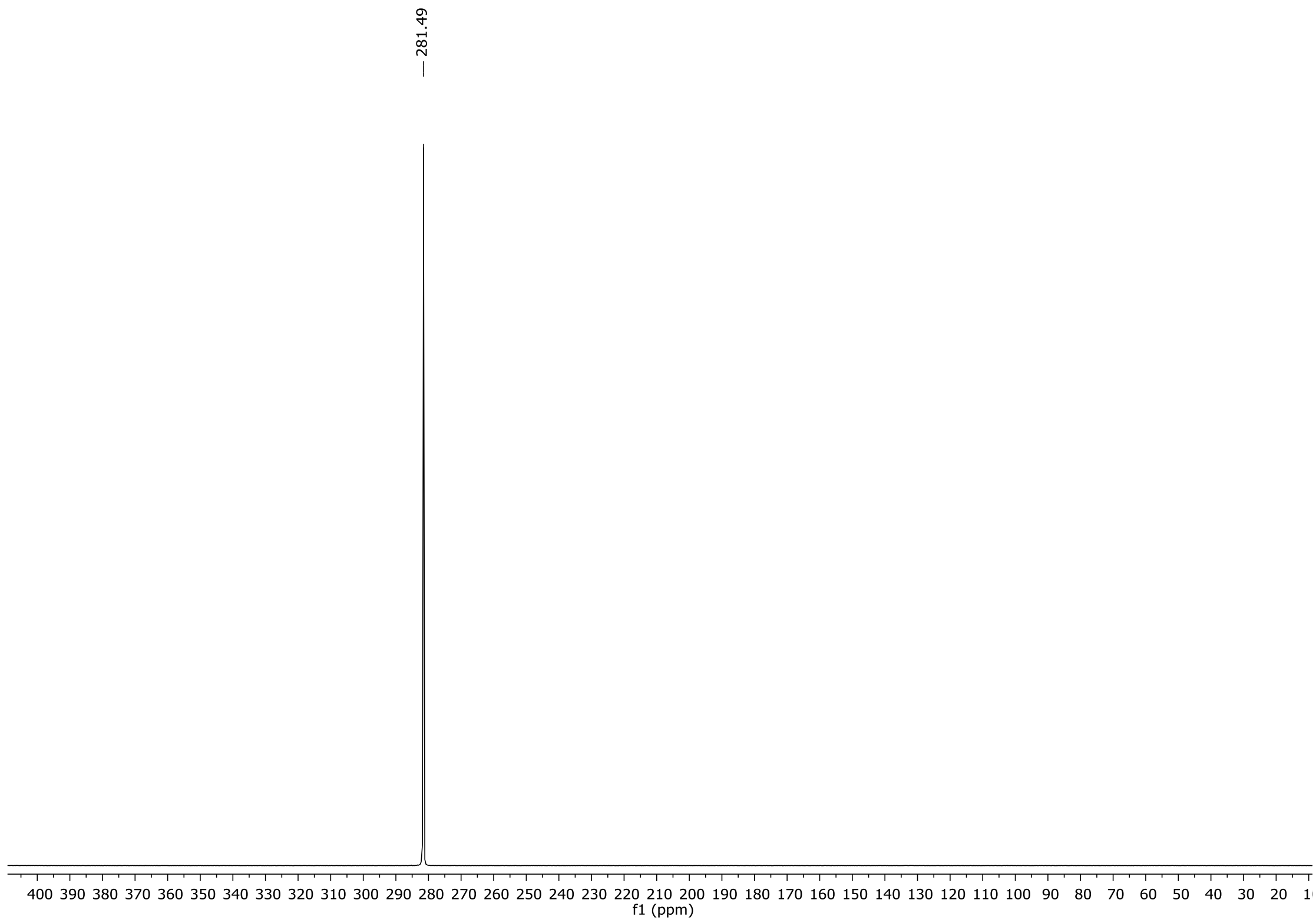

Object	Integral [abs]	Integral [rel]	Range (F1) from	Range (F1) to	ν(F1) [ppm]
Integral 1	2764141.93	1	282.36	280.94	281.65

Figure S7. ^{15}N-hyperpolarization of pyridine-N-oxide **2** (2.5 mM) and the corresponding integrations. A hyperpolarization efficiency of 19.4 % was achieved with this sample. (1st measurement).

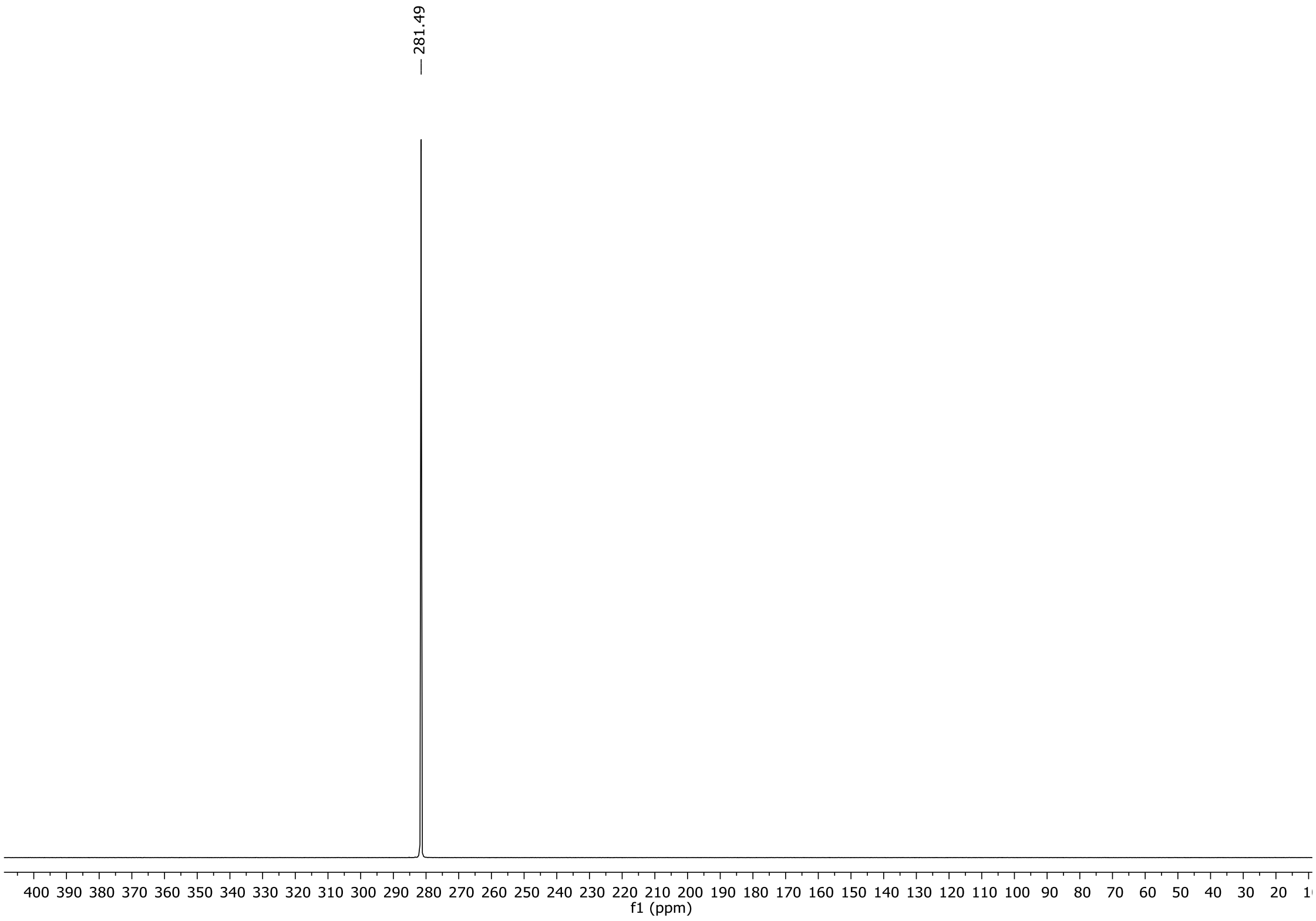

Object	Integral [abs]	Integral [rel]	Range (F1) from	Range (F1) to	ν(F1) [ppm]
Integral 1	2600332.264	1	282.3181536	281.0409216	281.6795376

Figure S8. ^{15}N-hyperpolarization of pyridine-N-oxide **2** (2.5 mM) and the corresponding integrations. A hyperpolarization efficiency of 18.3 % was achieved with this sample. (2nd measurement).

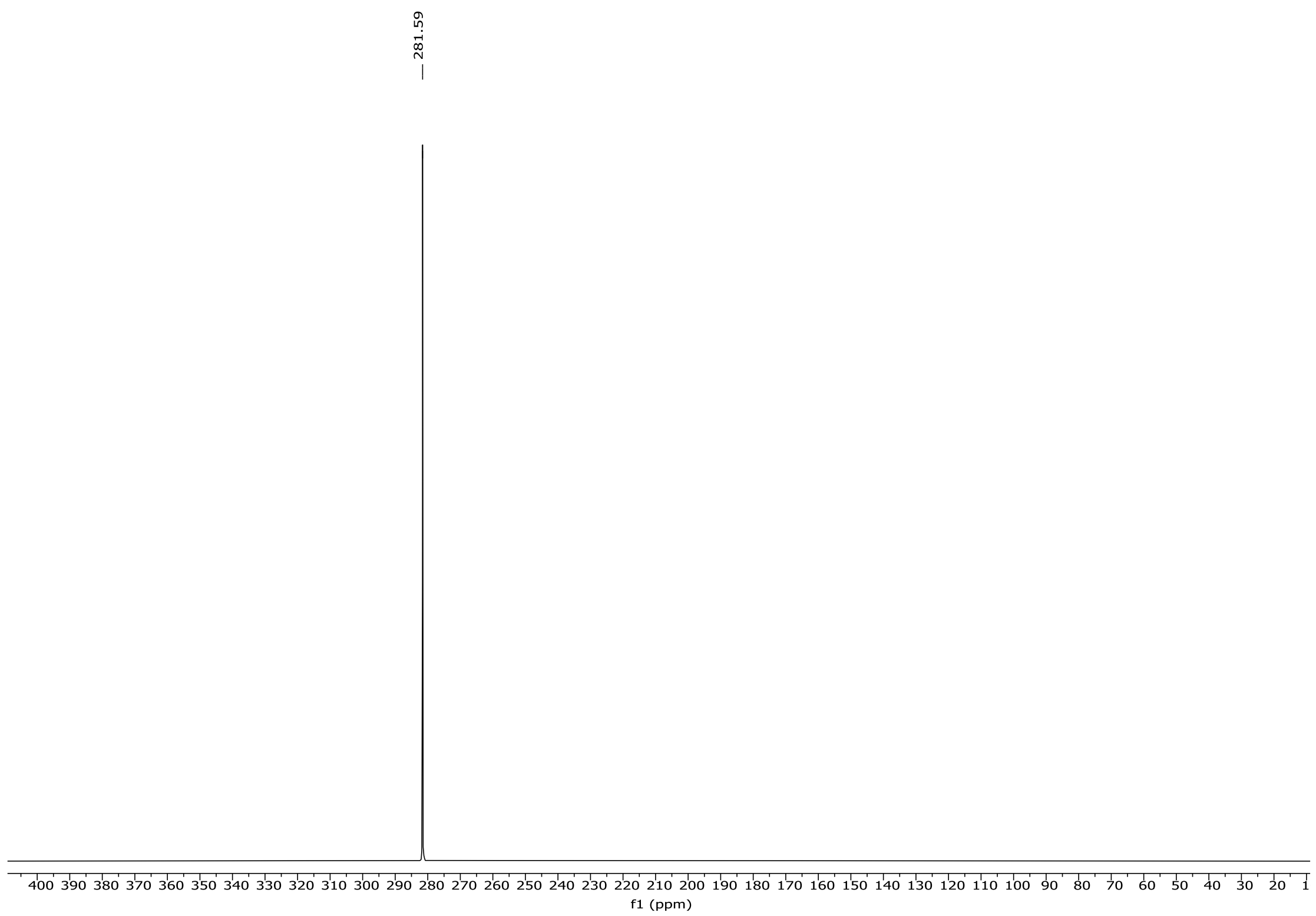

Object	Integral [abs]	Integral [rel]	Range (F1) from	Range (F1) to	(F1) [ppm]
Integral 1	3760269	1	282.069	281.209	281.6391

Figure S9. ^{15}N-hyperpolarization of pyridine-N-oxide **2** (4.0 mM) and the corresponding integrations. A hyperpolarization efficiency of 16.5 % was achieved with this sample. (3rd measurement).

Object	Effi (%)1	Effi (%)2	Effi (%)3	AVR Effi (%)1
Integral 1	19.4	18.3	16.5	**18±2**

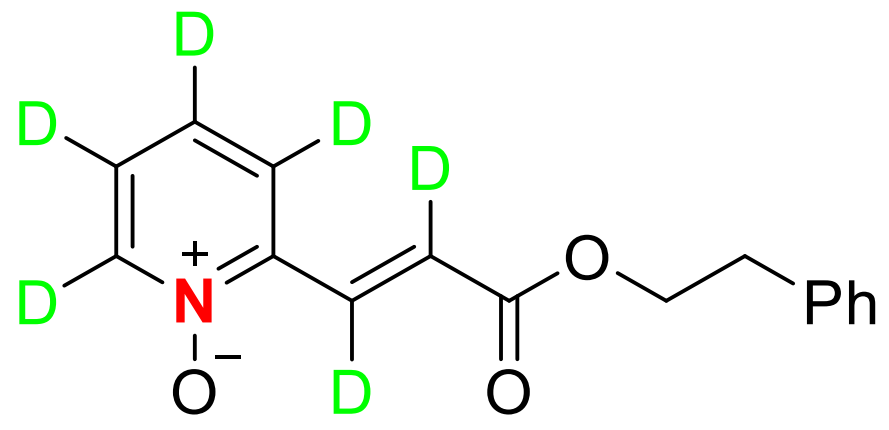

3

Hyperpolarization conditions: **3** / [Rh(dppb)(COD)][BF_4] 20:1, MeOD-d_4 (0.2 mL). pH_2 bubbling time = 9 s, 330 K, J_{HH}= 7.1 Hz, J_{NH}=3.75 Hz.

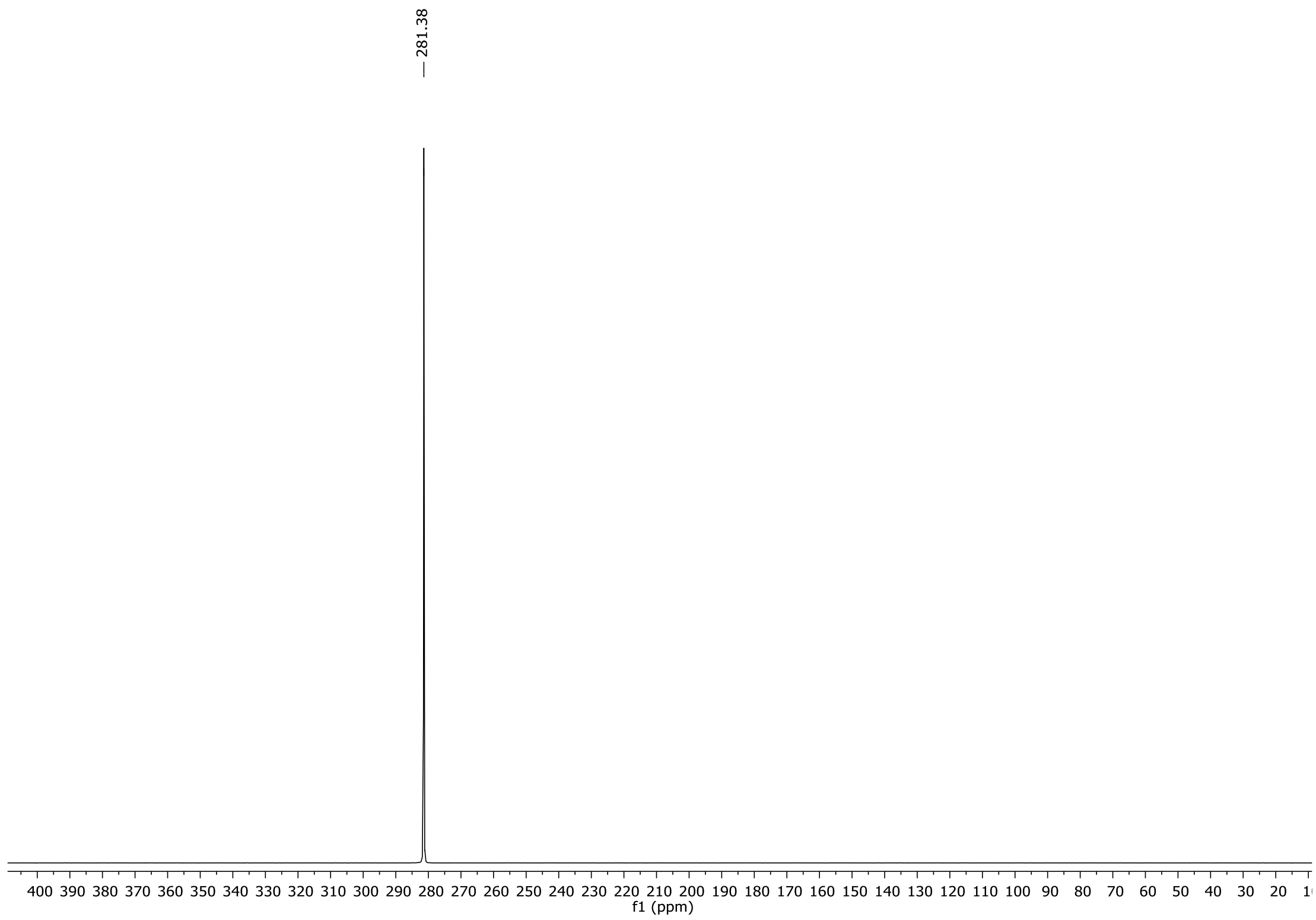

Object	Integral [abs]	Integral [rel]	Range (F1) from	Range (F1) to	ν(F1) [ppm]
Integral 1	4145153.52	1.0	282.99	279.76	281.38

Figure S10. ^{15}N-hyperpolarization of pyridine-N-oxide **2** (2.5 mM) and the corresponding integrations. A hyperpolarization efficiency of 29.1 % was achieved with this sample. (1st measurement).

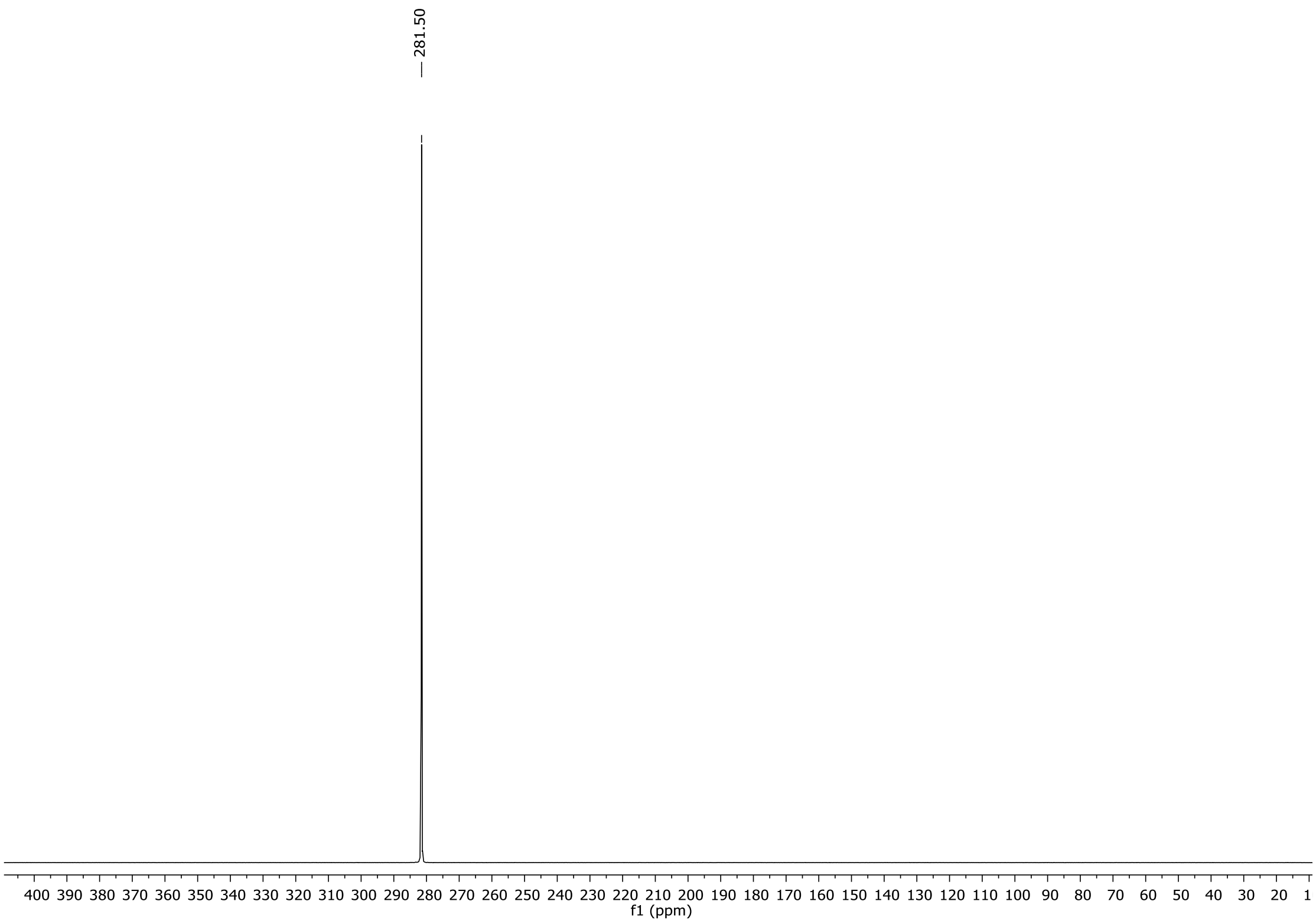

Object	Integral [abs]	Integral [rel]	Range (F1) from	Range (F1) to	ν(F1) [ppm]
Integral 1	3790501.86	1	281.99	281.02	281.50

Figure S11. ^{15}N-hyperpolarization of pyridine-N-oxide **2** (2.5 mM) and the corresponding integrations. A hyperpolarization efficiency of 26.6 % was achieved with this sample. (2nd measurement).

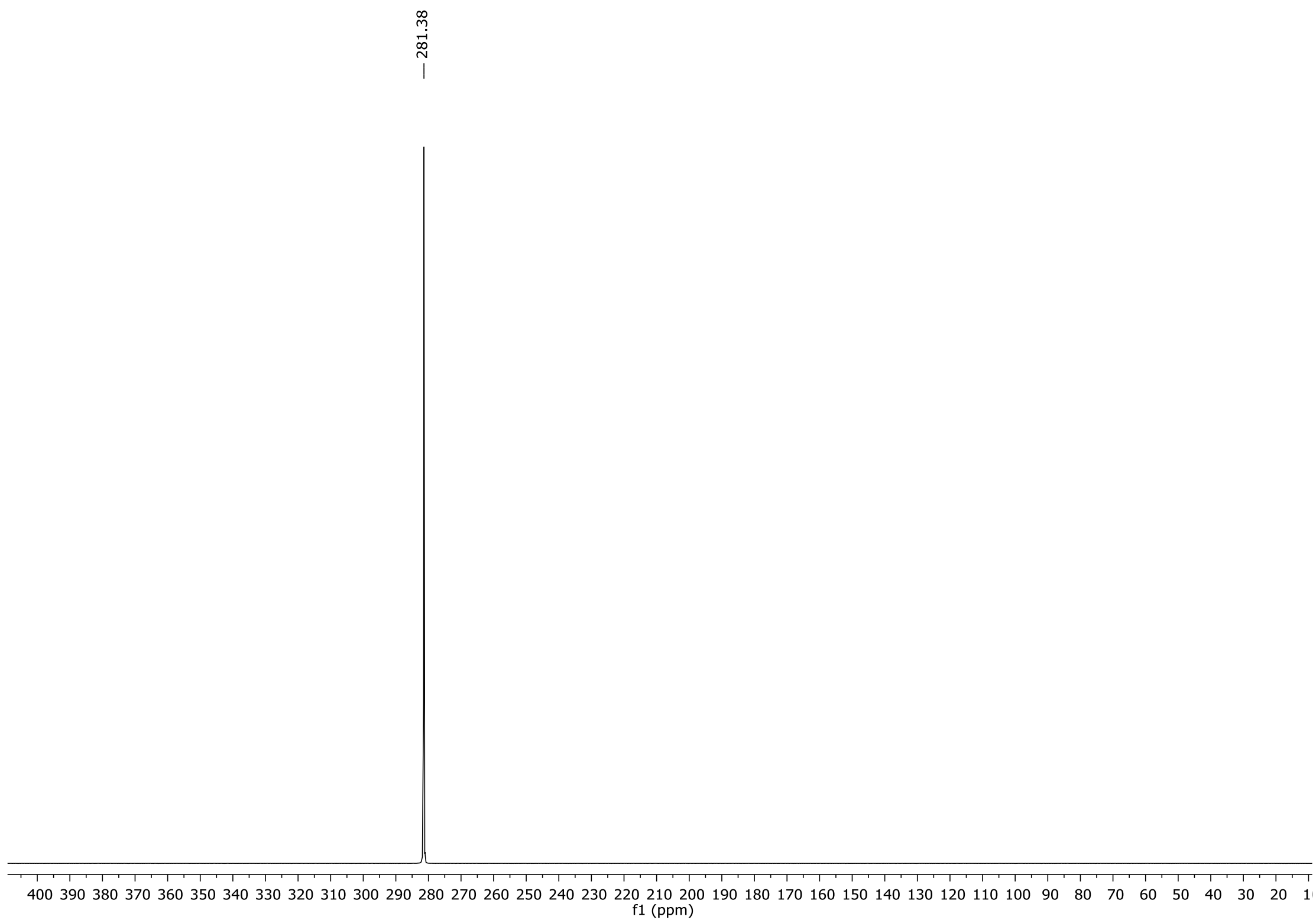

Object	Integral [abs]	Integral [rel]	Range (F1) from	Range (F1) to	ν(F1) [ppm]
Integral 1	3926222.356	1.00002464	282.4211877	280.8163358	281.38

Figure S12. ^{15}N-hyperpolarization of pyridine-N-oxide **2** (2.5 mM) and the corresponding integrations.

A hyperpolarization efficiency of 27.6 % was achieved with this sample. (3rd measurement).

Object	Effi (%)1	Effi (%)2	Effi (%)3	AVR Effi (%)1
Integral 1	29.1	26.6	27.6	**28±2**

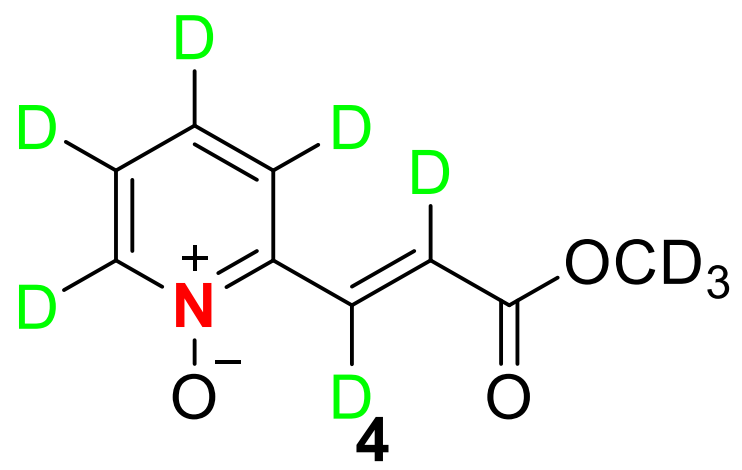

4

Hyperpolarization conditions: **4** / [Rh(dppb)(COD)][BF_4] 15:1, MeOD-d_4 (0.2 mL). pH_2 bubbling time = 9 s, 330 K, J_{HH}= 7.1 Hz, J_{NH}=3.75 Hz.

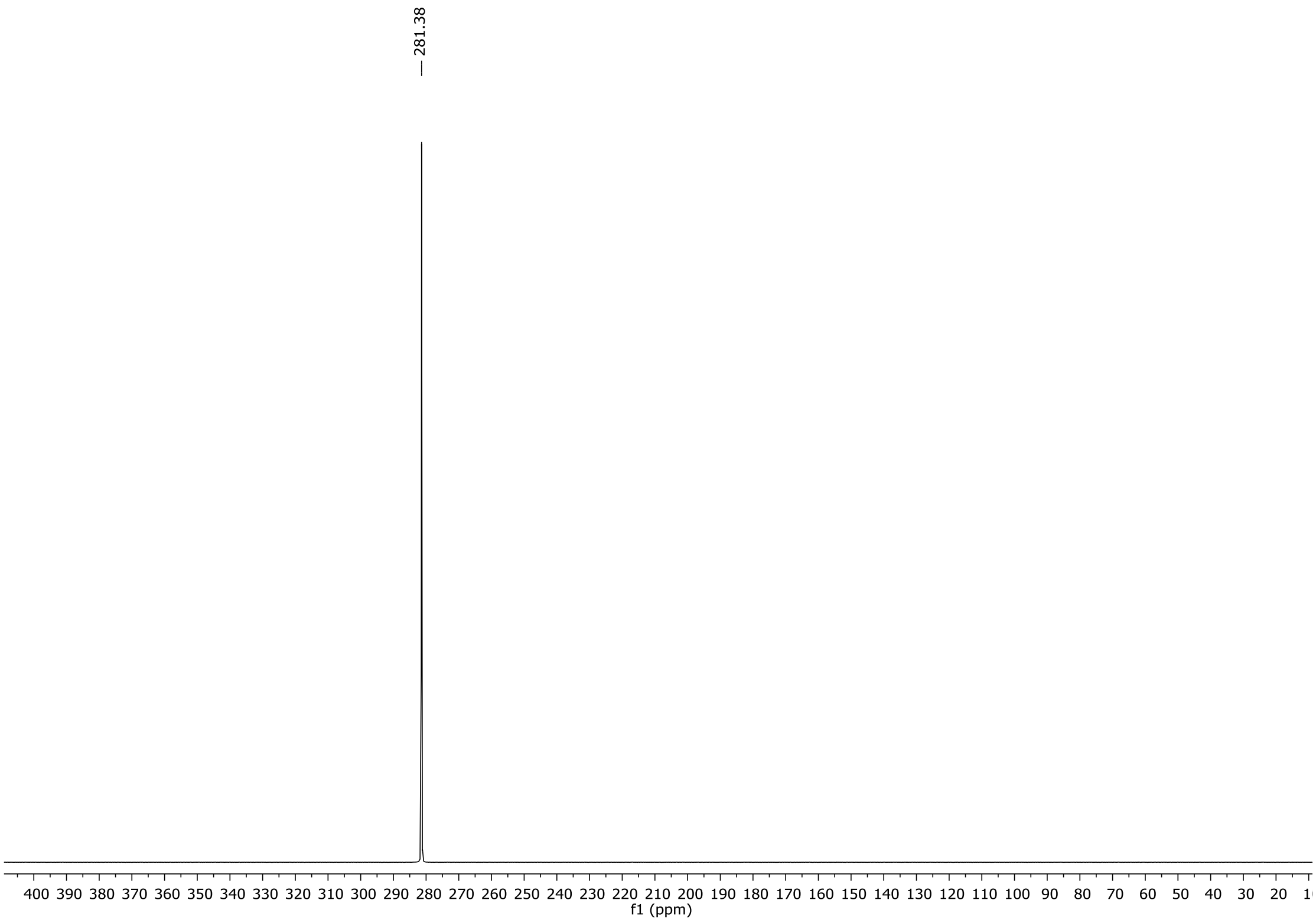

Object	Integral [abs]	Integral [rel]	Range (F1) from	Range (F1) to	ν(F1) [ppm]
Integral 1	3475711.20	1.0	281.95	280.88	281.42

Figure S13. ^{15}N-hyperpolarization of pyridine-N-oxide **2** (2.5 mM) and the corresponding integrations. A hyperpolarization efficiency of 24.4 % was achieved with this sample. (1st measurement)

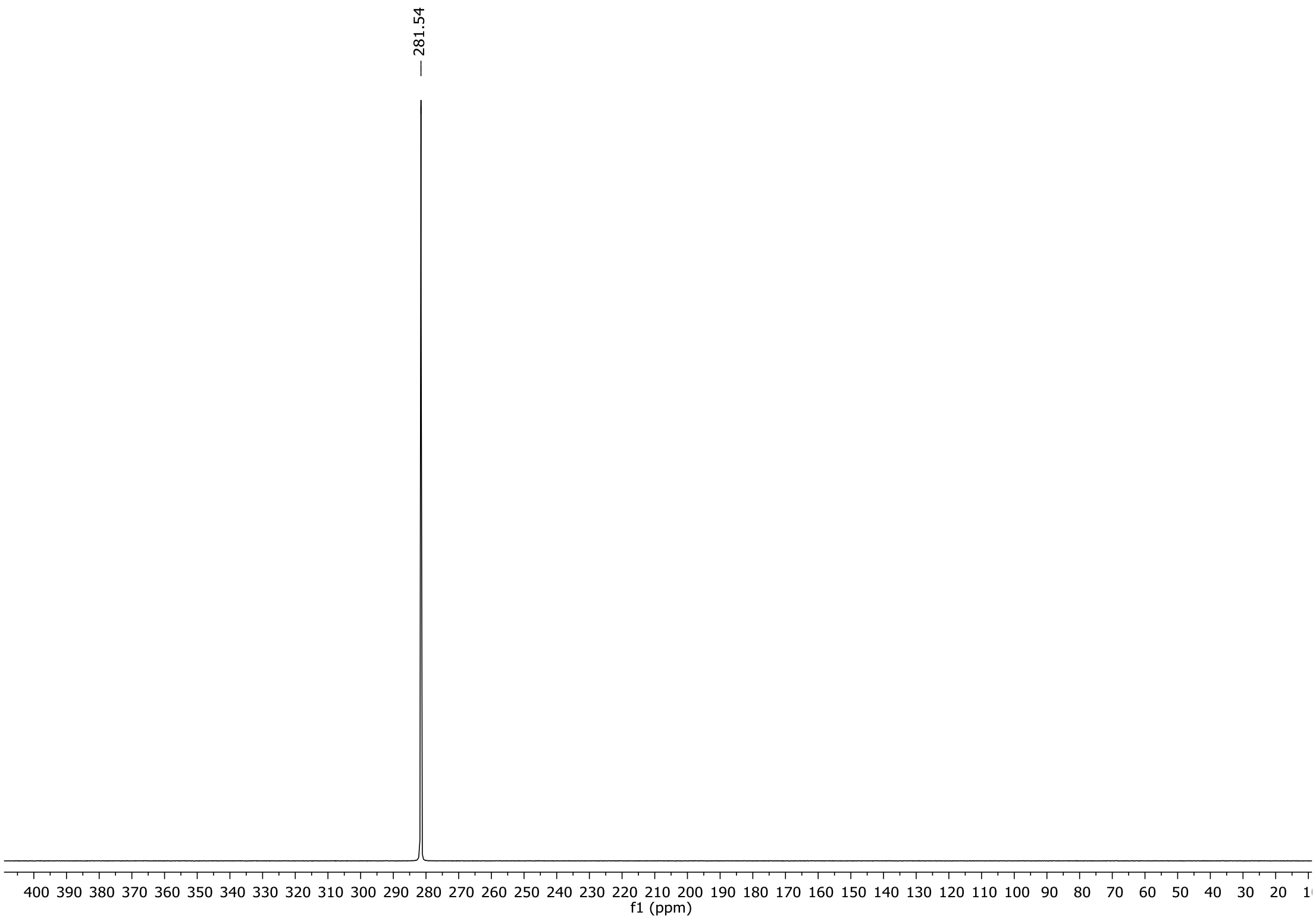

Object	Integral [abs]	Integral [rel]	Range (F1) from	Range (F1) to	ν(F1) [ppm]
Integral 1	3285482.49	1	282.35	280.82	281.58

Figure S14. ^{15}N-hyperpolarization of pyridine-N-oxide **2** (2.5 mM) and the corresponding integrations. A hyperpolarization efficiency of 23.1 % was achieved with this sample. (2nd measurement)

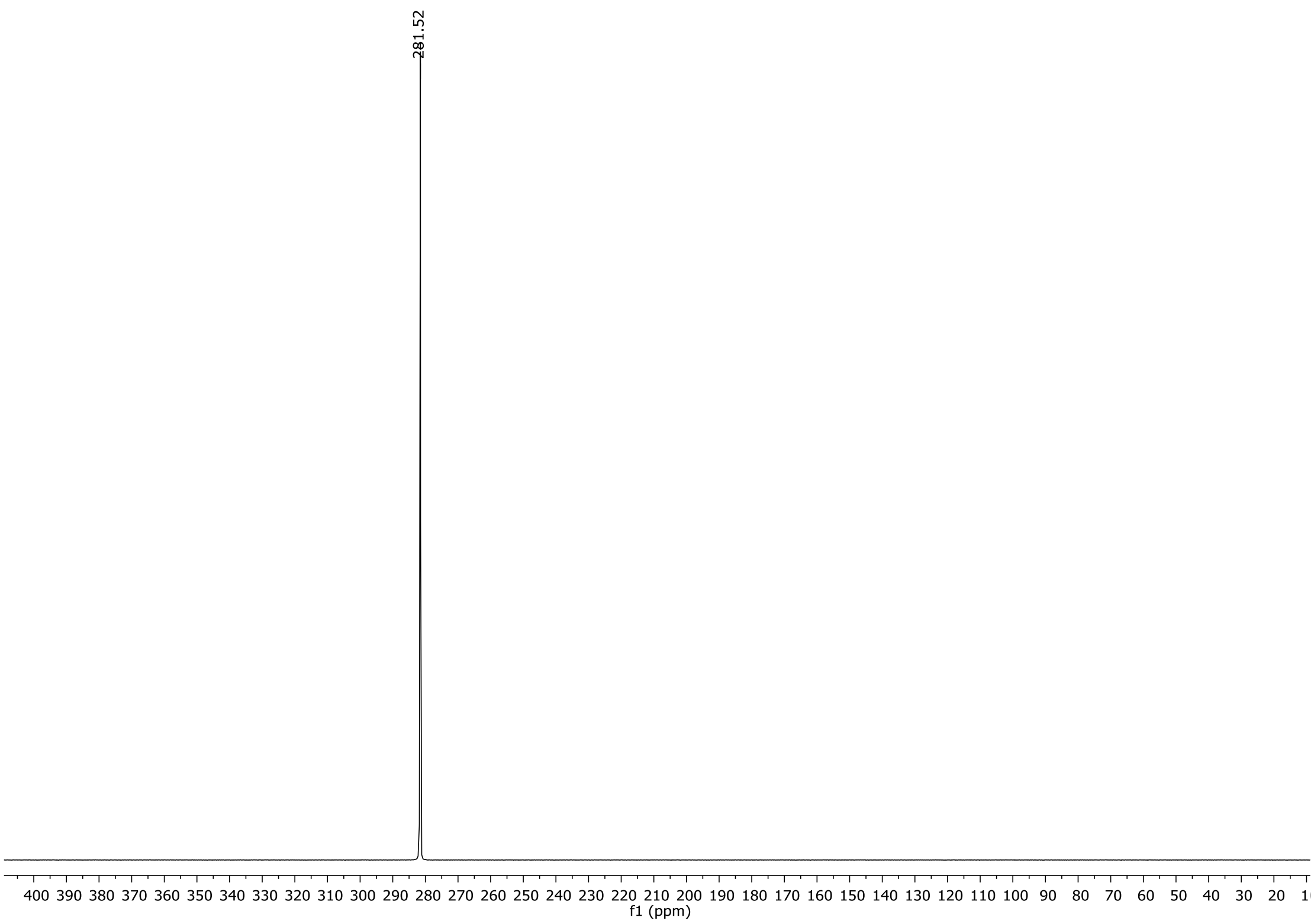

Object	Integral [abs]	Integral [rel]	Range (F1) from	Range (F1) to	ν(F1) [ppm]
Integral 1	3646876.65	1	282.61	280.84	281.73

Figure S15. ^{15}N-hyperpolarization of pyridine-N-oxide **2** (2.5 mM) and the corresponding integrations. A hyperpolarization efficiency of 25.6 % was achieved with this sample. (3rd measurement)

Object	Effi (%)1	Effi (%)2	Effi (%)3	AVR Effi (%)
Integral 1	24.4	23.1	25.6	**24±2**

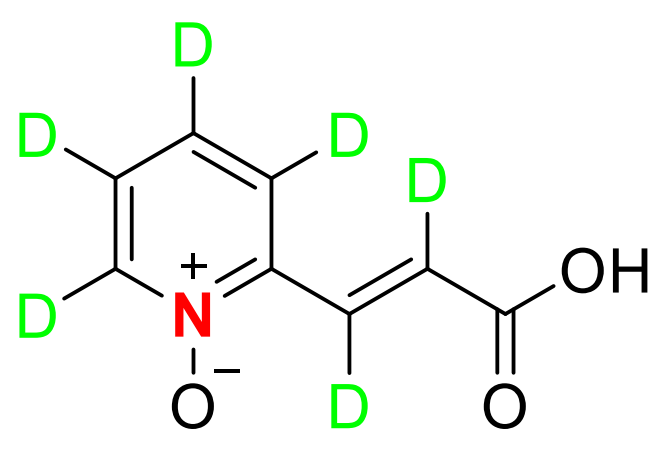

S-5

Hyperpolarization conditions: **S-5** / [Rh(dppb)(COD)][BF_4] =10~20:1, MeOD-d_4 (0.2 mL). pH_2 bubbling time = 9 s, 330 K, J_{HH}= 7.1 Hz, J_{NH}=3.75 Hz.

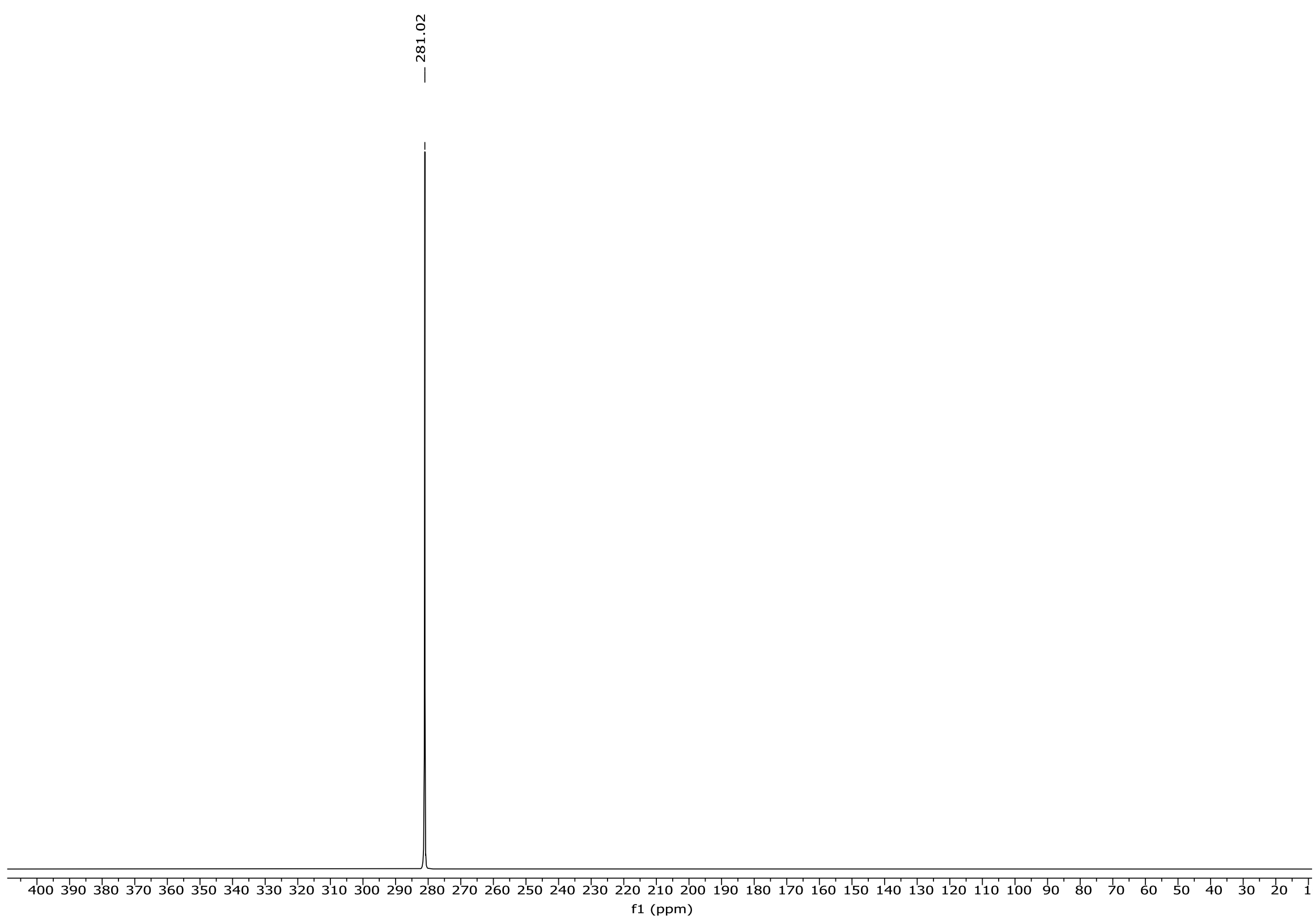

Object	Integral [abs]	Integral [rel]	Range (F1) from	Range (F1) to	?(F1) [ppm]
Integral 1	2518178	1	281.923	280.405	281.1636

Figure S16. ^{15}N-hyperpolarization of pyridine-N-oxide **S-5** (2.5 mM) and the corresponding integrations. A hyperpolarization efficiency of **17.7** % was achieved with this sample. (1st measurement), **S-5** / [Rh(dppb)(COD)][BF_4] =10:1

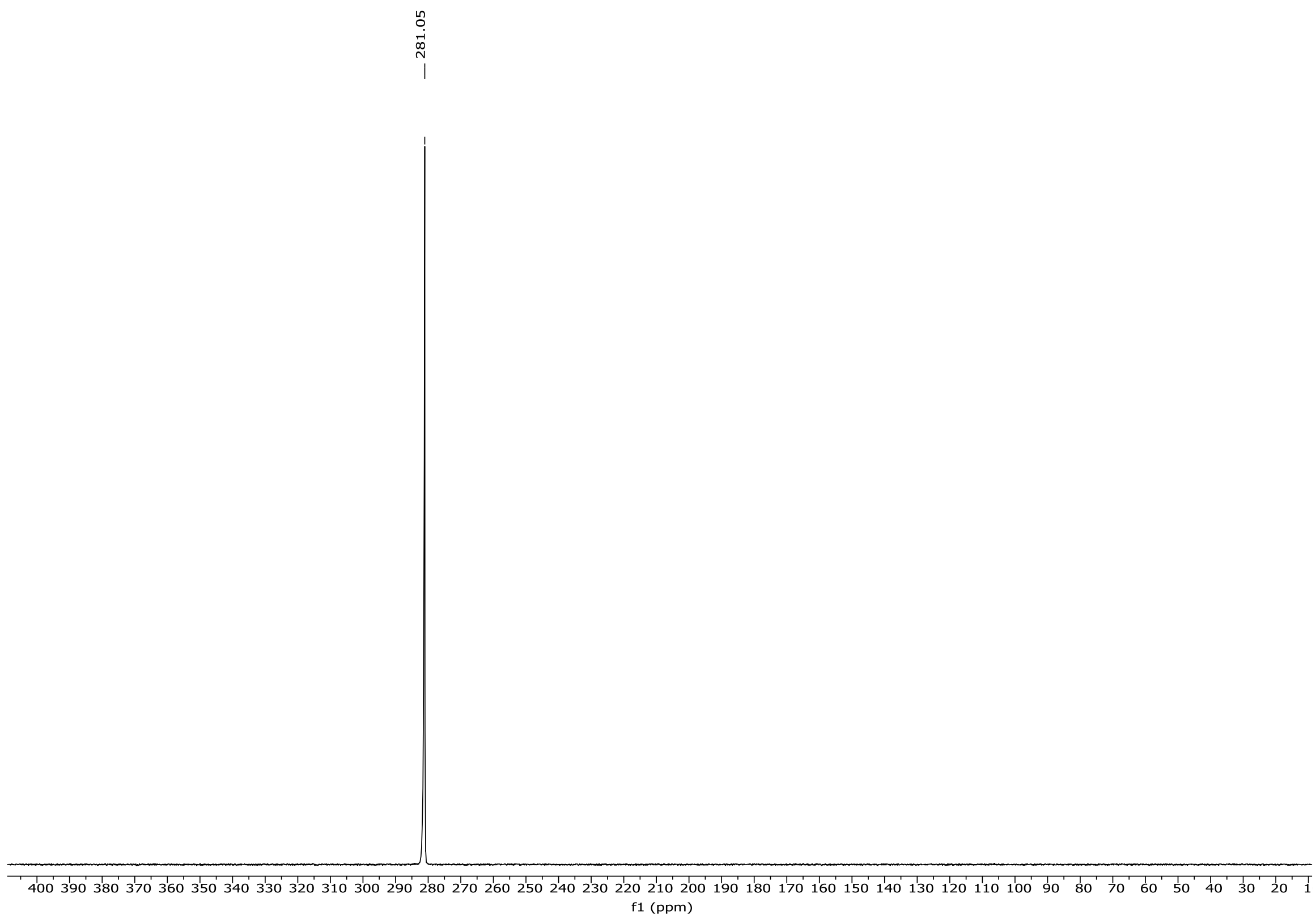

Object	Integral [abs]	Integral [rel]	Range (F1) from	Range (F1) to	?(F1) [ppm]
Integral 1	1105775	1	284.481	278.62	281.5505

Figure S17. ^{15}N-hyperpolarization of pyridine-N-oxide **S-5** (2.5 mM) and the corresponding integrations. A hyperpolarization efficiency of **7.8** % was achieved with this sample. (1st measurement), **S-5** / [Rh(dppb)(COD)][BF_4] =15:1

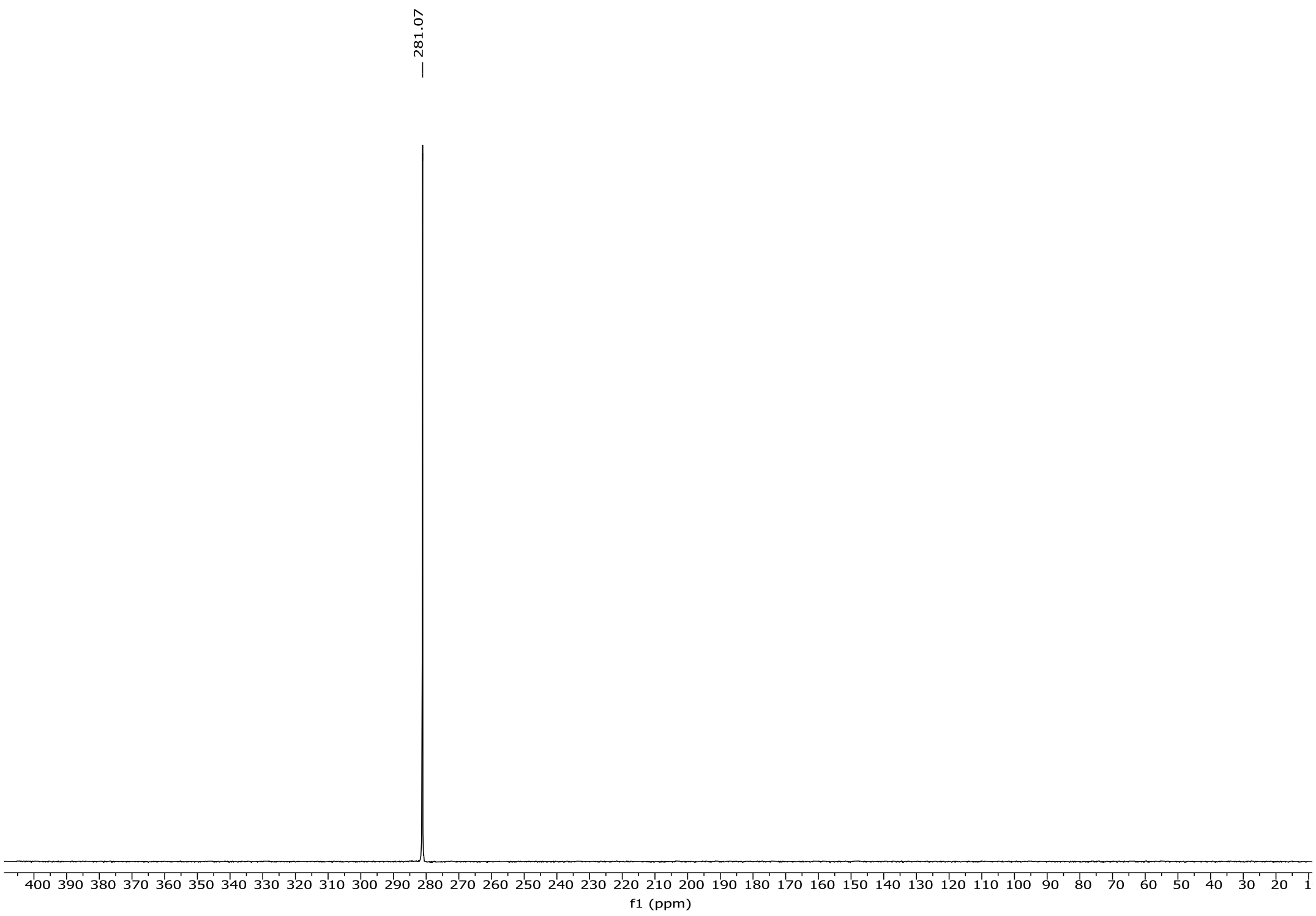

Object	Integral [abs]	Integral [rel]	Range (F1) from	Range (F1) to	?(F1) [ppm]
Integral 1	566560.8	1	282.446	280.314	281.3799

Figure S18 ^{15}N-hyperpolarization of pyridine-N-oxide **S-5** (2.5 mM) and the corresponding integrations. A hyperpolarization efficiency of **4.0** % was achieved with this sample. (1st measurement), **S-5** / [Rh(dppb)(COD)][BF_4] =20:1

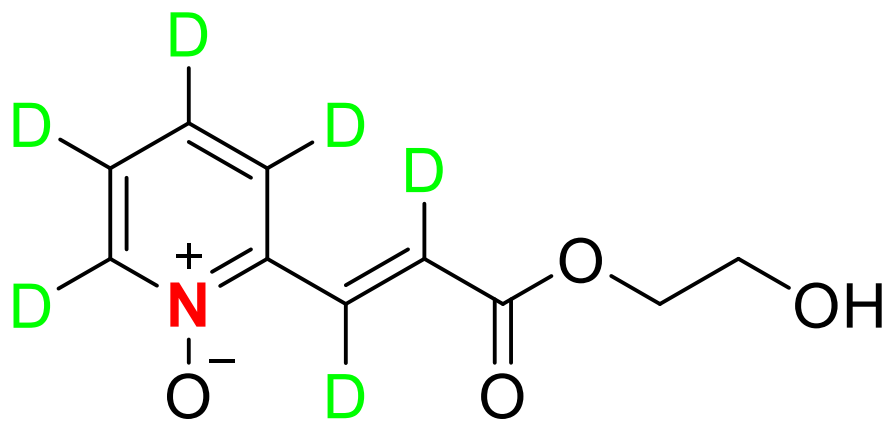

S-6

Hyperpolarization conditions: **S-6** / [Rh(dppb)(COD)][BF_4] =2~3:1, MeOD-d_4 (0.2 mL). pH_2 bubbling time = 9 s, 330 K, J_{HH}= 7.1 Hz, J_{NH}=3.75 Hz.

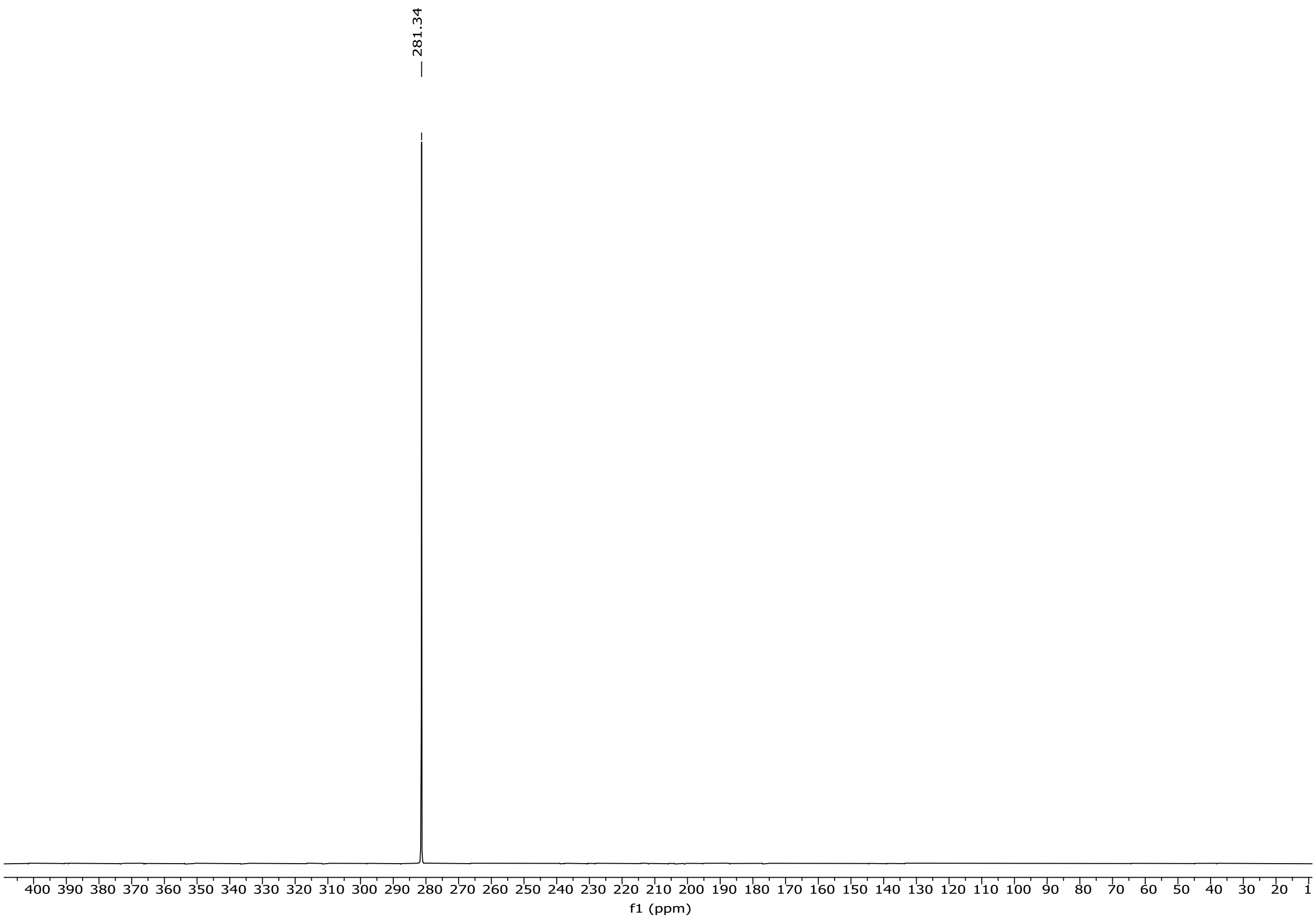

Object	Integral [abs]	Integral [rel]	Range (F1) from	Range (F1) to	?(F1) [ppm]
Integral 1	742301.6	1	282.193	280.704	281.4485

Figure S19 ^{15}N-hyperpolarization of pyridine-N-oxide **S-6** (1.81 mM) and the corresponding integrations. A hyperpolarization efficiency of **7.2** % was achieved with this sample. (1st measurement), **S-6** / [Rh(dppb)(COD)][BF_4] =2:1

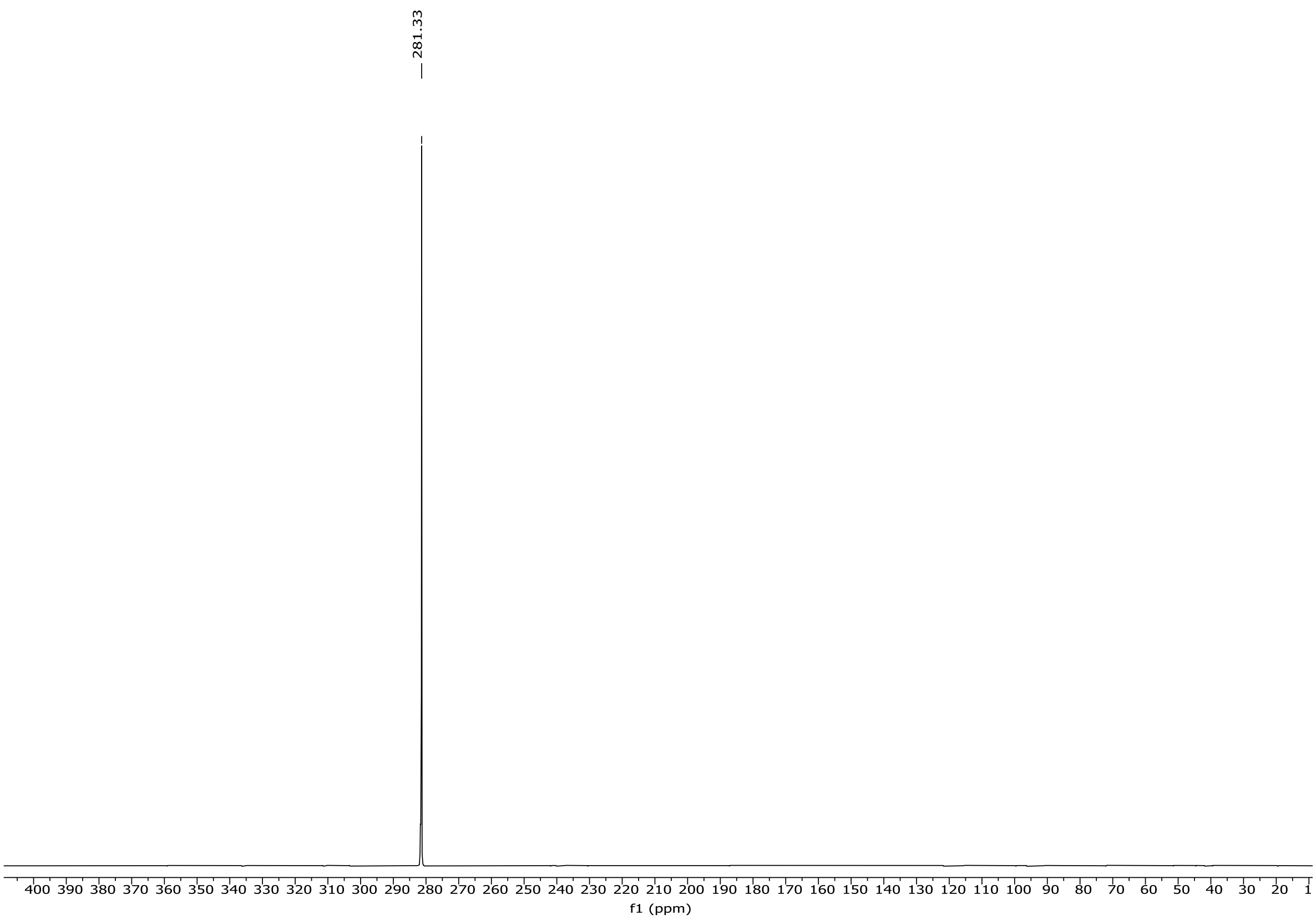

Object	Integral [abs]	Integral [rel]	Range (F1) from	Range (F1) to	?(F1) [ppm]
Integral 1	981323	1	283.94	279.614	281.7768

Figure S20 ^{15}N-hyperpolarization of pyridine-N-oxide **S-6** (1.92 mM) and the corresponding integrations. A hyperpolarization efficiency of **9.5** % was achieved with this sample. (2nd measurement), **S-6** / [Rh(dppb)(COD)][BF_4] =3:1

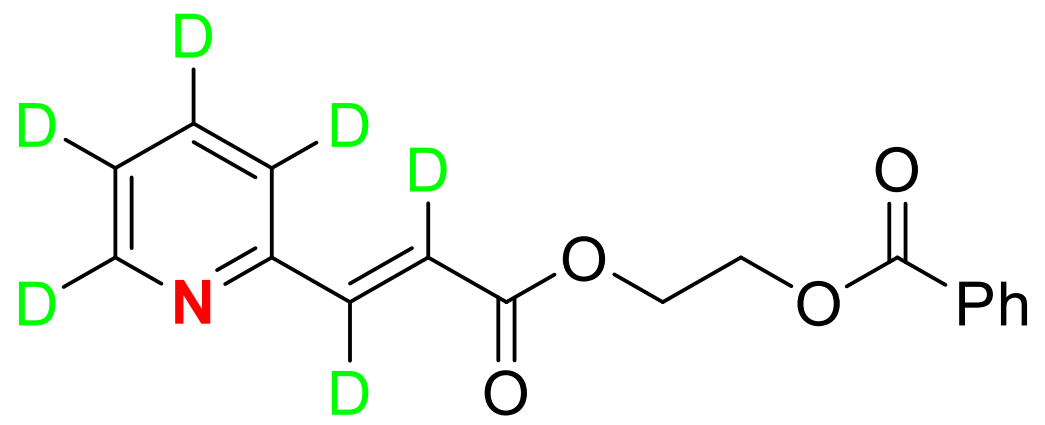

5

Hyperpolarization conditions: **5** / [Rh(dppb)(COD)][BF_4] 10:1, MeOD-d_4 (0.2 mL). pH_2 bubbling time = 9 s, 330 K, J_{HH}= 7.1 Hz, J_{NH}=3.75 Hz.

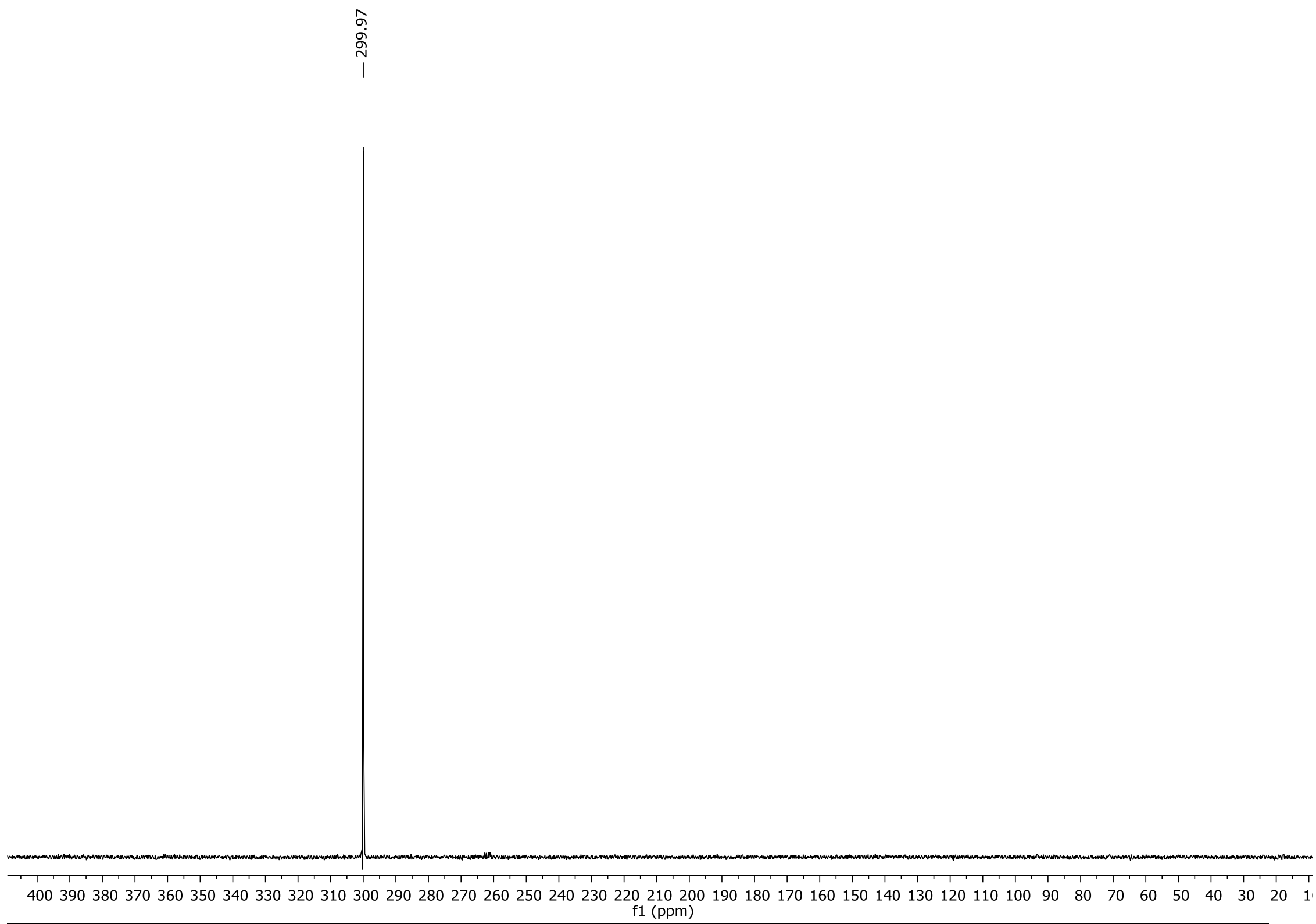

Object	Integral [abs]	Integral [rel]	Range (F1) from	Range (F1) to	ν(F1) [ppm]
Integral 1	181156.3345	0.999999996	300.3794046	299.5088973	299.944151

Figure S21. ^{15}N-hyperpolarization of pyridine-N-oxide **2** (2.5 mM) and the corresponding integrations. A hyperpolarization efficiency of 1.27 % was achieved with this sample. (1st measurement).

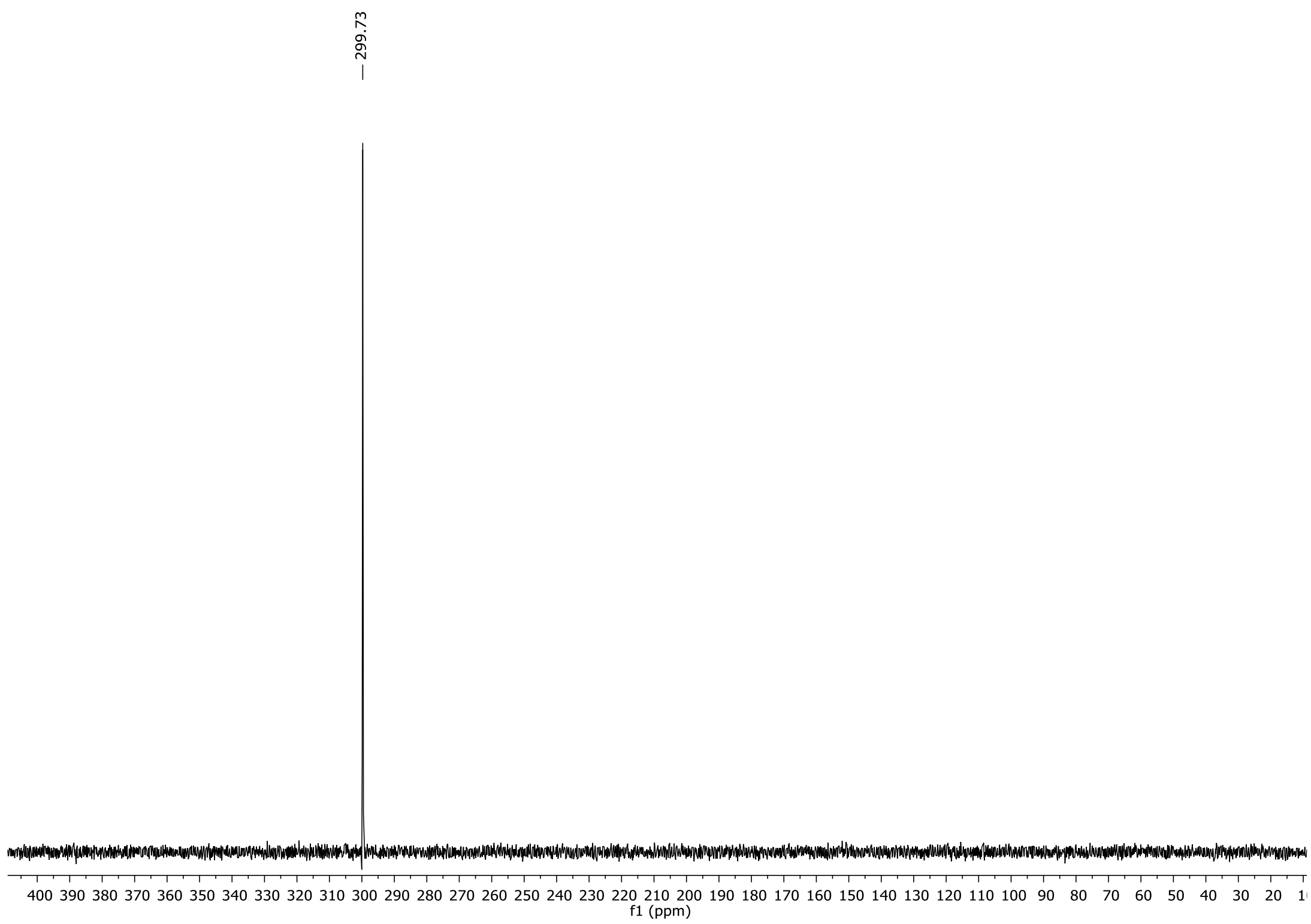

Object	Integral [abs]	Integral [rel]	Range (F1) from	Range (F1) to	ν(F1) [ppm]
Integral 1	62418.7798	0.999999983	300.1928284	299.0873054	299.6400669

Figure S22. ^{15}N-hyperpolarization of pyridine-N-oxide **2** (2.5 mM) and the corresponding integrations. A hyperpolarization efficiency of 0.44 % was achieved with this sample. (2nd measurement).

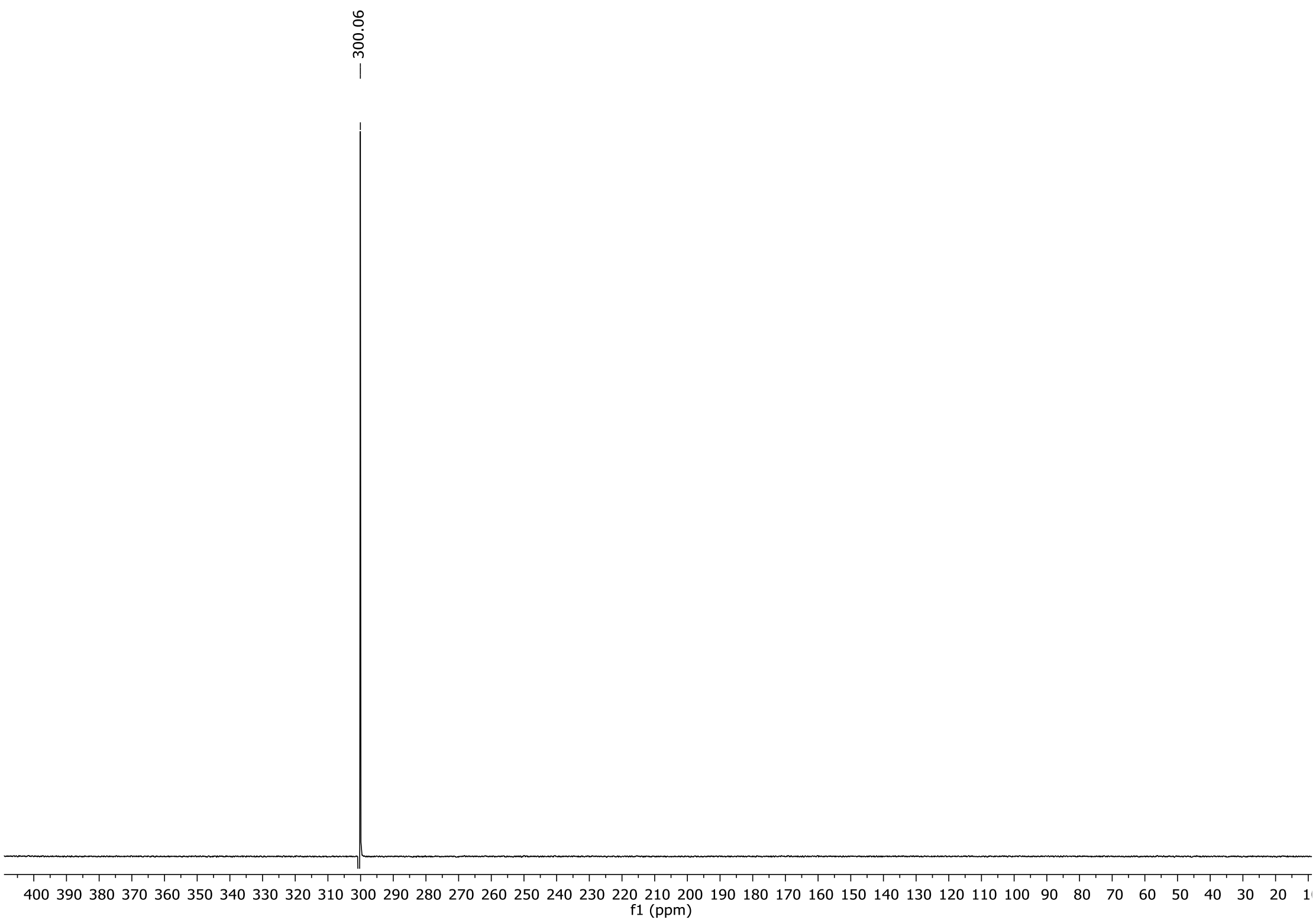

Object	Integral [abs]	Integral [rel]	Range (F1) from	Range (F1) to	ν(F1) [ppm]
Integral 1	435075.2716	1.000002287	300.2976515	299.174585	299.7361183

Figure S23. ^{15}N-hyperpolarization of pyridine-N-oxide **2** (3.33 mM) and the corresponding integrations. A hyperpolarization efficiency of 2.30 % was achieved with this sample. (3rd measurement).

Object	Effi (%)1	Effi (%)2	Effi (%)3	AVR Effi (%)
Integral 1	1.27	0.44	2.30	1±1

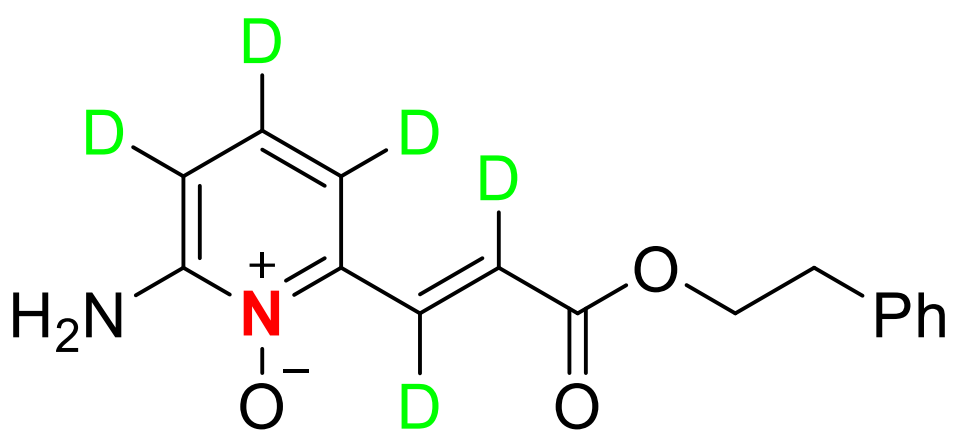

6

Hyperpolarization conditions: **6** / [Rh(dppb)(COD)][BF_4] 15:1, MeOD-d_4 (0.2 mL). pH_2 bubbling time = 9 s, 330 K, J_{HH}= 7.1 Hz, J_{NH}=3.75 Hz.

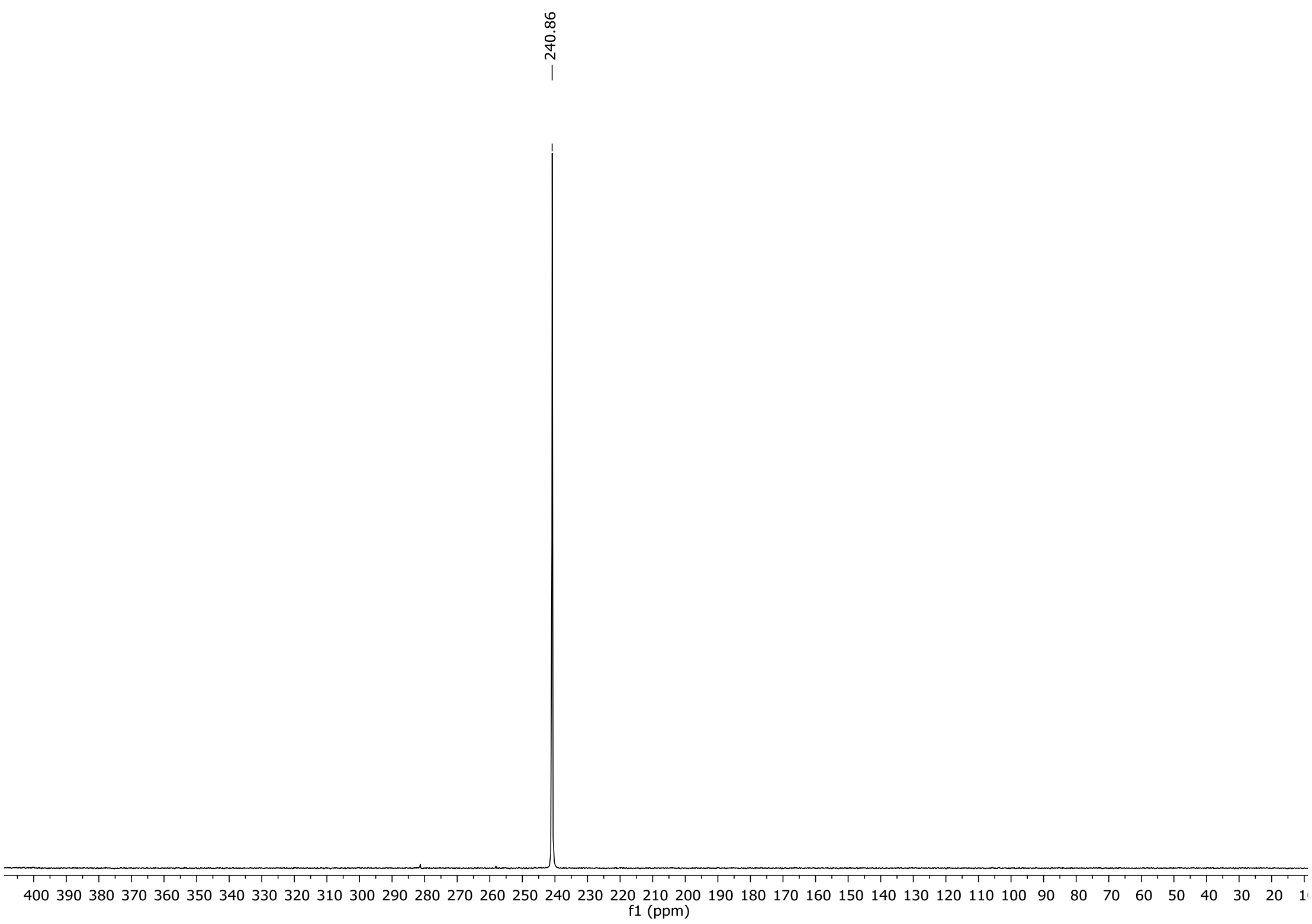

Object	Integral [abs]	Integral [rel]	Range (F1) from	Range (F1) to	ν(F1) [ppm]
Integral 1	1256740.27	1.0	245.70	237.75	241.73

Figure S24. ^{15}N-hyperpolarization of pyridine-N-oxide **6** (2.5 mM) and the corresponding integrations. A hyperpolarization efficiency of 8.83% was achieved with this sample. (1st measurement).

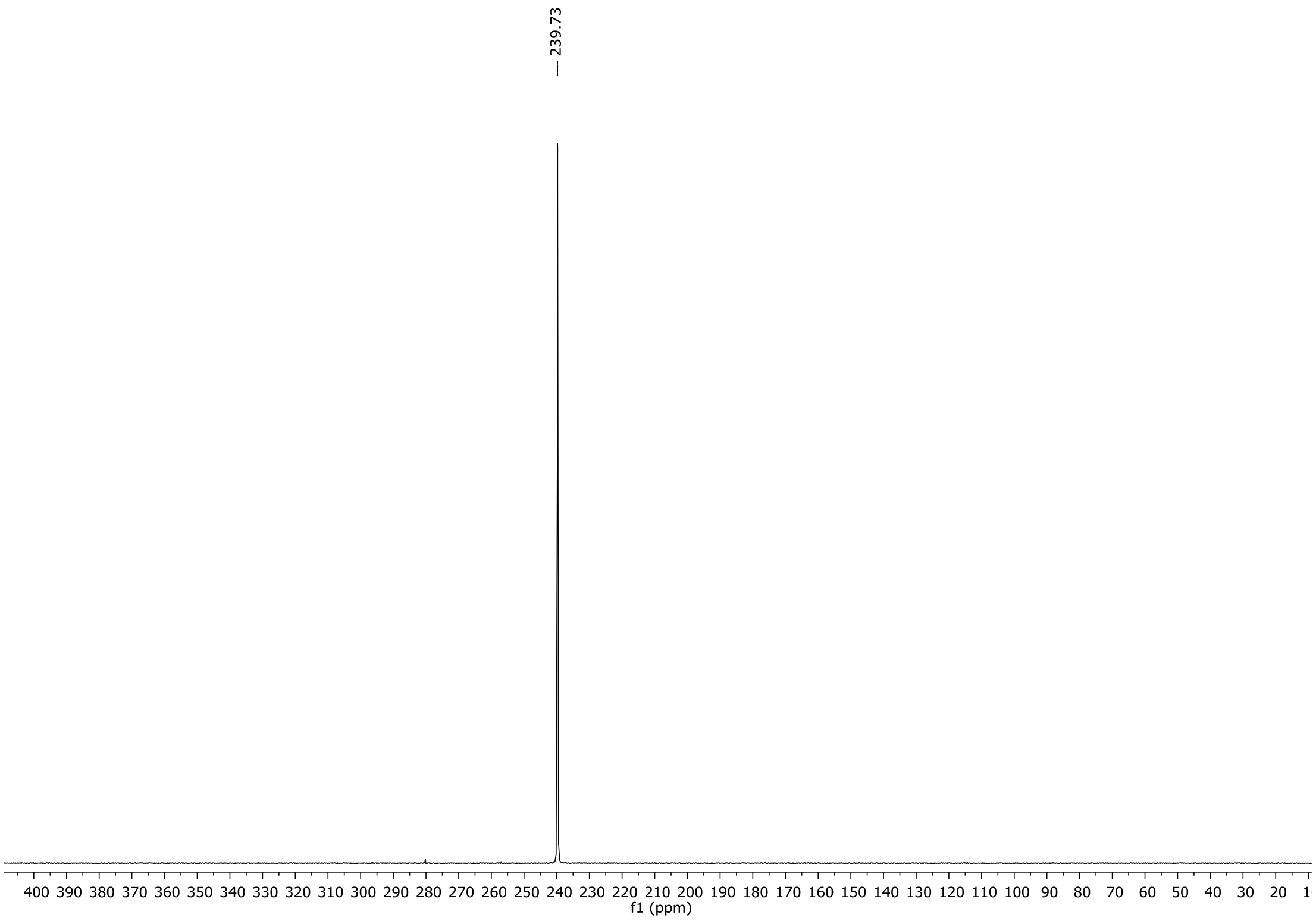

Object	Integral [abs]	Integral [rel]	Range (F1) from	Range (F1) to	ν(F1) [ppm]
Integral 1	1188281.00	1	240.73	239.016	239.87

Figure S25. ^{15}N-hyperpolarization of pyridine-N-oxide **6** (2.5 mM) and the corresponding integrations. A hyperpolarization efficiency of 8.35% was achieved with this sample. (2nd measurement).

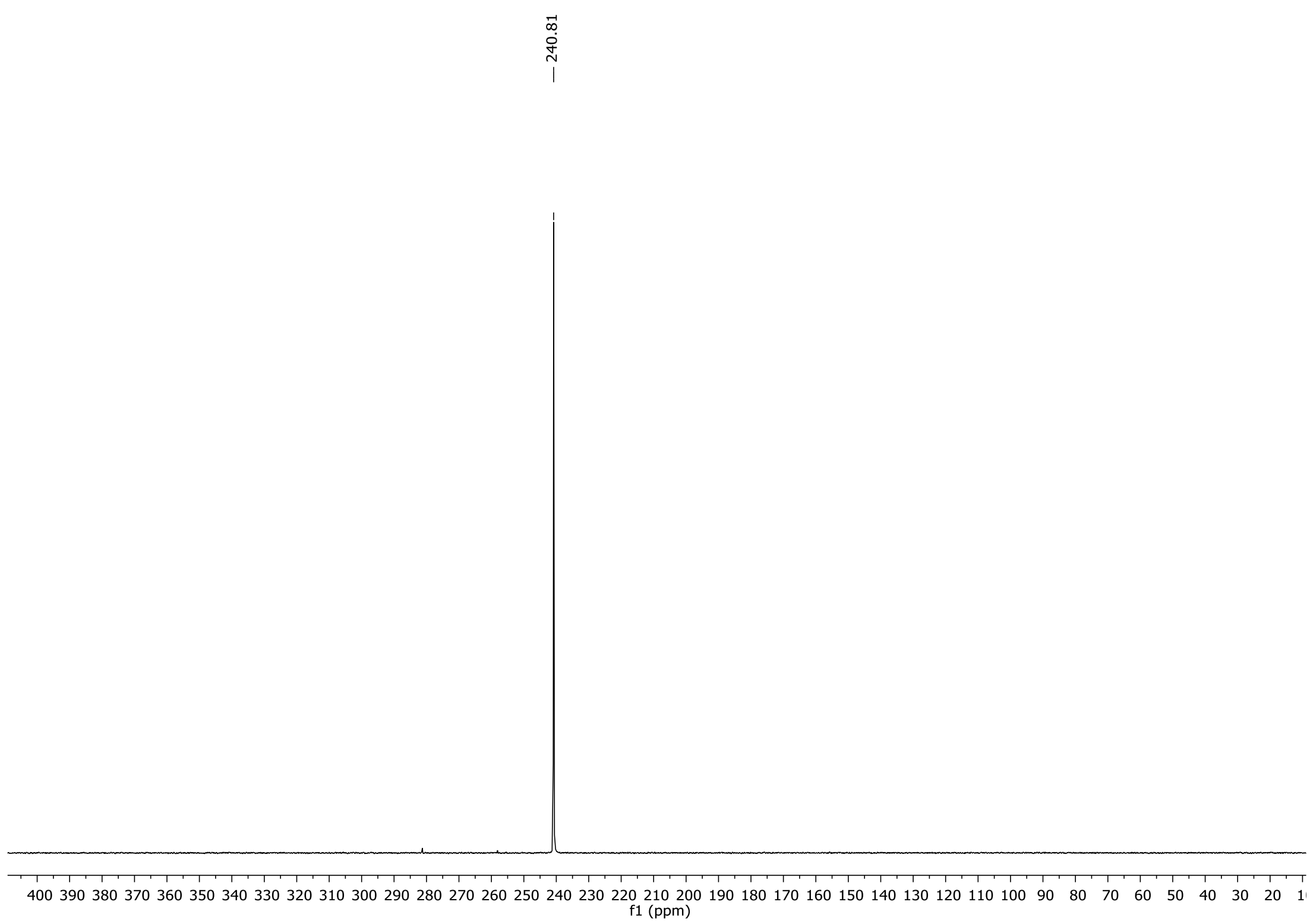

Object	Integral [abs]	Integral [rel]	Range (F1) from	Range (F1) to	ν(F1) [ppm]
Integral 1	610136.50	1	241.38	240.01	240.69

Figure S26. ^{15}N-hyperpolarization of pyridine-N-oxide **6** (2.5 mM) and the corresponding integrations. A hyperpolarization efficiency of 4.3 % was achieved with this sample. (3rd measurement)

Object	Effi (%)1	Effi (%)2	Effi (%)3	AVR Effi (%)
Integral 1	8.83	8.35	4.3	7±3

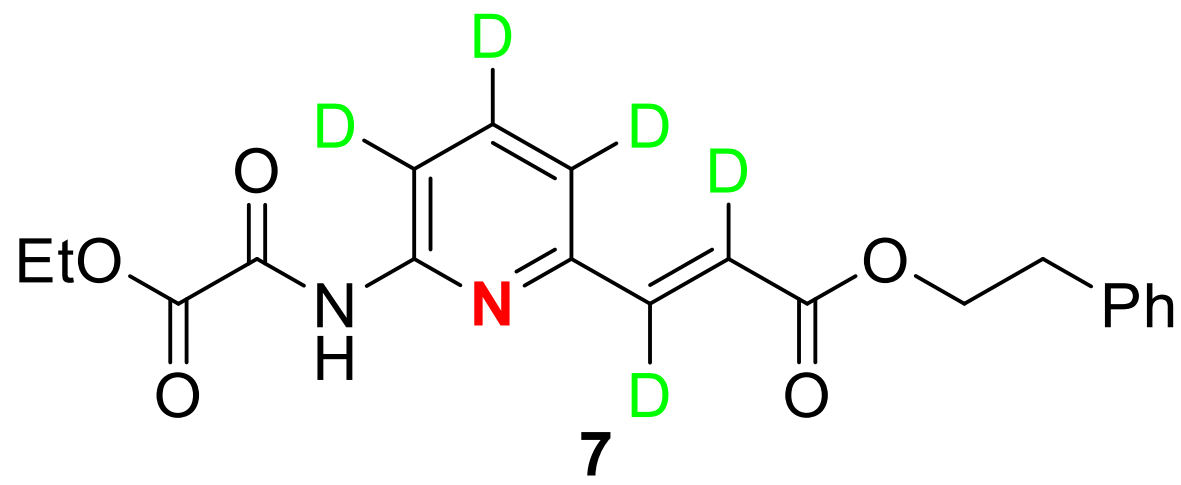

7

Hyperpolarization conditions: **7** / $[Rh(dppb)(COD)][BF_4]$ 10:1, MeOD-d_4 (0.2 mL). pH_2 bubbling time = 9 s, 330 K, J_{HH}= 7.1 Hz, J_{NH}=3.75 Hz.

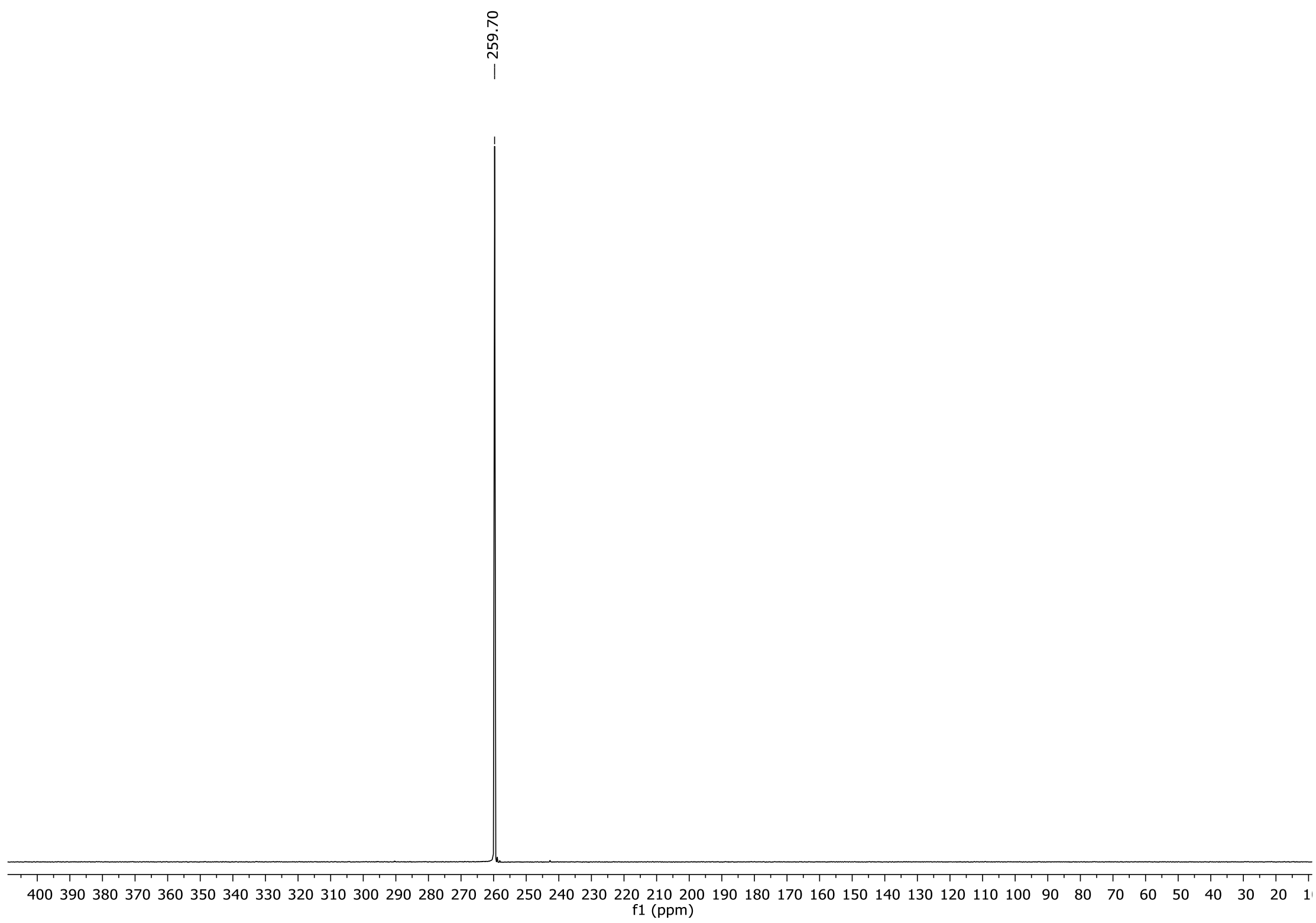

Object	Integral [abs]	Integral [rel]	Range (F1) from	Range (F1) to	ν(F1) [ppm]
Integral 1	1476436.33	1	260.37	259.32	259.85

Figure S27. ^{15}N-hyperpolarization of pyridine-N-oxide **7** (2.22 mM) and the corresponding integrations. A hyperpolarization efficiency of 11.7 % was achieved with this sample. (1st measurement).

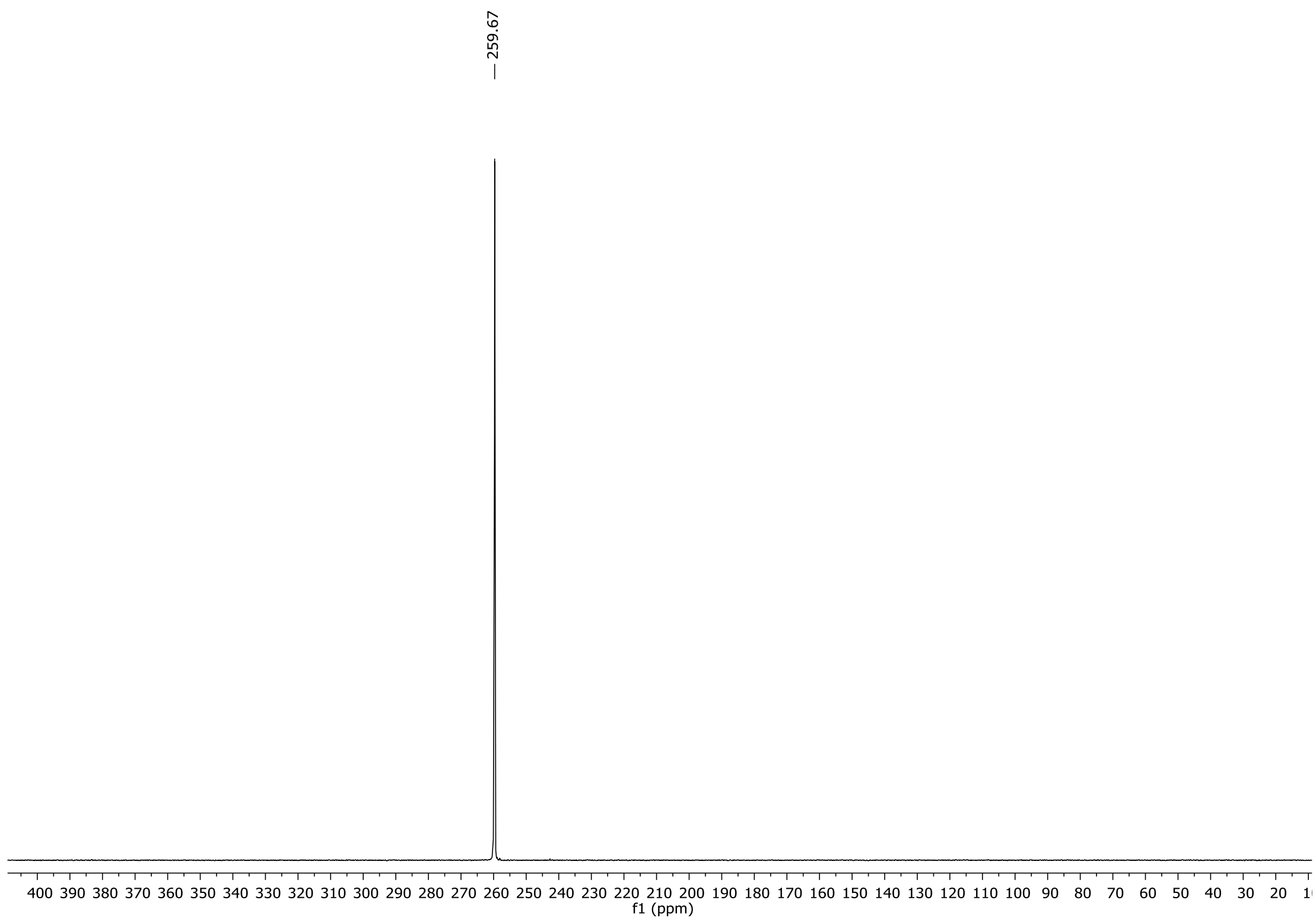

Object	Integral [abs]	Integral [rel]	Range (F1) from	Range (F1) to	ν(F1) [ppm]
Integral 1	1089640.06	1	261.05	258.84	259.94

Figure S28. ^{15}N-hyperpolarization of pyridine-N-oxide **7** (2.34 mM) and the corresponding integrations. A hyperpolarization efficiency of 8.18 % was achieved with this sample. (2nd measurement).

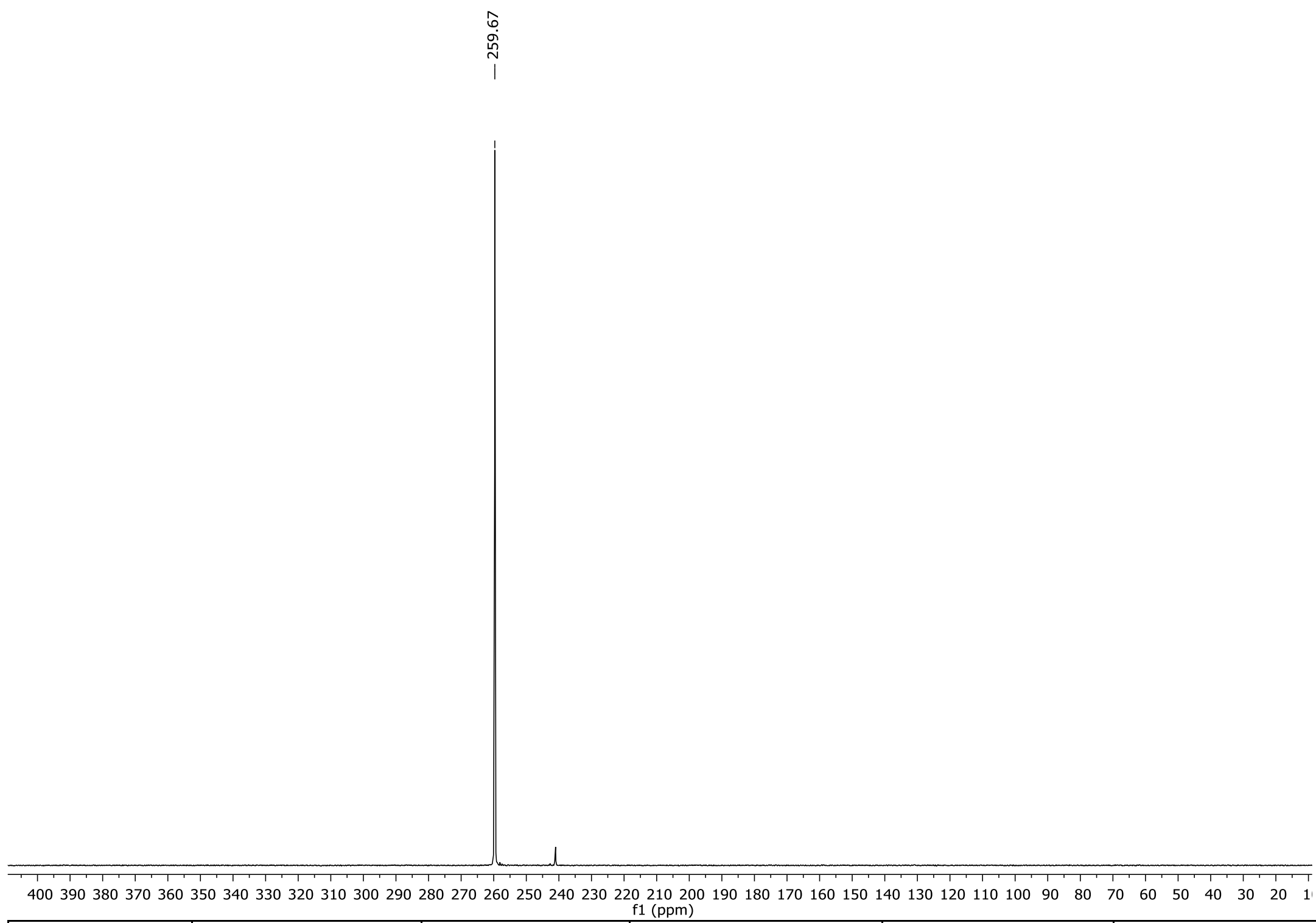

Object	Integral [abs]	Integral [rel]	Range (F1) from	Range (F1) to	ν(F1) [ppm]
Integral 1	1035865.51	1	260.91	258.74	259.82

Figure S29. ^{15}N-hyperpolarization of pyridine-N-oxide **7** (2.0 mM) and the corresponding integrations. A hyperpolarization efficiency of 9.1 % was achieved with this sample. (3rd measurement).

Object	Effi (%)1	Effi (%)2	Effi (%)3	AVR Effi (%)
Integral 1	11.7	8.18	9.1	10±2

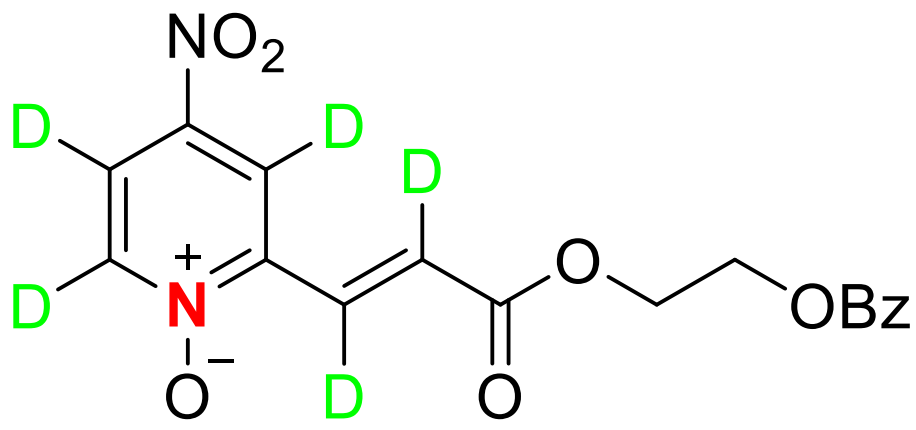

8

Hyperpolarization conditions: **8** / [Rh(dppb)(COD)][BF_4] 10:1, **MeOD-d$_4$** (0.2 mL). pH_2 bubbling time = 9 s, 330 K, J_{HH}= 7.1 Hz, J_{NH}=3.75 Hz.

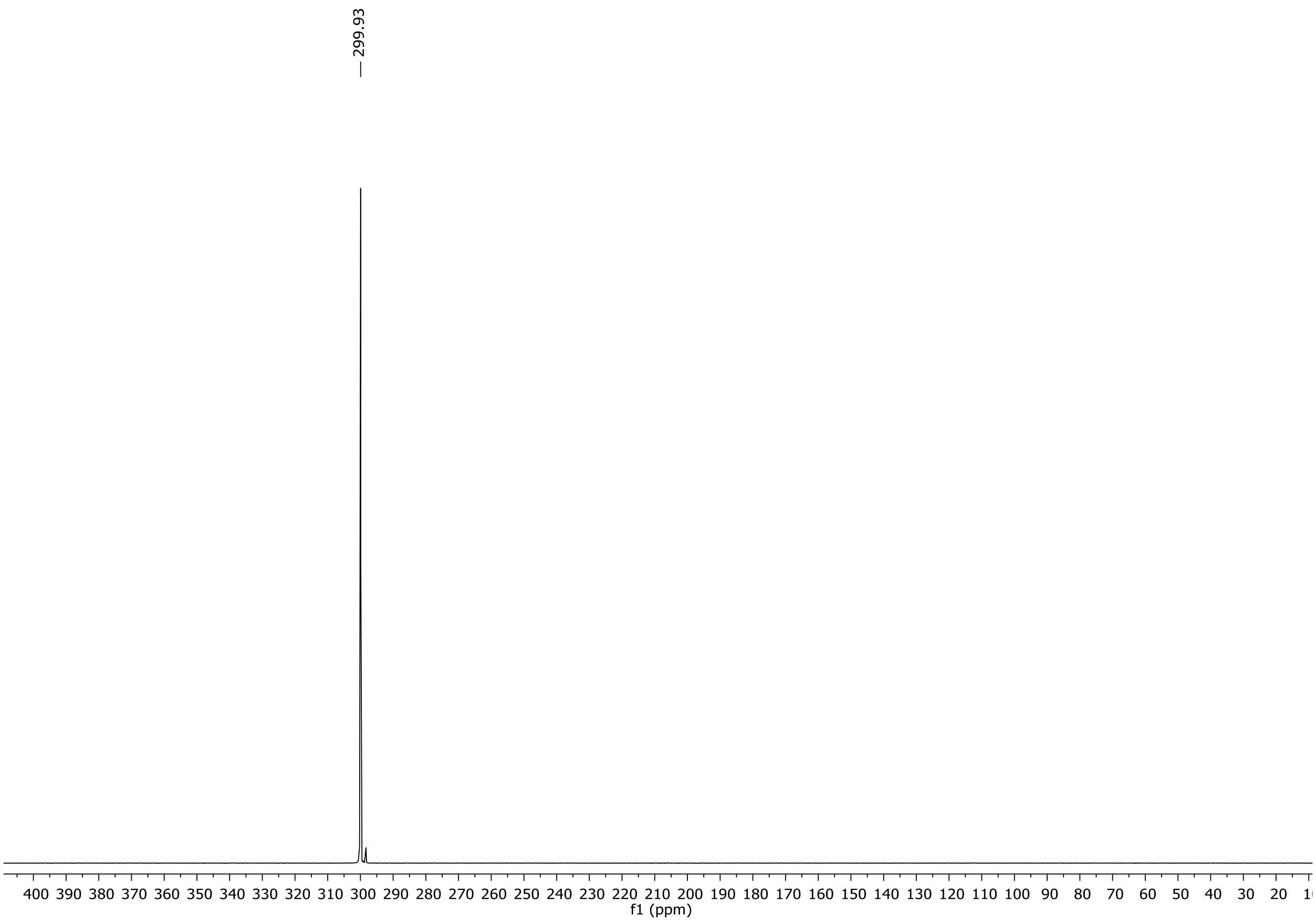

Object	Integral [abs]	Integral [rel]	Range (F1) from	Range (F1) to	ν(F1) [ppm]
Integral 1	2457864.709	1.000000244	300.8348233	299.2061858	300.0205046

Figure S30 ^{15}N-hyperpolarization of pyridine-N-oxide **8** (2.5 mM) and the corresponding integrations. A hyperpolarization efficiency of 17.3 % was achieved with this sample. (1st measurement)

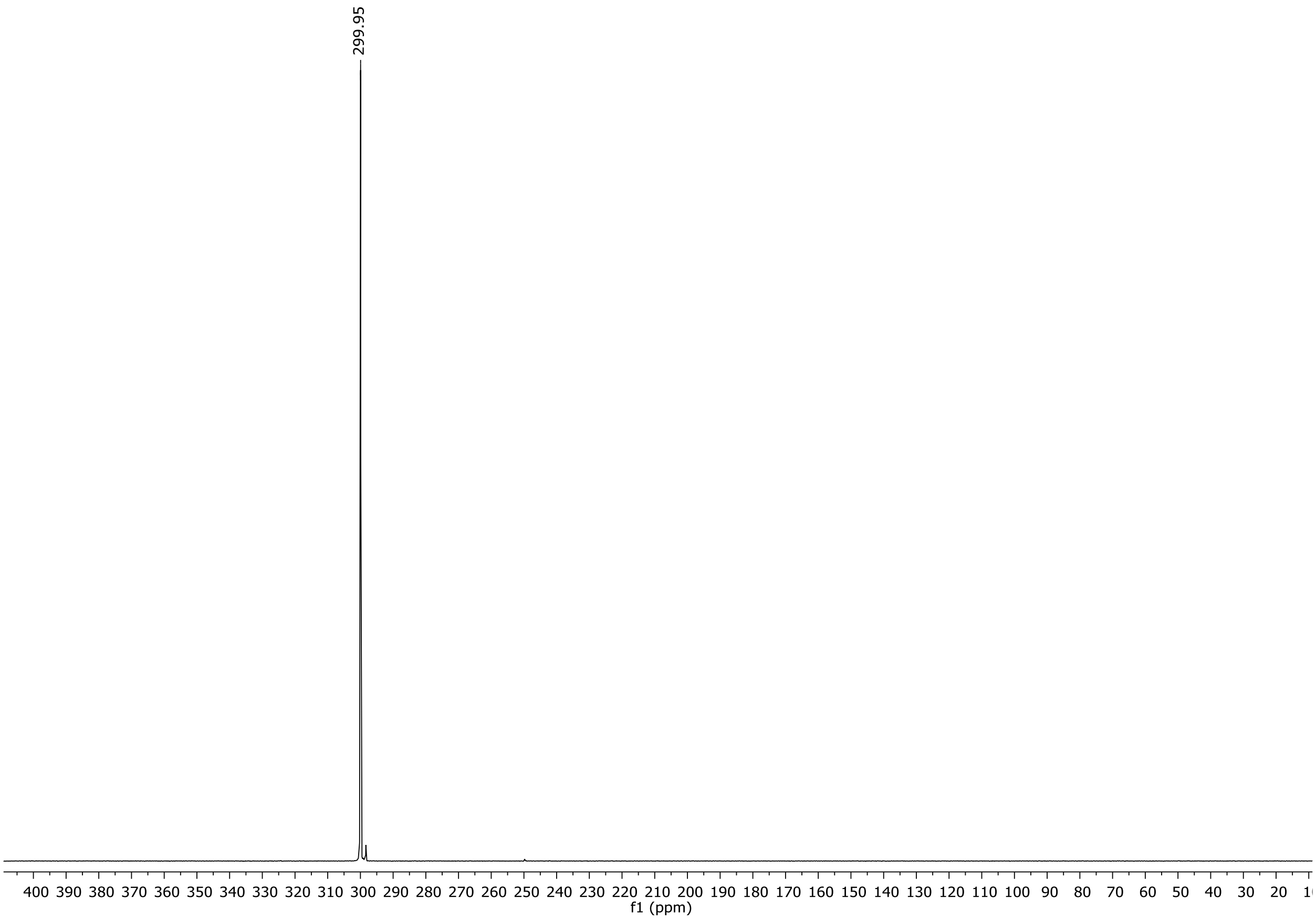

Object	Integral [abs]	Integral [rel]	Range (F1) from	Range (F1) to	ν(F1) [ppm]
Integral 1	2776794.666	1.0000000	301.5506164	298.9779392	300.2642778

Figure S31. ^{15}N-hyperpolarization of pyridine-N-oxide **8** (2.5 mM) and the corresponding integrations. A hyperpolarization efficiency of 19.51 % was achieved with this sample. (2nd measurement)

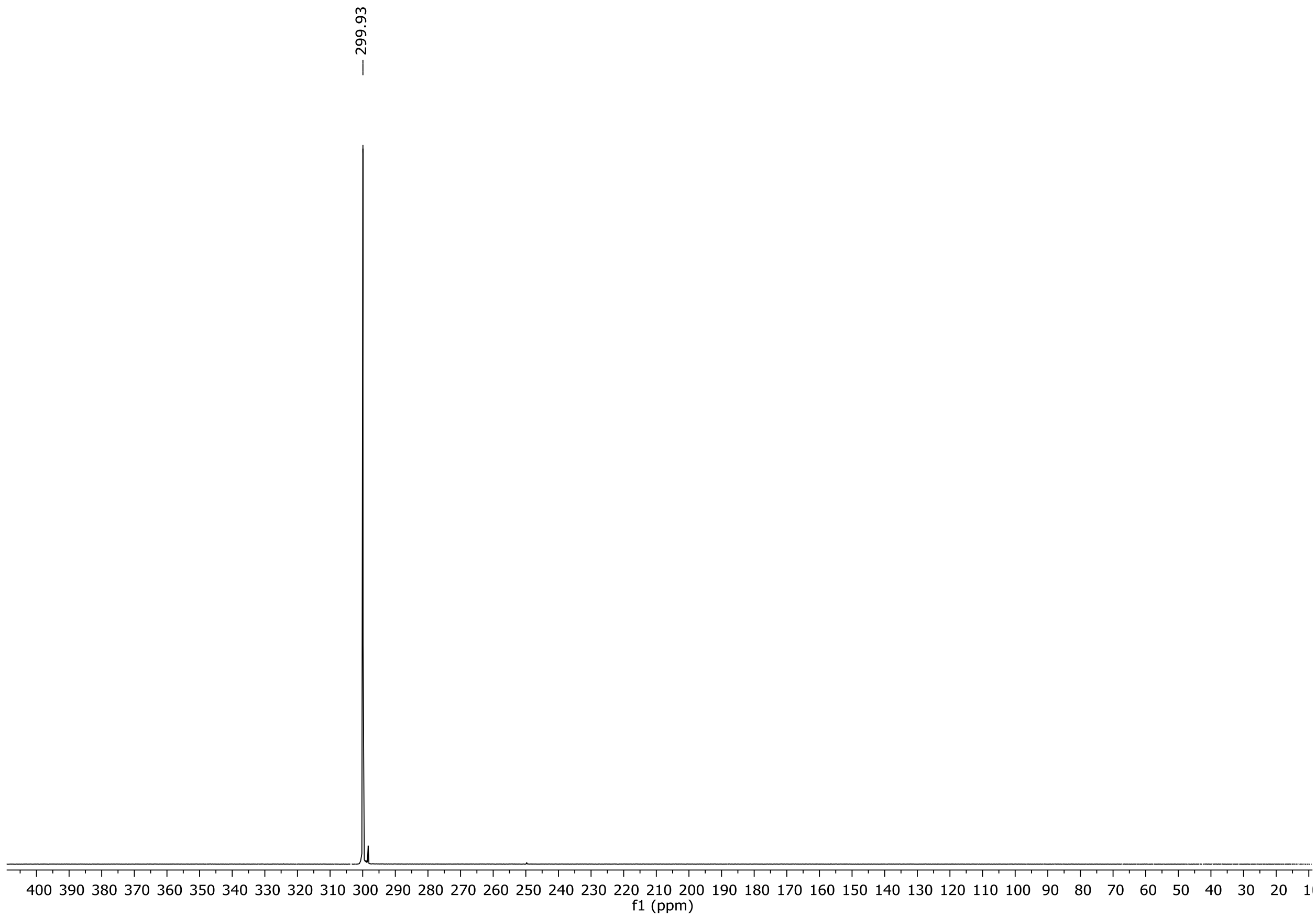

Object	Integral [abs]	Integral [rel]	Range (F1) from	Range (F1) to	ν(F1) [ppm]
Integral 1	2335323.361	0.999999901	300.6235205	299.298544	299.9610323

Figure S32. ^{15}N-hyperpolarization of pyridine-N-oxide **8** (2.5 mM) and the corresponding integrations. A hyperpolarization efficiency of 16.41 % was achieved with this sample. (3rd measurement)

Object	Effi (%)1	Effi (%)2	Effi (%)3	AVR Effi (%)
Integral 1	17.3	19.5	16.4	18±2

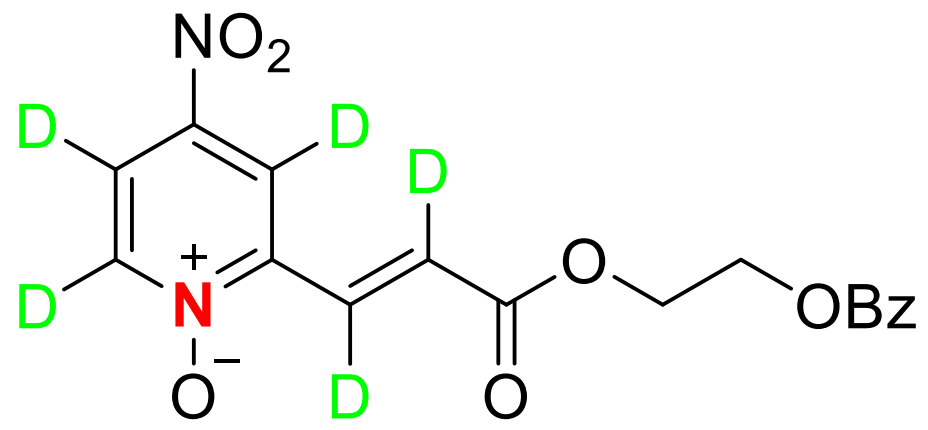

8

Hyperpolarization conditions: **8** / [Rh(dppb)(COD)][BF_4] 10:1, **Acetone** (0.2 mL). pH_2 bubbling time = 12 s, 330 K, J_{HH}= 7.1 Hz, J_{NH}=3.75 Hz.

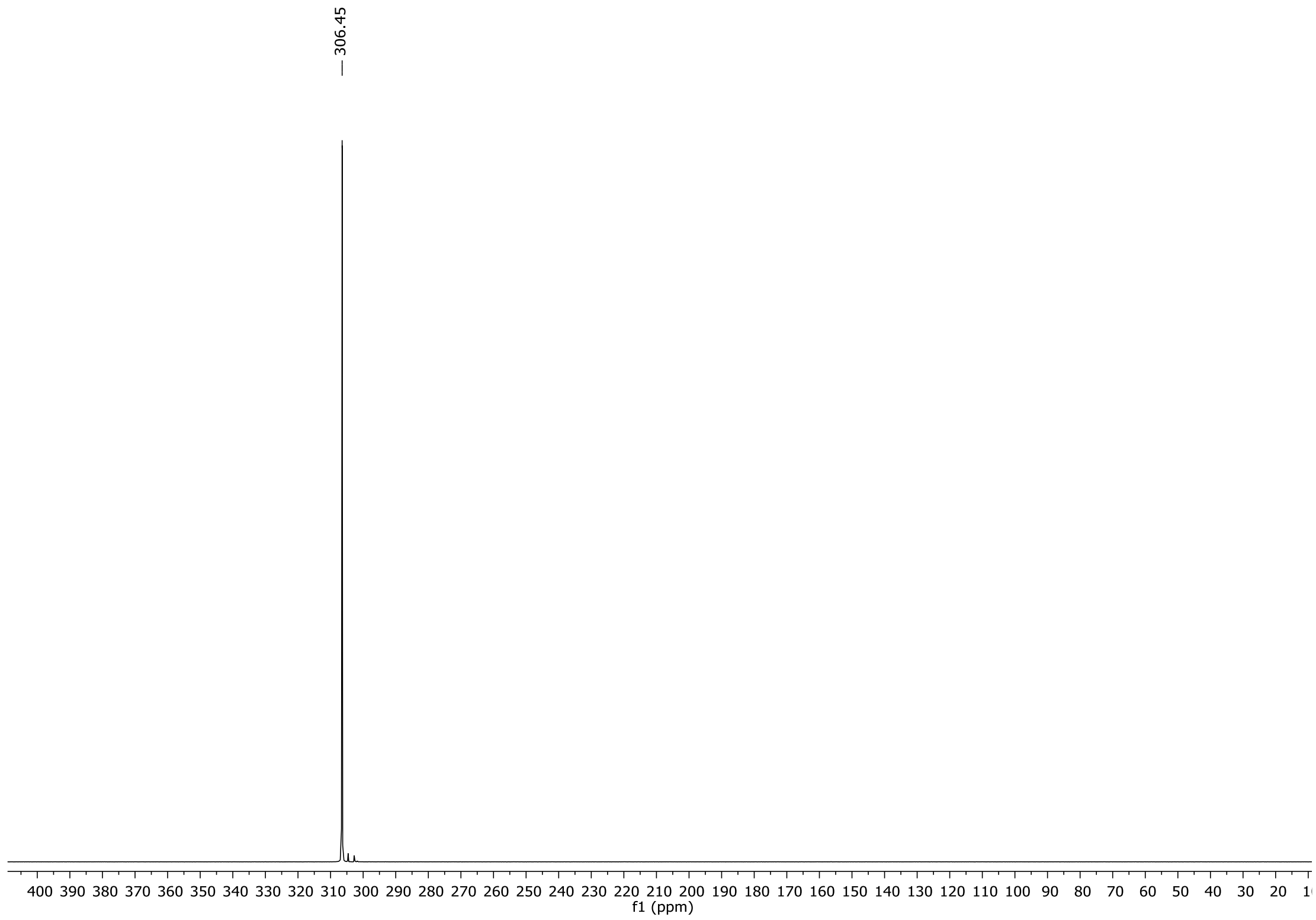

Object	Integral [abs]	Integral [rel]	Range (F1) from	Range (F1) to	ν(F1) [ppm]
Integral 1	5168453.598	1.00000001	307.1619219	305.6665583	306.4142401

Figure S33. ^{15}N-hyperpolarization of pyridine-N-oxide **8** (5.0 mM) and the corresponding integrations. A hyperpolarization efficiency of 18.15 % was achieved with this sample. (1st measurement)

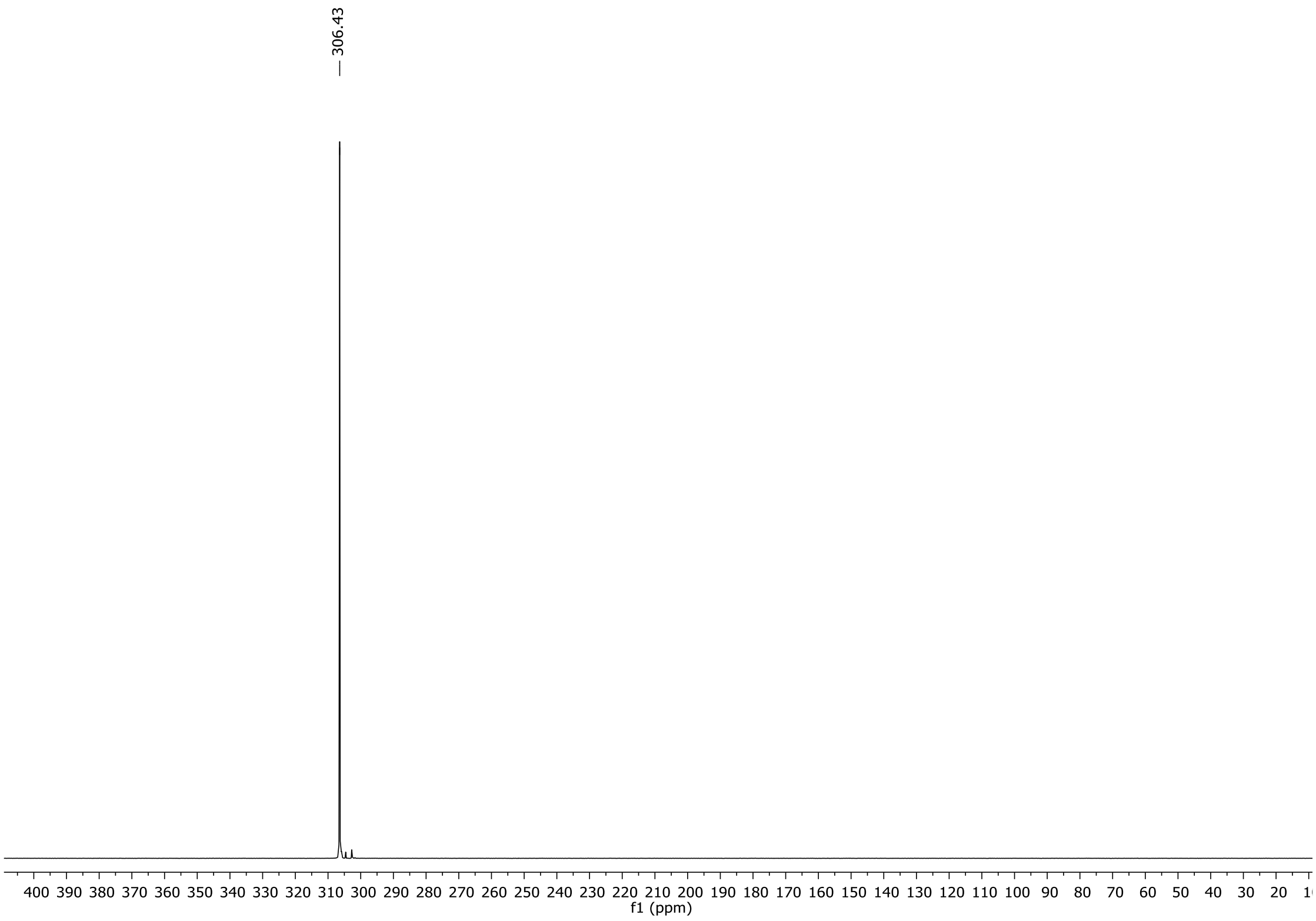

Object	Integral [abs]	Integral [rel]	Range (F1) from	Range (F1) to	ν(F1) [ppm]
Integral 1	3074875.524	0.999999821	308.6375632	305.1936258	306.9155945

Figure S34. ^{15}N-hyperpolarization of pyridine-N-oxide **8** (2.5 mM) and the corresponding integrations. A hyperpolarization efficiency of 21.60 % was achieved with this sample. (2nd measurement)

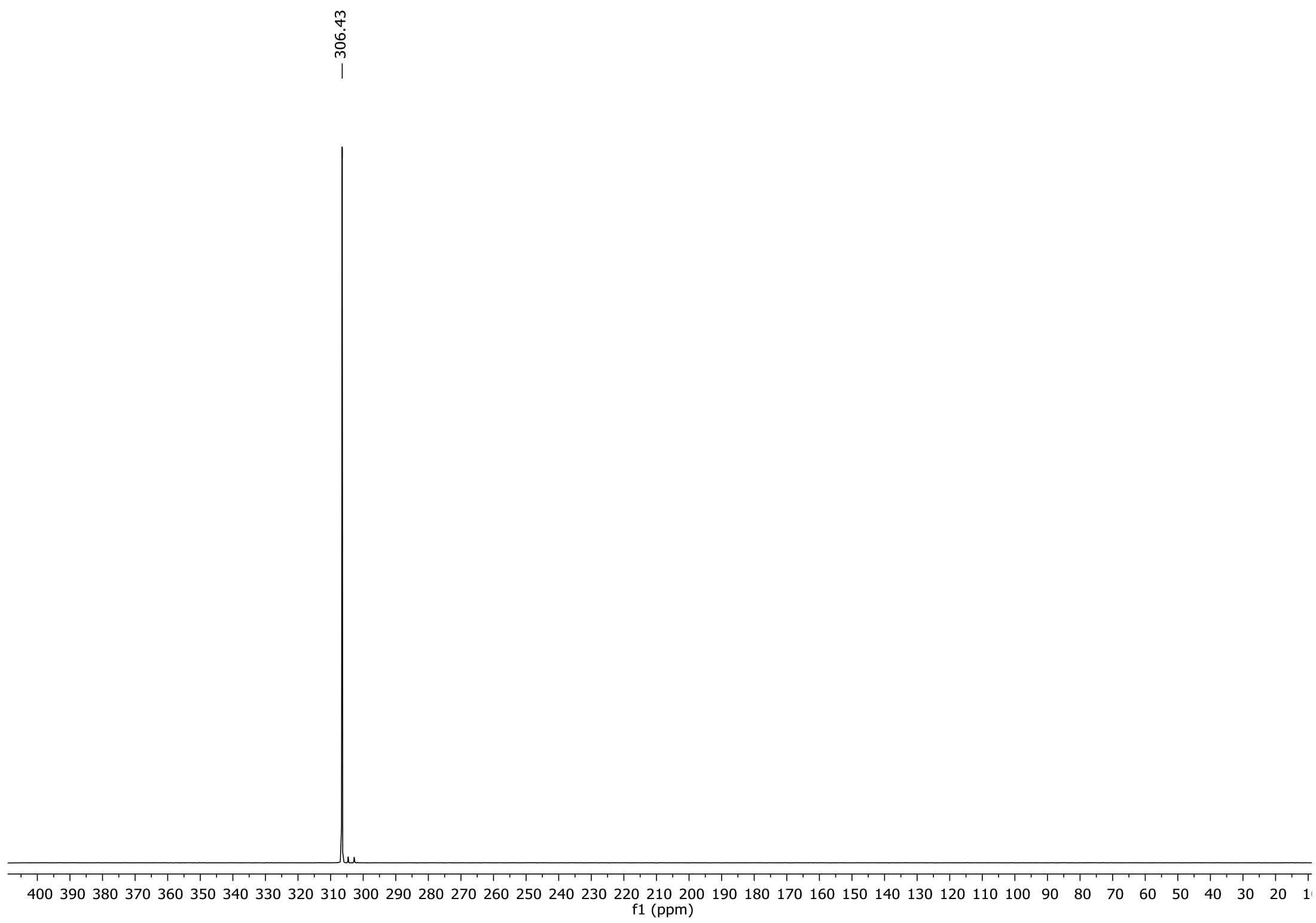

Object	Integral [abs]	Integral [rel]	Range (F1) from	Range (F1) to	ν(F1) [ppm]
Integral 1	3156021.231	1.000000102	308.0201626	305.1791555	306.5996591

Figure S35. ^{15}N-hyperpolarization of pyridine-N-oxide **8** (2.5 mM) and the corresponding integrations. A hyperpolarization efficiency of 22.17 % was achieved with this sample. (3rd measurement)

Object	Effi (%)1	Effi (%)2	Effi (%)3	AVR Effi (%)1
value	22.17	21.6	18.15	21±3

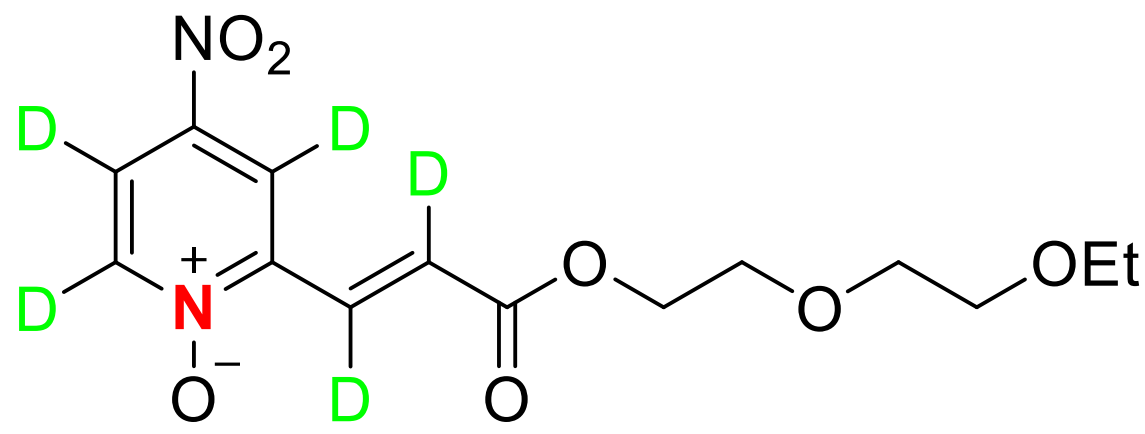

9

Hyperpolarization conditions: **9** / [Rh(dppb)(COD)][BF_4] 10:1, **MeOD-d4** (0.2 mL). pH_2 bubbling time = 9 s, 330 K, J_{HH}= 7.1 Hz, J_{NH}=3.75 Hz.

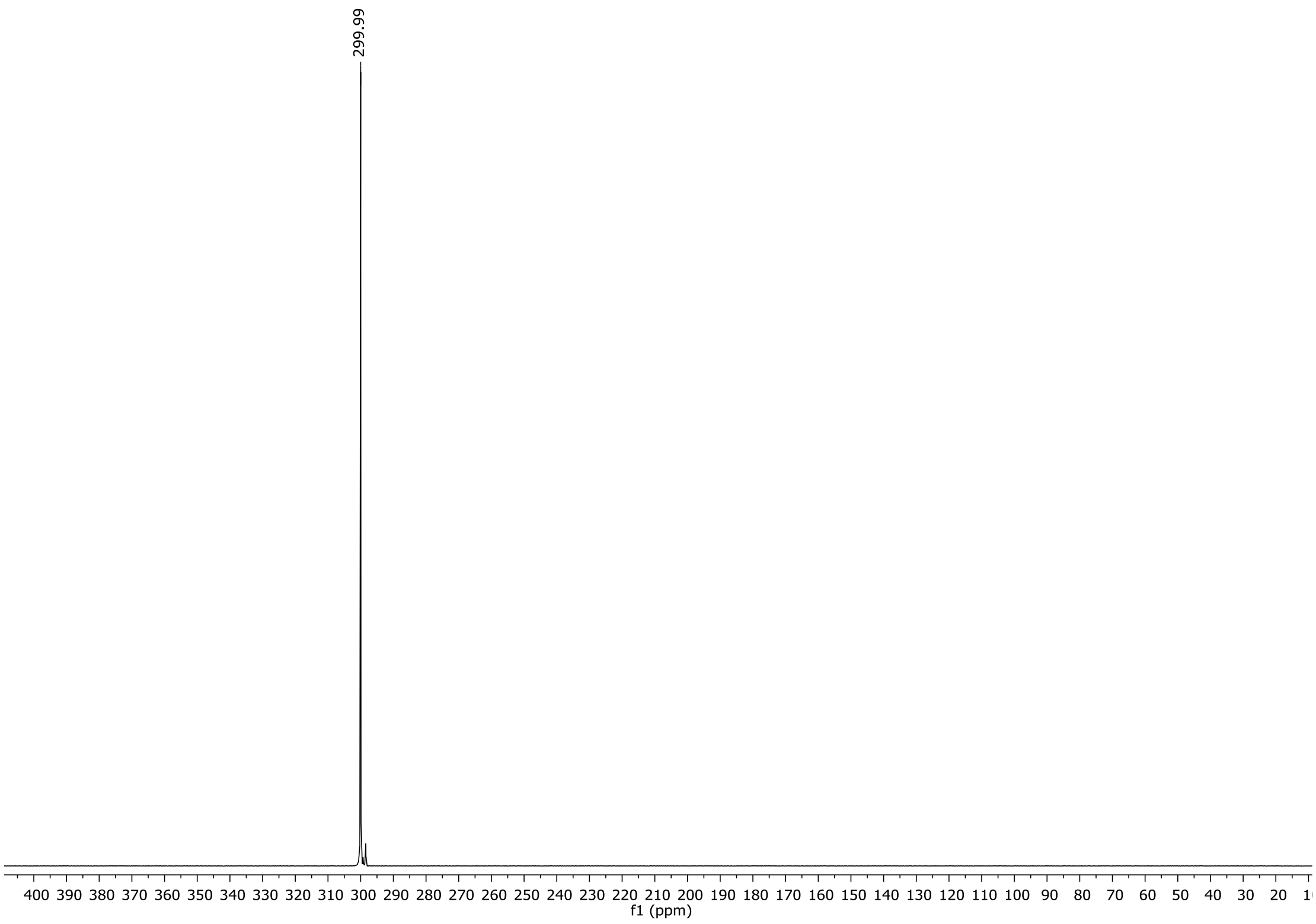

Object	Integral [abs]	Integral [rel]	Range (F1) from	Range (F1) to	ν(F1) [ppm]
Integral 1	2247718.747	1.000000044	300.8573858	299.5426596	300.2000227

Figure S36. ^{15}N-hyperpolarization of pyridine-N-oxide **9** (2.5 mM) and the corresponding integrations. A hyperpolarization efficiency of 15.8 % was achieved with this sample. (1st measurement)

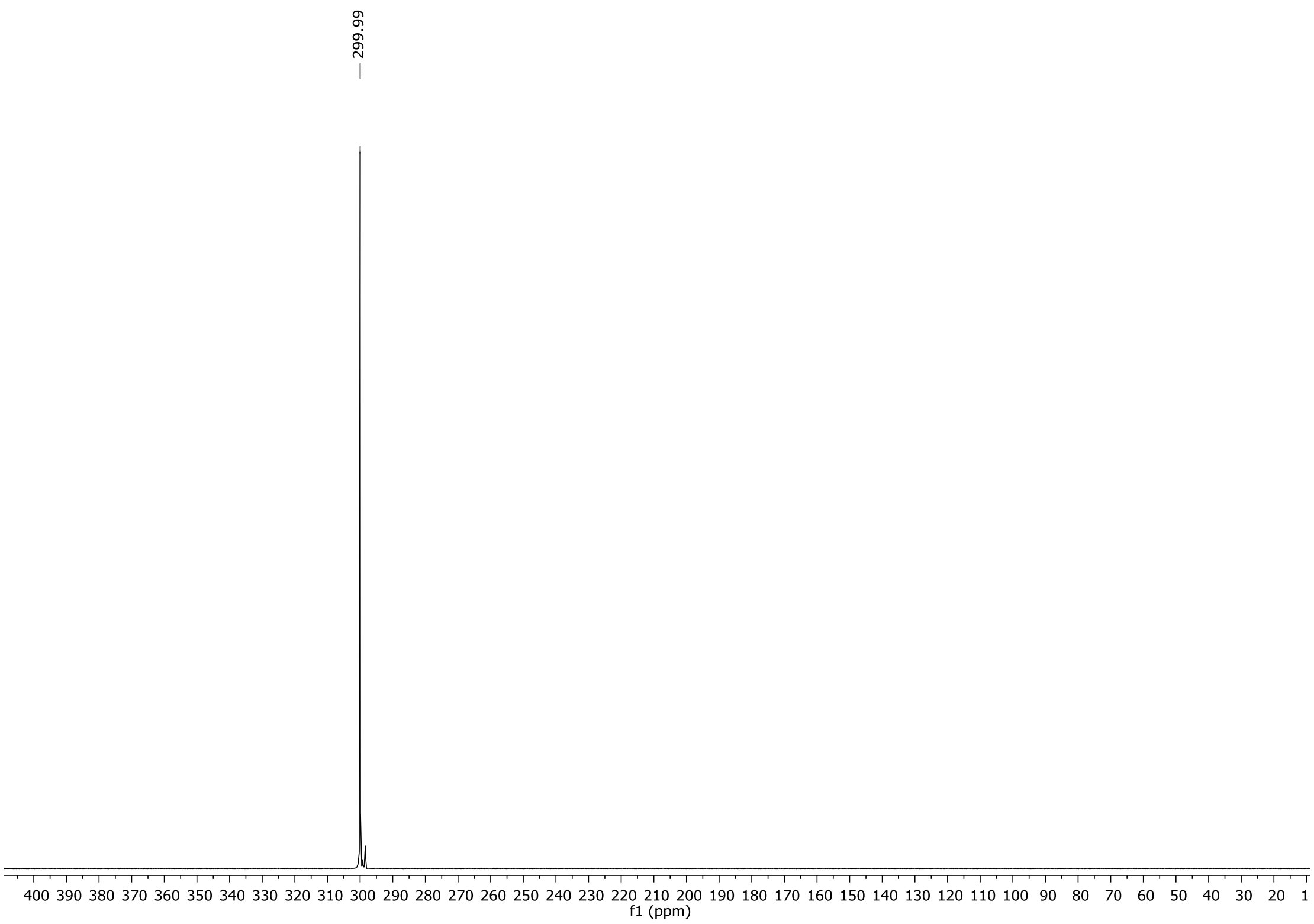

Object	Integral [abs]	Integral [rel]	Range (F1) from	Range (F1) to	ν(F1) [ppm]
Integral 1	2450577.599	1.000000025	300.7630704	299.4647313	300.1139009

Figure S37. ^{15}N-hyperpolarization of pyridine-N-oxide **9** (2.5 mM) and the corresponding integrations. A hyperpolarization efficiency of 17.2 % was achieved with this sample. (2nd measurement)

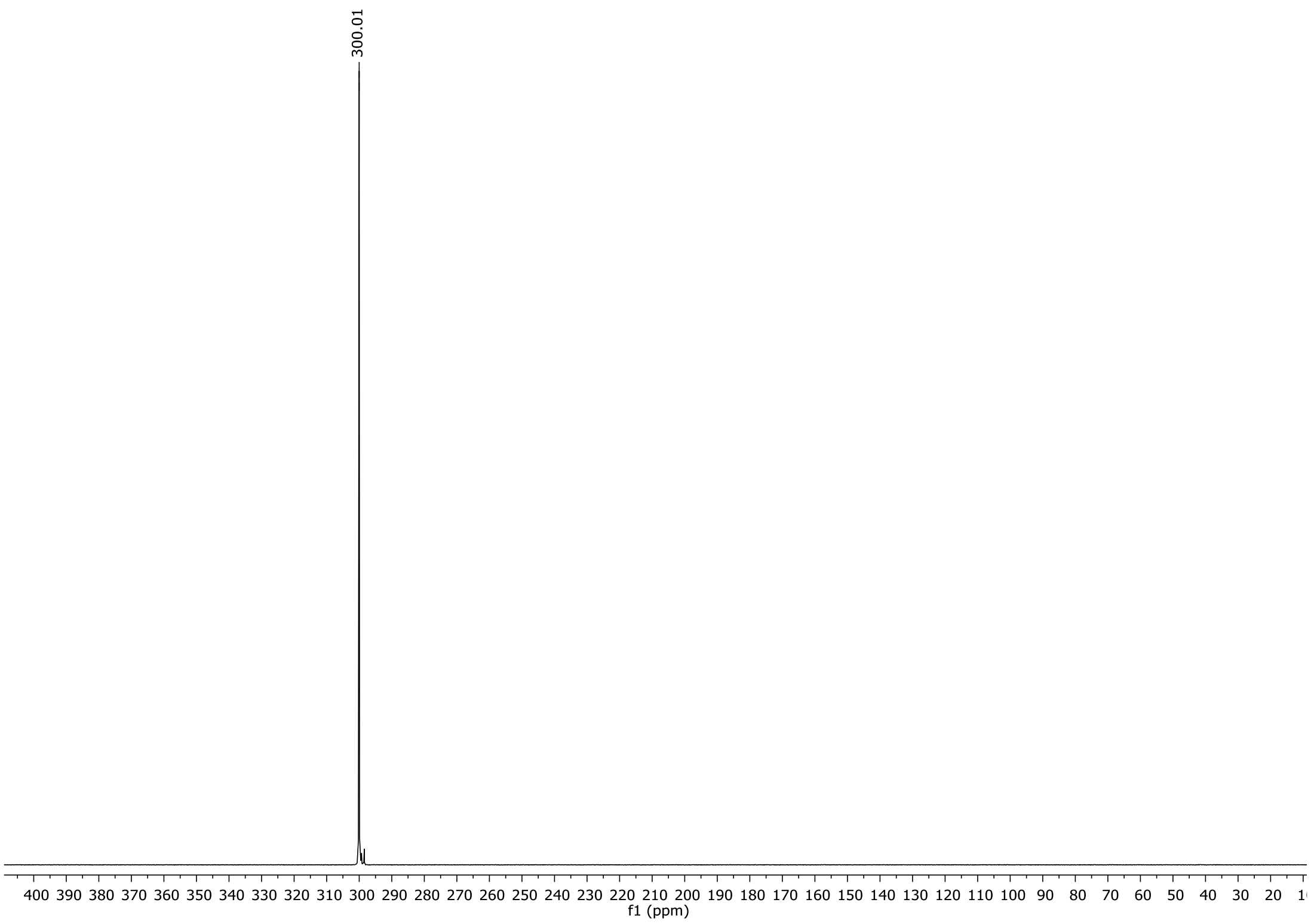

Object	Integral [abs]	Integral [rel]	Range (F1) from	Range (F1) to	ν(F1) [ppm]
Integral 1	1324744.008	1.000000102	300.7315702	299.4525201	300.0920451

Figure S38. ^{15}N-hyperpolarization of pyridine-N-oxide **9** (1.25 mM) and the corresponding integrations. A hyperpolarization efficiency of 18.6 % was achieved with this sample. (3rd measurement)

Object	Effi (%)1	Effi (%)2	Effi (%)3	AVR Effi (%)1
value	15.8	17.2	18.6	17±2

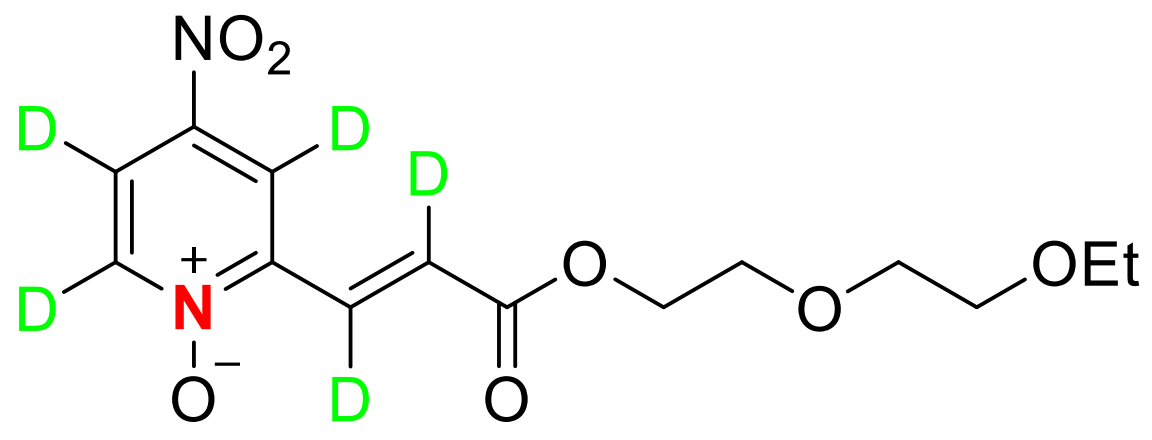

9

Hyperpolarization conditions: **9** / [Rh(dppb)(COD)][BF_4] 20:1, **Acetone** (0.2 mL). pH_2 bubbling time = 12 s, 330 K, J_{HH}= 7.1 Hz, J_{NH}=3.75 Hz.

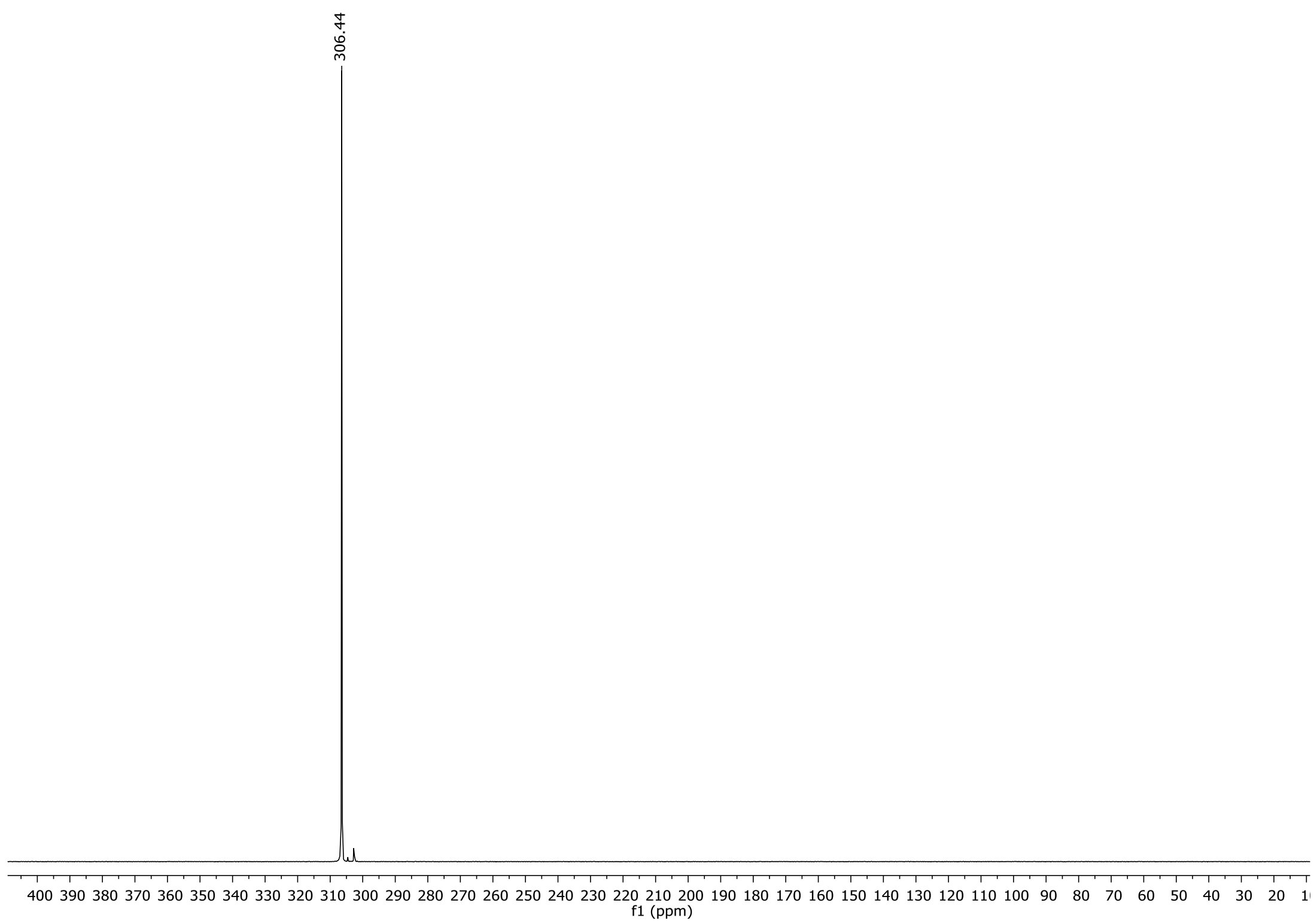

Object	Integral [abs]	Integral [rel]	Range (F1) from	Range (F1) to	ν(F1) [ppm]
Integral 1	2620035.589	1.000000226	307.4521269	305.7000695	306.5760982

Figure S39. ^{15}N-hyperpolarization of pyridine-N-oxide **9** (2.5 mM) and the corresponding integrations. A hyperpolarization efficiency of 18.41 % was achieved with this sample. (1st measurement)

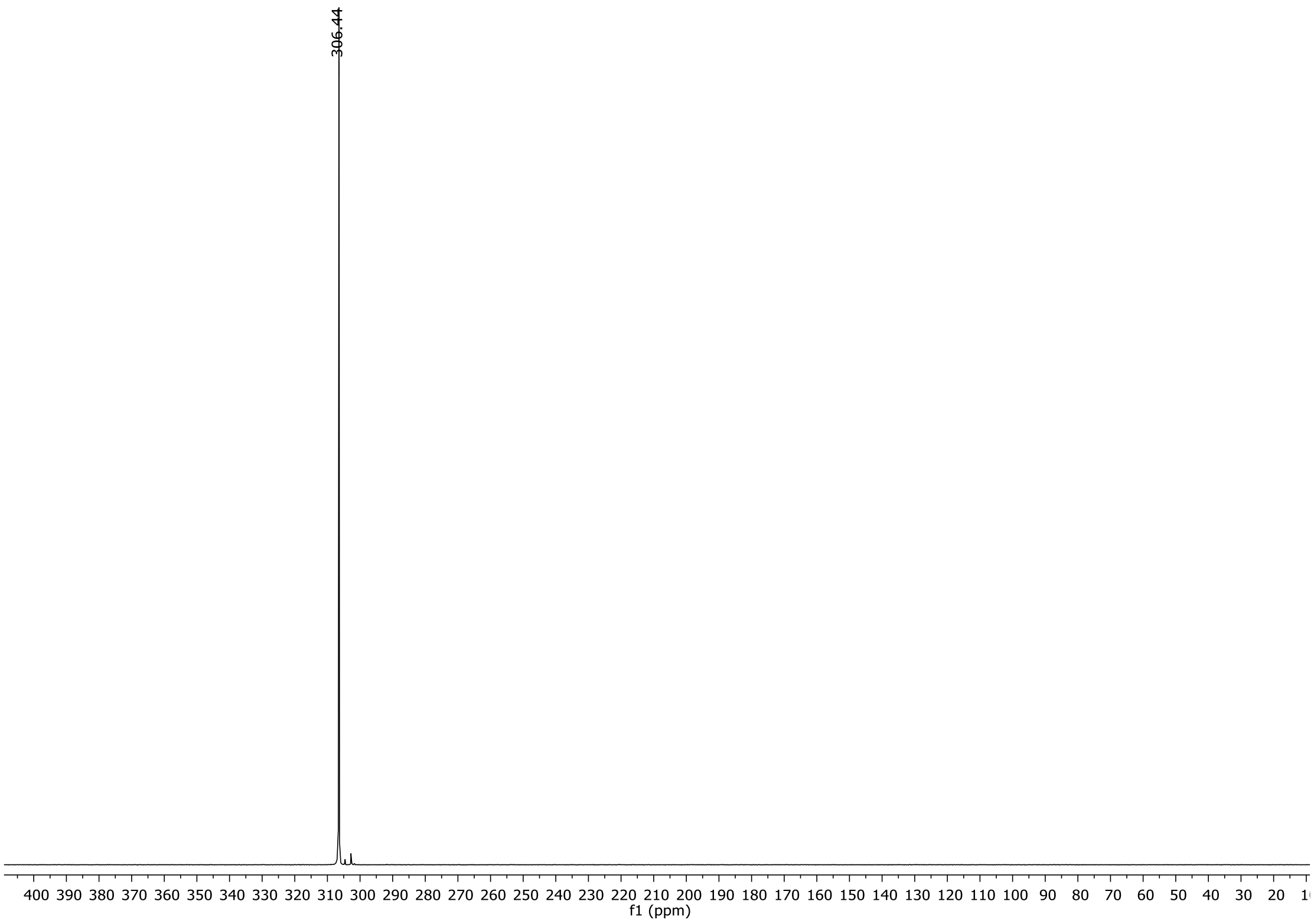

Object	Integral [abs]	Integral [rel]	Range (F1) from	Range (F1) to	ν(F1) [ppm]
Integral 1	2038554.553	1.000000131	307.5560041	305.6856408	306.6208224

Figure S40. ^{15}N-hyperpolarization of pyridine-N-oxide **9** (2.5 mM) and the corresponding integrations. A hyperpolarization efficiency of 14.32 % was achieved with this sample. (2nd measurement)

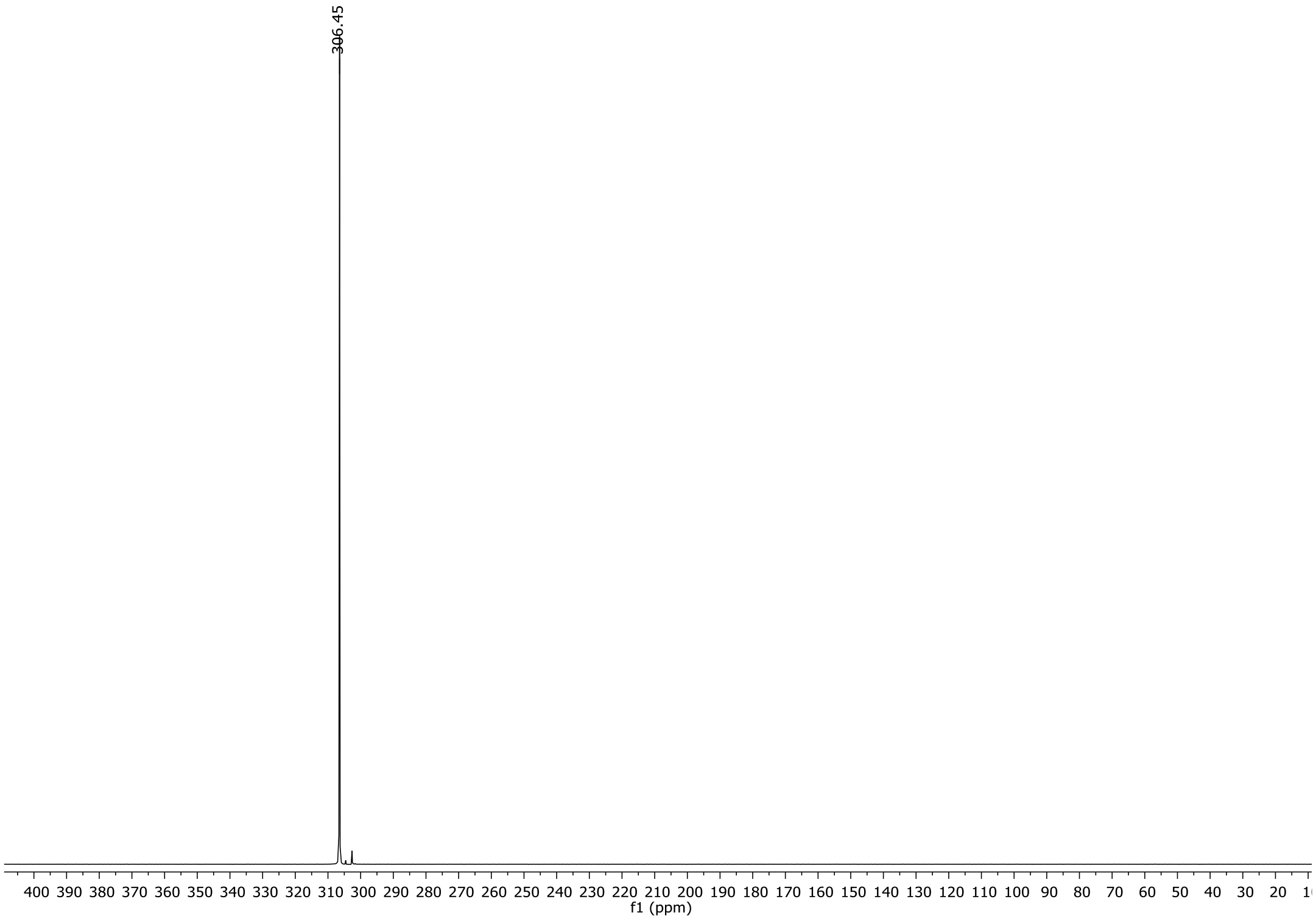

Object	Integral [abs]	Integral [rel]	Range (F1) from	Range (F1) to	ν(F1) [ppm]
Integral 1	5378803.508	0.99999988	307.4046325	305.6455927	306.5251126

Figure S41. ^{15}N-hyperpolarization of pyridine-N-oxide **9** (5.0 mM) and the corresponding integrations. A hyperpolarization efficiency of 18.89 % was achieved with this sample. (3rd measurement)

Object	Effi (%)1	Effi (%)2	Effi (%)3	AVR Effi (%)
value	15.8	17.2	18.9	17±3

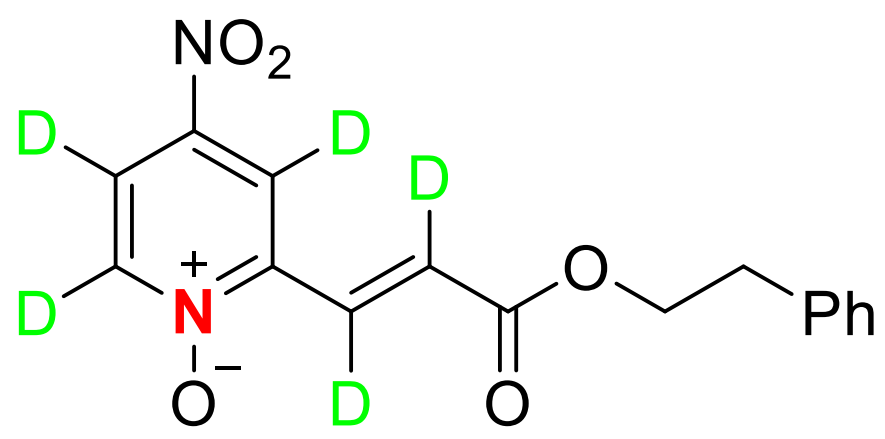

10

Hyperpolarization conditions: **10** / [Rh(dppb)(COD)][BF_4] 20:1, **Acetone-d$_6$** (0.2 mL). pH_2 bubbling time = 12 s, 330 K, J_{HH}= 7.1 Hz, J_{NH}=3.75 Hz.

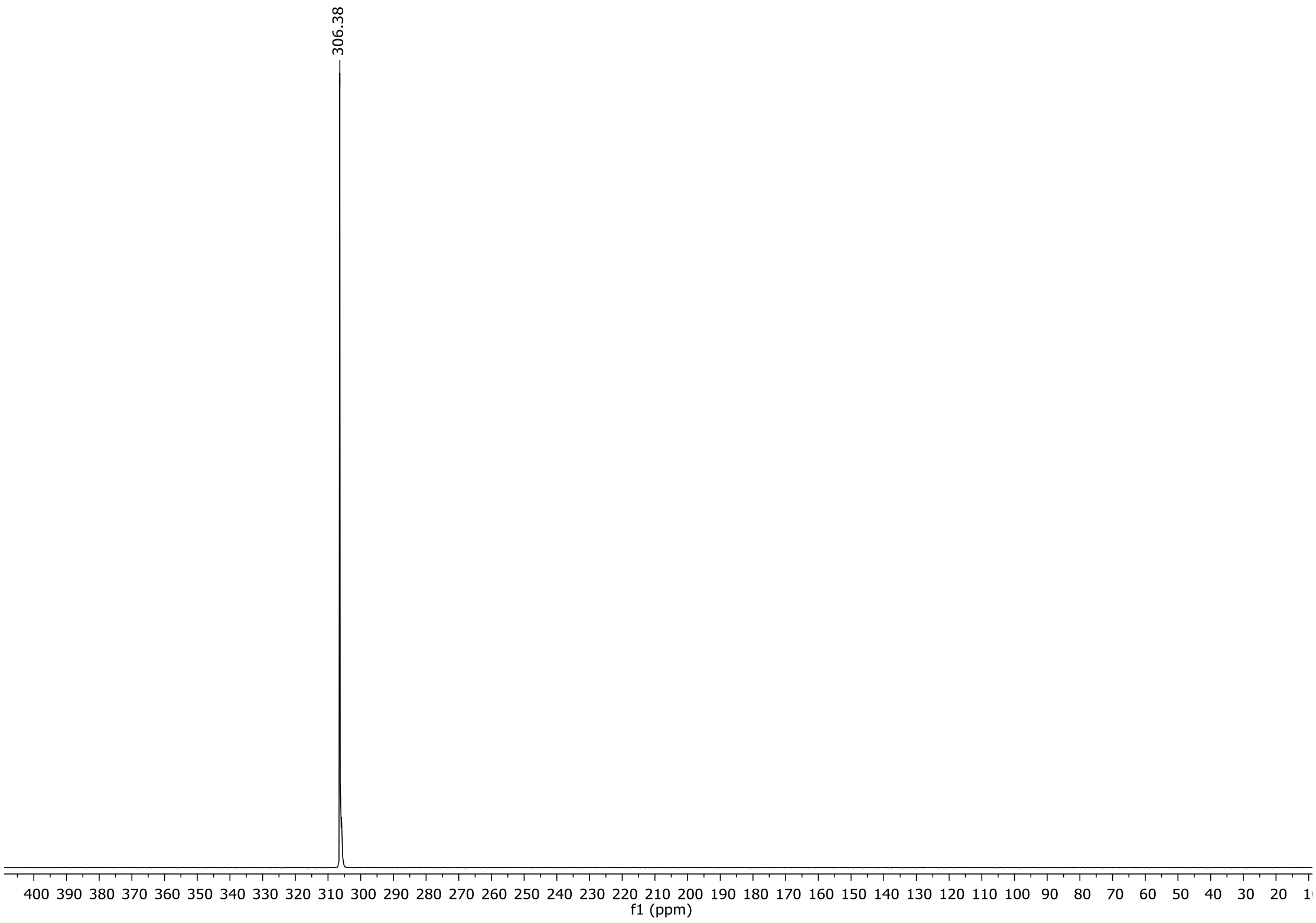

Object	Integral [abs]	Integral [rel]	Range (F1) from	Range (F1) to	ν(F1) [ppm]
Integral 1	1824856.58	1.00000001	306.9260304	305.0133876	305.969709

Figure S42. ^{15}N-hyperpolarization of pyridine-N-oxide **10** (5.0 mM) and the corresponding integrations. A hyperpolarization efficiency of 12.82 % was achieved with this sample. (1st measurement).

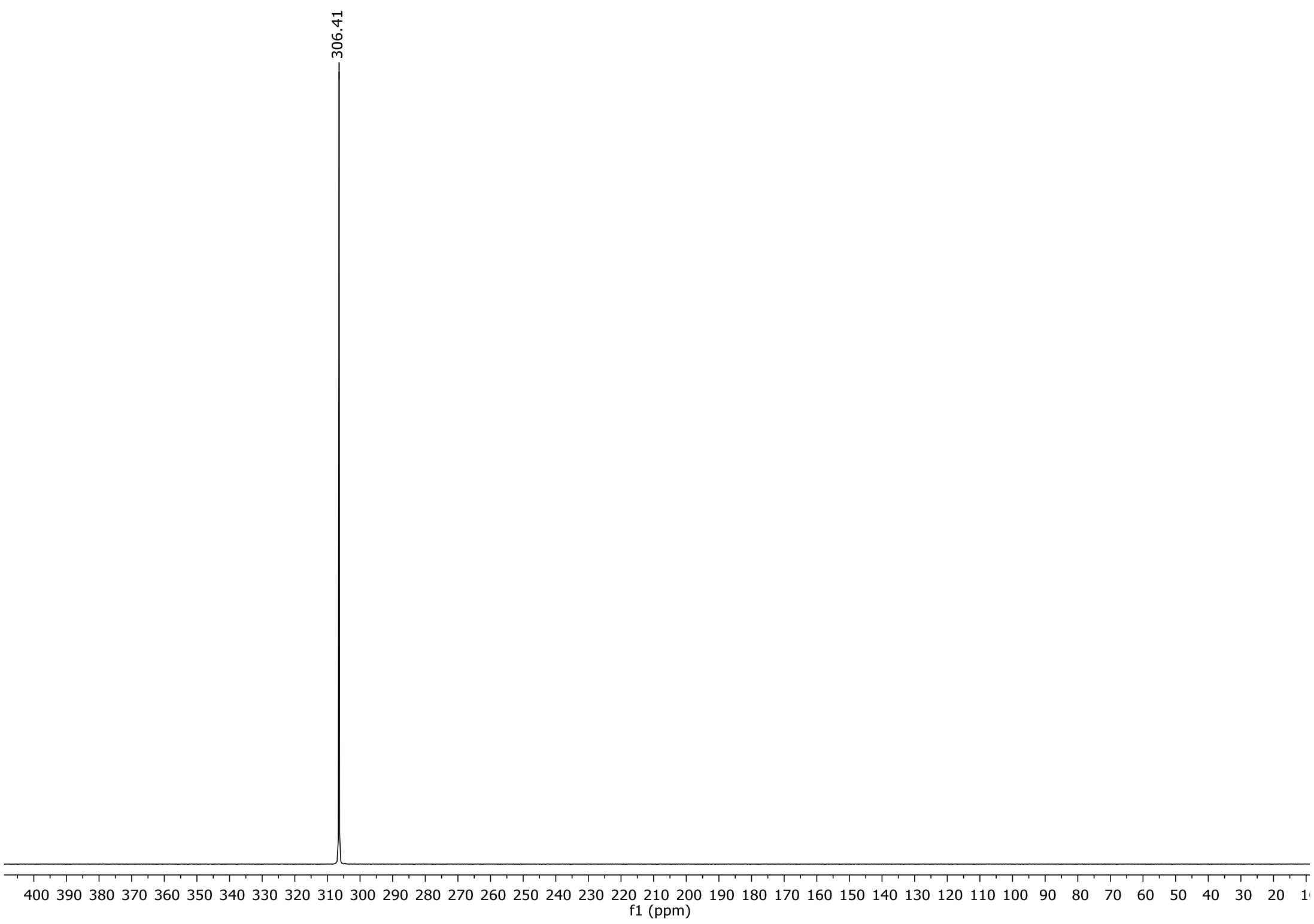

Object	Integral [abs]	Integral [rel]	Range (F1) from	Range (F1) to	ν(F1) [ppm]
Integral 1	2024305.295	1.000000027	307.2896192	305.5027783	306.3961988

Figure S43. ^{15}N-hyperpolarization of pyridine-N-oxide **10** (5.0 mM) and the corresponding integrations. A hyperpolarization efficiency of 14.2 % was achieved with this sample. (2nd measurement).

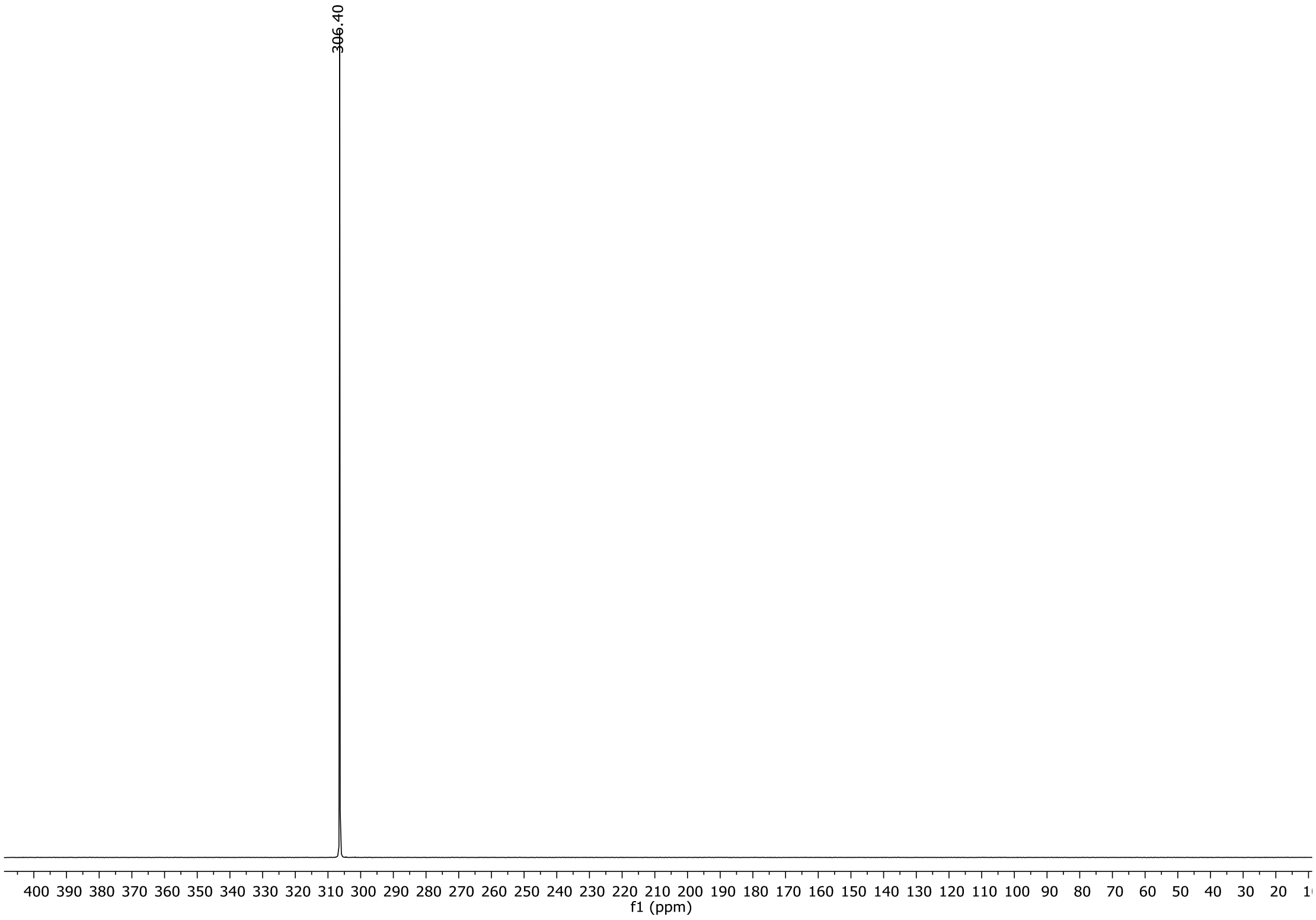

Object	Integral [abs]	Integral [rel]	Range (F1) from	Range (F1) to	ν(F1) [ppm]
Integral 1	2393676.346	1.000000001	307.0858567	305.5968225	306.3413396

Figure S44 ^{15}N-hyperpolarization of pyridine-N-oxide **10** (5.0 mM) and the corresponding integrations. A hyperpolarization efficiency of 16.82 % was achieved with this sample. (3rd measurement).

Object	Effi (%)1	Effi (%)2	Effi (%)3	AVR Effi (%)
value	12.8	14.2	16.8	15±2

2.7 T_1 field dependence for pyridine ^{15}N-oxide derivatives

These T_1 data were obtained by measuring with low flip angle (10 °) pulses at 335 K in the indicated solvents and magnetic field.

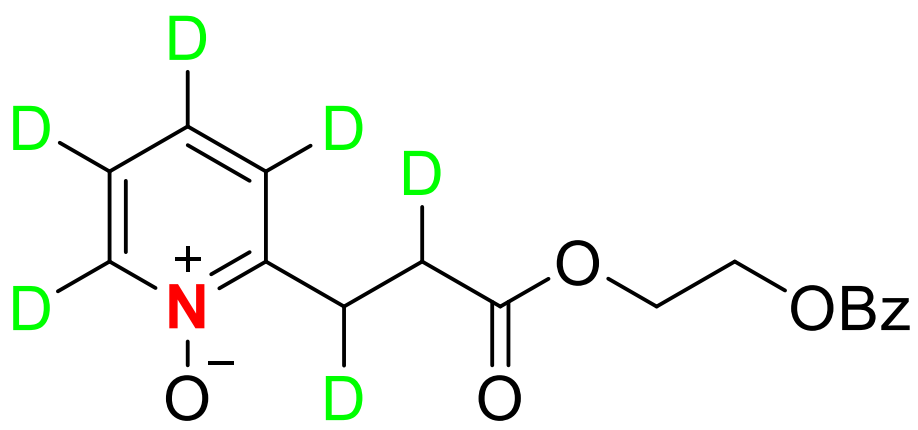

1'

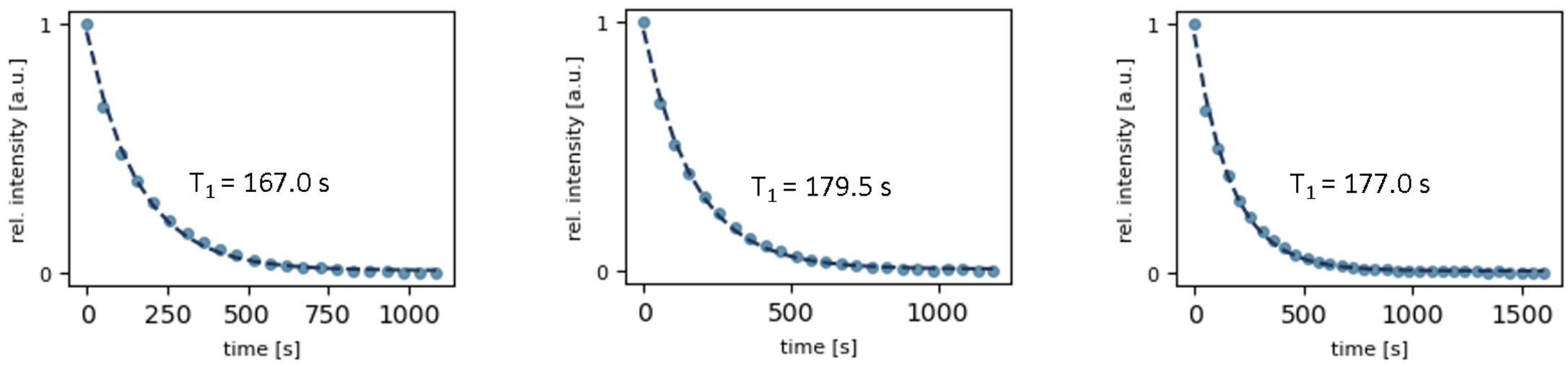

T_1 (s)	T_1 (s)-1st	T_1 (s)-2nd	T_1 (s)-3rd	AVR T_1 (%)
value	167.0	179.5	177.0	175±7

Figure S45. T_1 @ 7.0 T in MeOD-d_4.

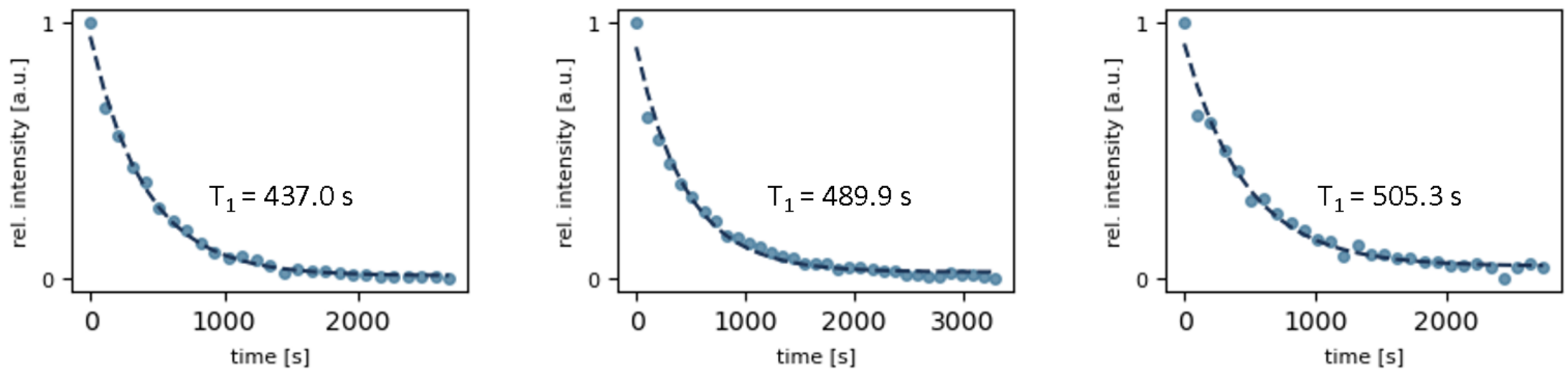

T_1 (s)	T_1 (s)-1st	T_1 (s)-2nd	T_1 (s)-3rd	AVR T_1 (s)
value	437.0	489.9	505.3	477±36

Figure S46. T_1 @ 0.1 T in MeOD-d_4.

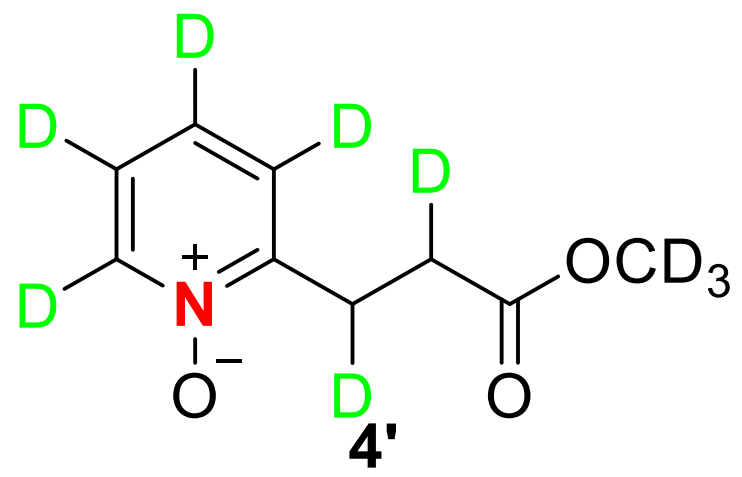

4'

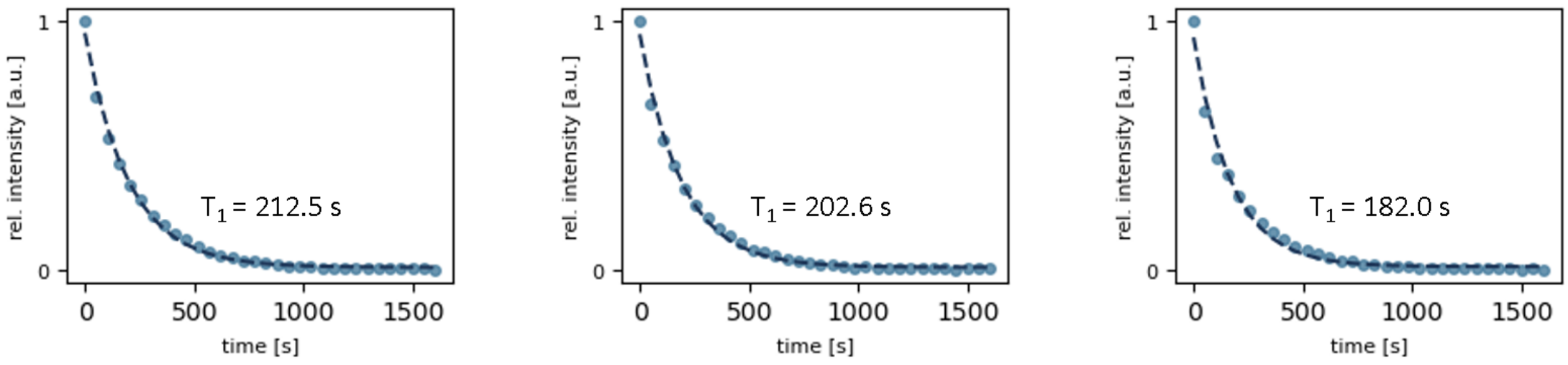

T_1 (s)	T_1 (s)-1st	T_1 (s)-2nd	T_1 (s)-3rd	AVR T_1 (s)
value	182.0	212.5	202.6	199±16

Figure S47. T_1 @ 7.0 T in MeOD-d4.

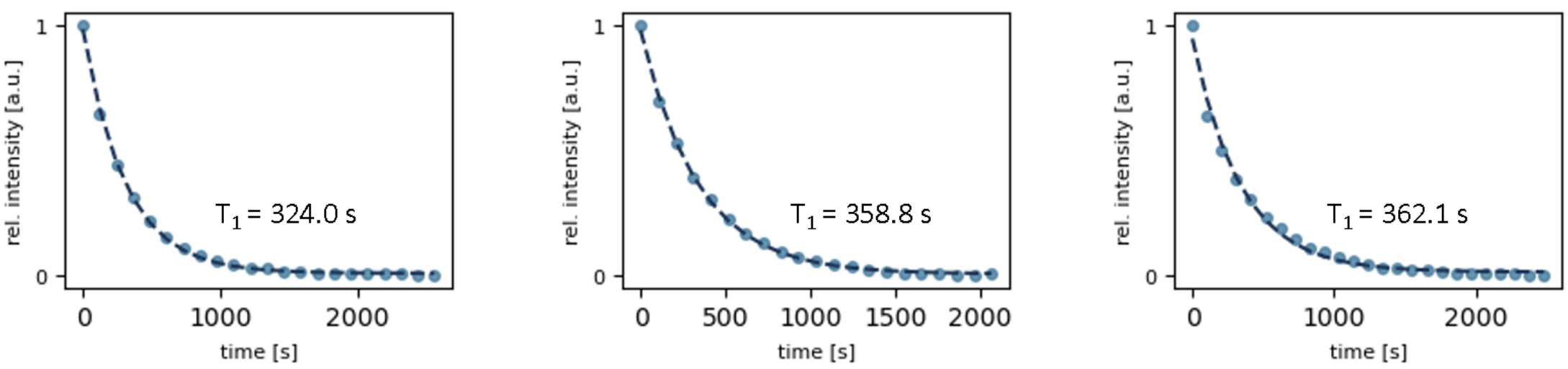

T_1 (s)	T_1 (s)-1st	T_1 (s)-2nd	T_1 (s)-3rd	AVR T_1 (s)
value	324.0	358.8	362.1	348±22

Figure S48. T_1 @ 0.1 T in MeOD-d4.

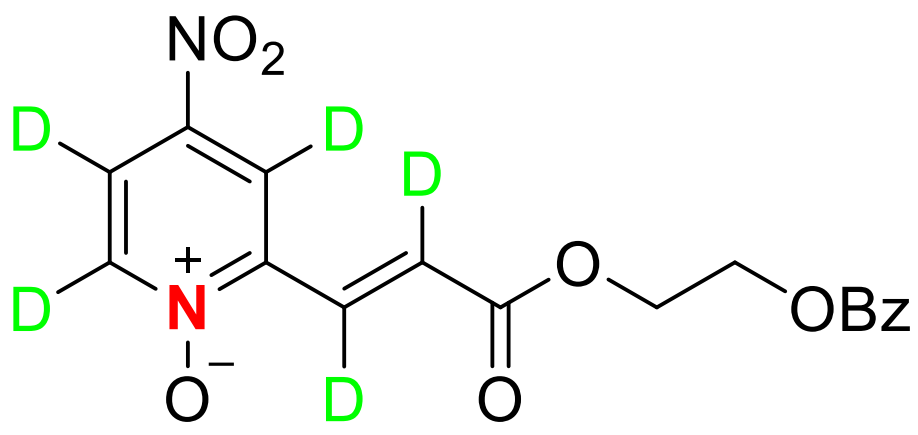

8'

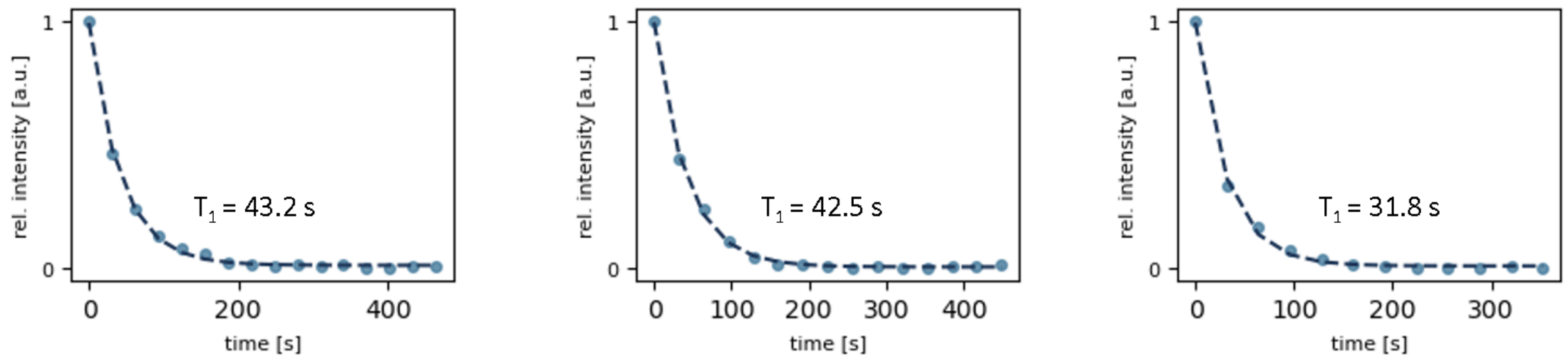

T_1 (s)	T_1 (s)-1st	T_1 (s)-2nd	T_1 (s)-3rd	AVR T_1 (s)
value	43.2	41.6	31.3	39±7

Figure S49. T_1 @ 7.0 T in MeOD-d_4

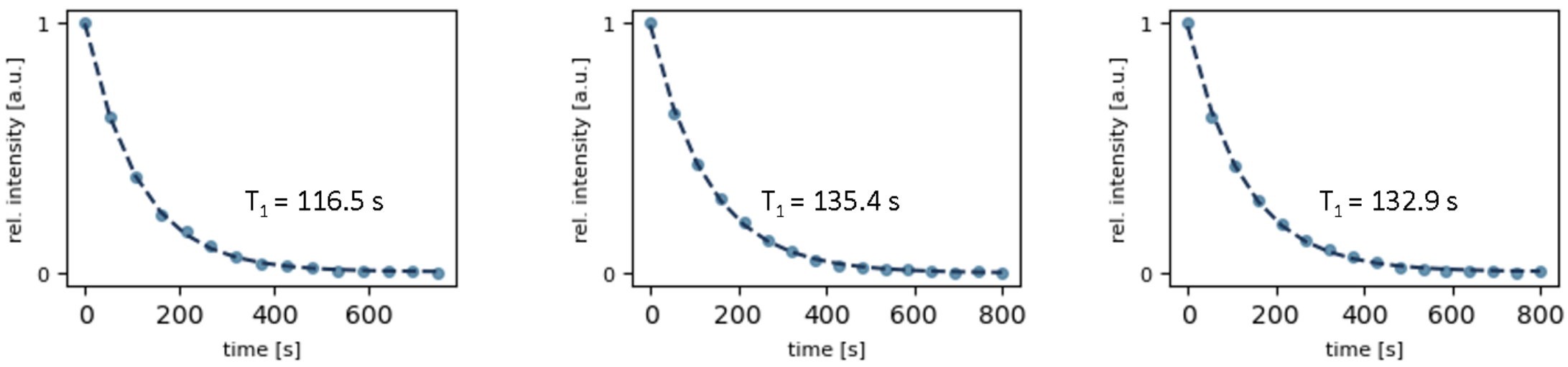

T_1 (s)	T_1 (s)-1st	T_1 (s)-2nd	T_1 (s)-3rd	AVR T_1 (s)
value	135.4	132.9	116.5	128±11

Figure S50. T_1 @ 7.0 T in Acetone-d_6.

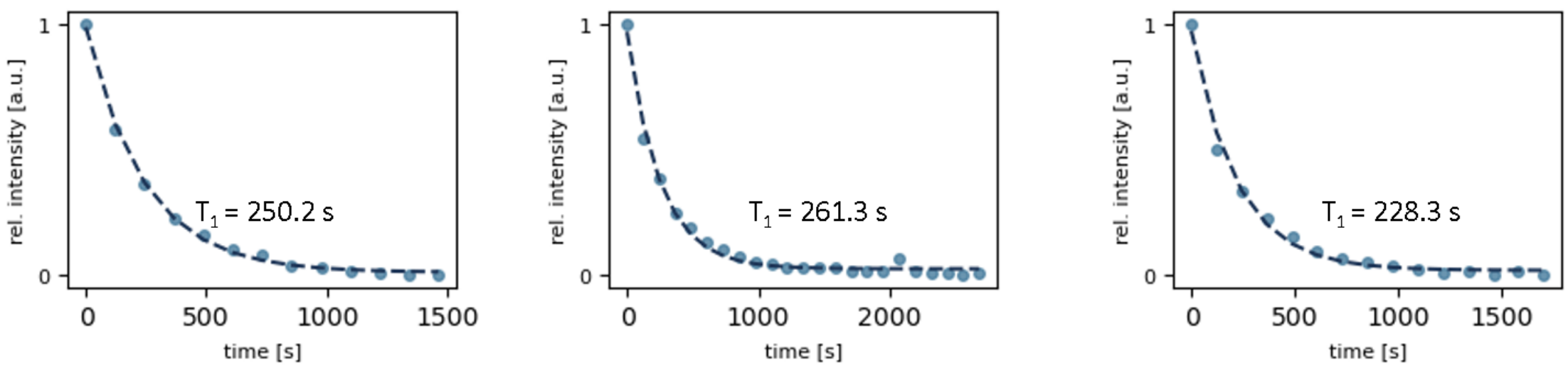

T_1 (s)	T_1 (s)-1st	T_1 (s)-2nd	T_1 (s)-3rd	AVR T_1 (s)
value	250.2	261.1	228.3	247±17

Figure S51. T_1 @ 0.1 T in Acetone-d_6.

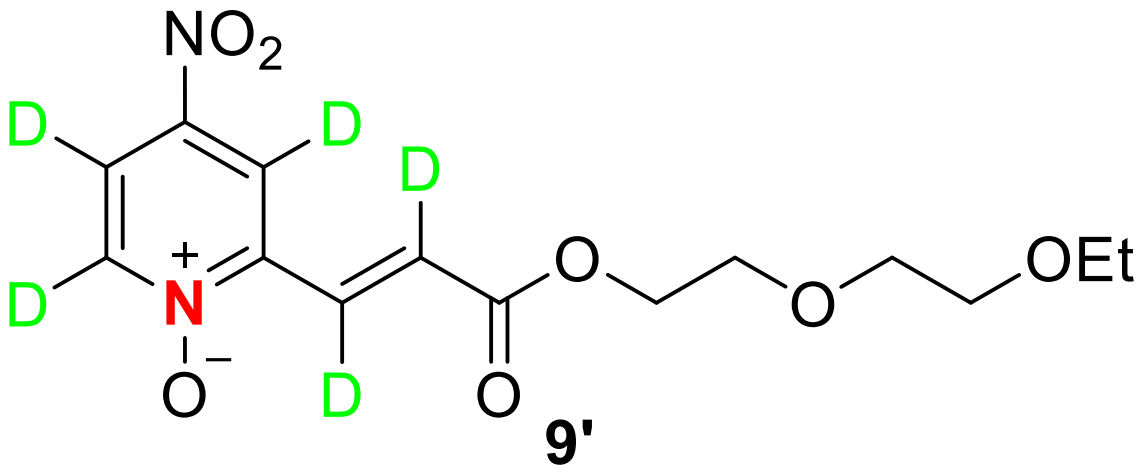

9'

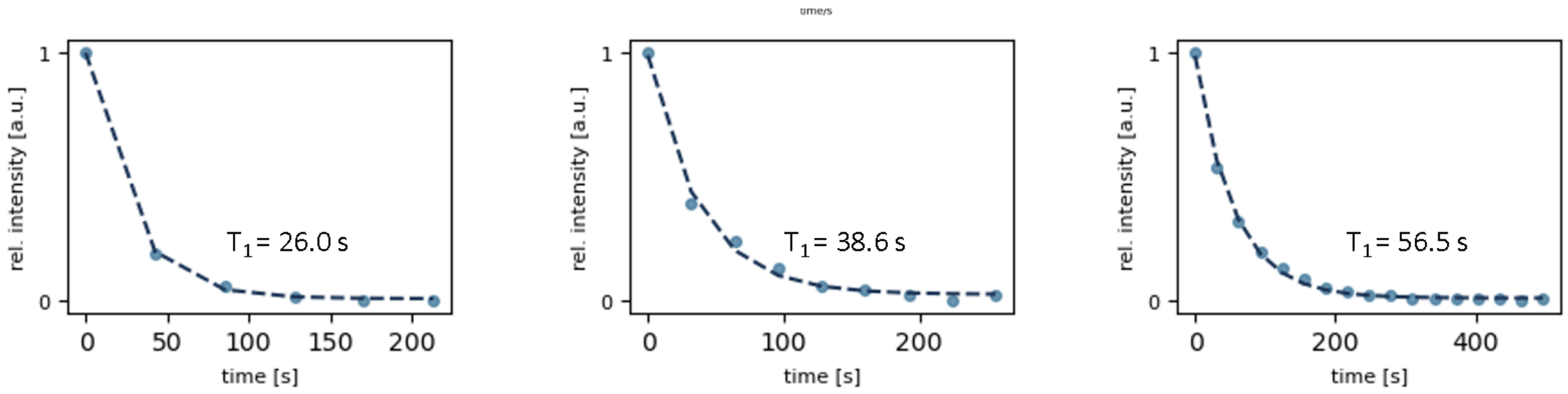

T₁ (s)	T₁ (s)-1st	T₁ (s)-2nd	T₁ (s)-3rd	AVR T₁ (%)
value	26.0	38.6	56.5	40±16

Figure S52. T_1 @ 7.0 T in MeOD-d_4.

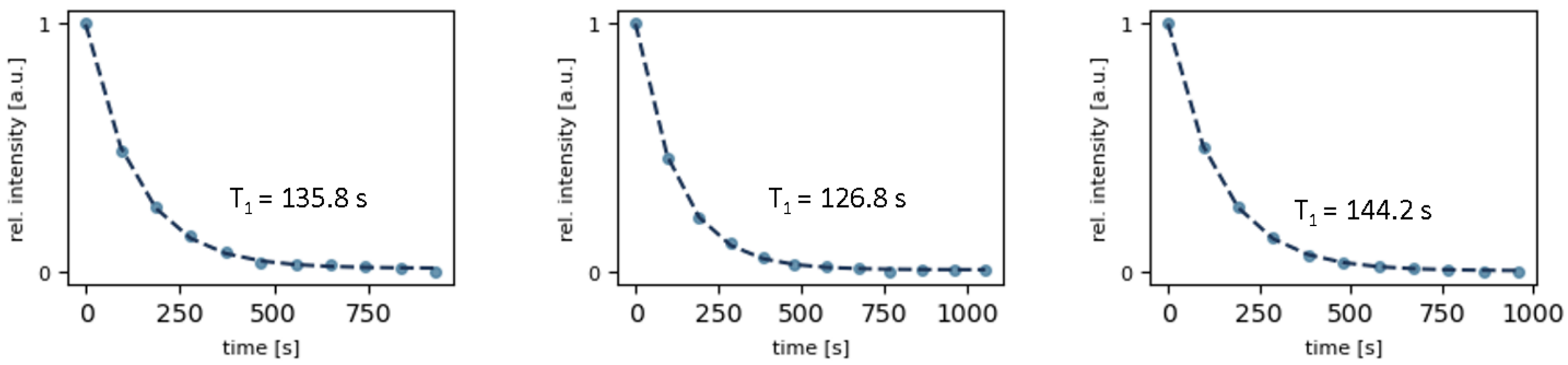

T₁ (s)	T₁ (s)-1st	T₁ (s)-2nd	T₁ (s)-3rd	AVR T₁ (s)
value	135.8	126.8	144.2	136±9

Figure S53. T_1 @ 7.0 T in Acetone-d_6.

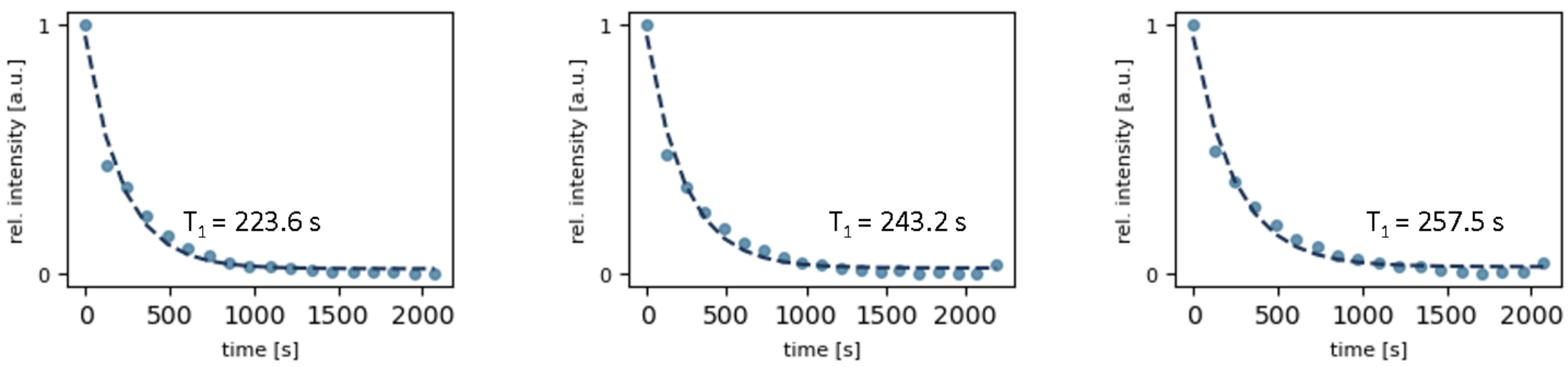

T₁ (s)	T₁ (s)-1st	T₁ (s)-2nd	T₁ (s)-3rd	AVR T₁ (s)
value	186.4	214.6	202.7	201±15

Figure S54. T_1 @ 0.1 T in Acetone-d_6.

2.8 Hyperpolarized amine 6' employed in pH measurement.

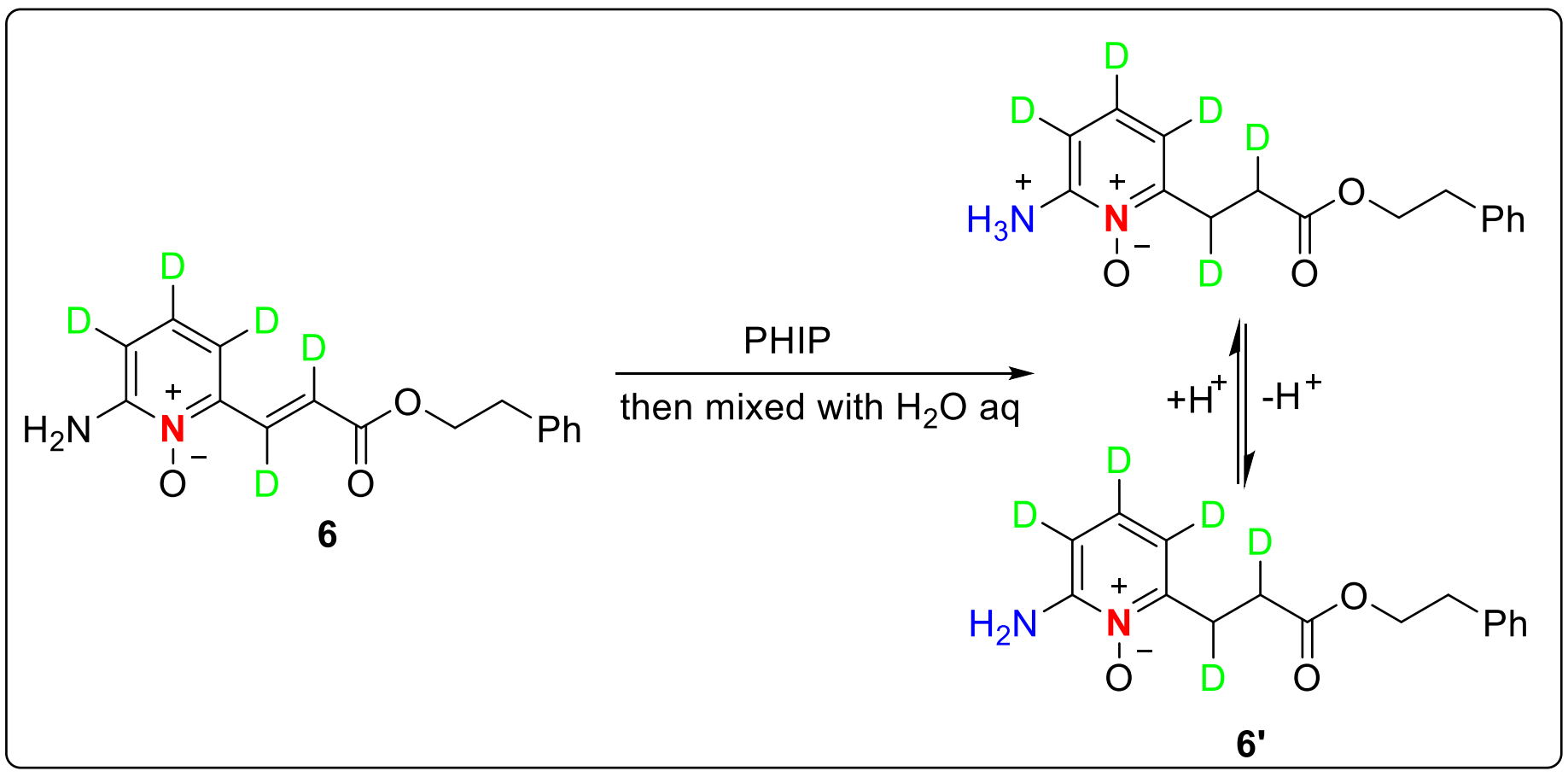

Procedure 1-(without remove of MeOD): A sample containing amine substituted ^{15}N-oxide **6** (1.875 mM), [Rh(dppb)(COD)][BF_4] (0.125 mM) in MeOD-d_4 (200 μL) was freshly prepared. A gentle N_2 flow was bubbling into the sample for 50s to remove oxygen, and the sample was equilibrated at 335 K for 2 min before the test. The MINERVA[5] sequence in Fig. 1 (2) of ref [5b] with a final 90° pulse was executed. After the polarization transfer, H_2O buffer with the indicated pH (100 μL) was added. 1D-Spectra were recorded 10s after injection of aqueous buffer using a 90° pulse (Figure S55a).

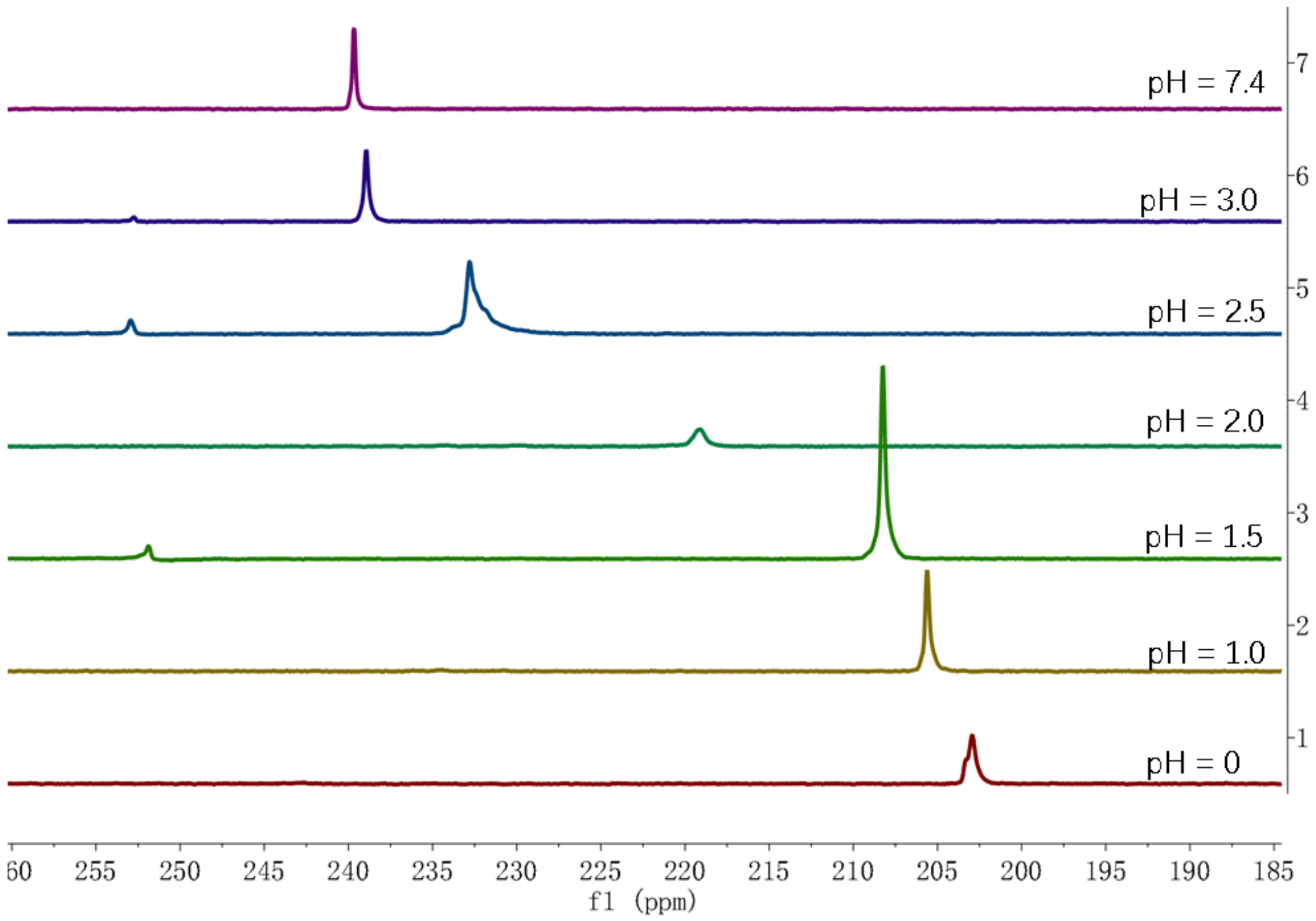

Figure S55a-1. ^{15}N chemical shift of HP-pyridine-N-oxide **6'** in various pH environments in MeOD/H_2O media.

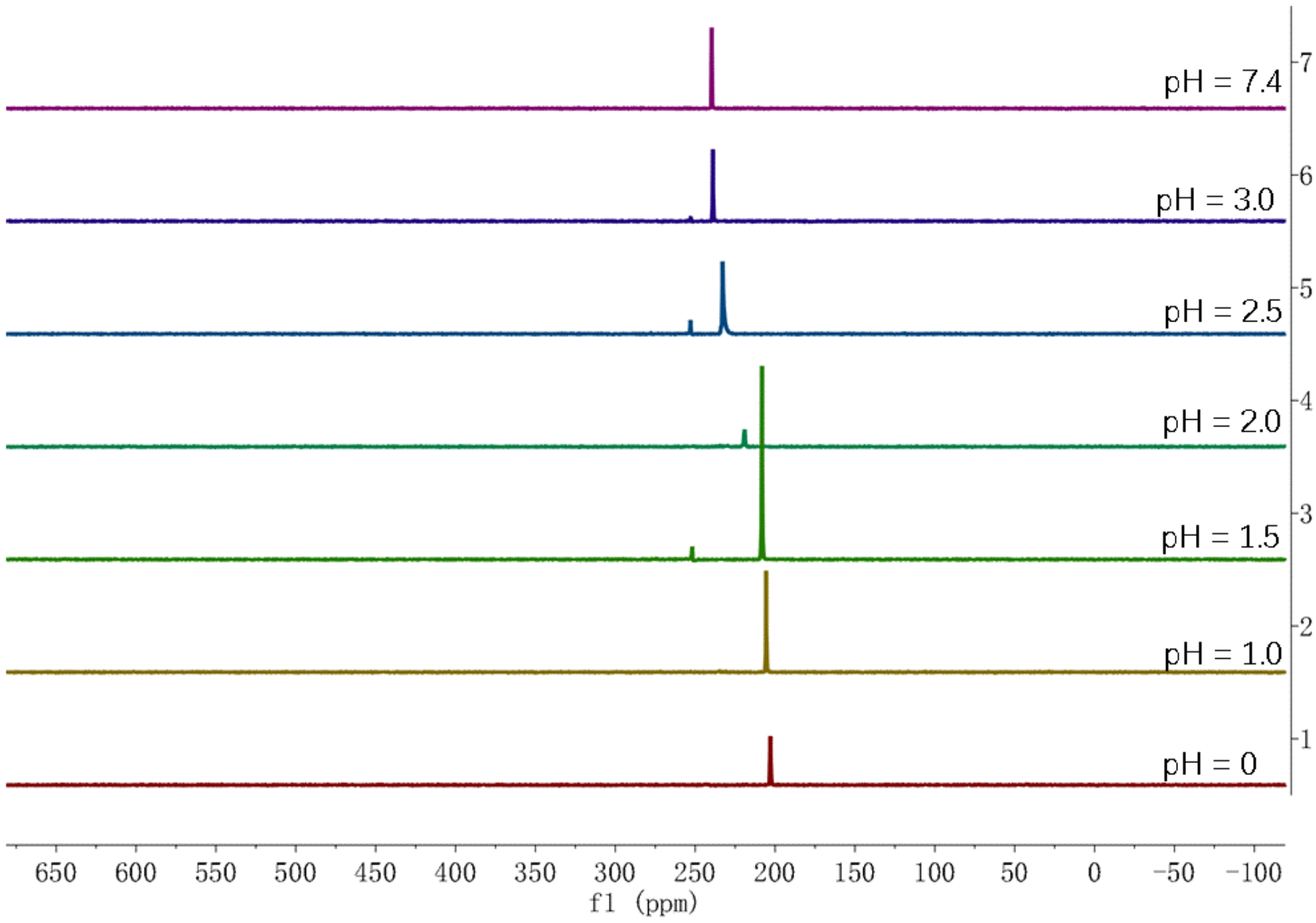

Figure S55a-2. ^{15}N chemical shift of HP-pyridine-N-oxide **6'** in various pH environments in MeOD/H_2O media (full spectra).

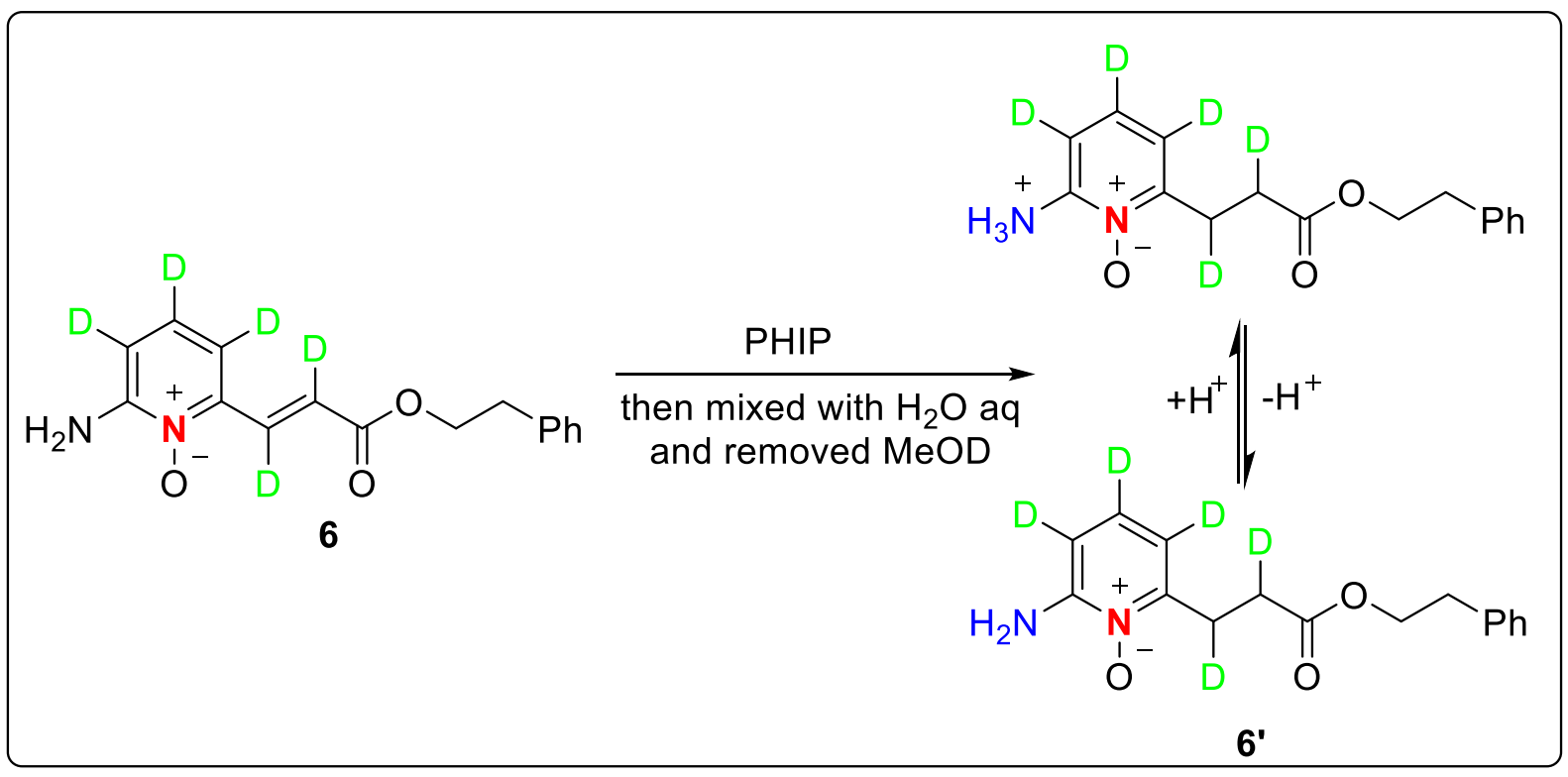

Procedure 2-(removal of MeOD): A sample containing amine substituted ^{15}N-oxide **6** (5.0 mM), [Rh(dppb)(COD)][BF_4] (0.33 mM) in MeOD-d_4 (200 μL) was freshly prepared. A gentle N_2 flow was bubbling into the sample for 50s to remove oxygen, and the sample was equilibrated at 335 K for 2 min before the test. The MINERVA[5] sequence in Fig. 1 (2) of ref [5b] with a final 90° pulse was executed. After the polarization transfer, H_2O buffer with the indicated pH (200 μL) was added, and MeOD was removed via vacuum at 371 K within 12s while keeping the sample under a magnetic field of 200 mT. The sample was then filtered to remove the insoluble precipitate. The clear filtrate was then detected

using a 90° pulse to obtain the 1D ^{15}N-NMR spectrum of **6'** (Figure S55b).

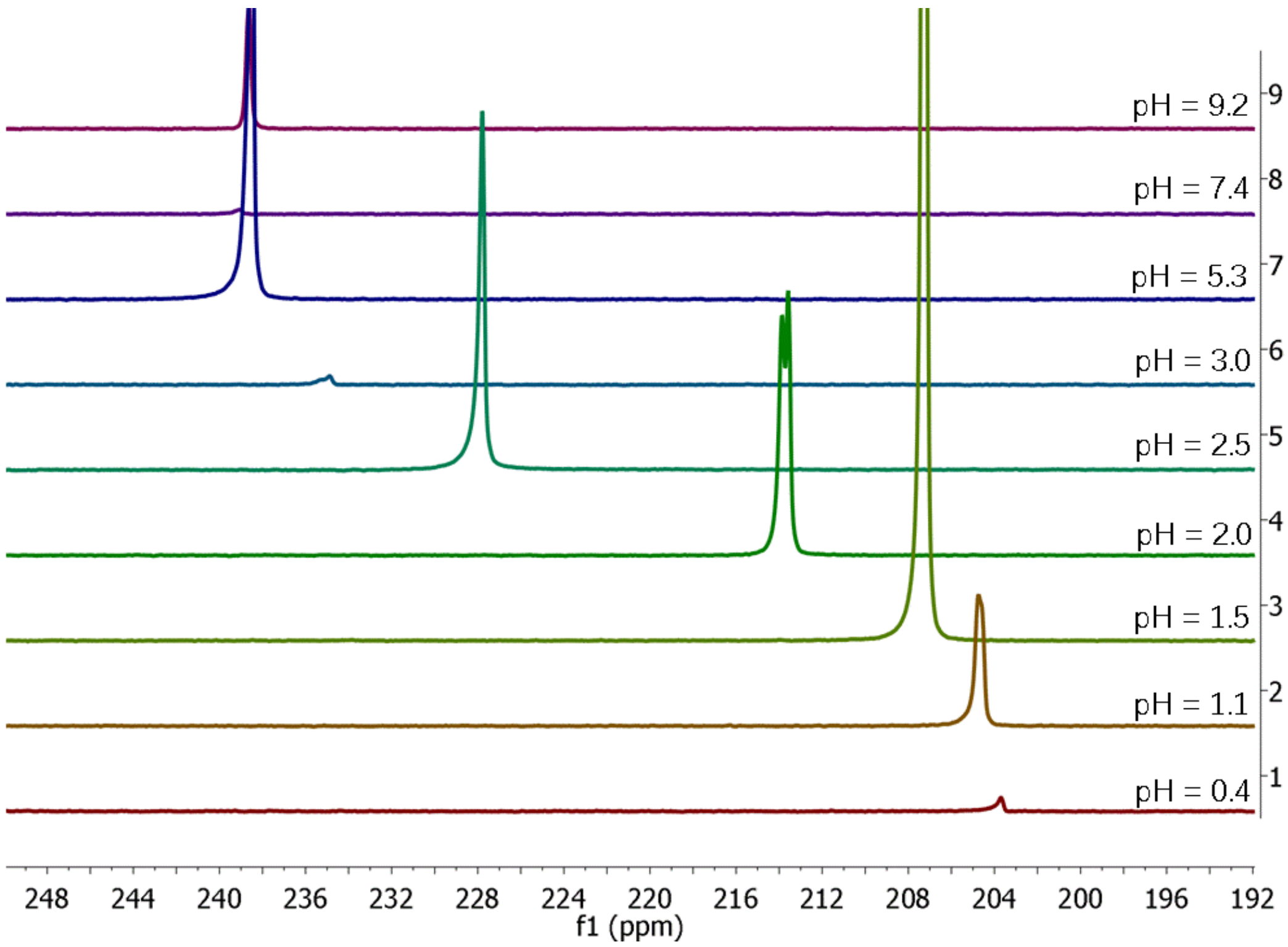

Figure S55b-1. ^{15}N chemical shift of HP-pyridine-N-oxide **6'** after the removal of MeOD in various pH environments.

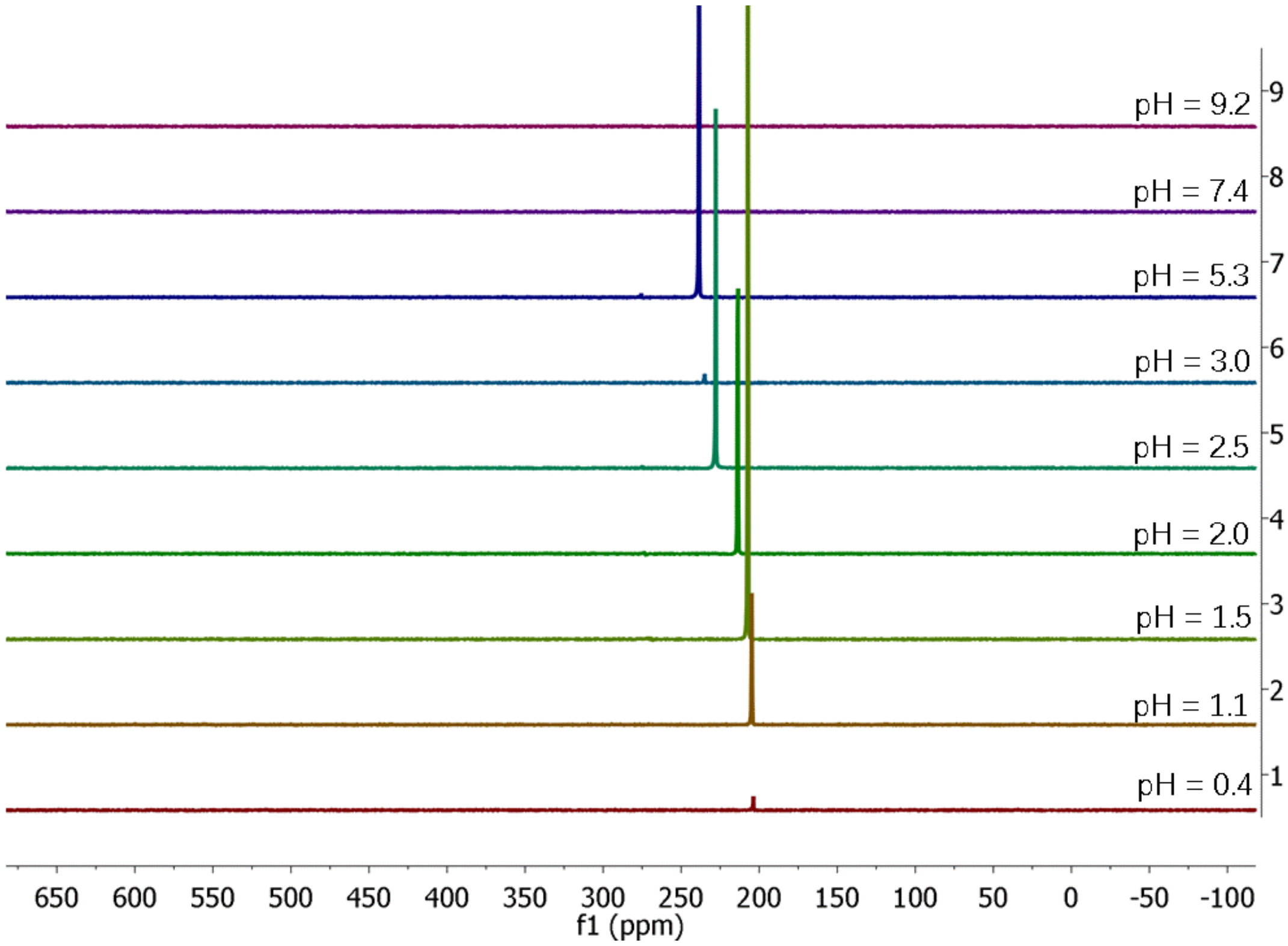

pH	0.4	1.1	1.5	2.0	2.5	3.0	5.3	7.4
Chemical Shift (ppm)-1st measure	202.5	203.5	206.0	212.5	226.6	233.7	237.3	237.9
Chemical Shift (ppm)-2nd measure	202.5	203.5	206.4	211.4	226.5	232.9	237.2	237.3
Chemical Shift (ppm)-3rd measure	202.1	203.7	206.4	212.7	227.8	233.6	238.1	237.5
Average chemical shrift (ppm)	202.4±0.3	203.6±0.2	206.3±0.3	212.2±0.7	227.0±0.8	233.4±0.5	237.5±0.5	237.6±0.4

Figure S55b-2. ^{15}N chemical shift of HP-pyridine-N-oxide **6'** after the removal of MeOD in various pH environments (full spectra).

3. Hyperpolarization with aqueous workup

3.1 T_1 measurement in biocompatible aqueous media.

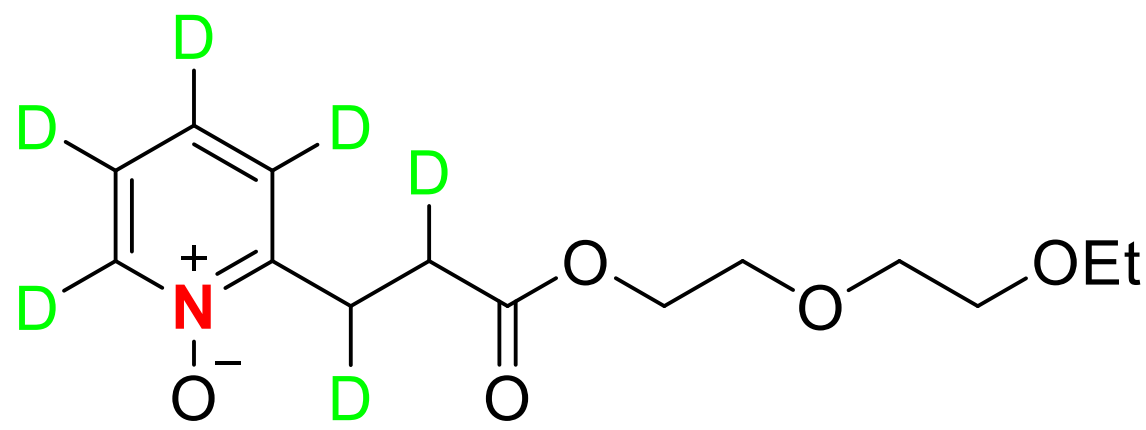

2'

A sample containing ^{15}N-oxide **2'** (8.0 mM), [Rh(dppb)(COD)][BF_4] (0.4 mM) in MeOD-d_4 (220 μL) was freshly prepared. A gentle N_2 flow was bubbling into the sample for 50s to remove oxygen, and the sample was equilibrated at 335 K for 2 min before the test. The MINERVA[5] sequence in Fig. 1 (2) of

ref [5b] with a final 90° pulse was executed. After the polarization transfer, PBS buffer in H_2O (pH = 7.4, 150 µL) was added, and MeOD was removed *via* vacuum at 371 K within 12s while keeping the sample with a magnetic field of 200 mT. The solution was filtered to remove insoluble precipitate. The clear filtrate was then detected with a tip flip angle (10°) pulse to obtain the 2D ^{15}N-NMR spectrum for the T_1 measurement (Figure S56).

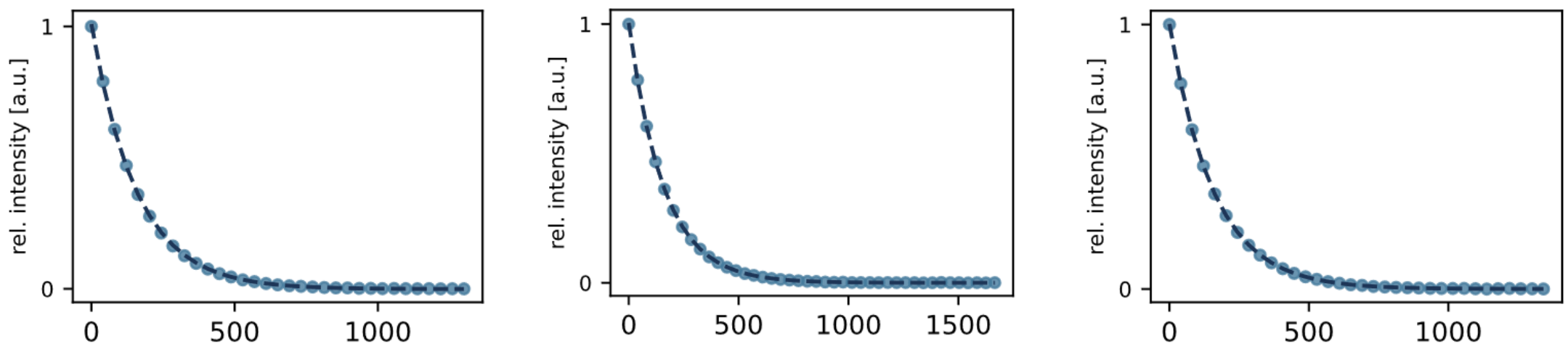

T_1 (s)	T_1 (s)-1st	T_1 (s)-2nd	T_1 (s)-3rd	AVR T_1 (s)
value	168.5	169.6	168.7	168±1

Figure S56. T_1 @ 7.0 T in H_2O.

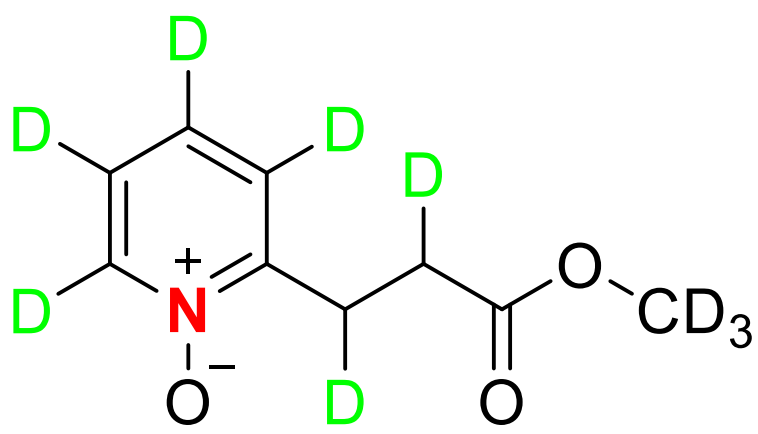

4'

A sample containing ^{15}N-oxide **4’** (31.6 mM), [Rh(dppb)(COD)][BF_4] (1.1 mM) in MeOD-d_4 (190 µL) was freshly prepared. A gentle N_2 flow was bubbling into the sample for 50s to remove oxygen, and the sample was equilibrated at 335 K for 2 min before the test. The MINERVA[5] sequence in Fig. 1 (2) of ref [5b] with a final 90° pulse was executed. After the polarization transfer, NaOH solution in H_2O (100 mM, 150 µL) was added, and MeOD was removed *via* vacuum at 371 K within 12s while keeping the sample with a magnetic field of 200 mT. Then the sample was neutralized with aqueous buffer (H_2O, pH=1, 150 µL) and filtered to remove insoluble precipitate. The clear filtrate was then detected with a tip flip angle (10°) pulse to obtain the 2D ^{15}N-NMR spectrum for the T_1 measurement (Figure S57).

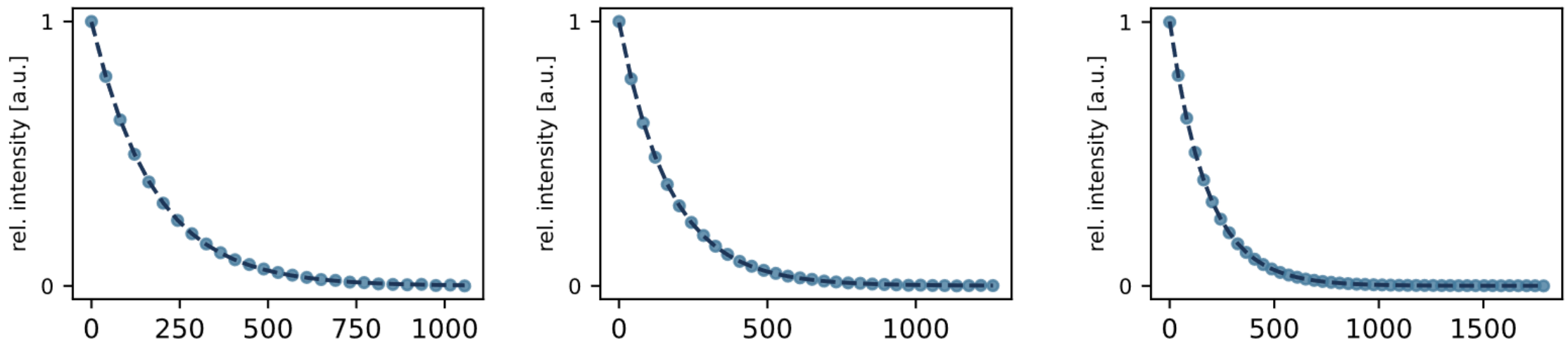

T_1 (s)	T_1 (s)-1st	T_1 (s)-2nd	T_1 (s)-3rd	AVR T_1 (s)
value	188.3	182.6	190.3	187±4

Figure S57. T_1 @ 7.0 T in H_2O.

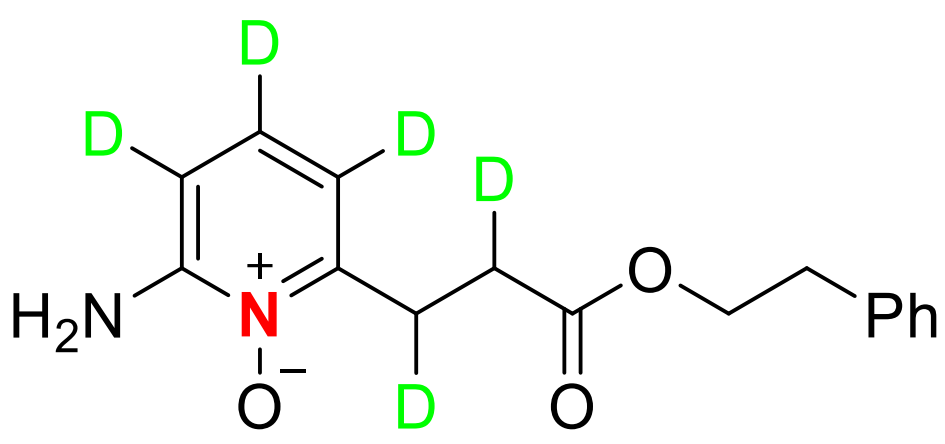

6'

A sample containing ^{15}N-oxide **4'** (21.4 mM), [Rh(dppb)(COD)][BF_4] (1.4 mM) in MeOD-d_4 (210 μL) was freshly prepared. A gentle N_2 flow was bubbling into the sample for 50s to remove oxygen, and the sample was equilibrated at 335 K for 2 min before the test. The MINERVA[5] sequence in Fig. 1 (2) of ref [5b] with a final 90° pulse was executed. After the polarization transfer, NaOH solution in H_2O (100 mM, 150 μL) was added, and MeOD was removed *via* vacuum at 371 K within 12s while keeping the sample with a magnetic field of 200 mT. Then the sample was neutralized with aqueous buffer (H_2O, pH=1, 150 μL) and filtered to remove insoluble precipitate. The clear filtrate was then detected with a tip flip angle (10°) pulse to obtain the 2D ^{15}N-NMR spectrum for the T_1 measurement (Figure S58).

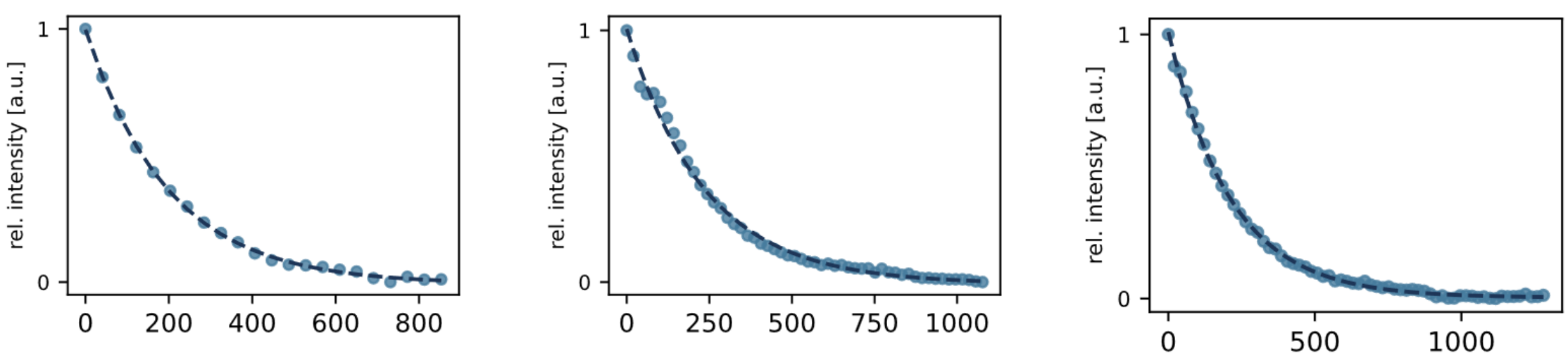

T_1 (s)	T_1 (s)-1st	T_1 (s)-2nd	T_1 (s)-3rd	AVR T_1 (s)
value	215.9	290.8	256.2	254±38

Figure S58. T_1 @ 7.0 T in H_2O.

3.2 Biochemically relevant transformation in aqueous media.

~Hydrolysis of pyridine-^{15}N-Oxide 4' with esterase

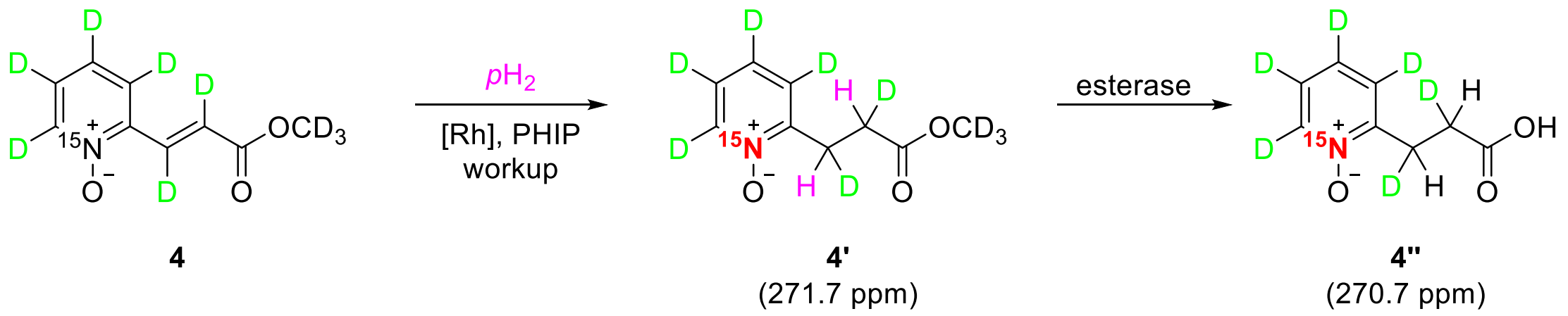

Procedure: A sample containing ^{15}N-oxide **4** (17.2 mM), [Rh(dppb)(COD)][BF_4] (0.69 mM) in acetone-d_6 (209 µL) was freshly prepared. A gentle N_2 flow was bubbling into the sample for 50s to remove oxygen, and the sample was equilibrated at 335 K for 2 min before the test. The MINERVA[5] sequence in Fig. 1 (2) of ref [5b] with a final 90° pulse was executed. After the polarization transfer, PBS buffer (pH = 7.4, in H_2O, 150 µL) was added, and acetone-d_6 was removed by vacuum at 345 K within 10s while keeping the sample under a magnetic field of 200 mT. The sample was then filtered to remove the insoluble precipitate. The clear filtrate was then mixed with esterase (150 units in 100 µL PBS buffer, pH = 7.4) and detected using a 90°pulse to obtain the 1D ^{15}N-NMR spectrum of **4'** and **4''** (Figure S59), or in the other case, the sample was detected with a tip flip angle (30°, every 10s) pulse to obtain the 2D ^{15}N-NMR spectra (Figure S60).

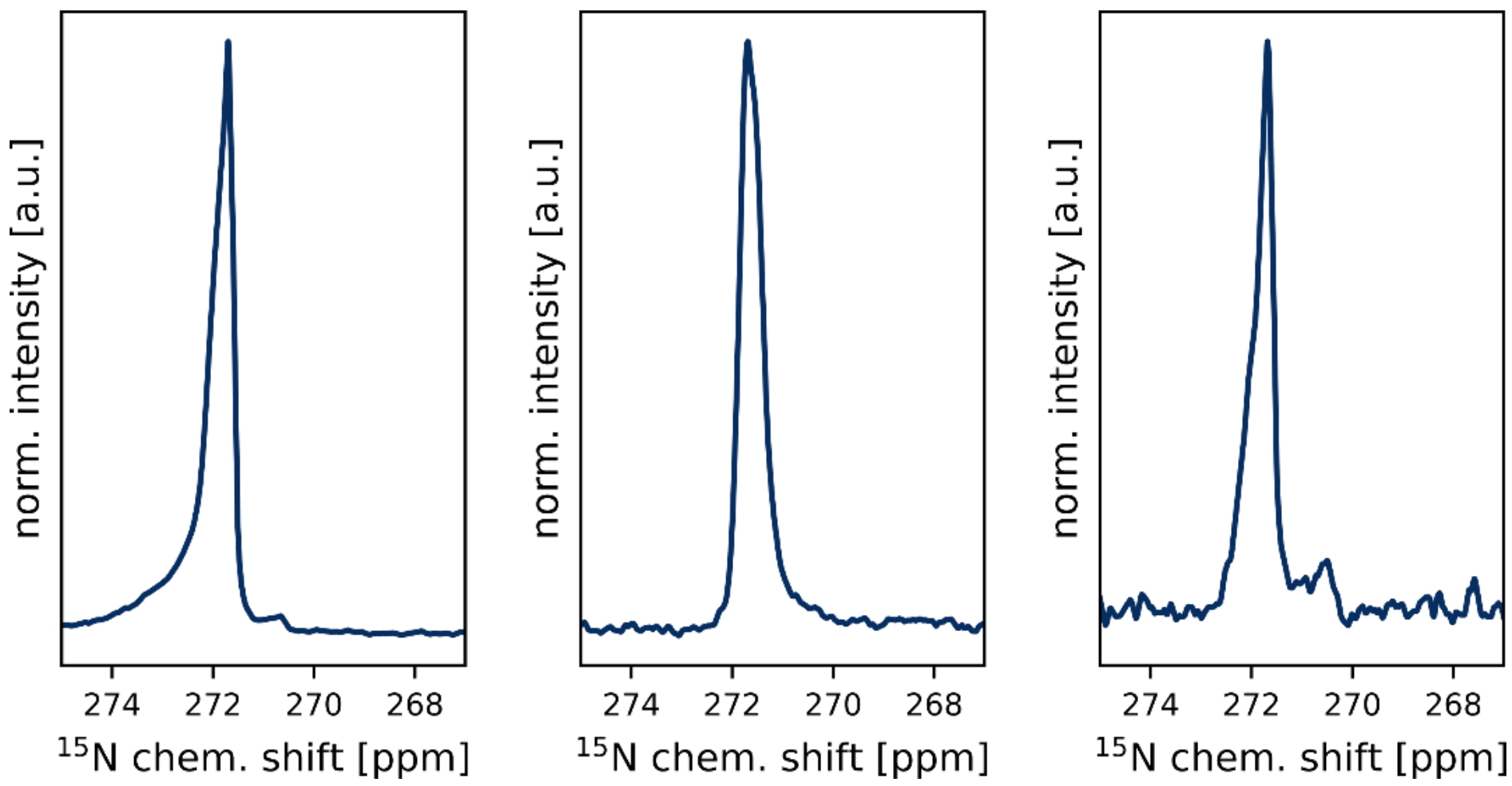

Figure S59 1D ^{15}N-NMR spectrum of **4'** (271.7 ppm) **4''** (270 ppm) after 40 s in PBS buffer of H_2O. A minor fraction of the compound (<2%) appears to be cleaved after 40s.

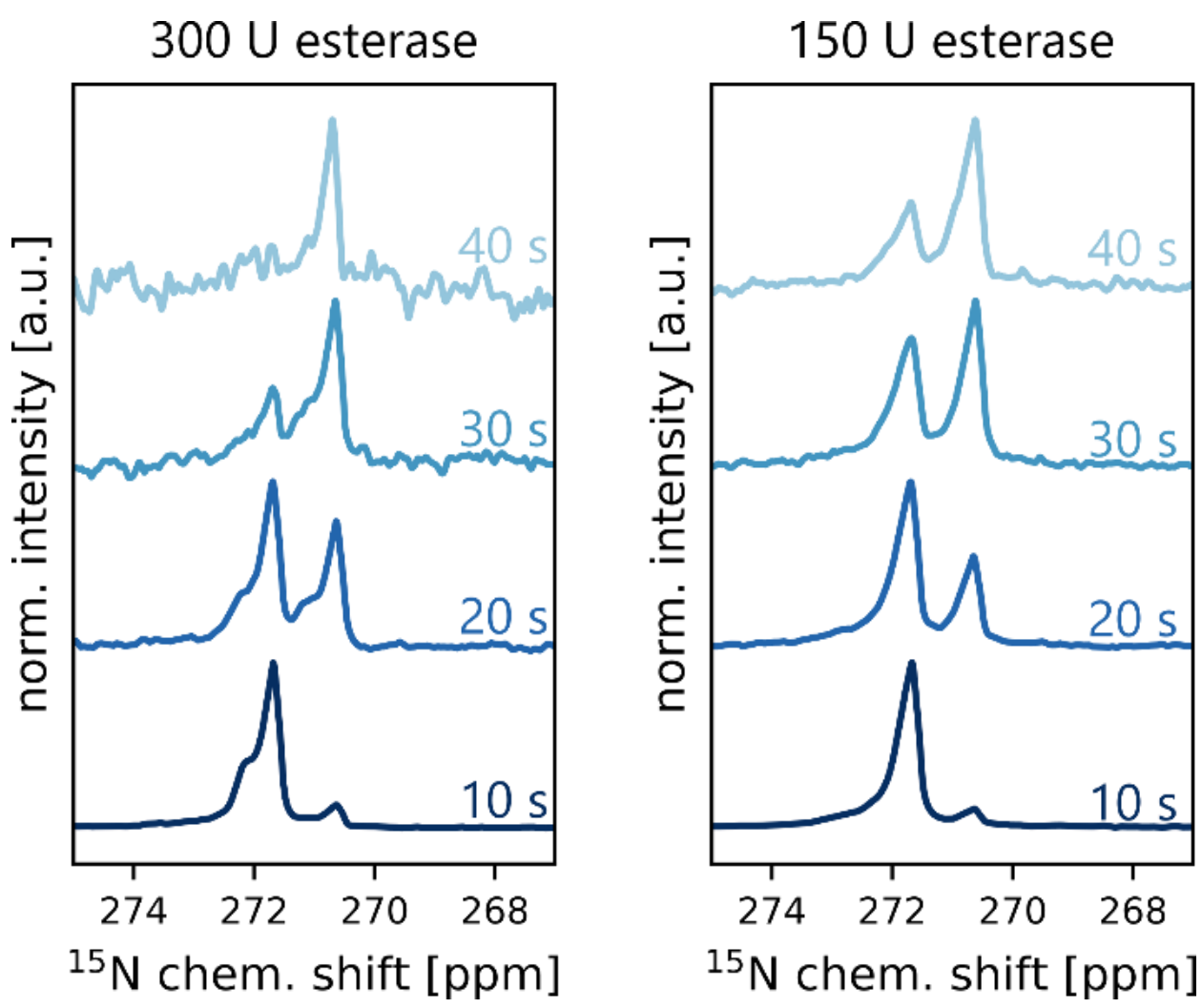

Figure S60 Series of ^{15}N-NMR spectra acquired upon mixing the esterase containing solution (300/150 U) with the aqueous solution of hyperpolarized **4'**. Spectra were acquired using flip angle pulse of 30° and 10 s delays between successive scans

~H_2O_2 detection with pyridine-^{15}N 7' in aqueous media

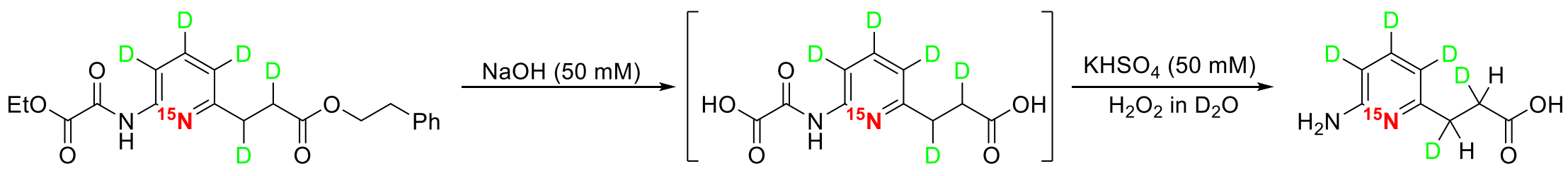

7' **7''** (207.8 ppm) **7'''** (235.7 ppm)

Procedure A: a sample containing ^{15}N-pyridine **7** (4.6 mM), [Rh(dppb)(COD)][BF_4] (0.37 mM) in MeOD-d4 (173 µL) was freshly prepared. A gentle N_2 flow was bubbling into the sample for 50s to remove oxygen, and the sample was equilibrated at 335 K for 2 min before the test. The MINERVA[5] sequence in Fig. 1 (2) of ref [5b] with a final 90° pulse was executed. After the polarization transfer, NaOH solution (50 mM in D_2O, 150 µL) was added, and MeOD was removed by vacuum at 371 K within 10s while keeping the sample under a magnetic field of 200 mT. The sample was then filtered to remove the insoluble precipitate. The clear filtrate was then mixed with a solution of $KHSO_4$ (50 mM) containing H_2O_2 (0, 250, or 1000 mM) in D_2O (150 µL) and detected using a 90°pulse to obtain the 1D ^{15}N-NMR spectrum of 7'' and/or 7''' (Figure S61).

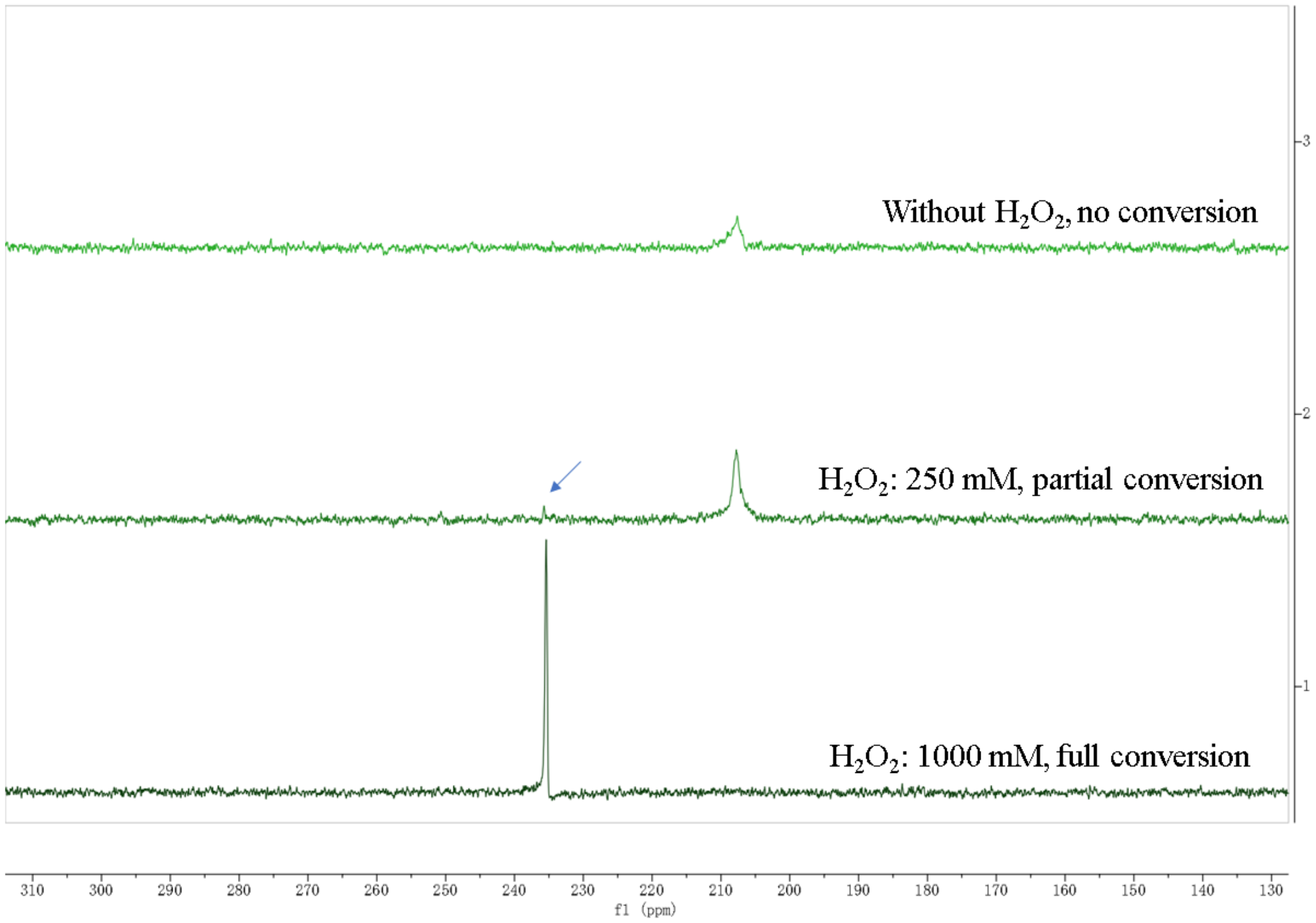

Figure S61 ^{15}N-NMR spectra of **7''** and **7'''** in **neutral** D_2O when treated with H_2O_2 of different concentrations and the negative control experiment.

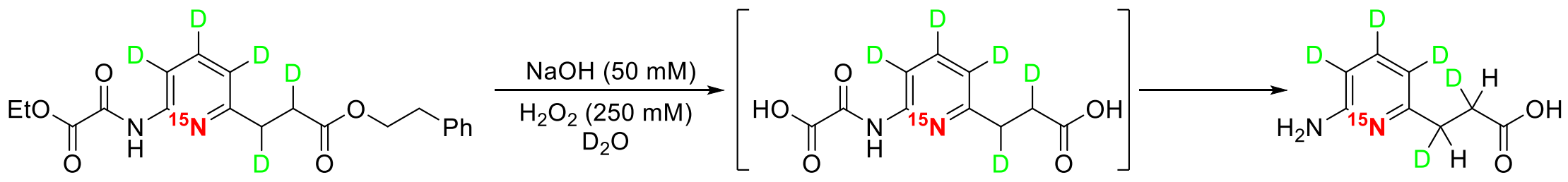

Procedure B: a sample containing ^{15}N-pyridine **7** (4.6 mM), [Rh(dppb)(COD)][BF_4] (0.37 mM) in MeOD-d_4 (173 μL) was freshly prepared. A gentle N_2 flow was bubbling into the sample for 50s to remove oxygen, and the sample was equilibrated at 335 K for 2 min before the test. The MINERVA[5] sequence in Fig. 1 (2) of ref [5b] with a final 90° pulse was executed. After the polarization transfer, NaOH solution (50 mM in D_2O, 150 μL, containing H_2O_2: 250 mM) was added, and MeOD was removed by vacuum at 371 K within 10s while keeping the sample under a magnetic field of 200 mT. The sample was then filtered to remove the insoluble precipitate. The clear filtrate was then detected using a 90°pulse to obtain the 1D ^{15}N-NMR spectrum of **7''** (237.2 ppm) and **7'''** (252.0 ppm) (Figure S62).

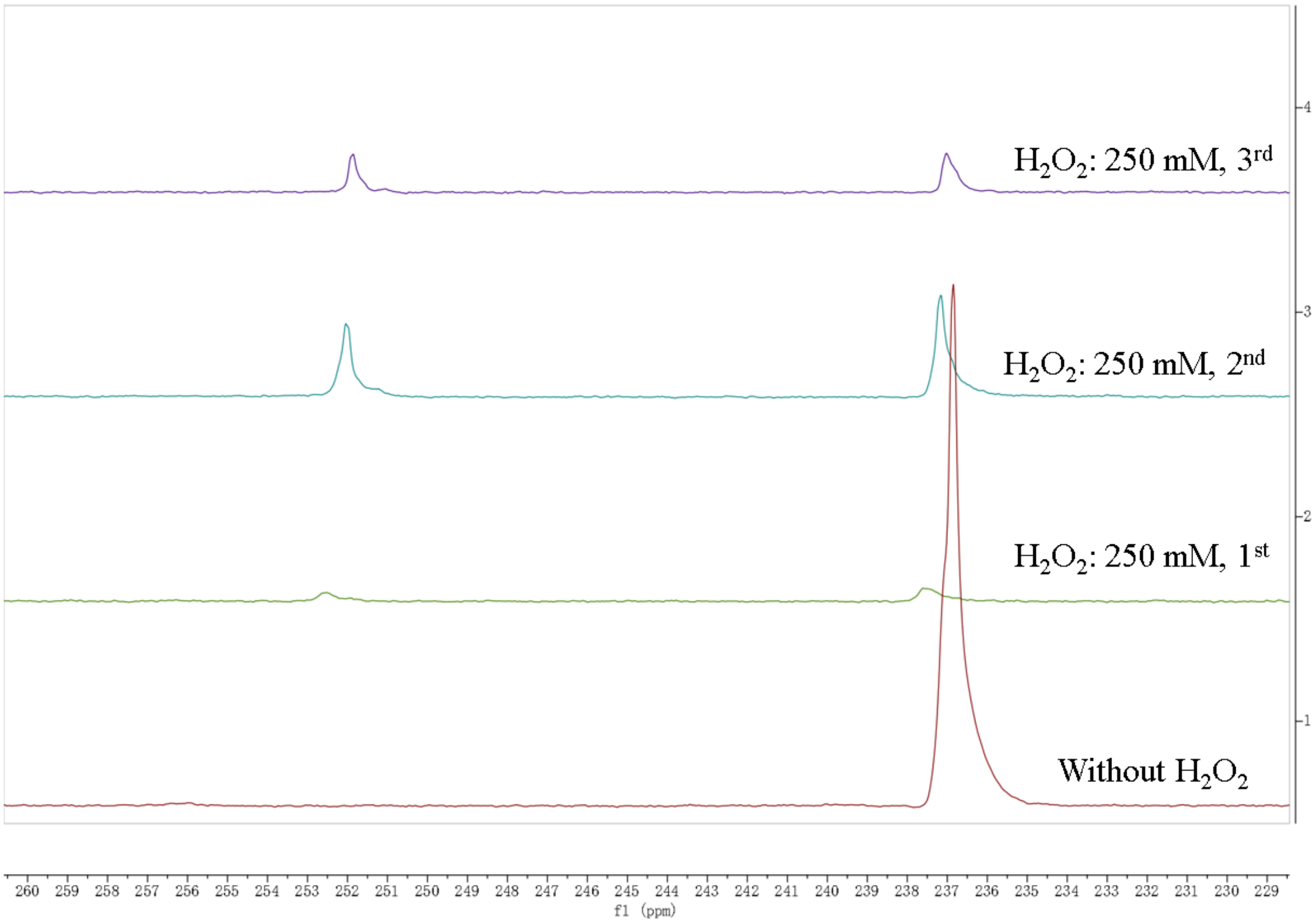

Figure S62 ^{15}N-NMR spectra of 3 parallel experiments to show the conversion from **7''** (237.2 ppm) to **7'''** (252.0 ppm) in **alkaline** D_2O when treated with H_2O_2 and the negative control experiment.

3.3 Quantification of residual methanol concentration after workup.

To quantify the residual methanol content after workup, the complete experimental procedure was performed under the same conditions as used for the hyperpolarization experiments. H_2O buffer (200 μL, pH = 7) was added, and MeOD-d_6 was removed under vacuum at 371 K within 12 s while maintaining the sample in a magnetic field of 200 mT. The resulting sample was filtered to remove insoluble precipitate. Residual methanol content was quantified by ^{13}C NMR spectroscopy using a 90° pulse experiment (NS = 32). Quantification was performed by comparison to an external 0.2 M ^{13}C-sodium acetate reference, with correction for the different ^{13}C isotopic abundances of the labeled reference compound and the residual methanol at natural ^{13}C abundance.

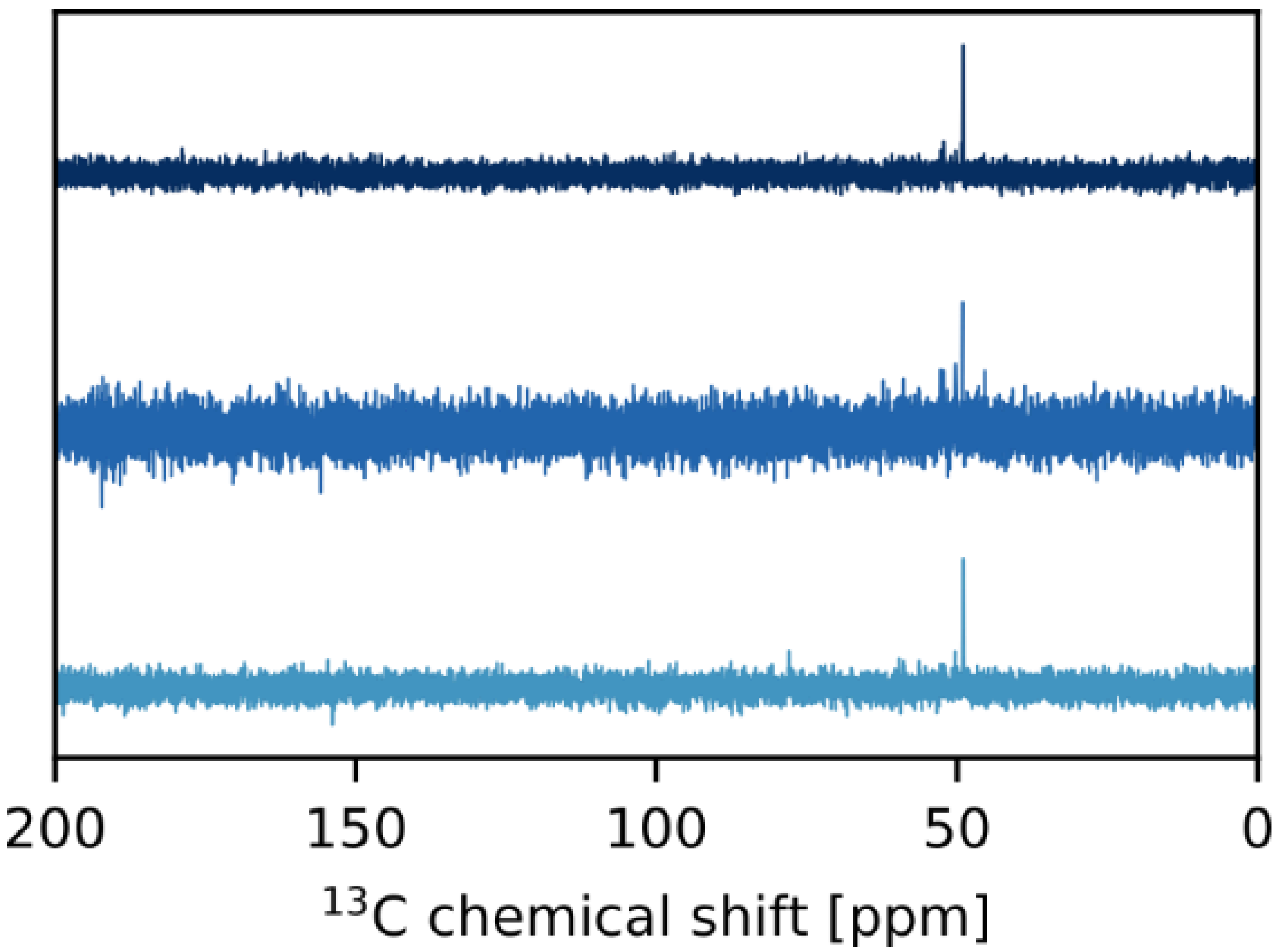

Figure S63. Thermal ^{13}C NMR spectra used to quantify residual methanol concentration after workup.

4. NMR & HR-MS spectra for the compound's characterization.

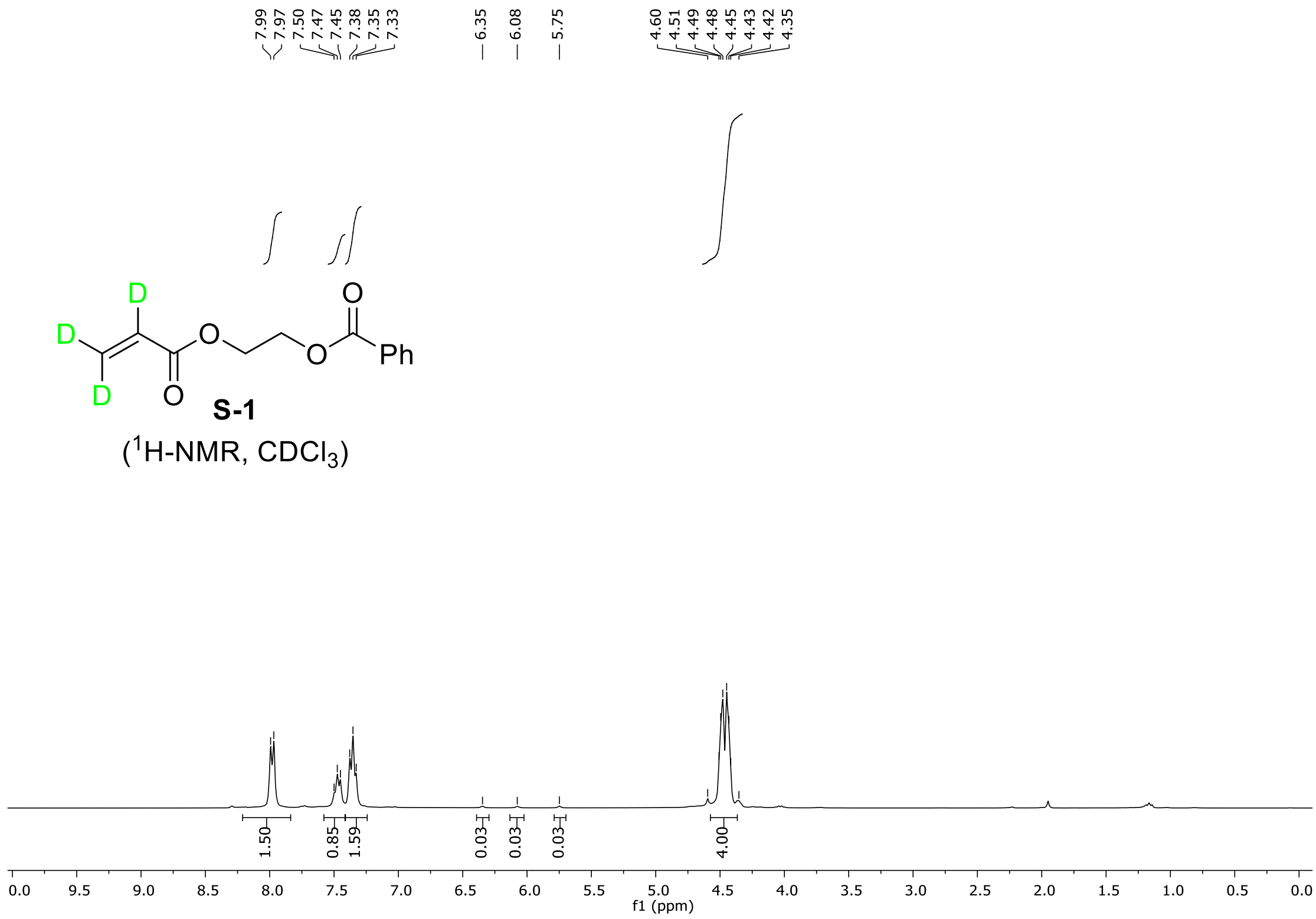

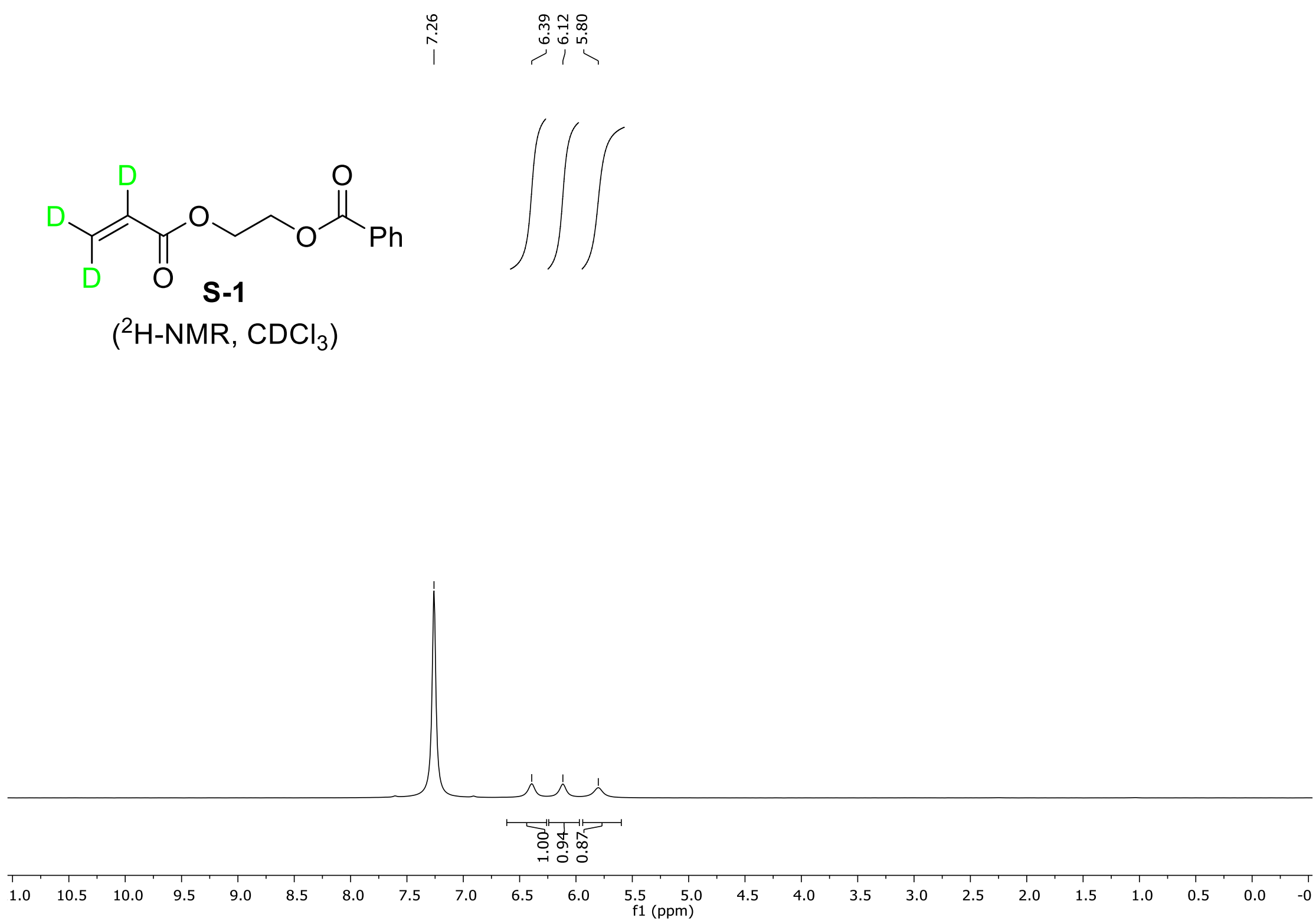

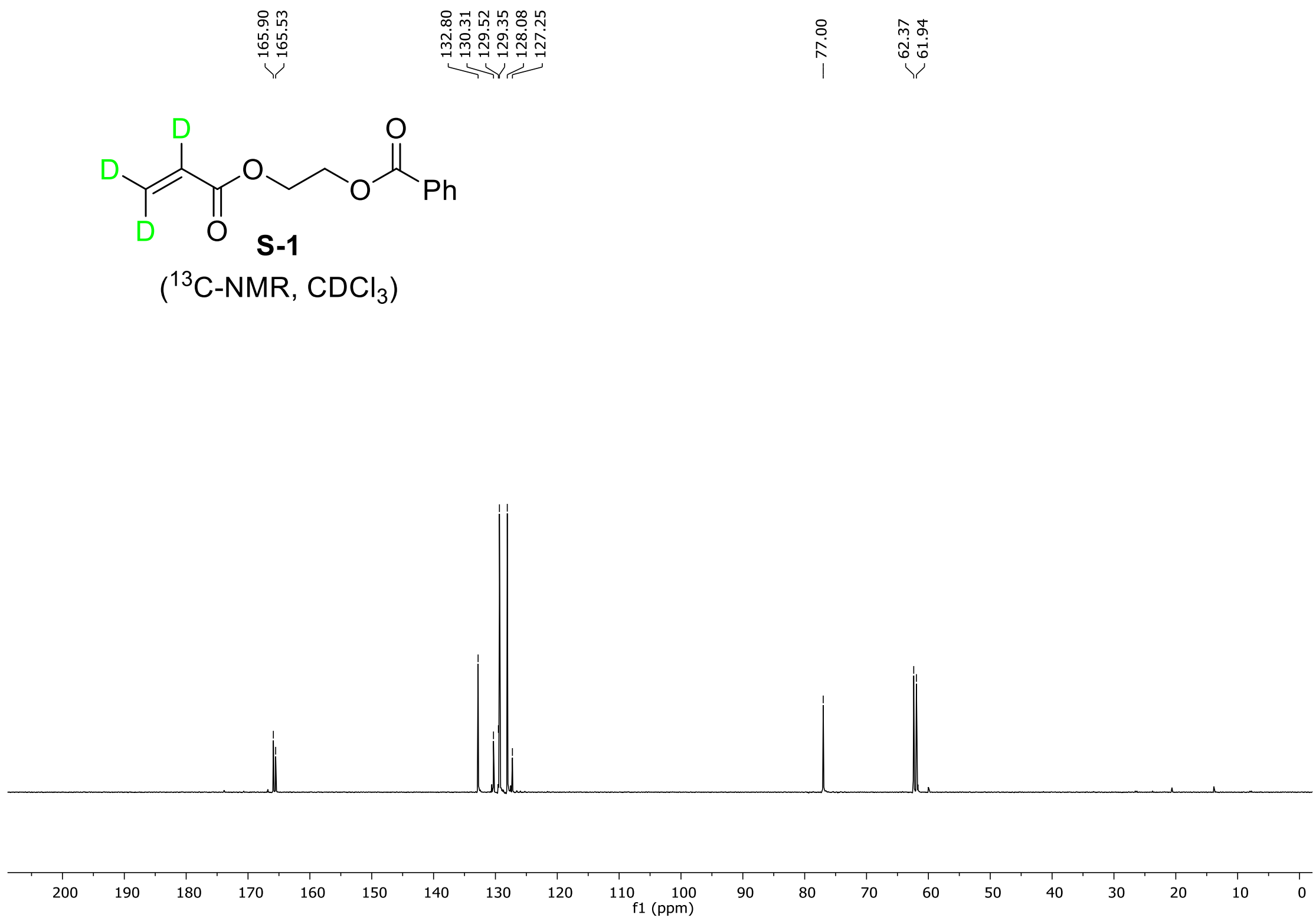

Display Report

**Analysis Info**		Acquisition Date	31.07.2024 21:23:15
Analysis Name	Z:\Data\2024\2407\sam310724\yli00093_1_01_126749.d		
Method	hystar_pl.m	Operator	BDAL@DE
Sample Name	yli00093	Instrument / Ser#	micrOTOF 10237
Comment			

Acquisition Parameter

Source Type	ESI	Ion Polarity	Positive	Set Nebulizer	1.2 Bar
Focus	Not active			Set Dry Heater	180 °C
Scan Begin	50 m/z	Set Capillary	4500 V	Set Dry Gas	4.0 l/min
Scan End	1600 m/z	Set End Plate Offset	-500 V	Set Divert Valve	Source

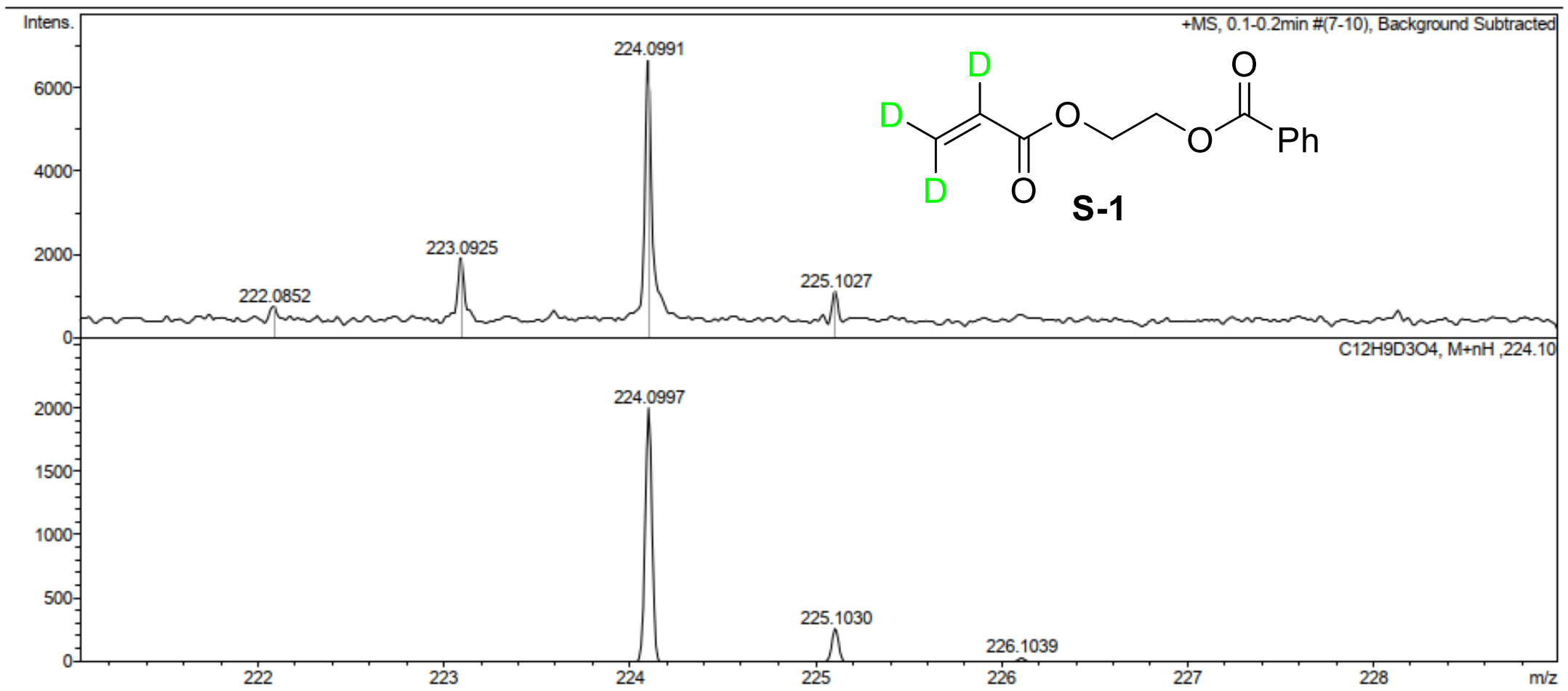

Bruker Compass DataAnalysis 4.0 printed: 31.07.2024 12:38:30 Page 1 of 1

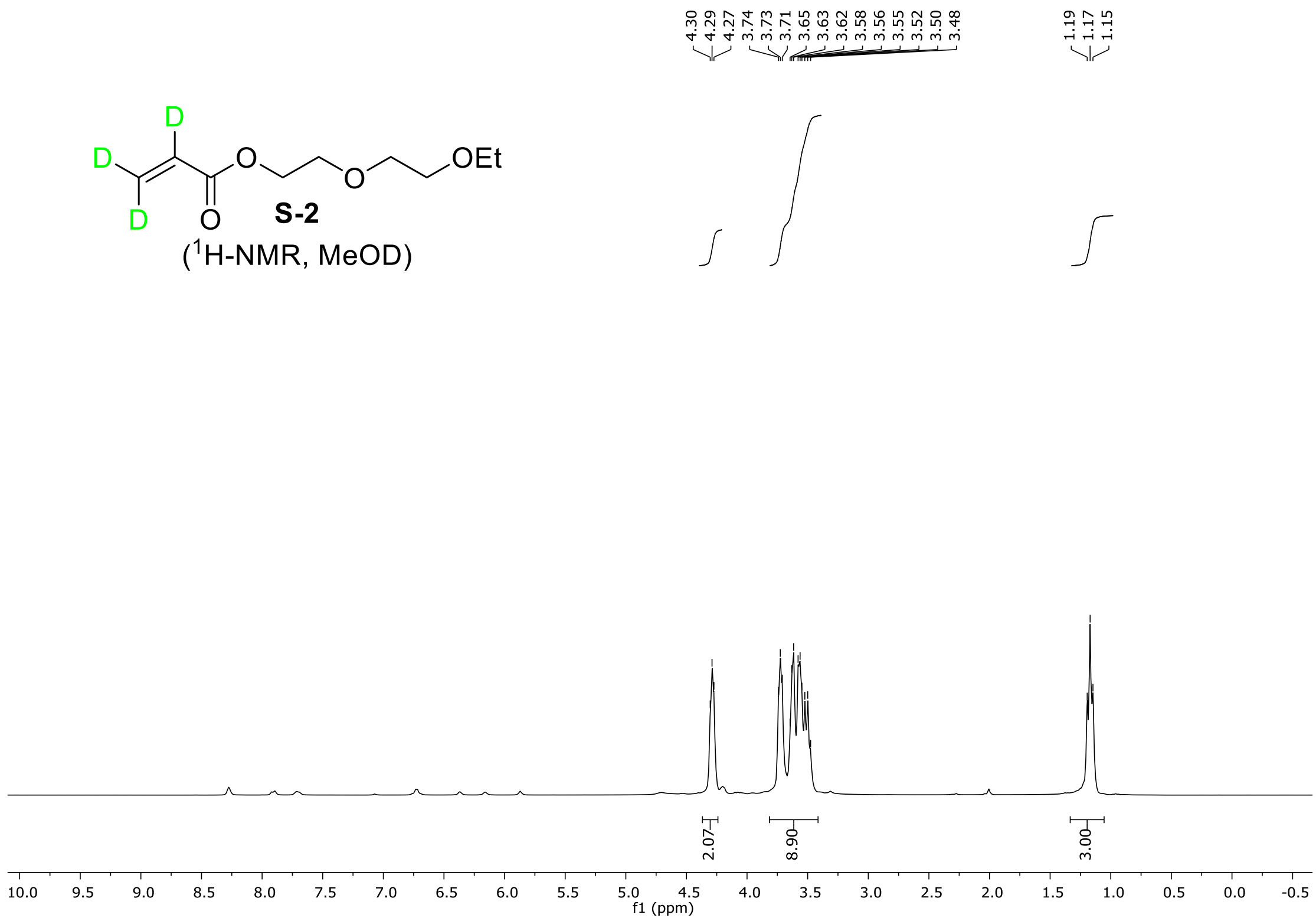
D
D
D
O
O
O
OEt
S-2
(1H-NMR, MeOD)
4.30
4.29
4.27
3.74
3.73
3.71
3.65
3.63
3.62
3.58
3.56
3.55
3.52
3.50
3.48
1.19
1.17
1.15
2.07
8.90
3.00
f1 (ppm)

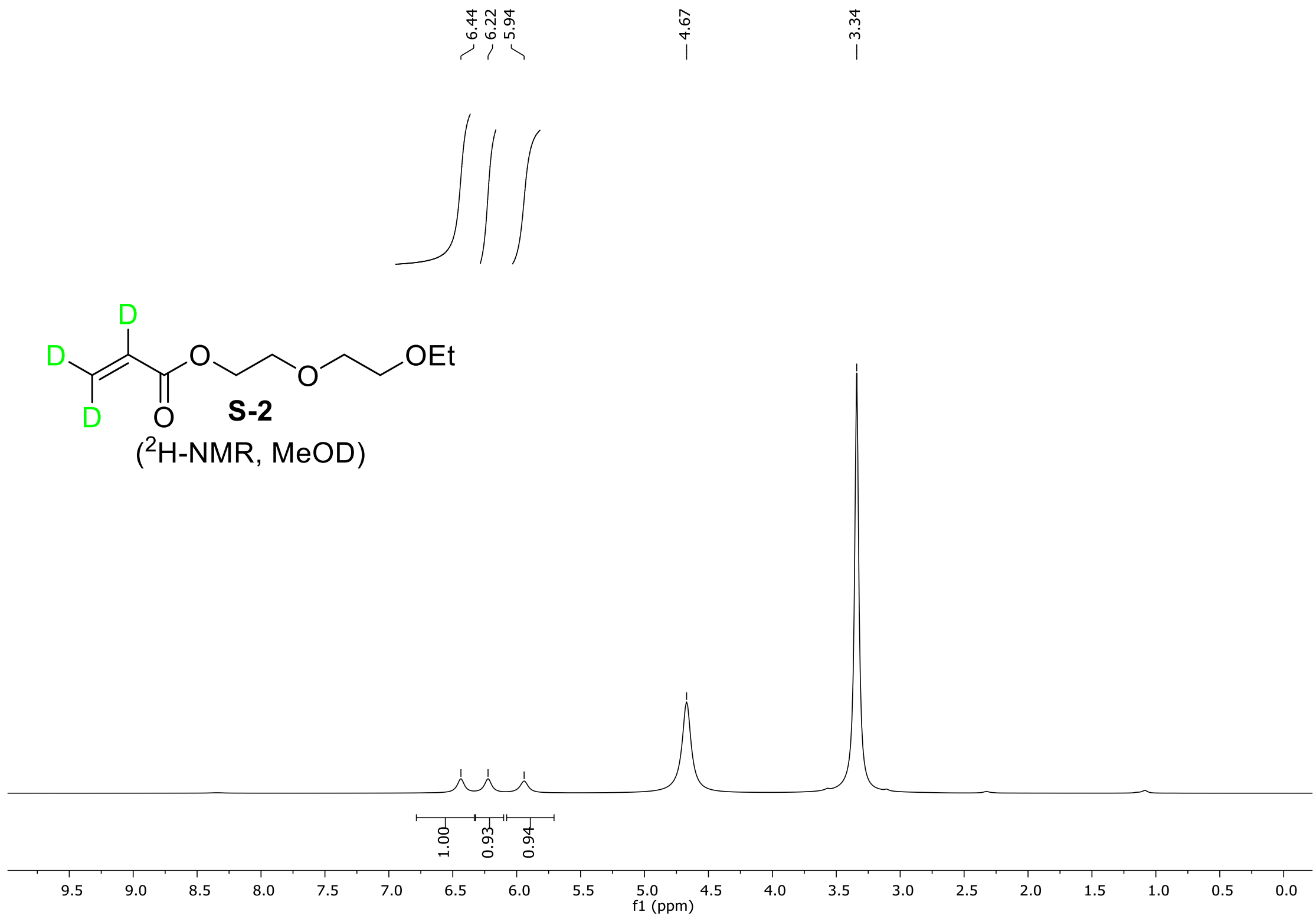
6.44
6.22
5.94
4.67
3.34
D
D
D
O
O
O
OEt
S-2
(2H-NMR, MeOD)
1.00
0.93
0.94
f1 (ppm)

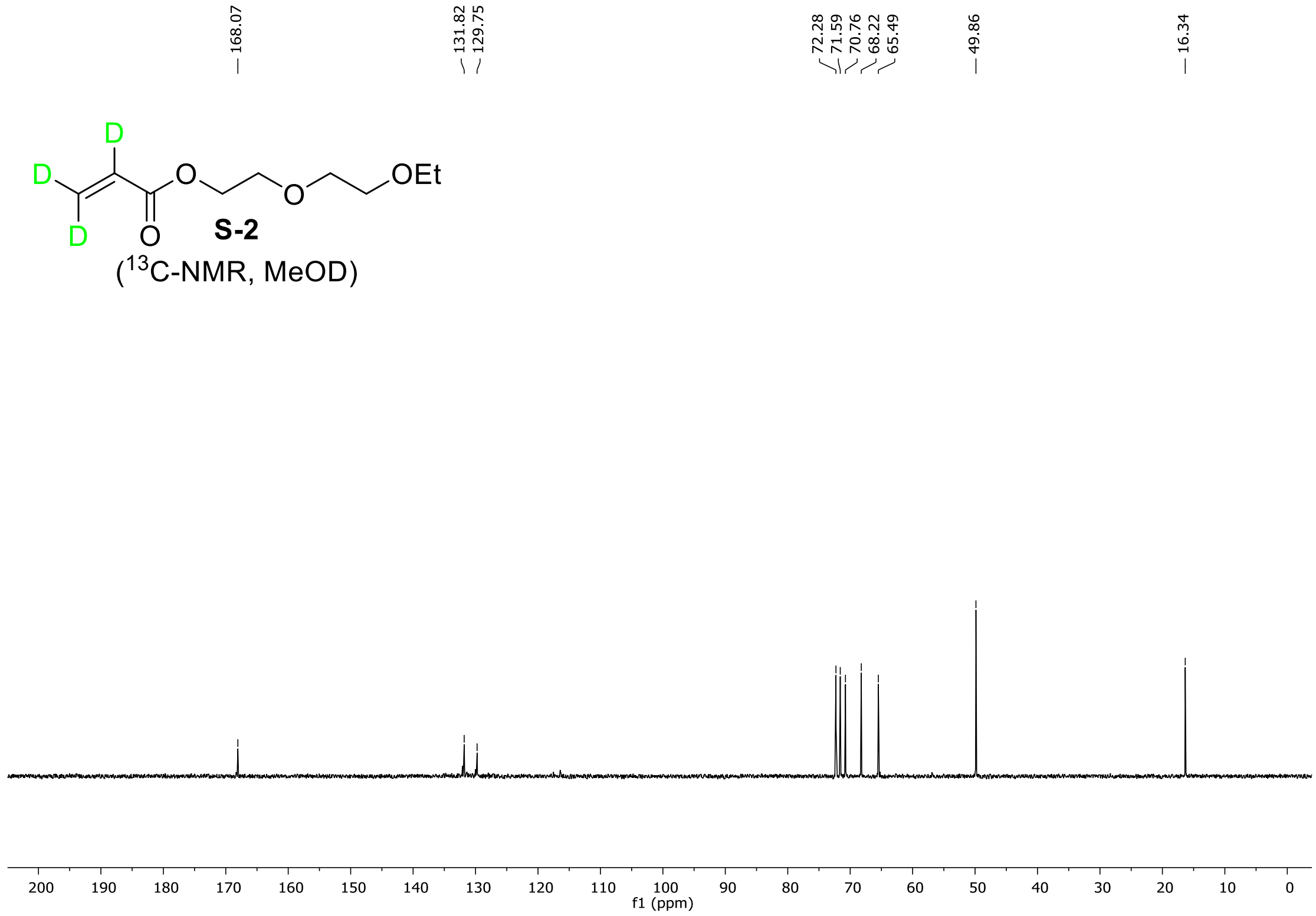

Display Report

Analysis Info			
Analysis Name	Z:\Data\2024\2407\sam310724\yli00094_2_01_126750.d	Acquisition Date	31.07.2024 21:27:55
Method	hystar_pl.m	Operator	BDAL@DE
Sample Name	yli00094	Instrument / Ser#	micrOTOF 10237
Comment			

Acquisition Parameter					
Source Type	ESI	Ion Polarity	Positive	Set Nebulizer	1.2 Bar
Focus	Not active			Set Dry Heater	180 °C
Scan Begin	50 m/z	Set Capillary	4500 V	Set Dry Gas	4.0 l/min
Scan End	1600 m/z	Set End Plate Offset	-500 V	Set Divert Valve	Source

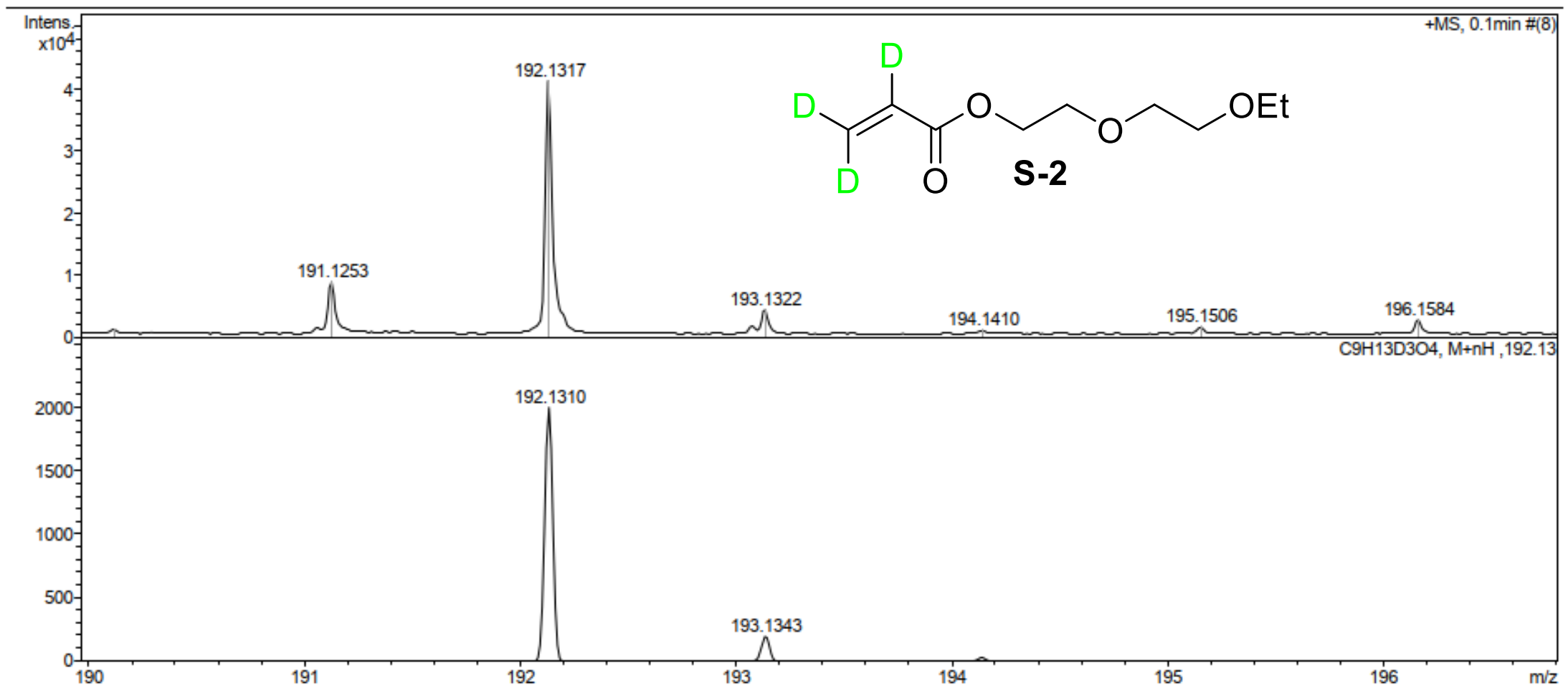

Bruker Compass DataAnalysis 4.0 printed: 31.07.2024 12:43:41 Page 1 of 1

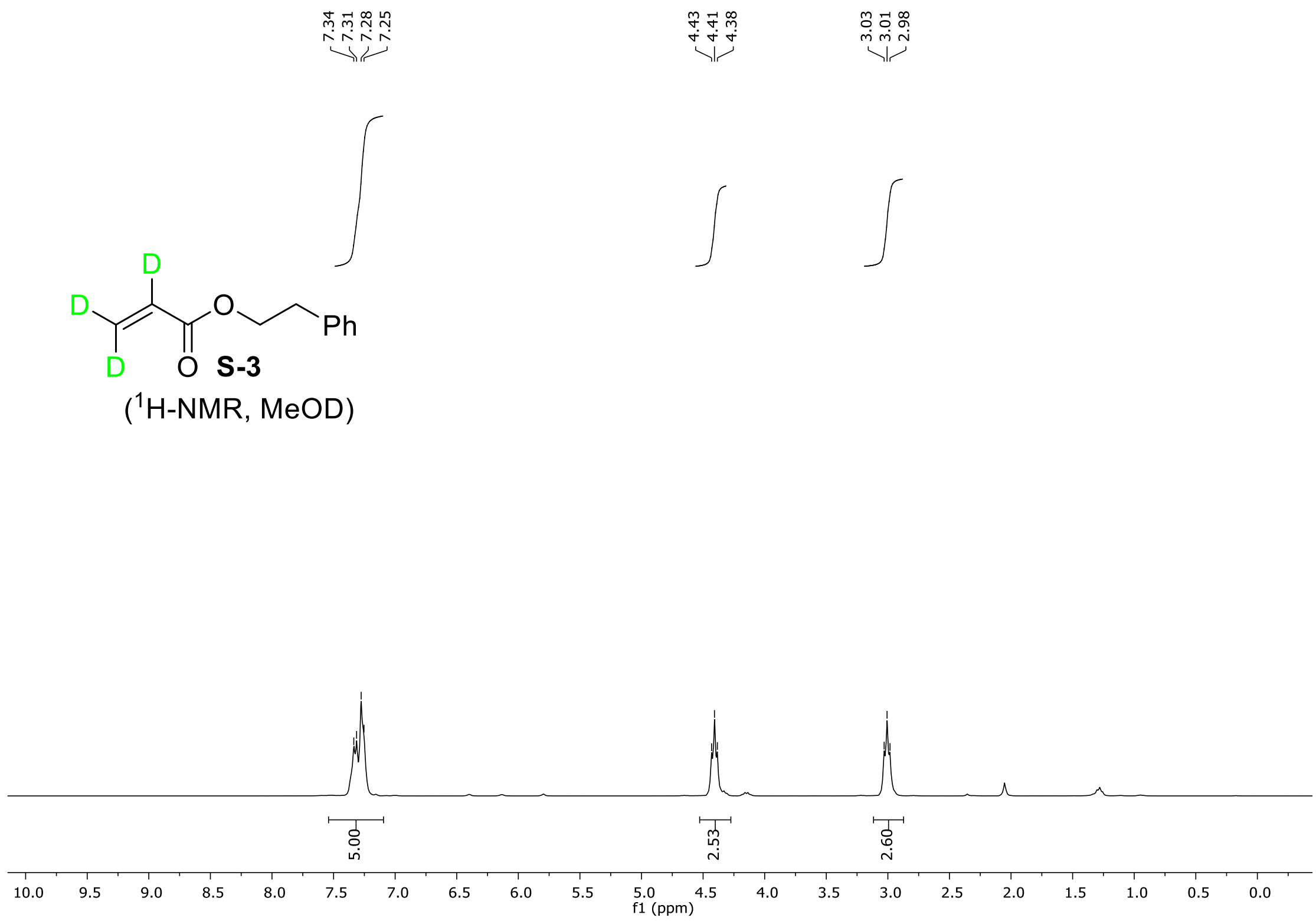

D
D
D
O
O
Ph
S-3
(1H-NMR, MeOD)
7.34
7.31
7.28
7.25
4.43
4.41
4.38
3.03
3.01
2.98
5.00
2.53
2.60
10.0 9.5 9.0 8.5 8.0 7.5 7.0 6.5 6.0 5.5 5.0 4.5 4.0 3.5 3.0 2.5 2.0 1.5 1.0 0.5 0.0
f1 (ppm)

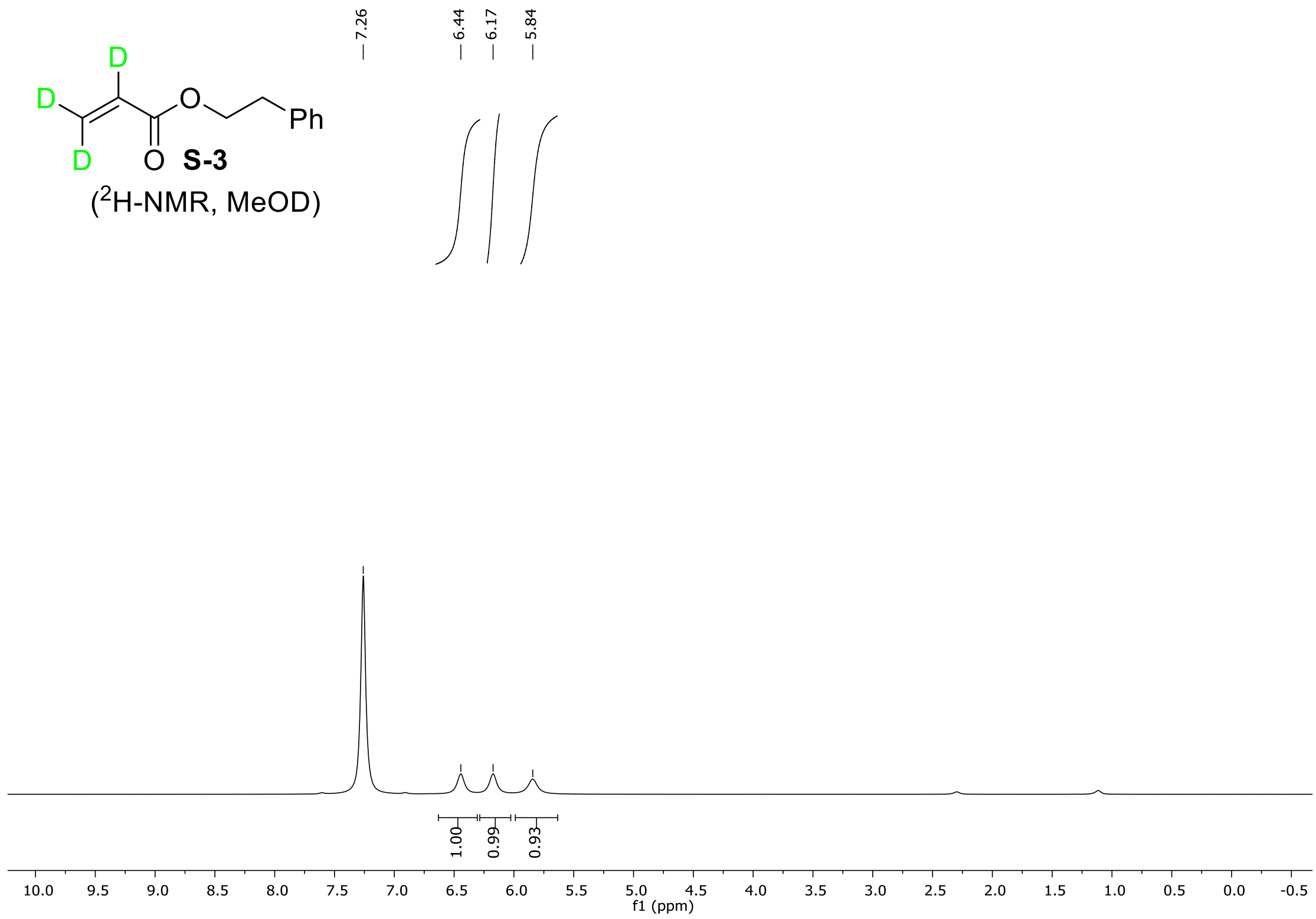

D
D
D
O
O
Ph
S-3
(2H-NMR, MeOD)
7.26
6.44
6.17
5.84
1.00
0.99
0.93
10.0 9.5 9.0 8.5 8.0 7.5 7.0 6.5 6.0 5.5 5.0 4.5 4.0 3.5 3.0 2.5 2.0 1.5 1.0 0.5 0.0 -0.5
f1 (ppm)

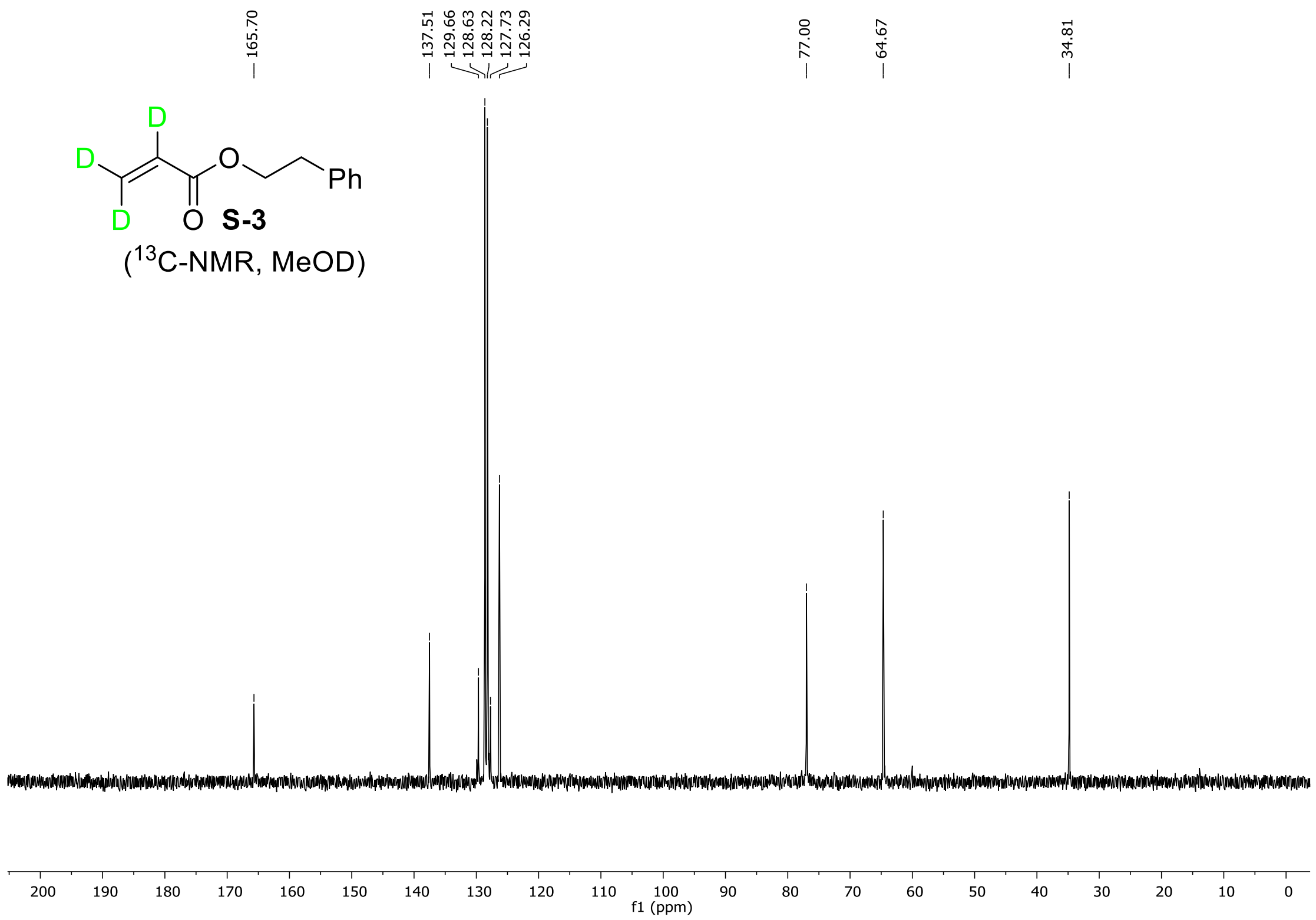

Display Report

Analysis Info

Analysis Name	Z:\Data\2024\2407\sam310724\yli00095_3_01_126751.d	Acquisition Date	31.07.2024 21:32:35
Method	hystar_pl.m		
Sample Name	yli00095	Operator	BDAL@DE
Comment		Instrument / Ser#	micrOTOF 10237

Acquisition Parameter

Source Type	ESI	Ion Polarity	Positive	Set Nebulizer	1.2 Bar
Focus	Not active			Set Dry Heater	180 °C
Scan Begin	50 m/z	Set Capillary	4500 V	Set Dry Gas	4.0 l/min
Scan End	1600 m/z	Set End Plate Offset	-500 V	Set Divert Valve	Source

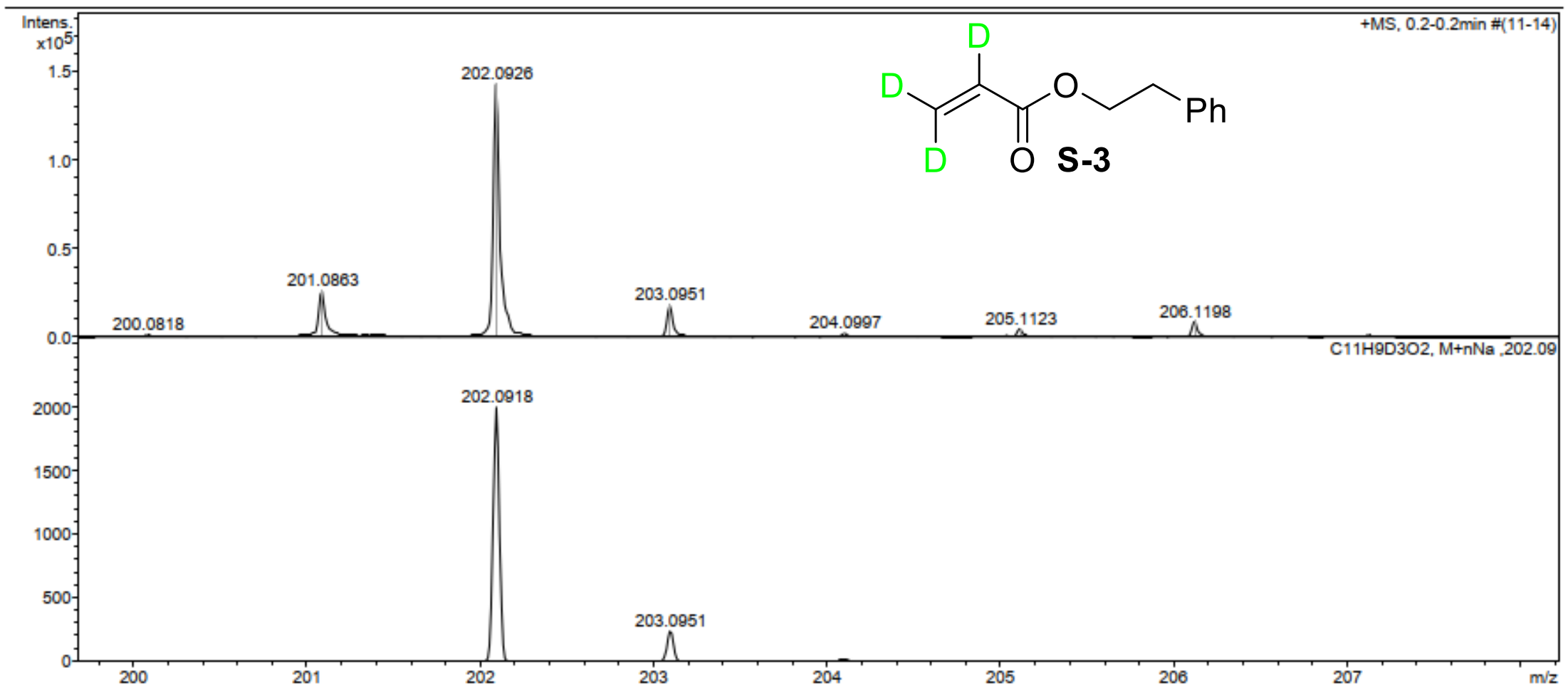

Bruker Compass DataAnalysis 4.0 printed: 31.07.2024 12:48:33 Page 1 of 1

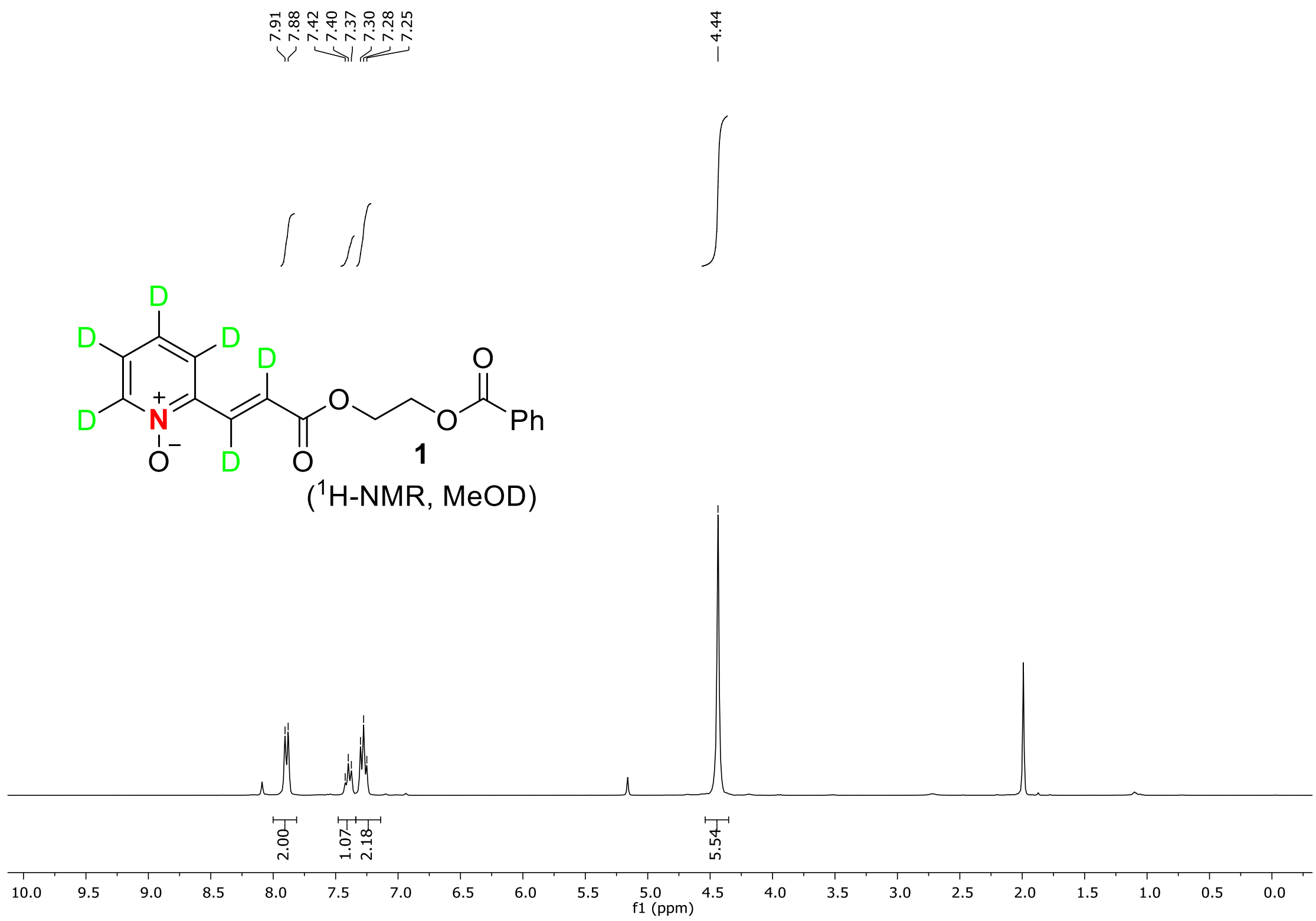
7.91
7.88
7.42
7.40
7.37
7.30
7.28
7.25
4.44
D
D
D
D
D
D
O
N
O
O
O
O
Ph
1
(^{1}H-NMR, MeOD)
2.00
1.07
2.18
5.54
10.0 9.5 9.0 8.5 8.0 7.5 7.0 6.5 6.0 5.5 5.0 4.5 4.0 3.5 3.0 2.5 2.0 1.5 1.0 0.5 0.0
f1 (ppm)

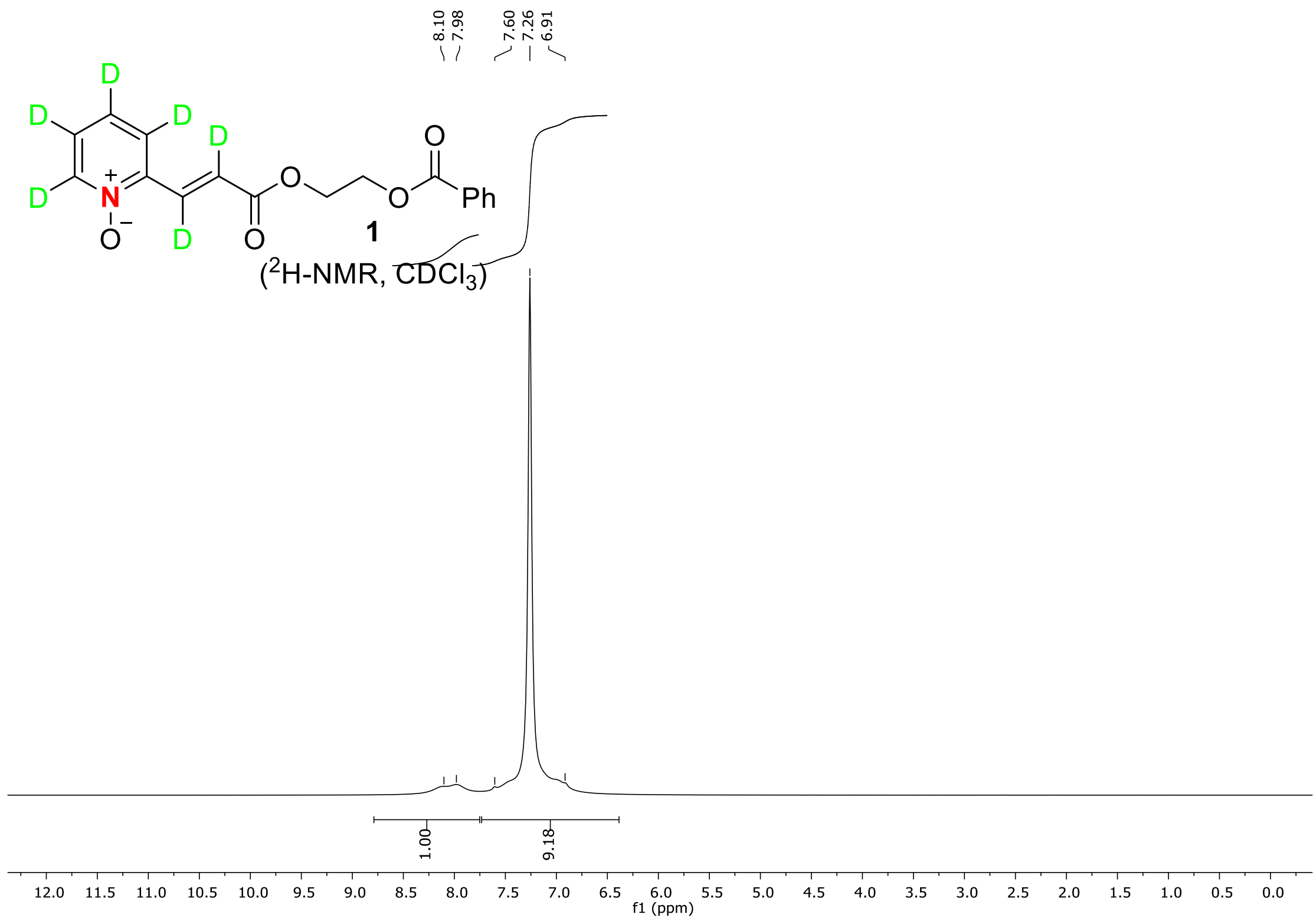
8.10
7.98
7.60
7.26
6.91
D
D
D
D
D
D
O
N
O
O
O
O
Ph
1
(^{2}H-NMR, $CDCl_3$)
1.00
9.18
12.0 11.5 11.0 10.5 10.0 9.5 9.0 8.5 8.0 7.5 7.0 6.5 6.0 5.5 5.0 4.5 4.0 3.5 3.0 2.5 2.0 1.5 1.0 0.5 0.0
f1 (ppm)

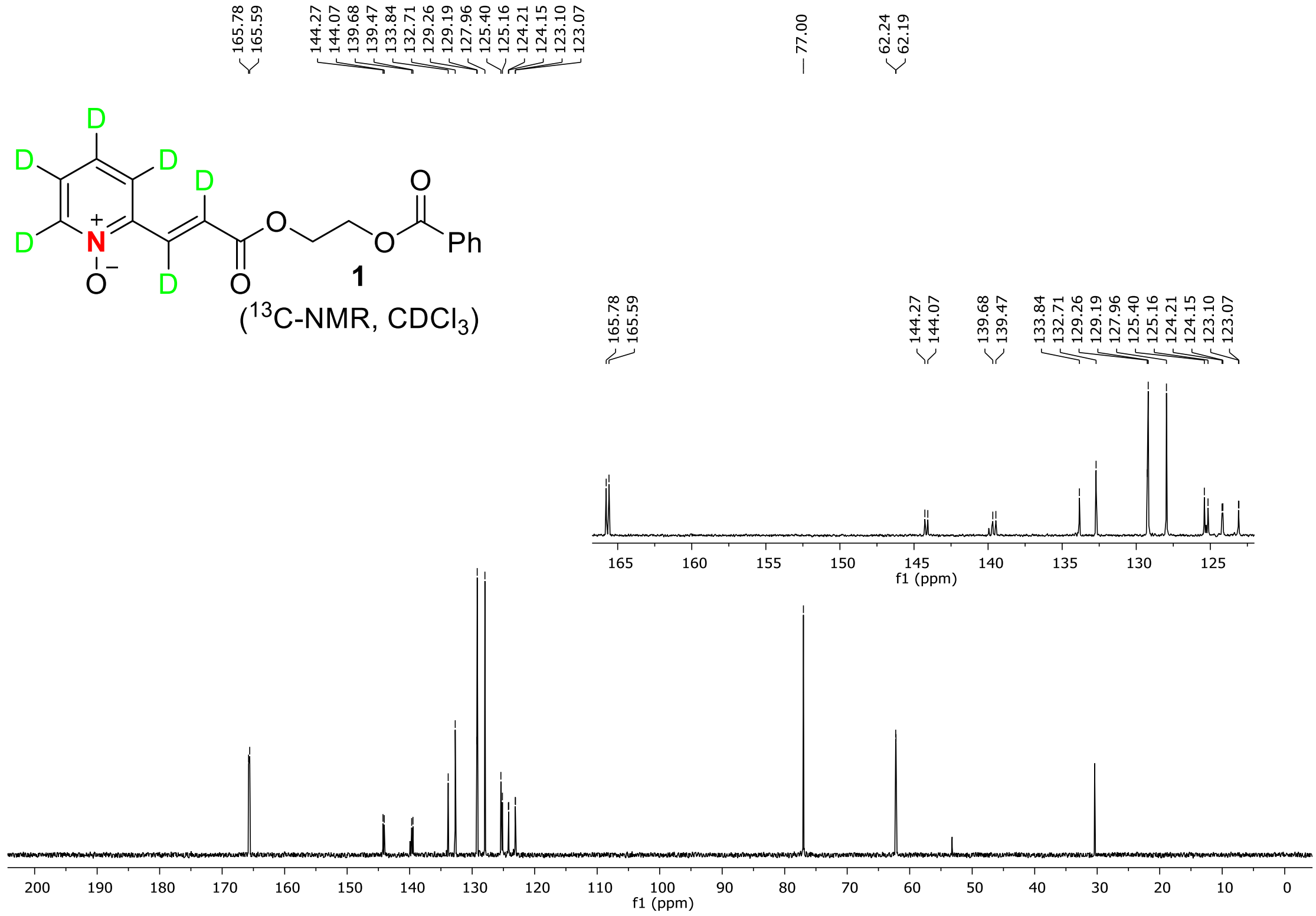

1
(^{13}C-NMR, $CDCl_3$)

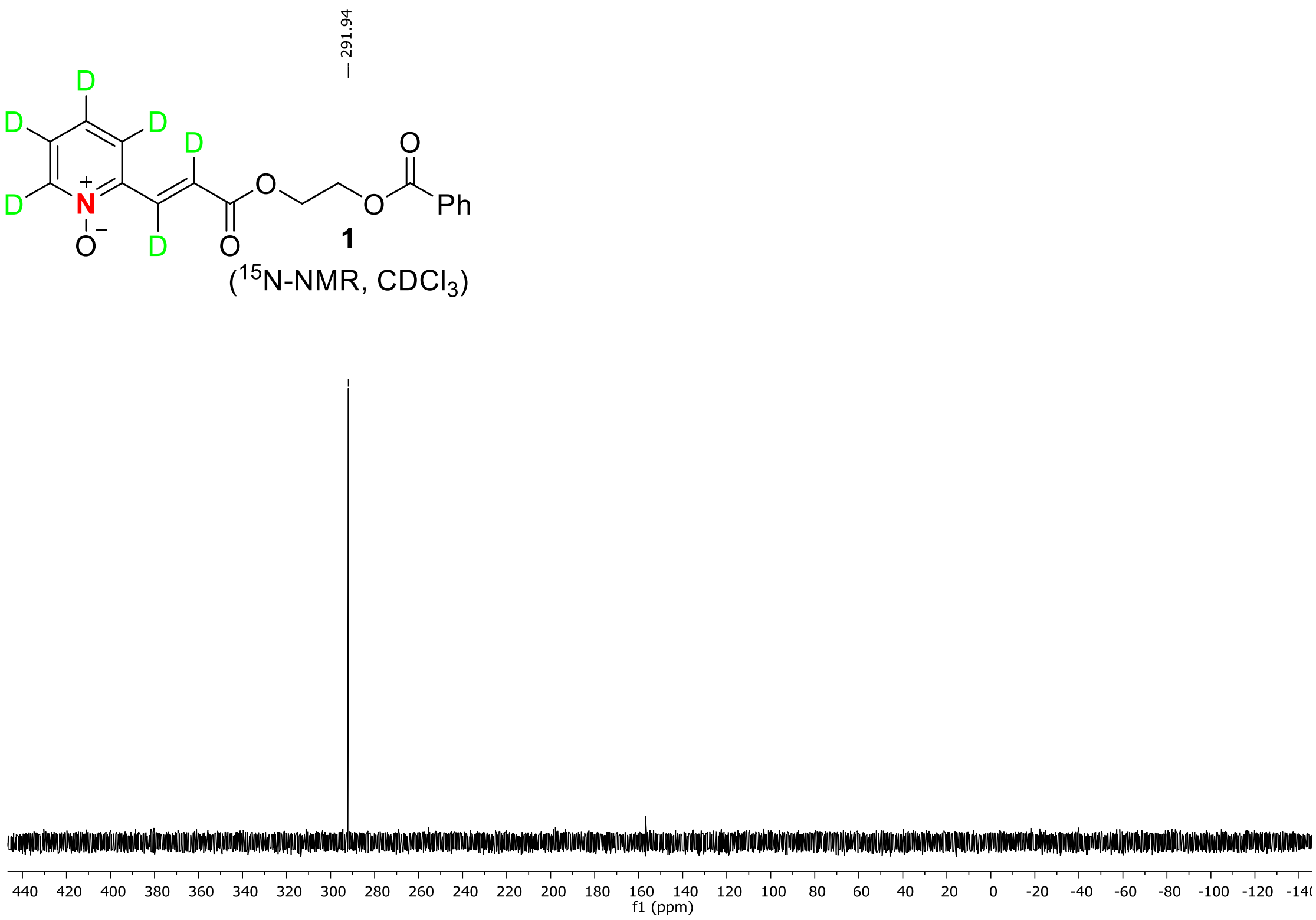

1
(^{15}N-NMR, $CDCl_3$)

Display Report

Analysis Info			
Analysis Name	Z:\Data\2024\2407\sam310724\yli00097_4_01_126752.d	Acquisition Date	31.07.2024 21:37:14
Method	hystar_pl.m	Operator	BDAL@DE
Sample Name	yli00097	Instrument / Ser#	micrOTOF 10237
Comment			

Acquisition Parameter

Source Type	ESI	Ion Polarity	Positive	Set Nebulizer	1.2 Bar
Focus	Not active			Set Dry Heater	180 °C
Scan Begin	50 m/z	Set Capillary	4500 V	Set Dry Gas	4.0 l/min
Scan End	1600 m/z	Set End Plate Offset	-500 V	Set Divert Valve	Source

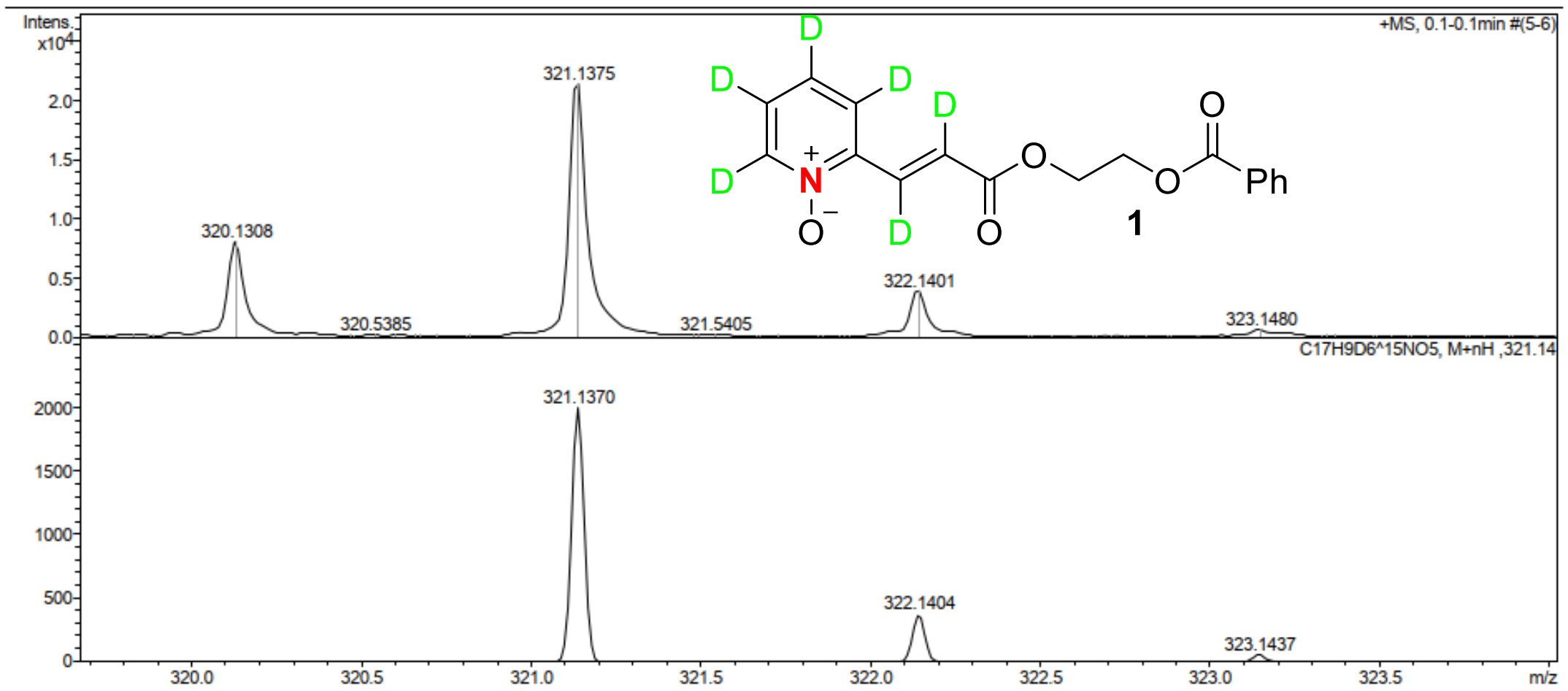

Bruker Compass DataAnalysis 4.0 printed: 31.07.2024 12:59:37 Page 1 of 1

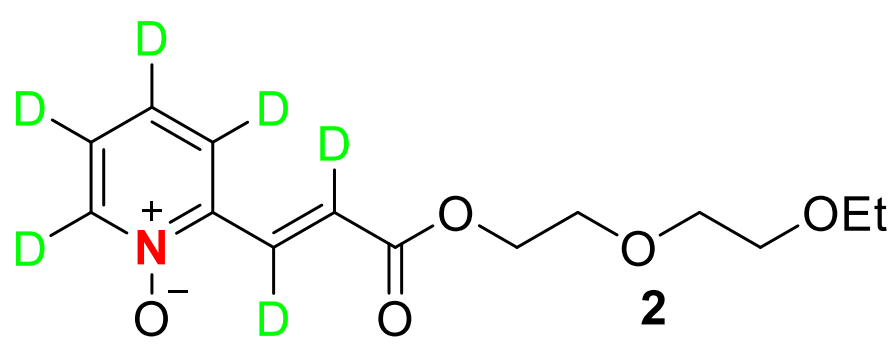

2

(^{1}H-NMR, $CDCl_3$)

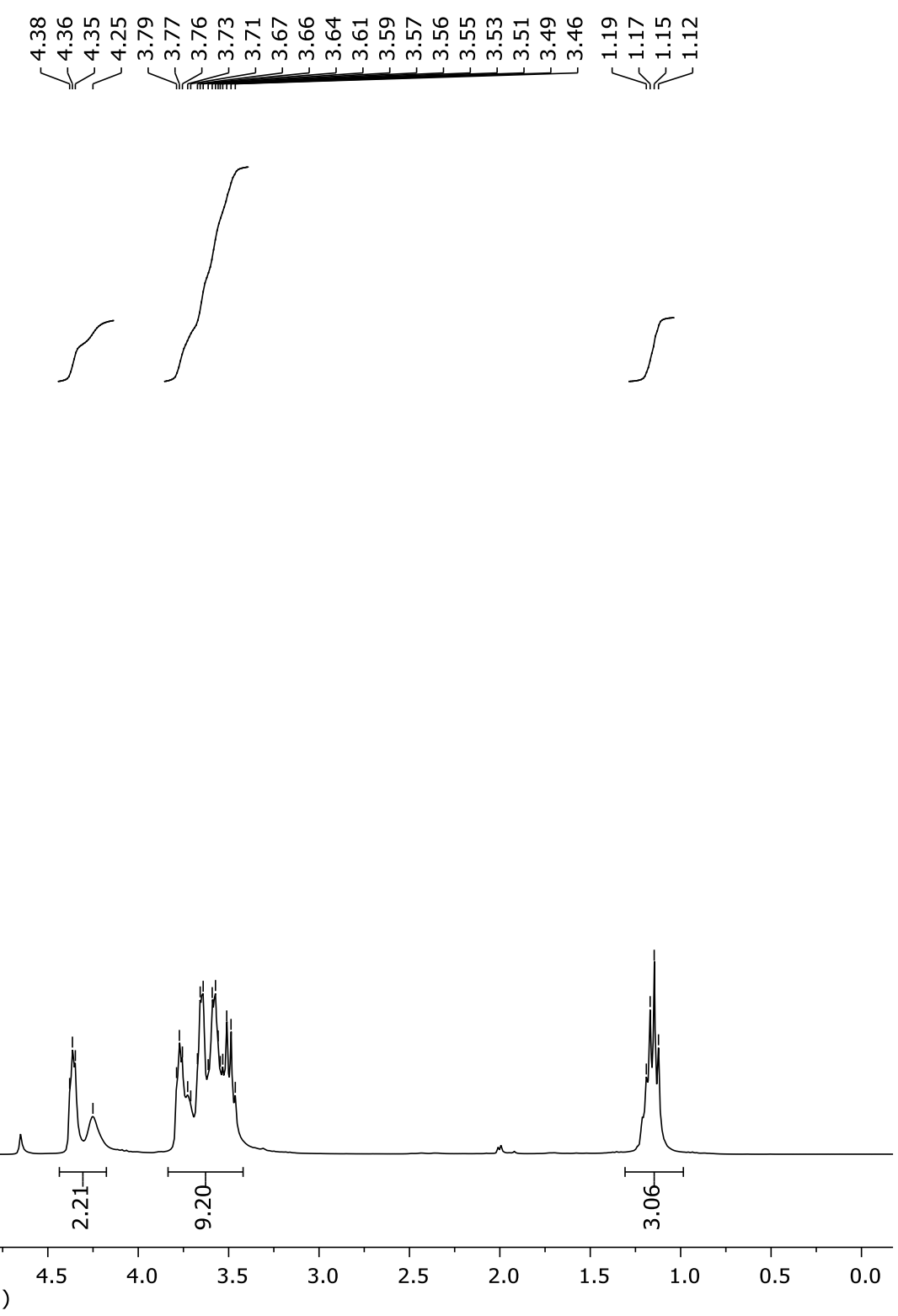

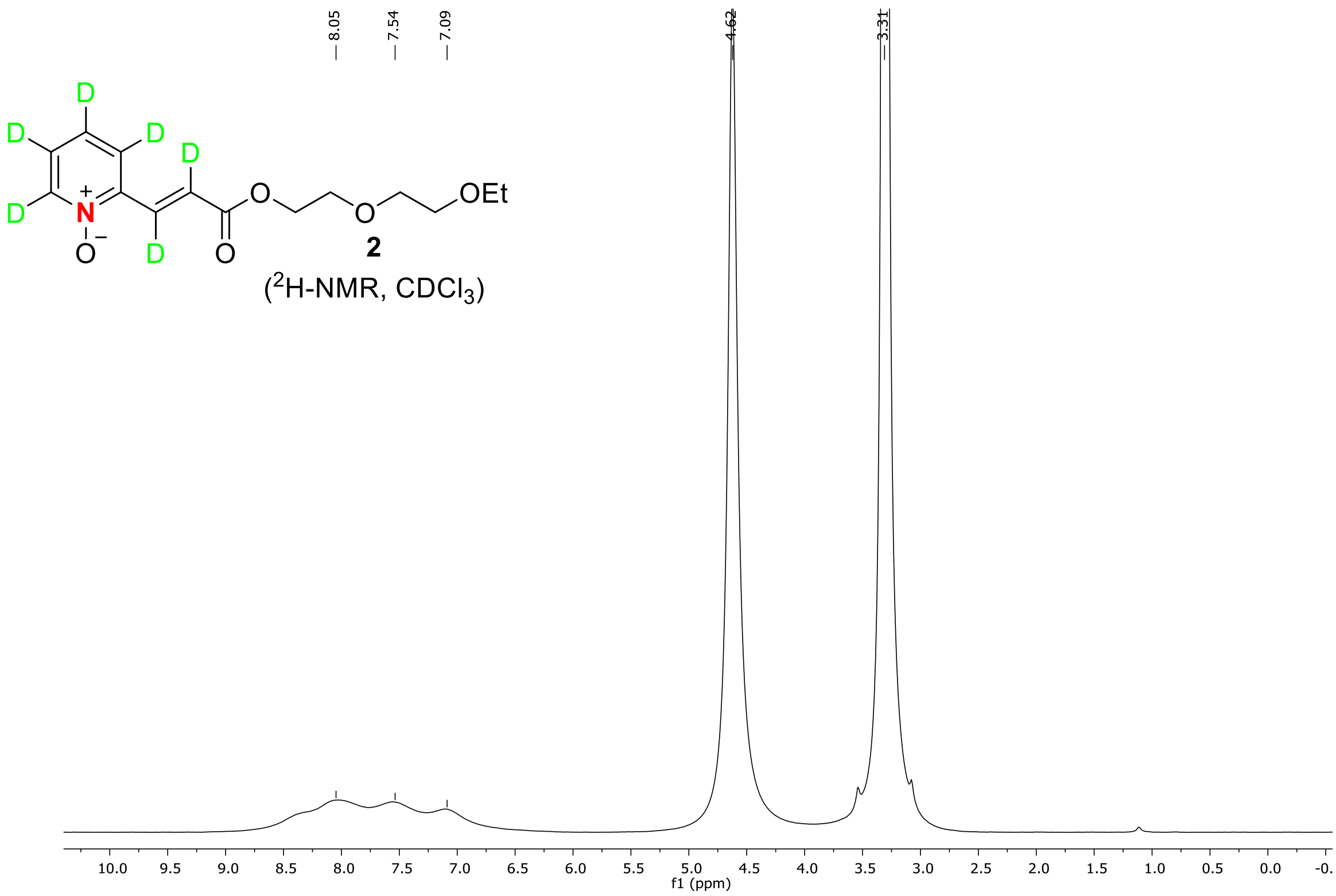

2

(^{2}H-NMR, $CDCl_3$)

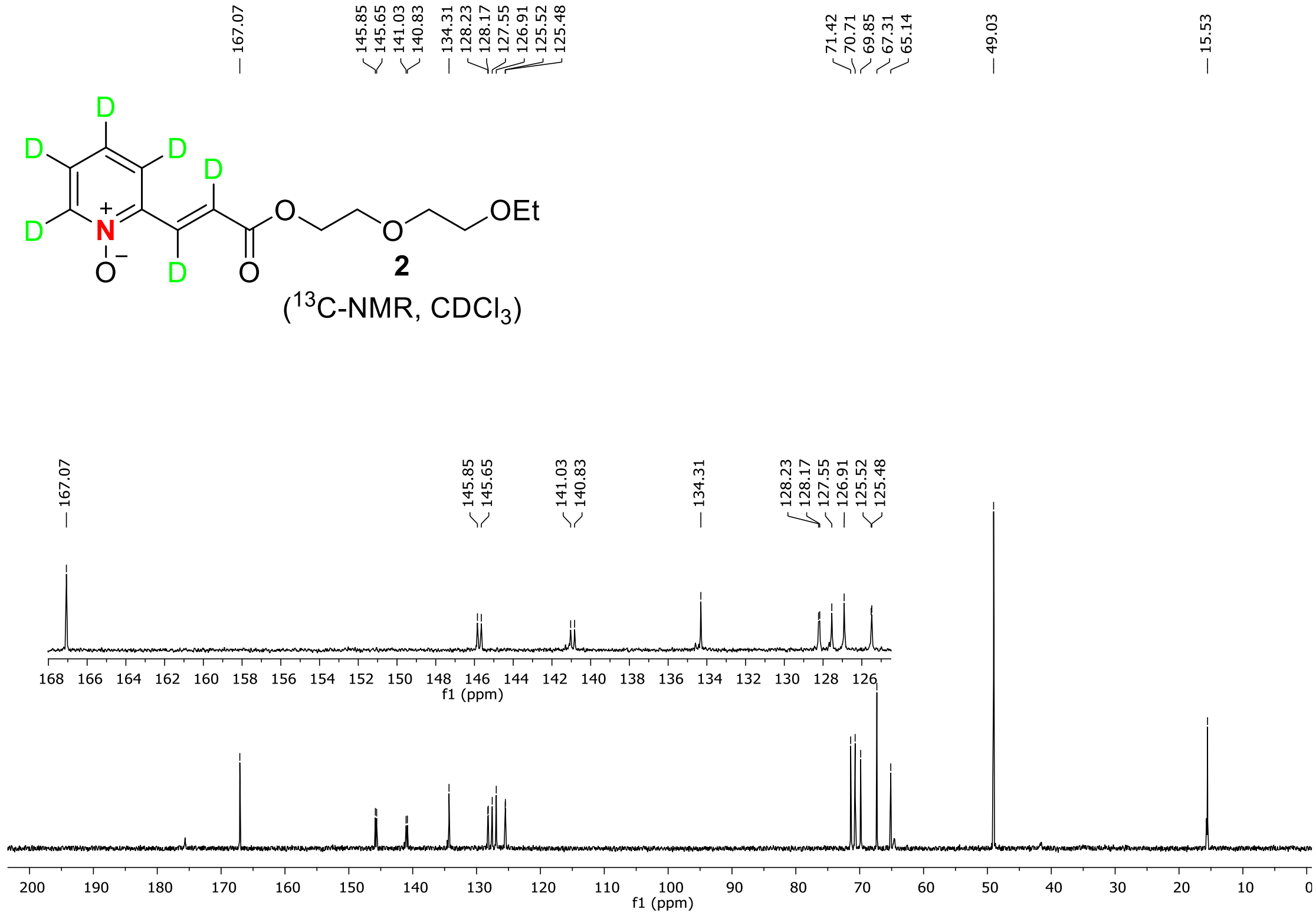

2

(^{13}C-NMR, $CDCl_3$)

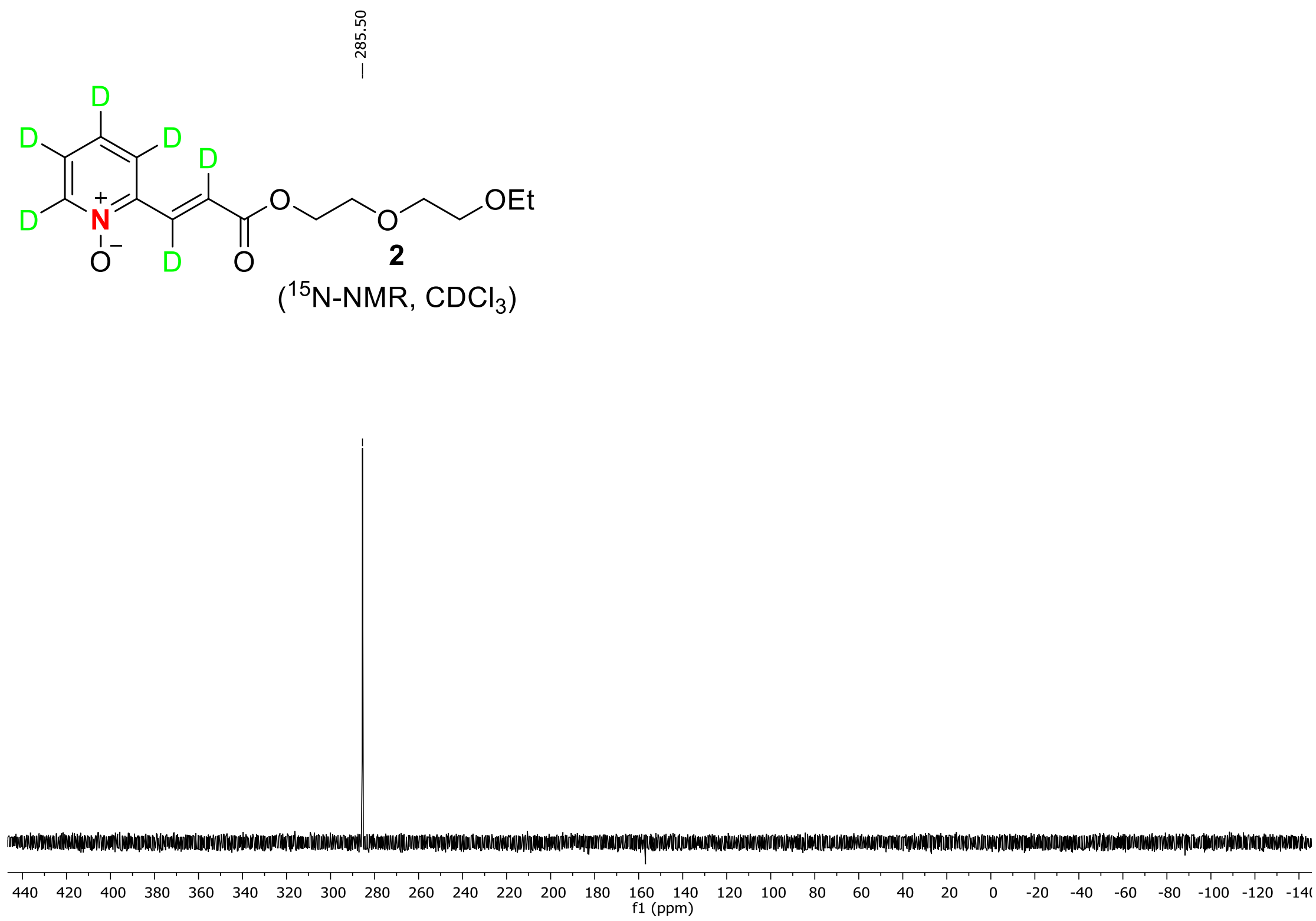

Display Report

Analysis Info

Analysis Name	Z:\Data\2024\2407\sam310724\yli00098_5_01_126753.d	Acquisition Date	31.07.2024 21:41:52
Method	hystar_pl.m	Operator	BDAL@DE
Sample Name	yli00098	Instrument / Ser#	micrOTOF 10237
Comment			

Acquisition Parameter

Source Type	ESI	Ion Polarity	Positive	Set Nebulizer	1.2 Bar
Focus	Not active			Set Dry Heater	180 °C
Scan Begin	50 m/z	Set Capillary	4500 V	Set Dry Gas	4.0 l/min
Scan End	1600 m/z	Set End Plate Offset	-500 V	Set Divert Valve	Source

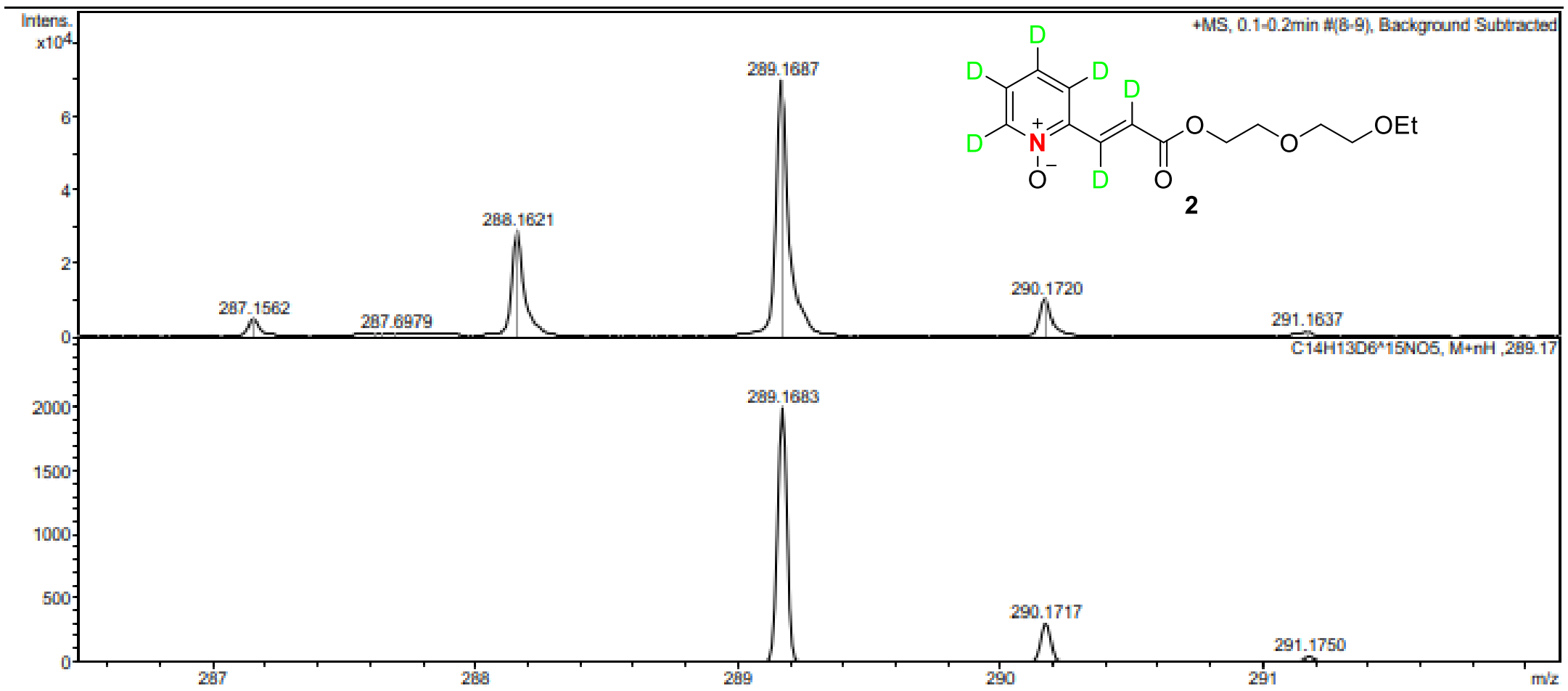

Bruker Compass DataAnalysis 4.0 printed: 31.07.2024 13:04:11 Page 1 of 1

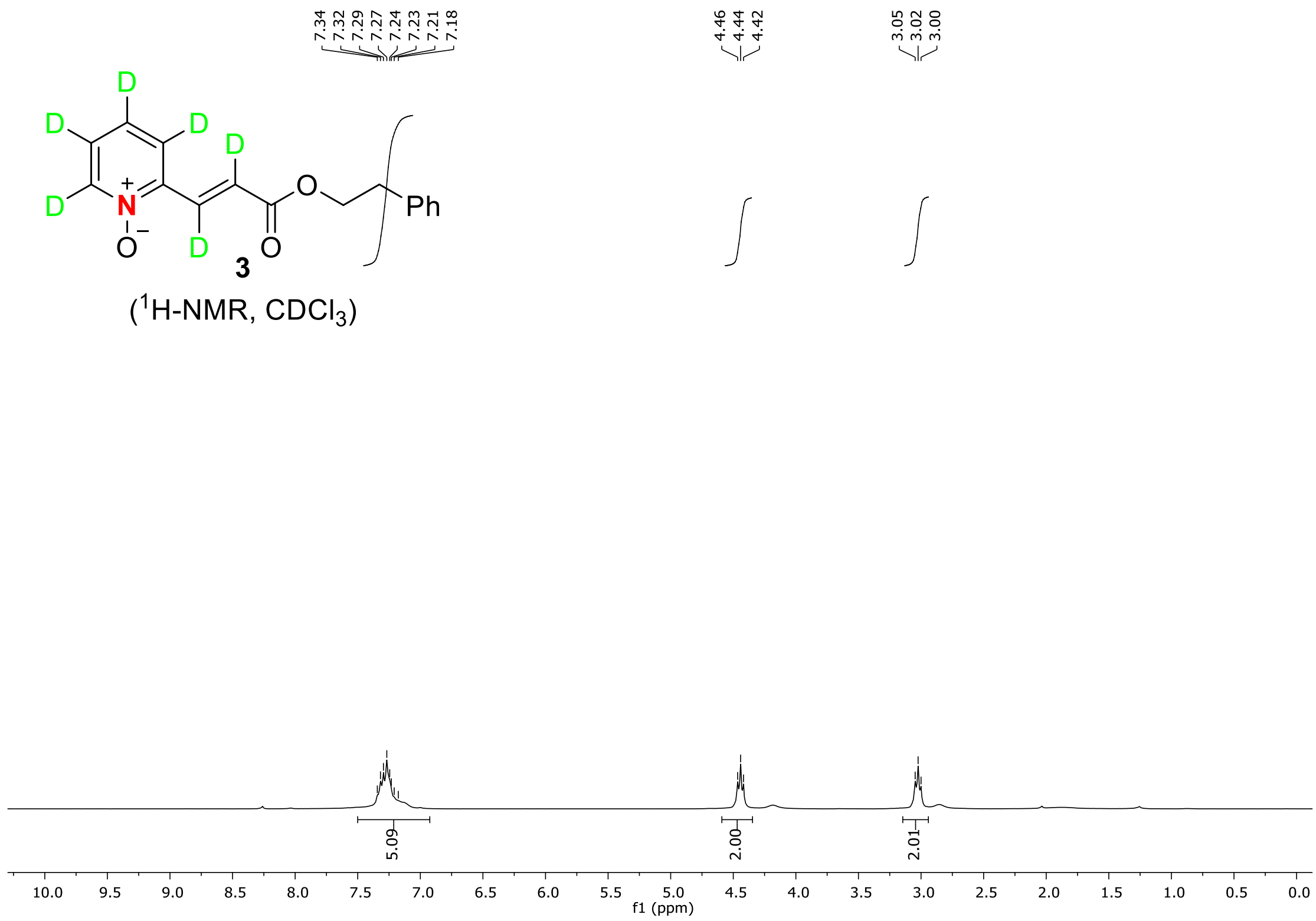

7.34
7.32
7.29
7.27
7.24
7.23
7.21
7.18
4.46
4.44
4.42
3.05
3.02
3.00
D
D
D
D
D
D
N
O
O
O
Ph
3
(1H-NMR, CDCl3)
5.09
2.00
2.01
10.0 9.5 9.0 8.5 8.0 7.5 7.0 6.5 6.0 5.5 5.0 4.5 4.0 3.5 3.0 2.5 2.0 1.5 1.0 0.5 0.0
f1 (ppm)

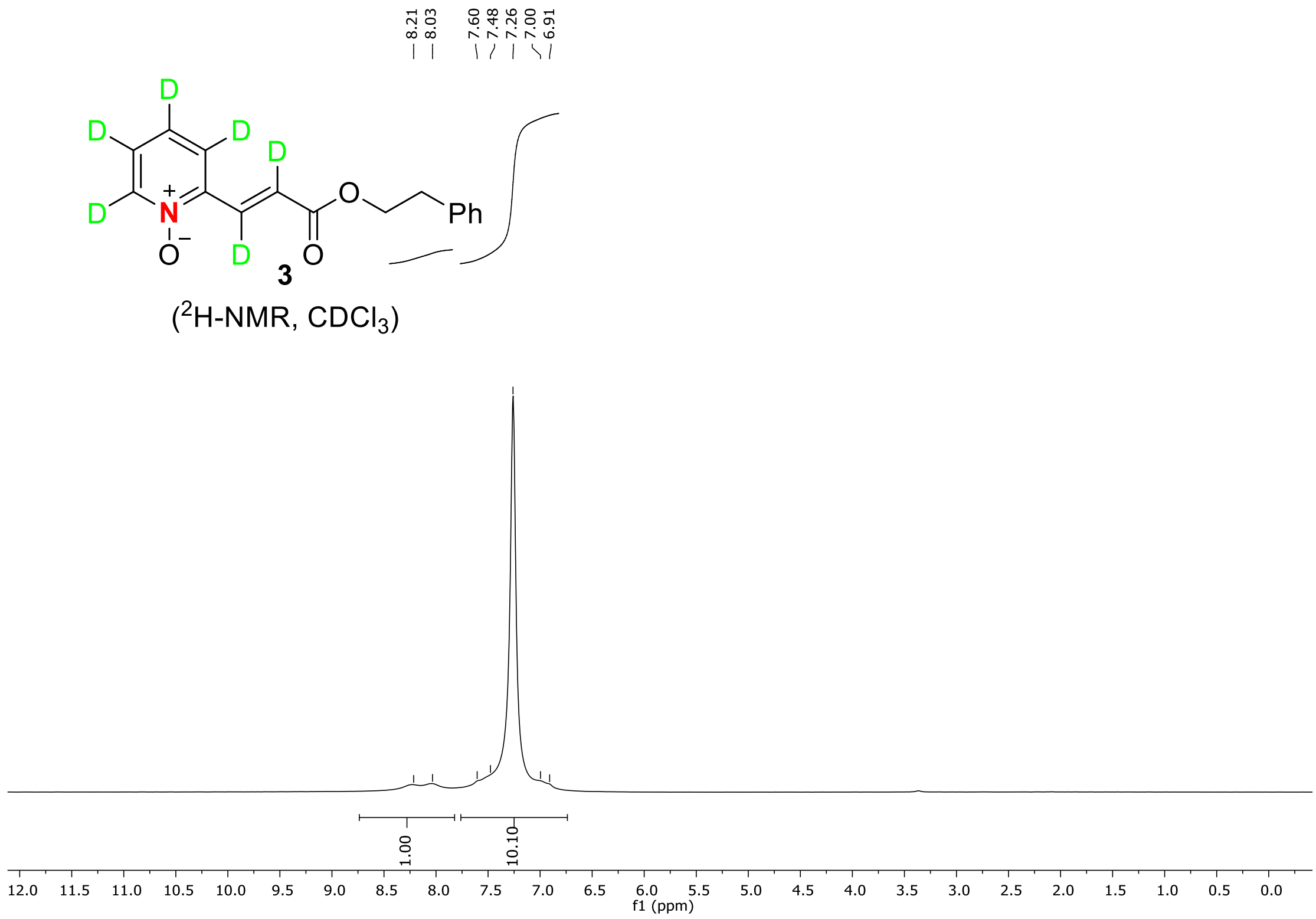

8.21
8.03
7.60
7.48
7.26
7.00
6.91
D
D
D
D
D
D
N
O
O
O
Ph
3
(2H-NMR, CDCl3)
1.00
10.10
12.0 11.5 11.0 10.5 10.0 9.5 9.0 8.5 8.0 7.5 7.0 6.5 6.0 5.5 5.0 4.5 4.0 3.5 3.0 2.5 2.0 1.5 1.0 0.5 0.0
f1 (ppm)

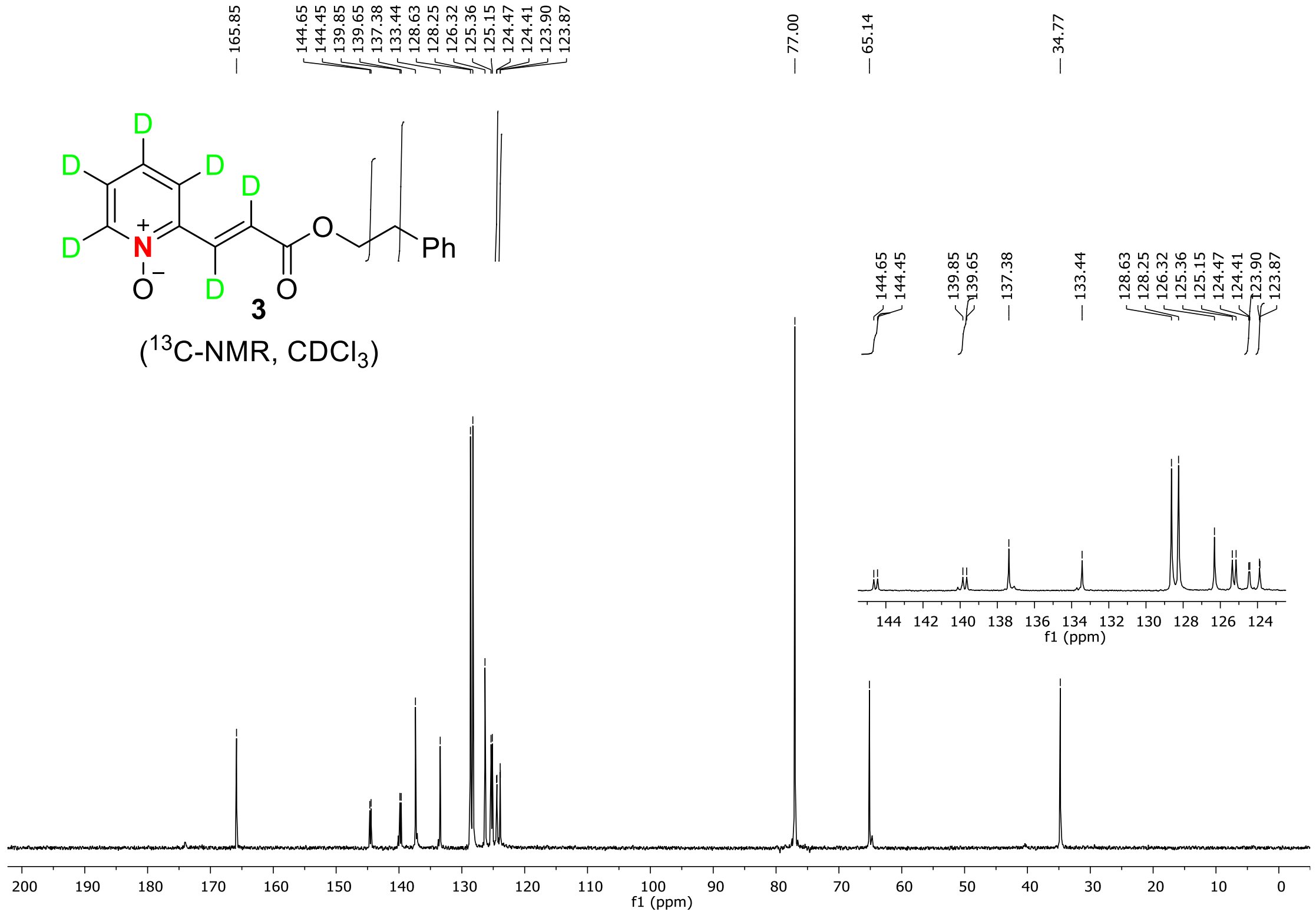
165.85
144.65
144.45
139.85
139.65
137.38
133.44
128.63
128.25
126.32
125.36
125.15
124.47
124.41
123.90
123.87
77.00
65.14
34.77
D
D
D
D
D
D
Ph
3
(^{13}C-NMR, $CDCl_3$)
144 142 140 138 136 134 132 130 128 126 124
f1 (ppm)
200 190 180 170 160 150 140 130 120 110 100 90 80 70 60 50 40 30 20 10 0
f1 (ppm)

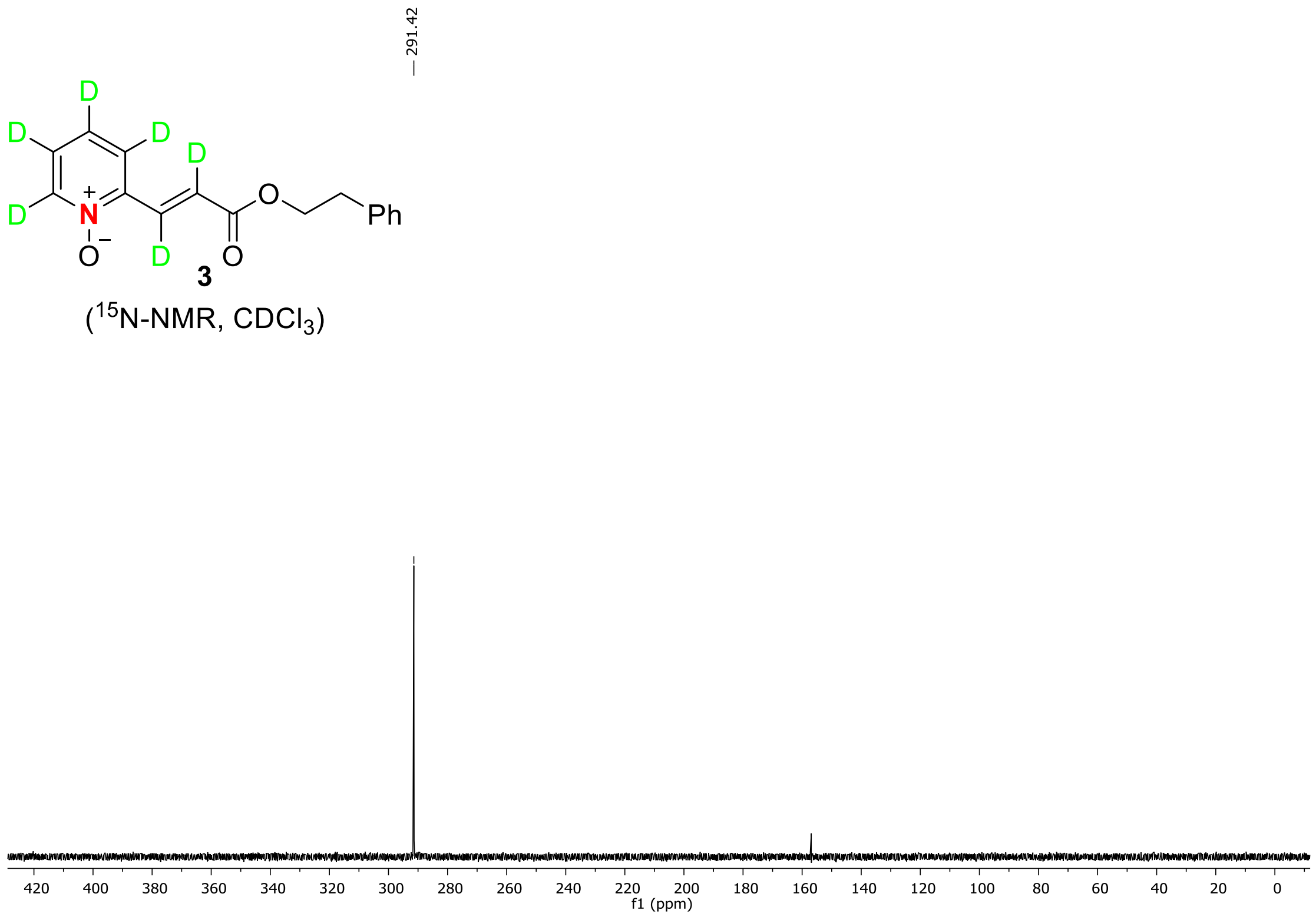
291.42
D
D
D
D
D
D
Ph
3
(^{15}N-NMR, $CDCl_3$)
420 400 380 360 340 320 300 280 260 240 220 200 180 160 140 120 100 80 60 40 20 0
f1 (ppm)

Display Report

Analysis Info

Analysis Name	Z:\Data\2024\2407\sam310724\yli00096_6_01_126754.d	Acquisition Date	31.07.2024 21:46:30
Method	hystar_pl.m	Operator	BDAL@DE
Sample Name	yli00096	Instrument / Ser#	micrOTOF 10237
Comment			

Acquisition Parameter

Source Type	ESI	Ion Polarity	Positive	Set Nebulizer	1.2 Bar
Focus	Not active			Set Dry Heater	180 °C
Scan Begin	50 m/z	Set Capillary	4500 V	Set Dry Gas	4.0 l/min
Scan End	1600 m/z	Set End Plate Offset	-500 V	Set Divert Valve	Source

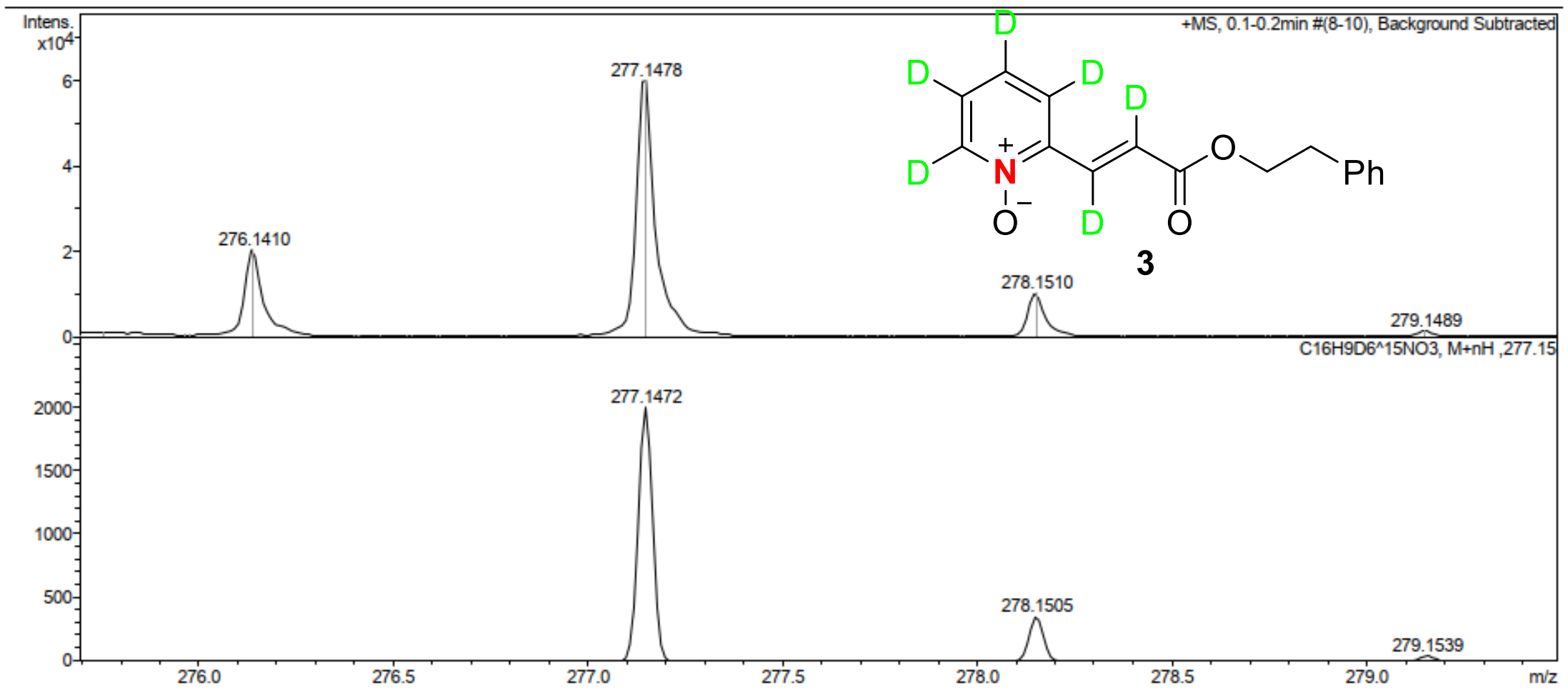

Bruker Compass DataAnalysis 4.0 printed: 31.07.2024 12:52:58 Page 1 of 1

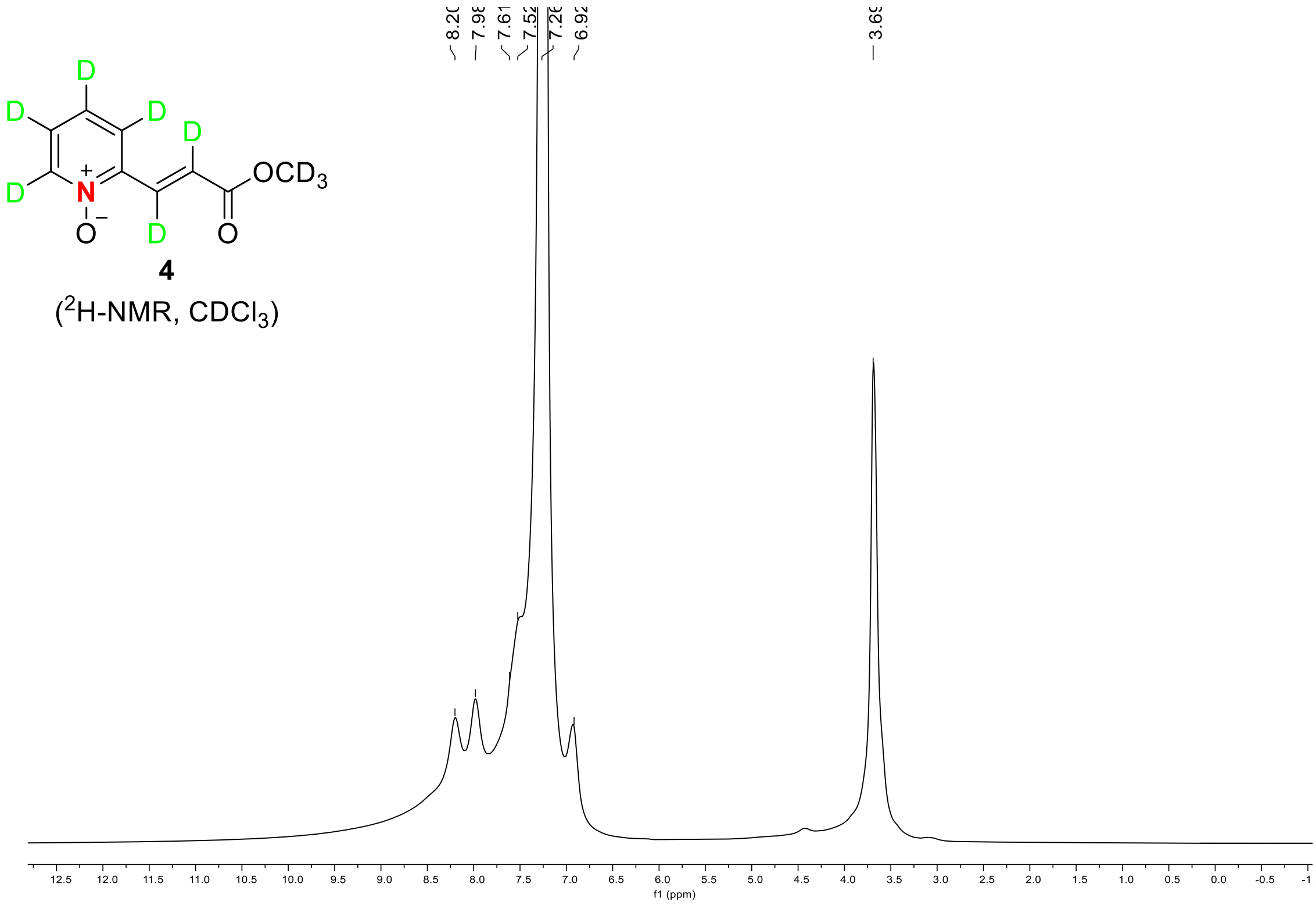

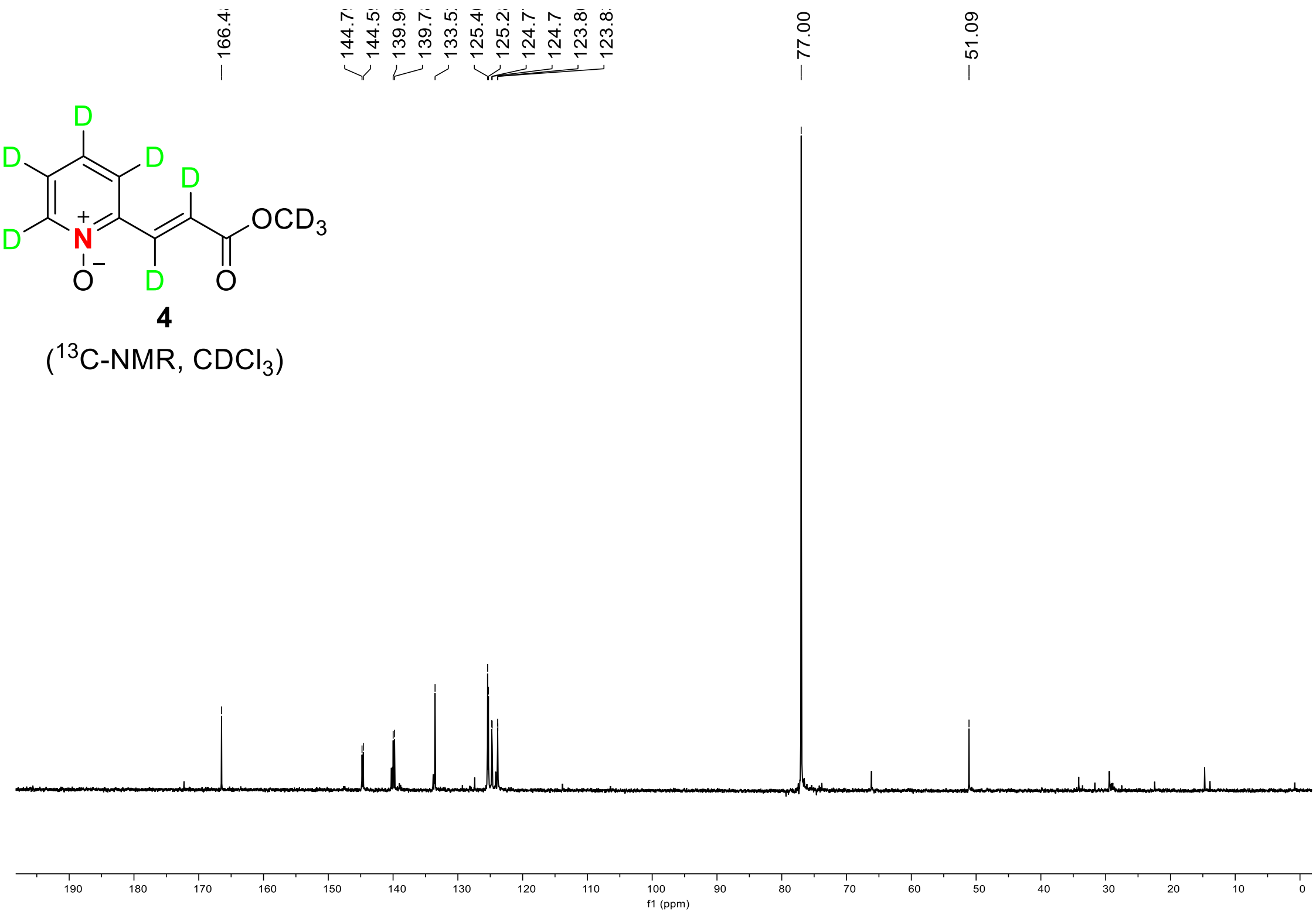
166.4
144.7
144.5
139.9
139.7
133.5
125.4
125.2
124.7
124.7
123.8
123.8
77.00
51.09
D
D
D
D
D
D
OCD3
N
O
O
4
(13C-NMR, CDCl3)
f1 (ppm)

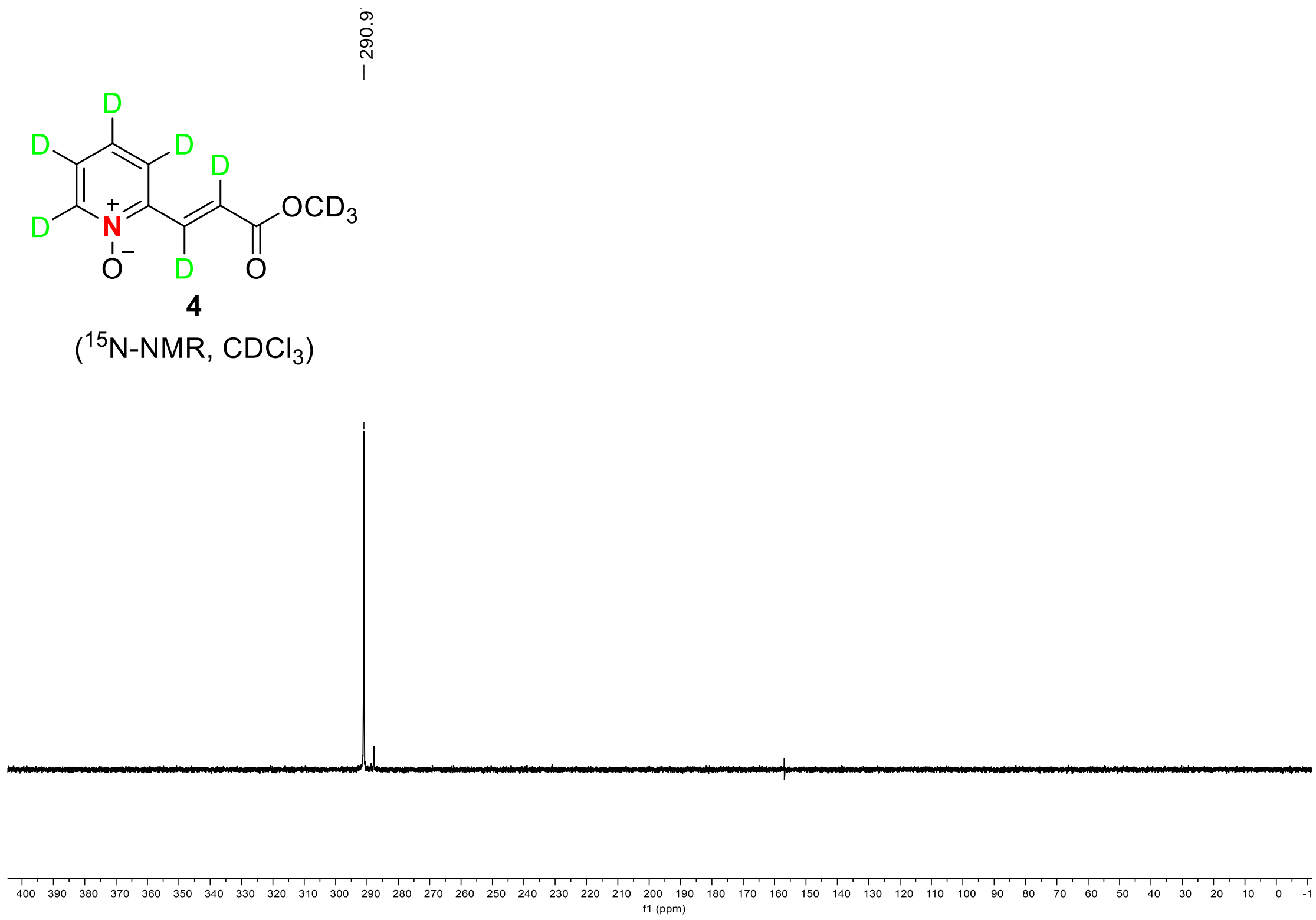
290.9
D
D
D
D
D
D
OCD3
N
O
O
4
(15N-NMR, CDCl3)
f1 (ppm)

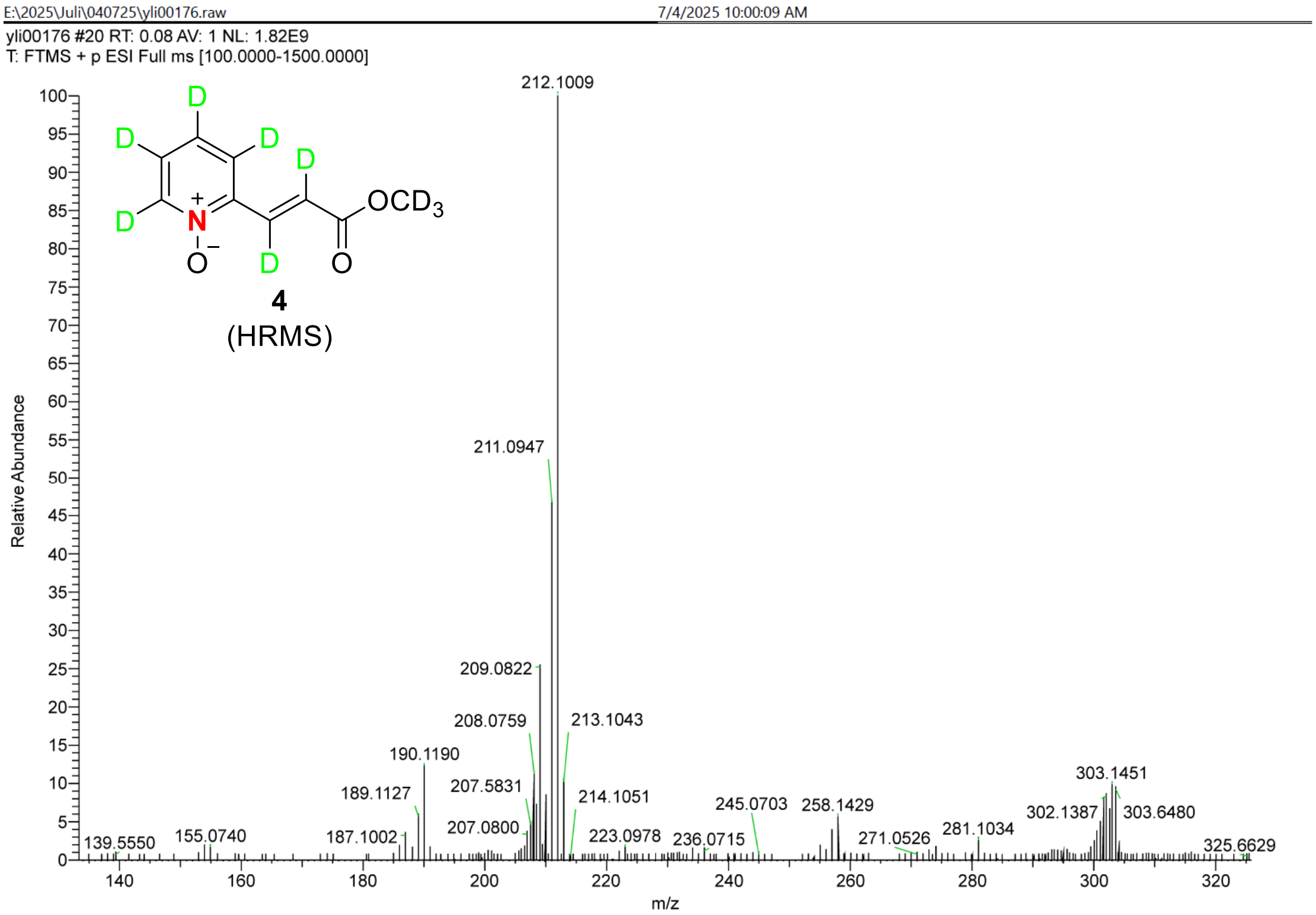

4
(HRMS)

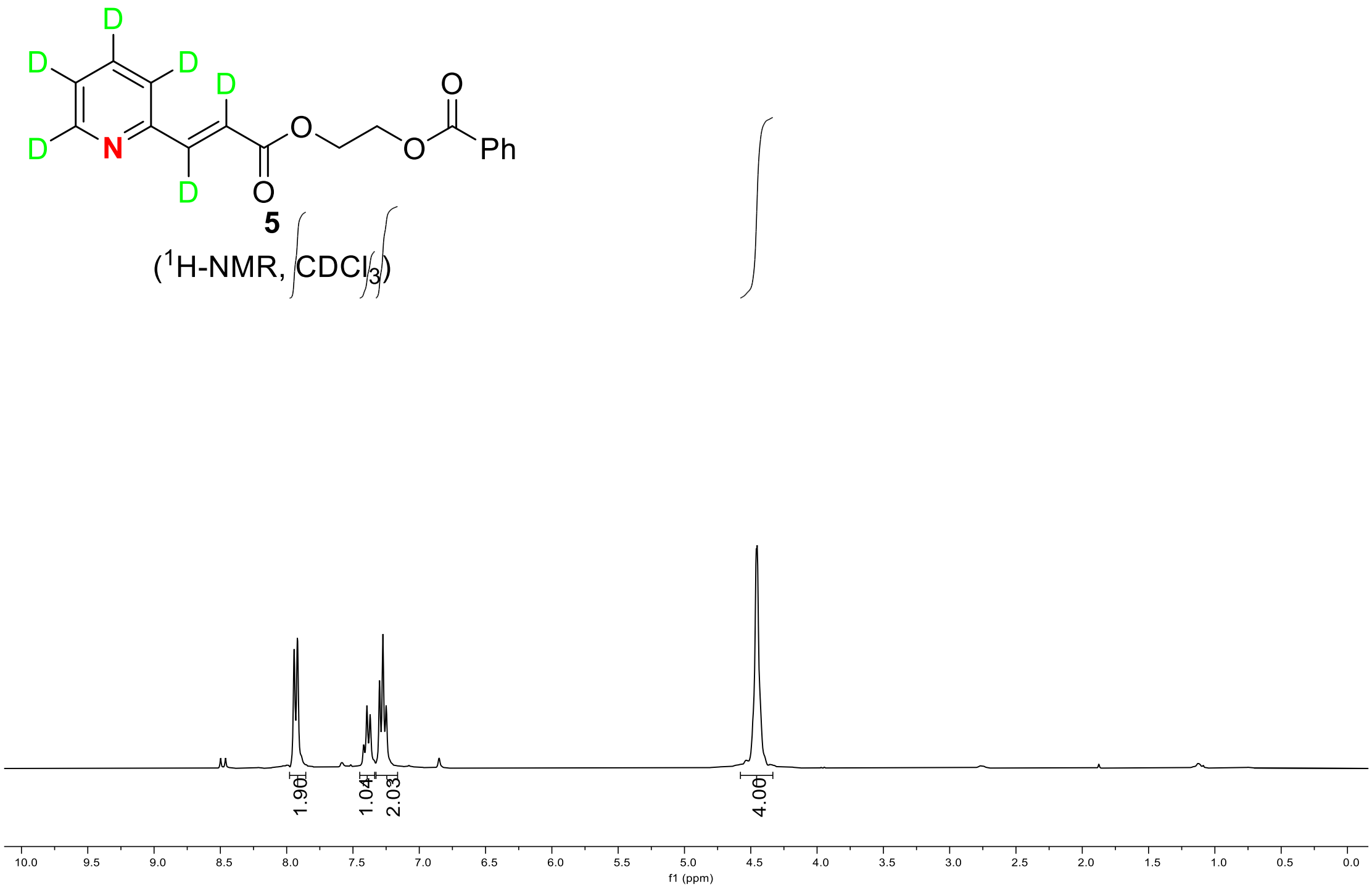

5
(^{1}H-NMR, $CDCl_3$)

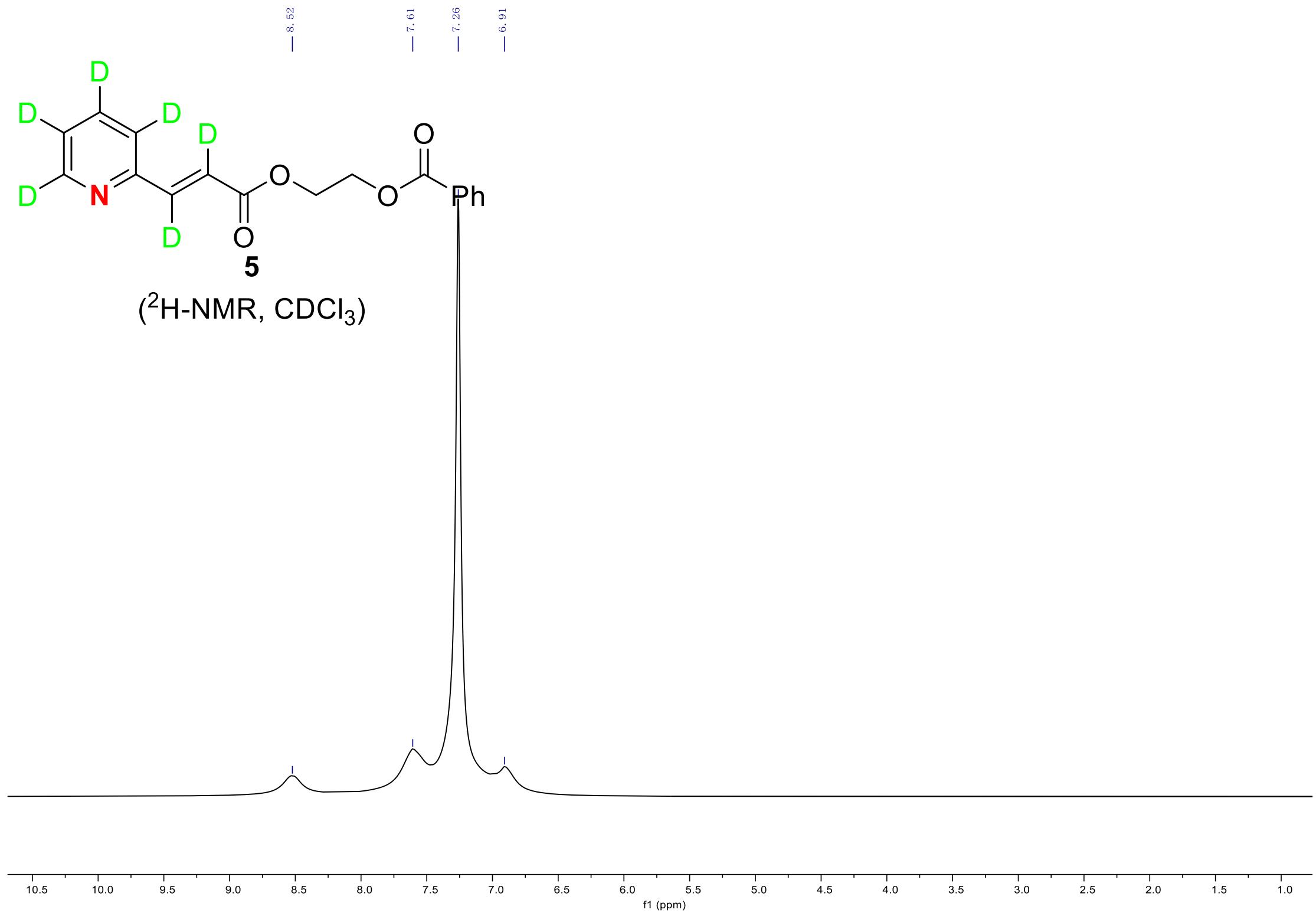
8.52
7.61
7.26
6.91
5
(^{2}H-NMR, CDCl$_3$)
f1 (ppm)

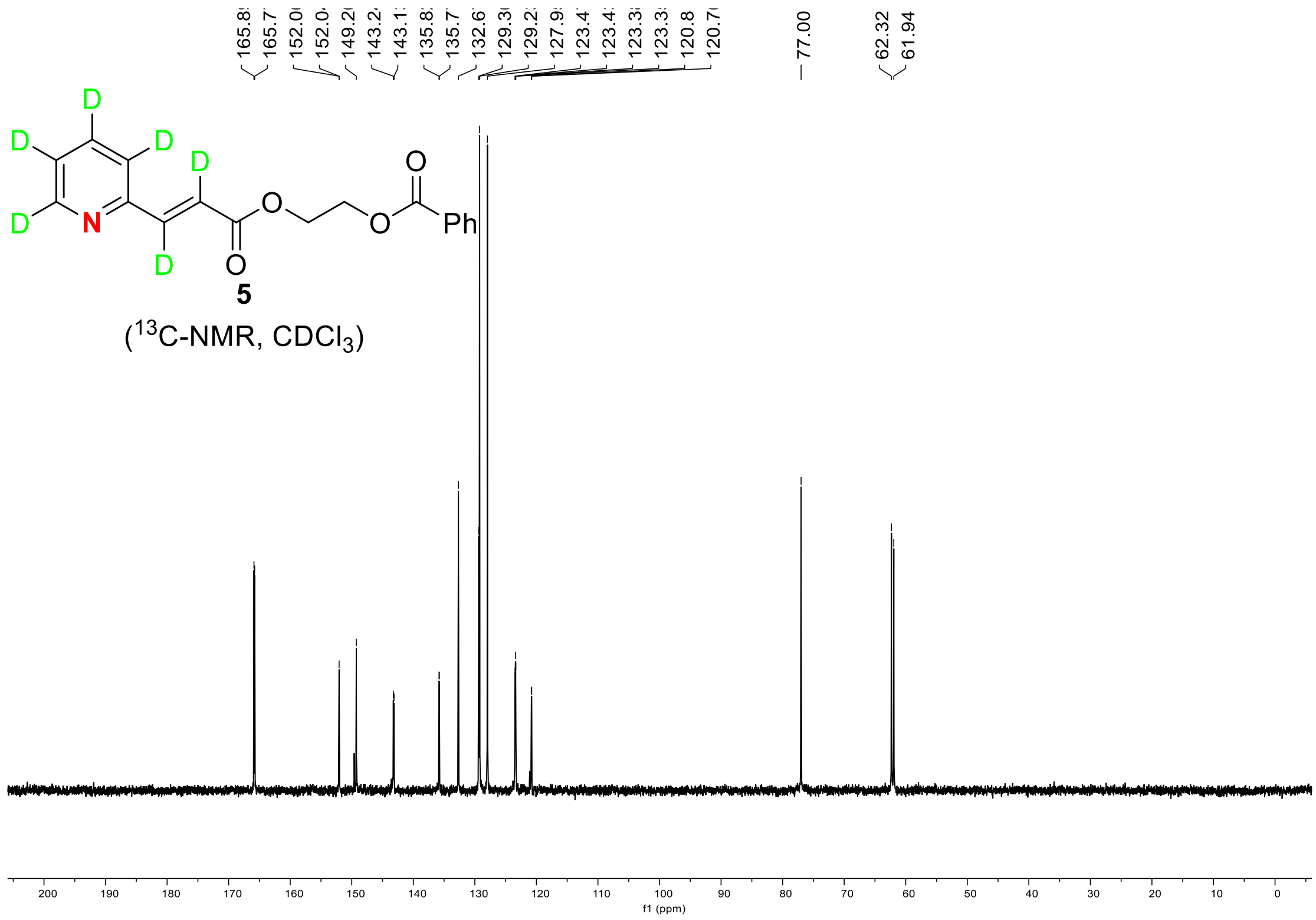
165.8
165.7
152.0
152.0
149.2
143.2
143.1
135.8
135.7
132.6
129.3
129.2
127.9
123.4
123.4
123.3
123.3
120.8
120.7
77.00
62.32
61.94
5
(^{13}C-NMR, CDCl$_3$)
f1 (ppm)

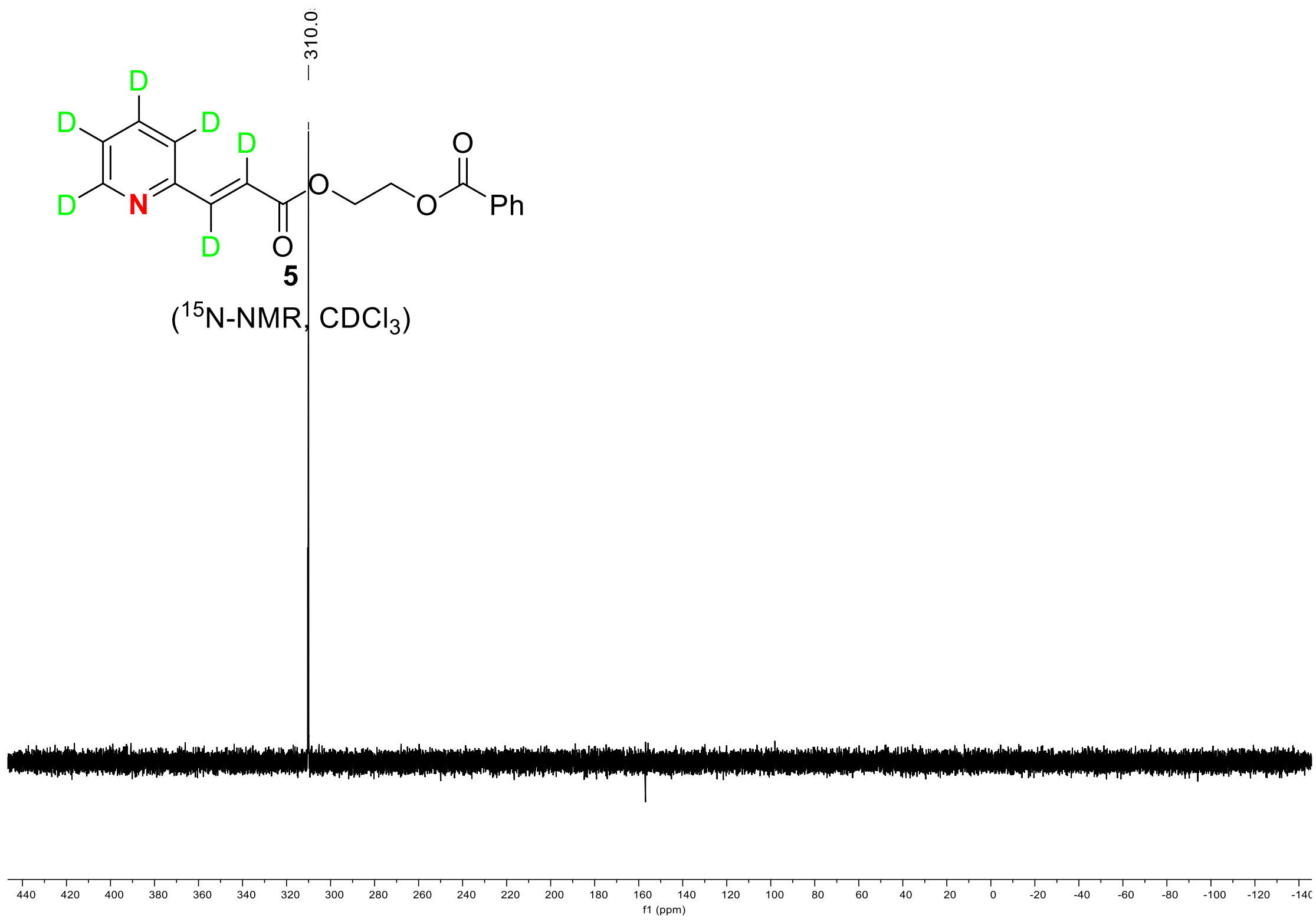
310.0
D
D
D
D
D
D
N
O
O
O
O
Ph
5
(^{15}N-NMR, CDCl$_3$)
f1 (ppm)

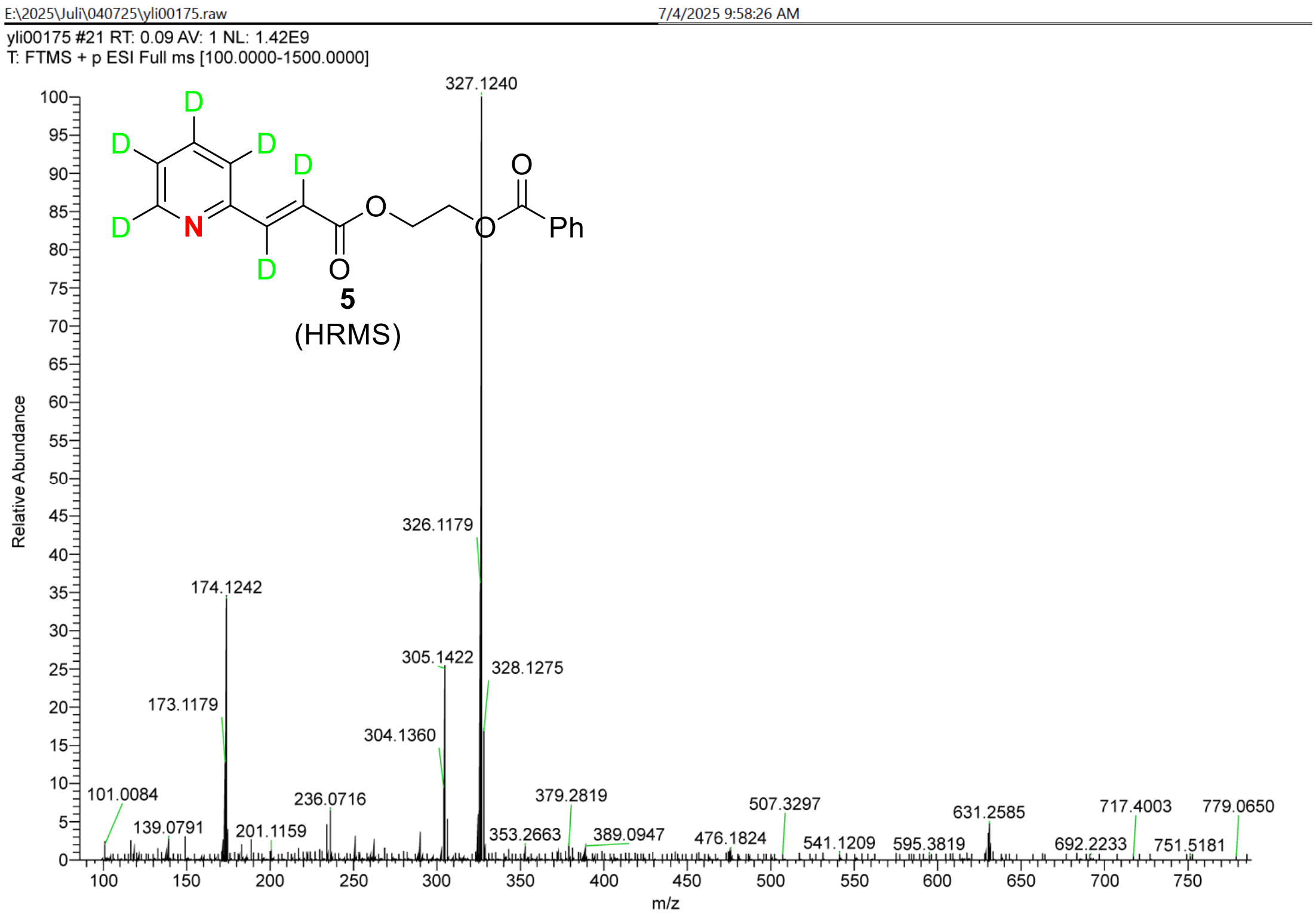
E:\2025\Juli\040725\yli00175.raw
7/4/2025 9:58:26 AM
yli00175 #21 RT: 0.09 AV: 1 NL: 1.42E9
T: FTMS + p ESI Full ms [100.0000-1500.0000]
327.1240
326.1179
174.1242
305.1422
328.1275
173.1179
304.1360
101.0084
236.0716
379.2819
507.3297
631.2585
717.4003
779.0650
139.0791
201.1159
353.2663
389.0947
476.1824
541.1209
595.3819
692.2233
751.5181
5
(HRMS)
Relative Abundance
m/z

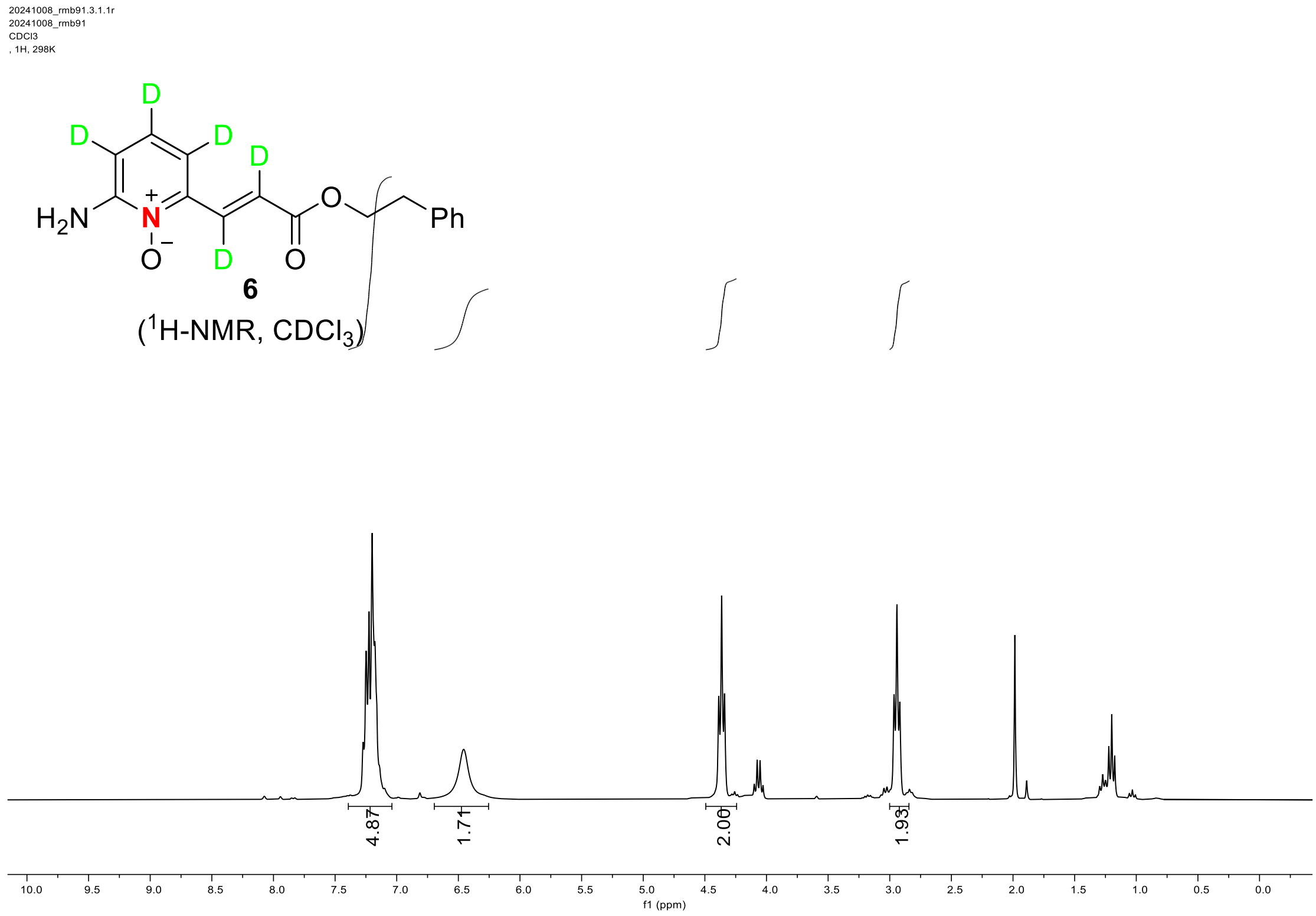
20241008_rmb91.3.1.1r
20241008_rmb91
CDCl3
, 1H, 298K
H2N
D
Ph
6
(1H-NMR, CDCl3)
4.87
1.71
2.00
1.93
f1 (ppm)

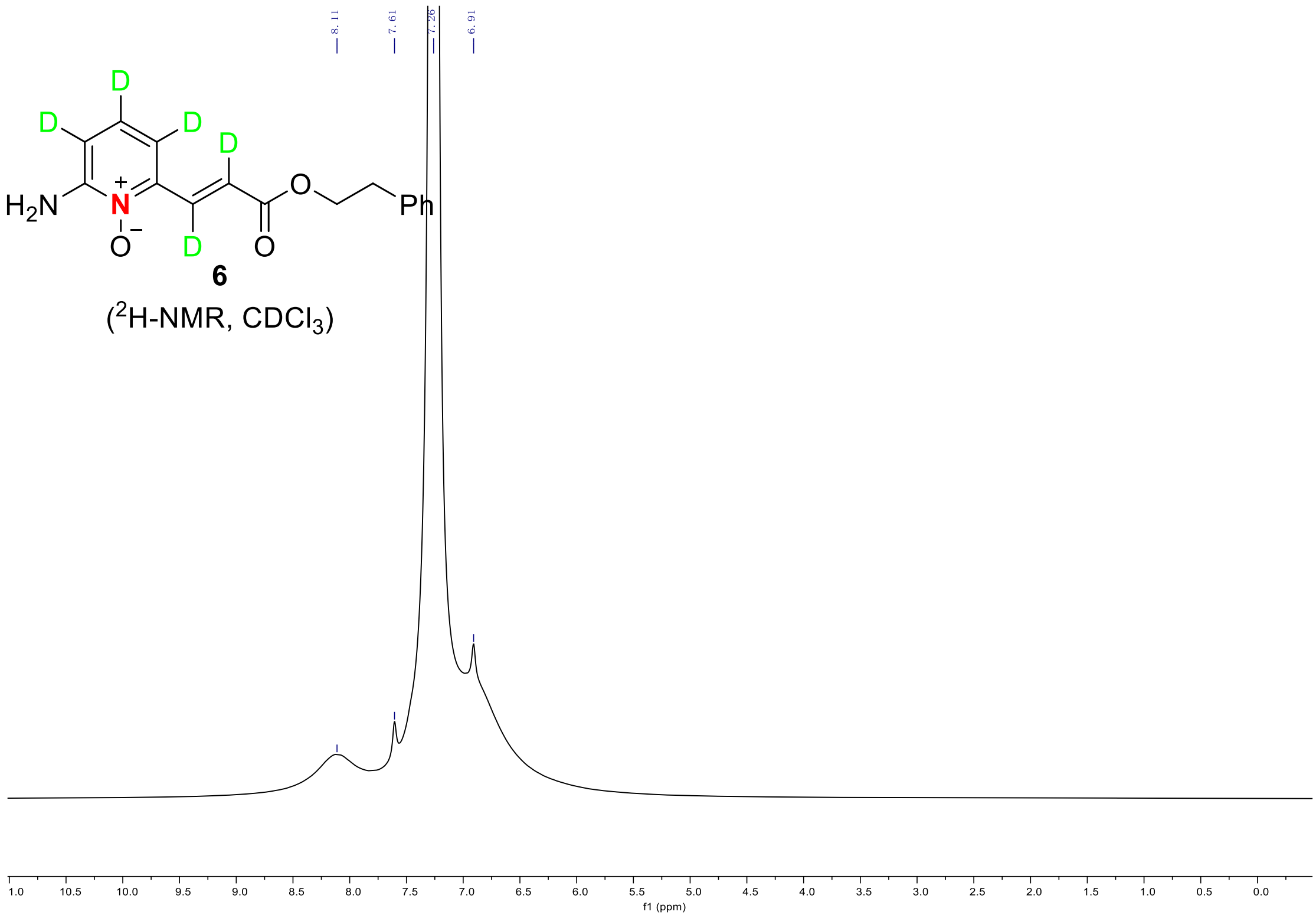
8.11
7.61
7.26
6.91
H2N
D
Ph
6
(2H-NMR, CDCl3)
f1 (ppm)

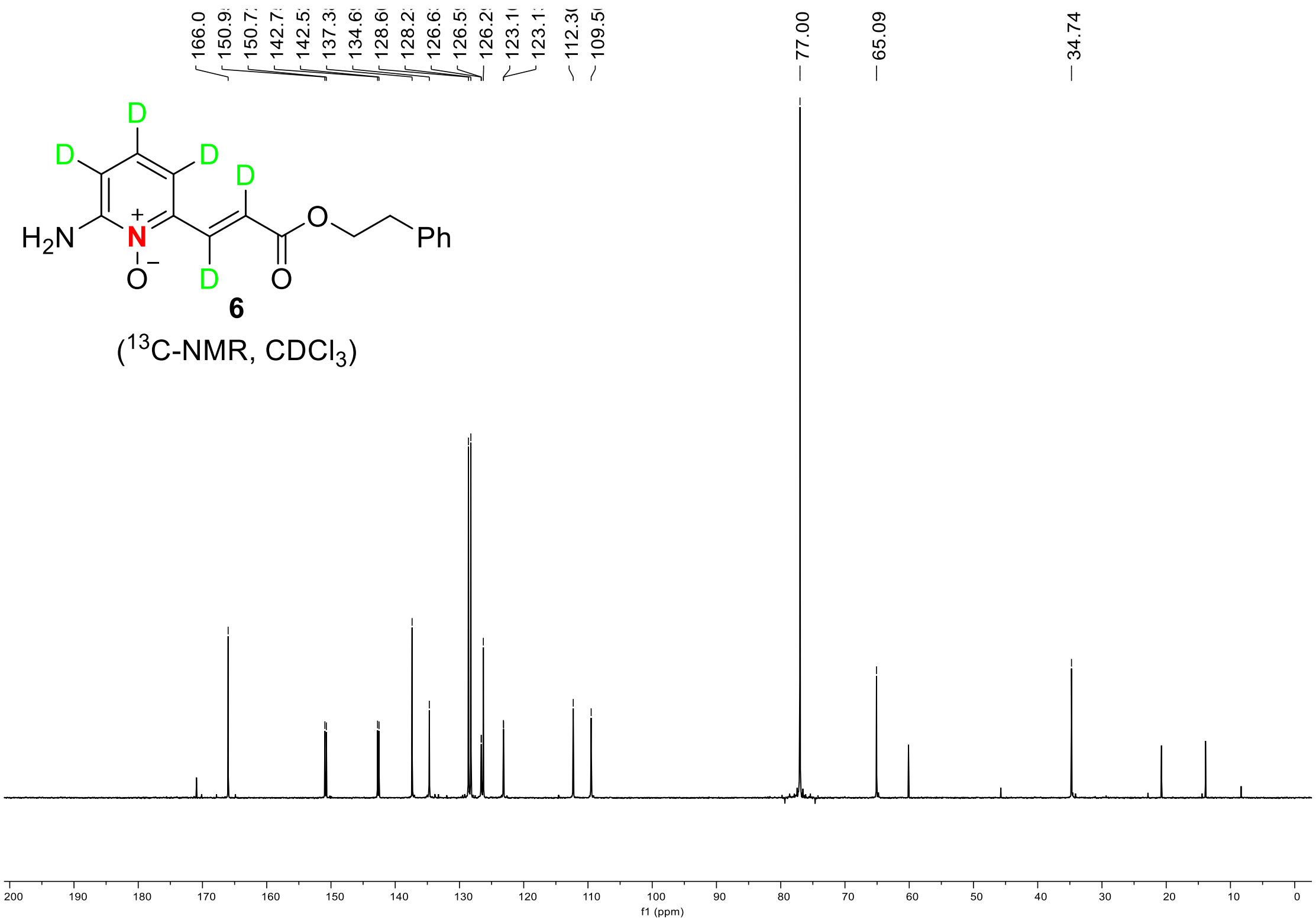

6

(^{13}C-NMR, $CDCl_3$)

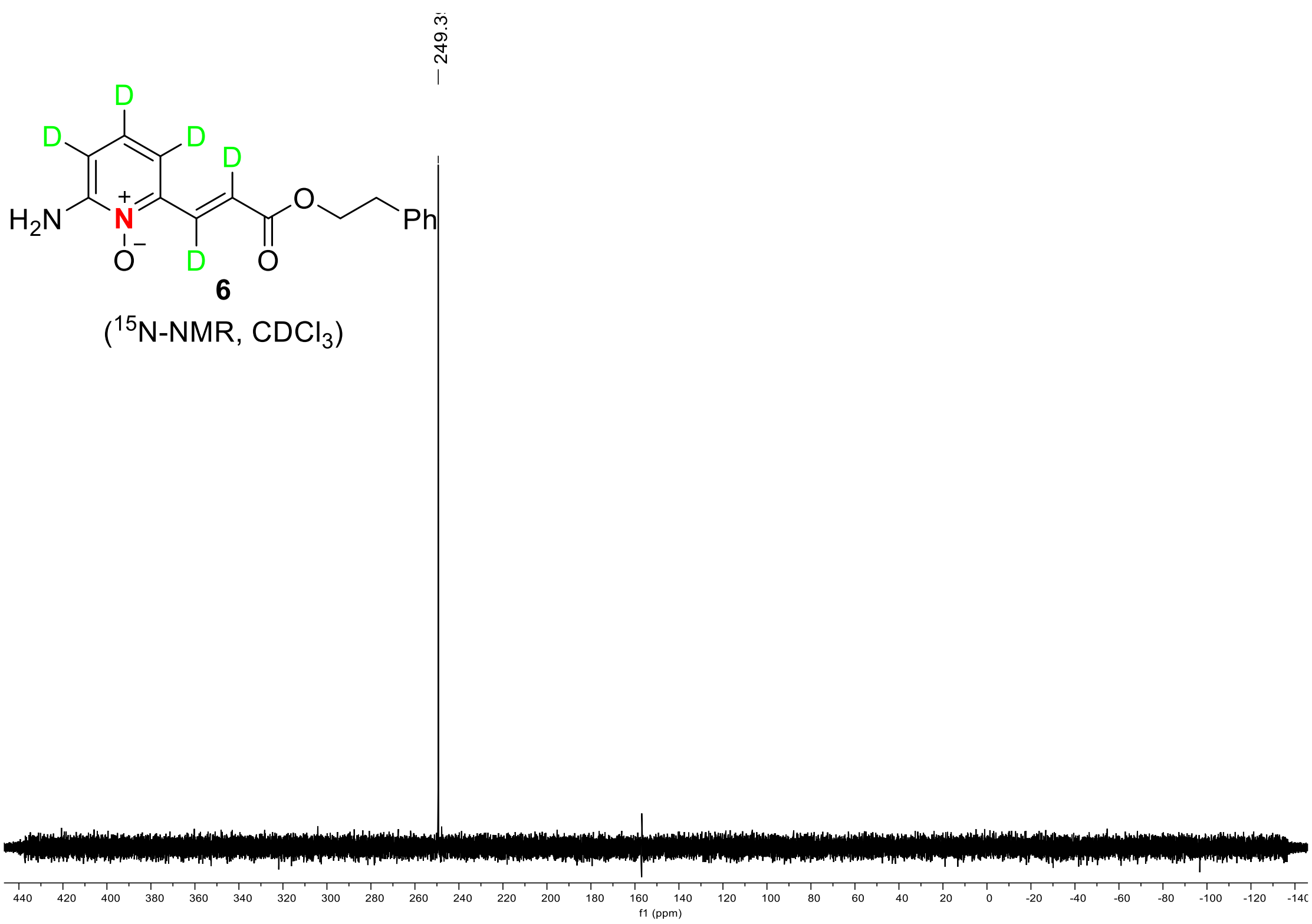

6

(^{15}N-NMR, $CDCl_3$)

Display Report

Analysis Info

Analysis Name	Z:\Data\2024\2409\sam030924\yli00100_3_01_127376.d	Acquisition Date	03.09.2024 18:07:02
Method	hystar_pl.m	Operator	BDAL@DE
Sample Name	yli00100	Instrument / Ser#	micrOTOF 10237
Comment			

Acquisition Parameter

Source Type	ESI	Ion Polarity	Positive	Set Nebulizer	1.2 Bar
Focus	Not active			Set Dry Heater	180 °C
Scan Begin	50 m/z	Set Capillary	4500 V	Set Dry Gas	4.0 l/min
Scan End	1600 m/z	Set End Plate Offset	-500 V	Set Divert Valve	Source

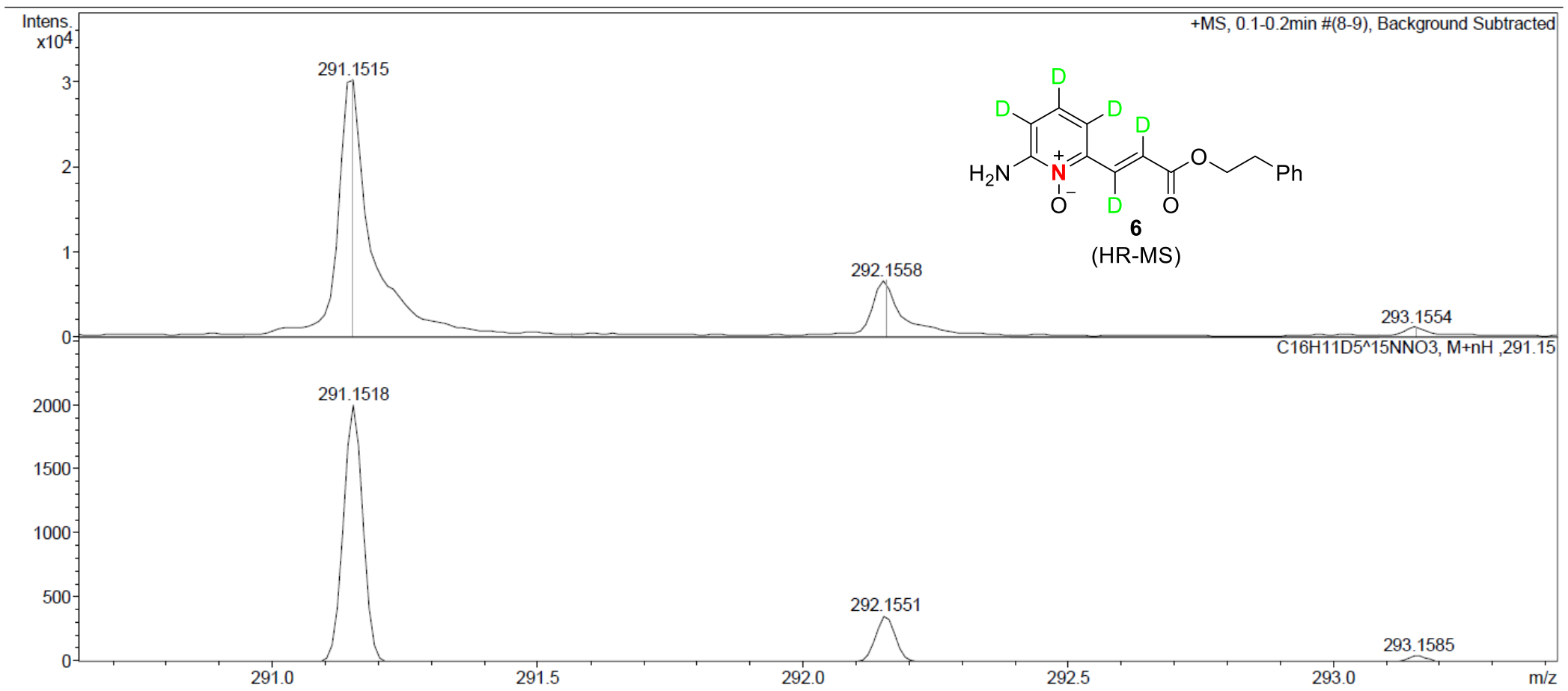

Bruker Compass DataAnalysis 4.0 printed: 03.09.2024 06:56:42 Page 1 of 1

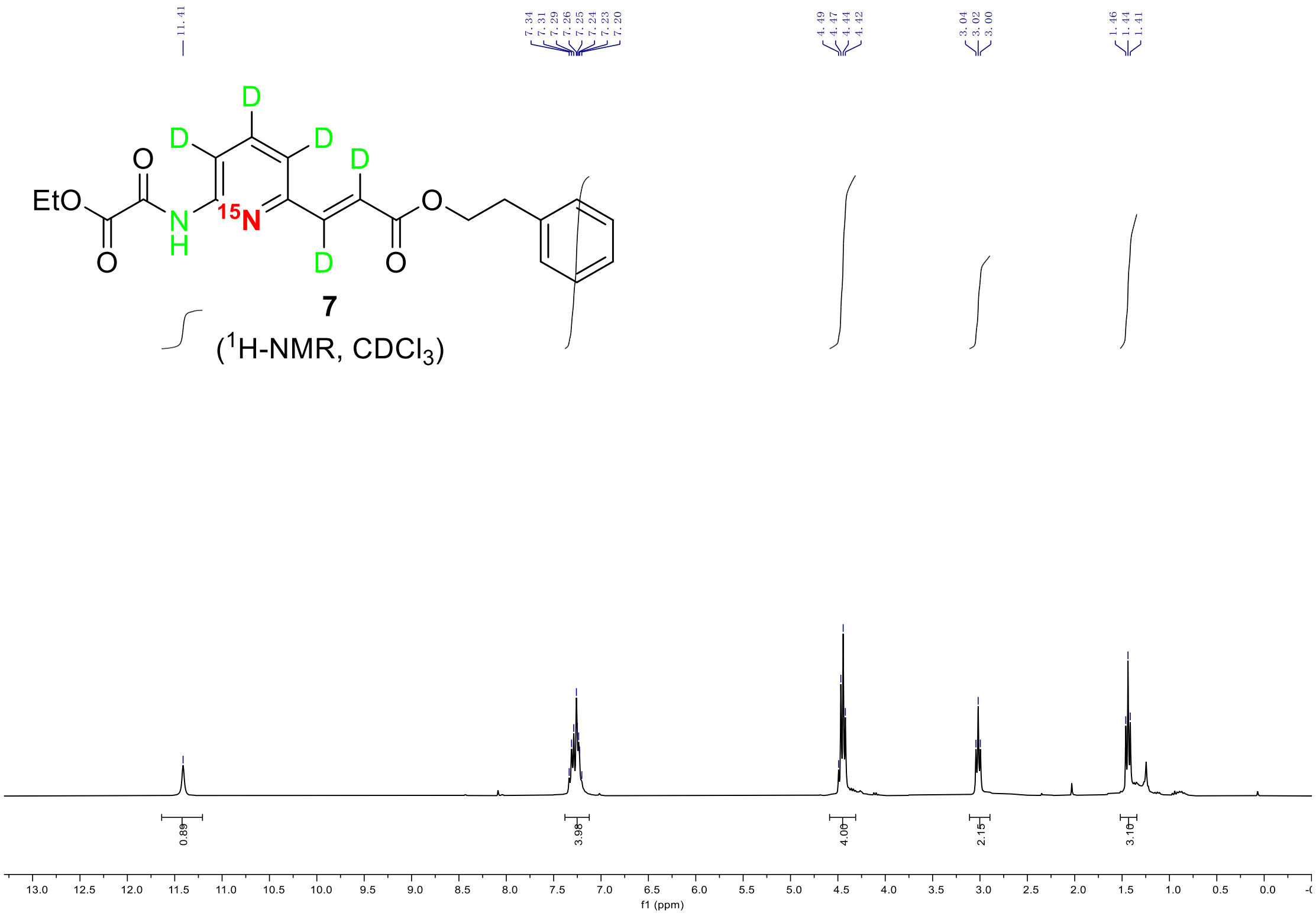

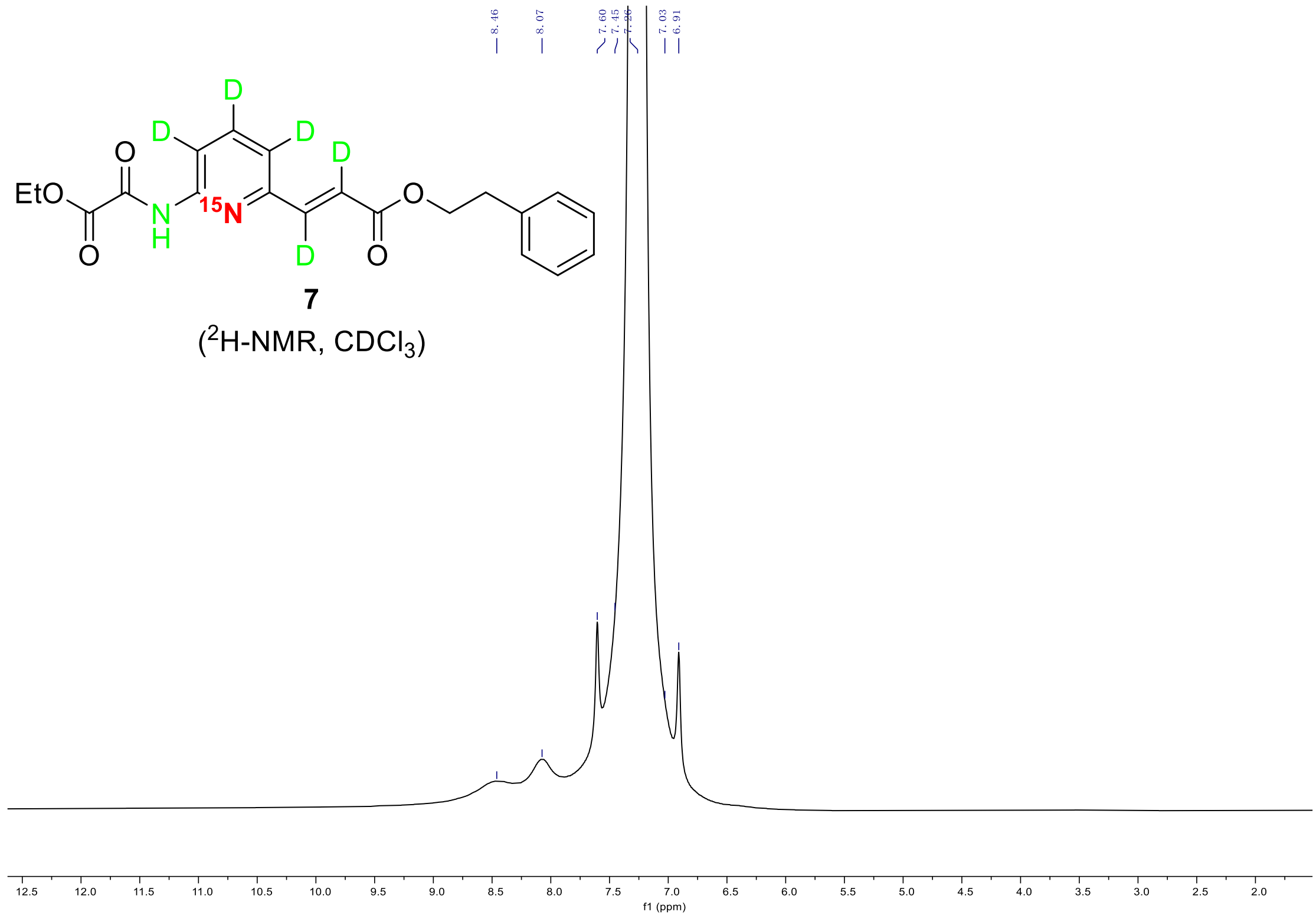
8.46
8.07
7.60
7.45
7.03
6.91
EtO
O
O
NH
15N
D
D
D
D
D
O
O
7
(2H-NMR, CDCl3)
f1 (ppm)

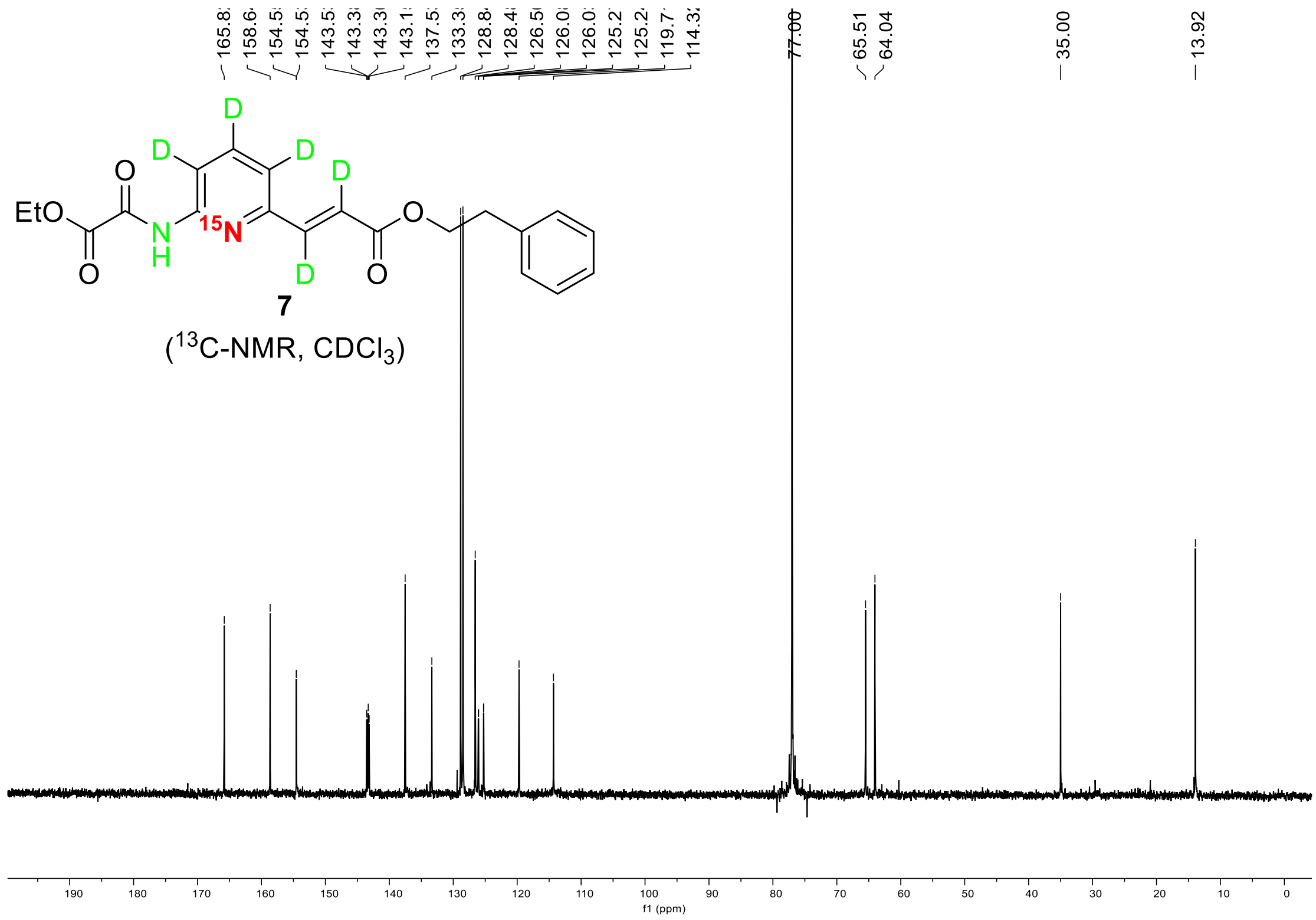
77.00
65.51
64.04
35.00
13.92
EtO
O
O
NH
15N
D
D
D
D
D
O
O
7
(13C-NMR, CDCl3)
f1 (ppm)

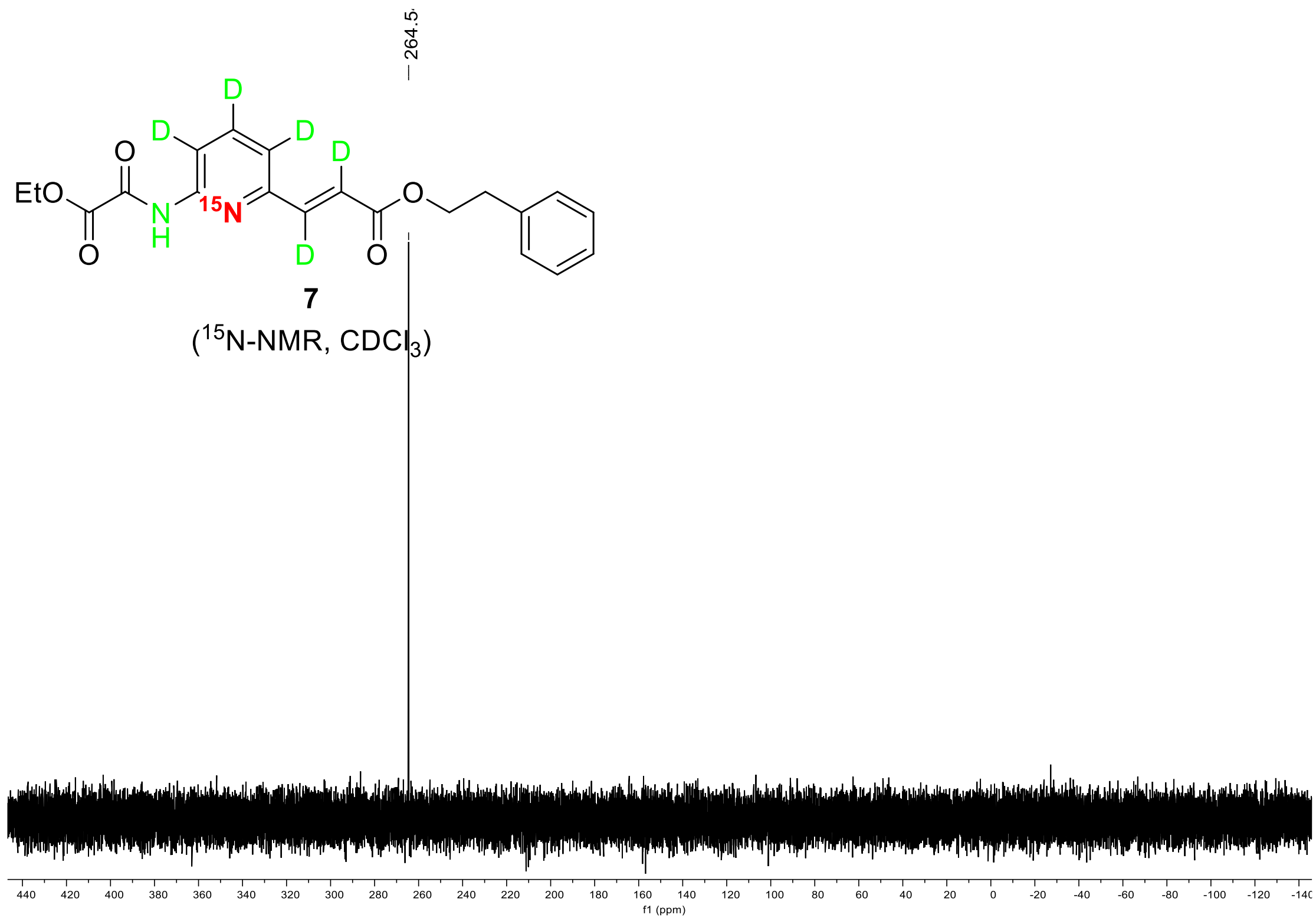

7

(^{15}N-NMR, $CDCl_3$)

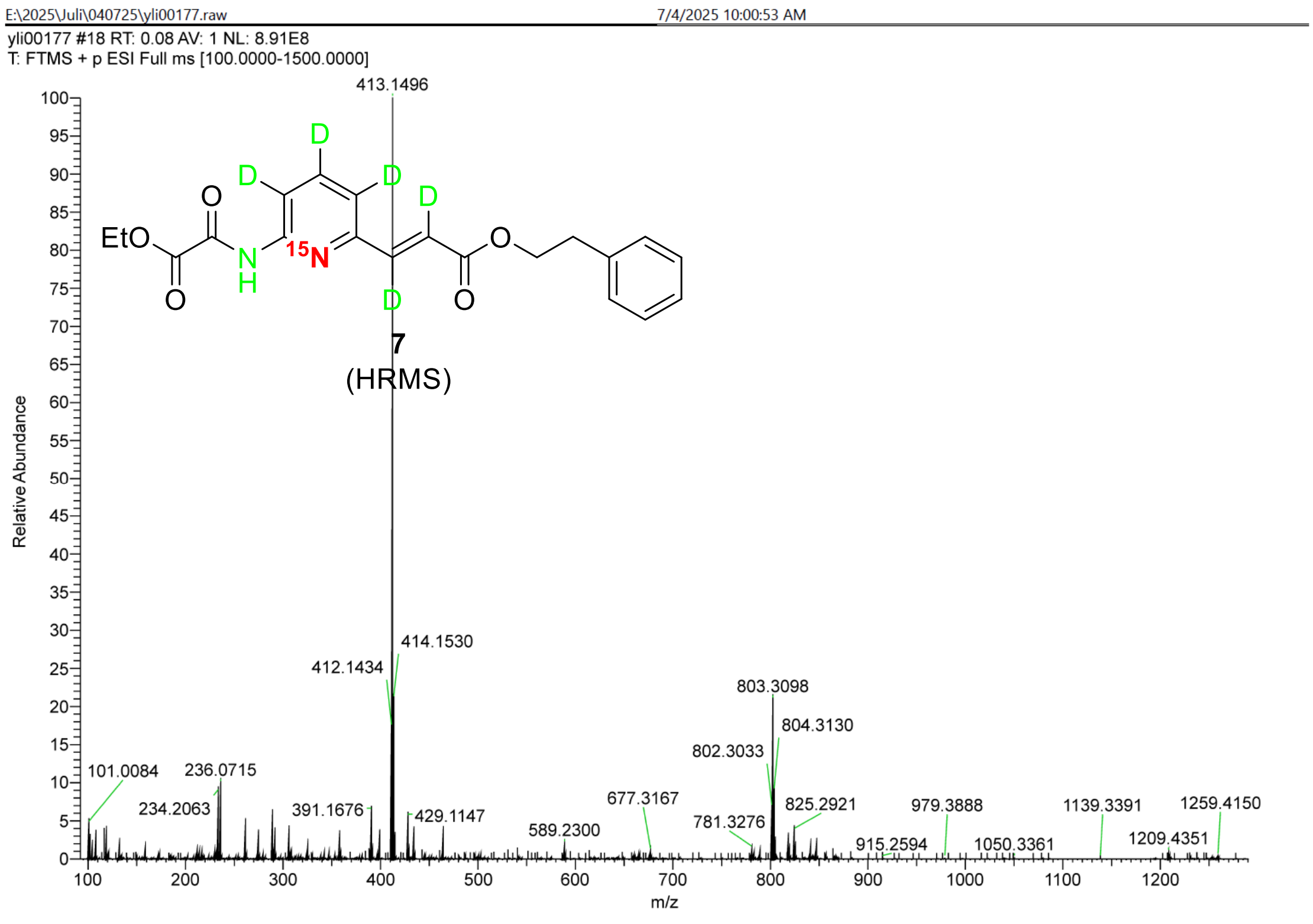

7

(HRMS)

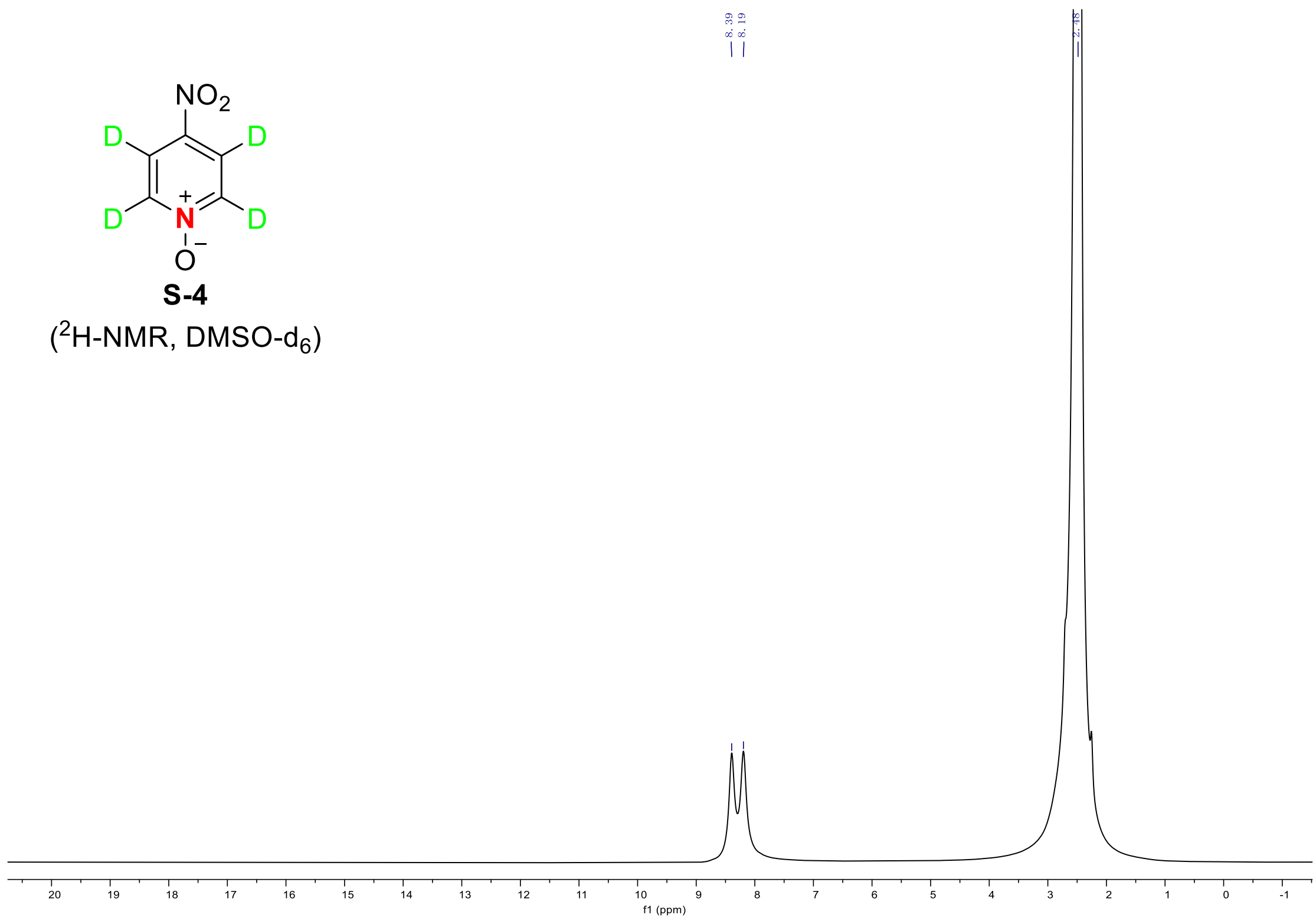

S-4

(^{2}H-NMR, DMSO-d_6)

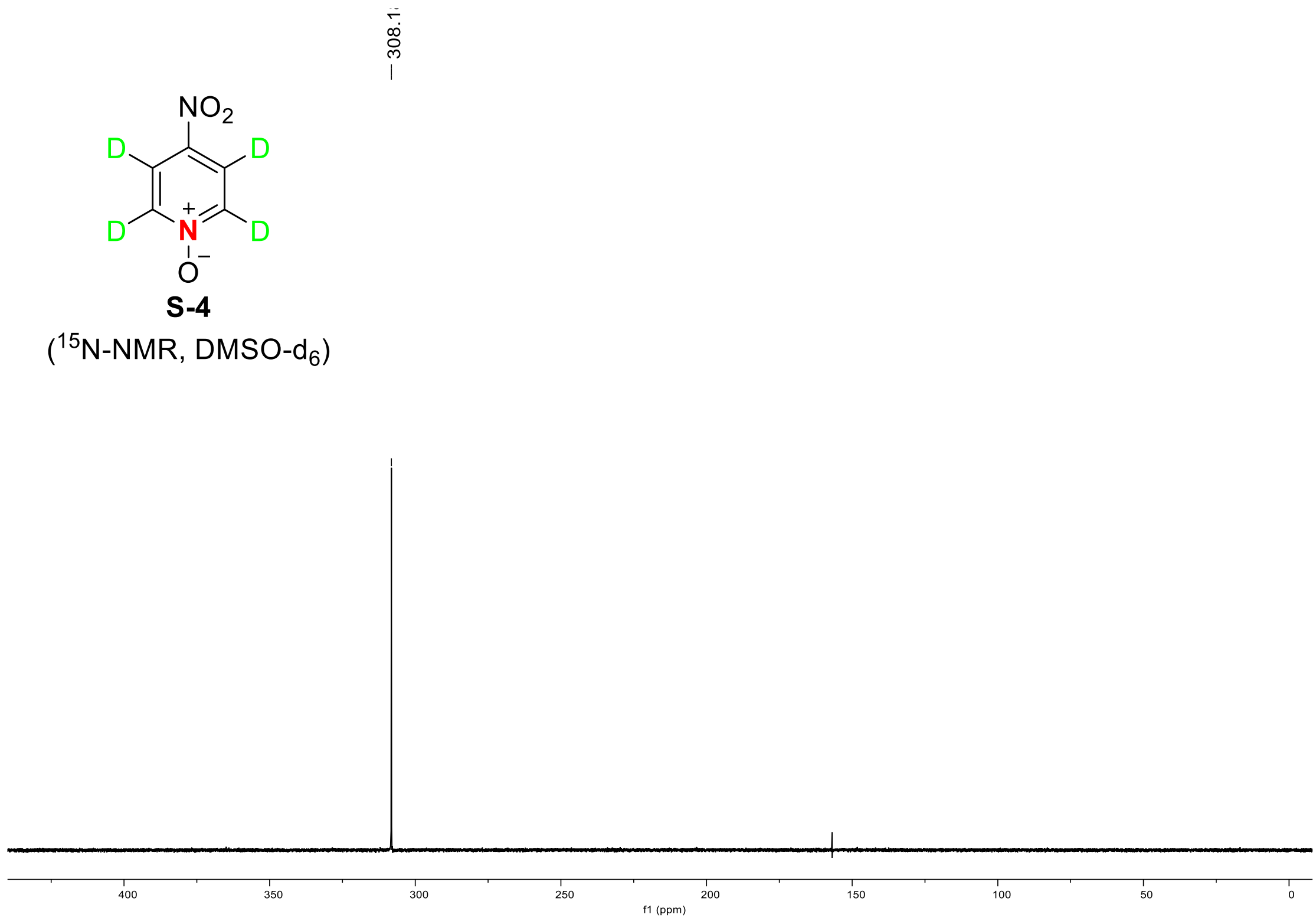

S-4

(^{15}N-NMR, DMSO-d_6)

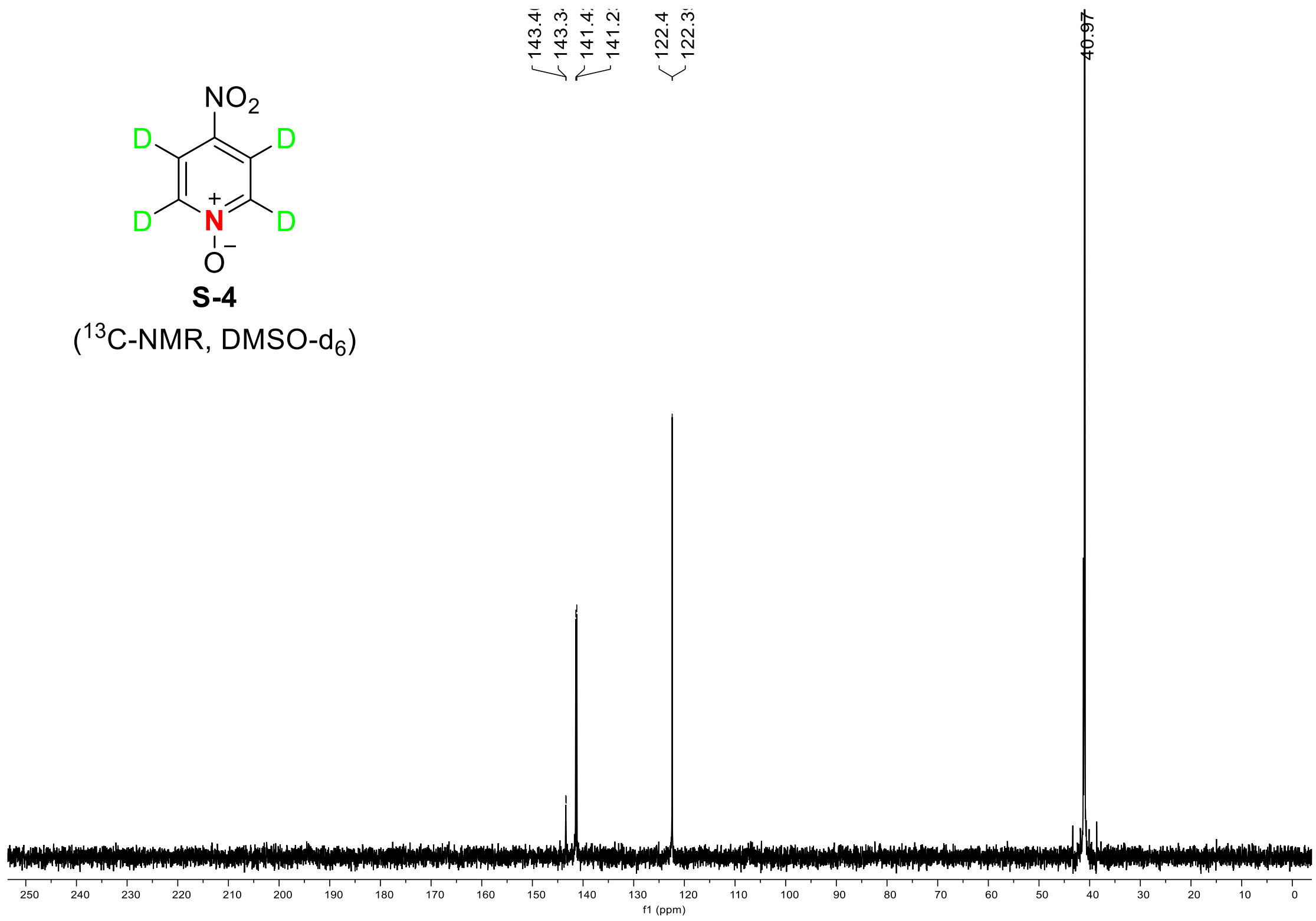
NO2
D
D
D
D
N
O
S-4
(13C-NMR, DMSO-d6)
143.4
143.3
141.4
141.2
122.4
122.3
40.97
f1 (ppm)

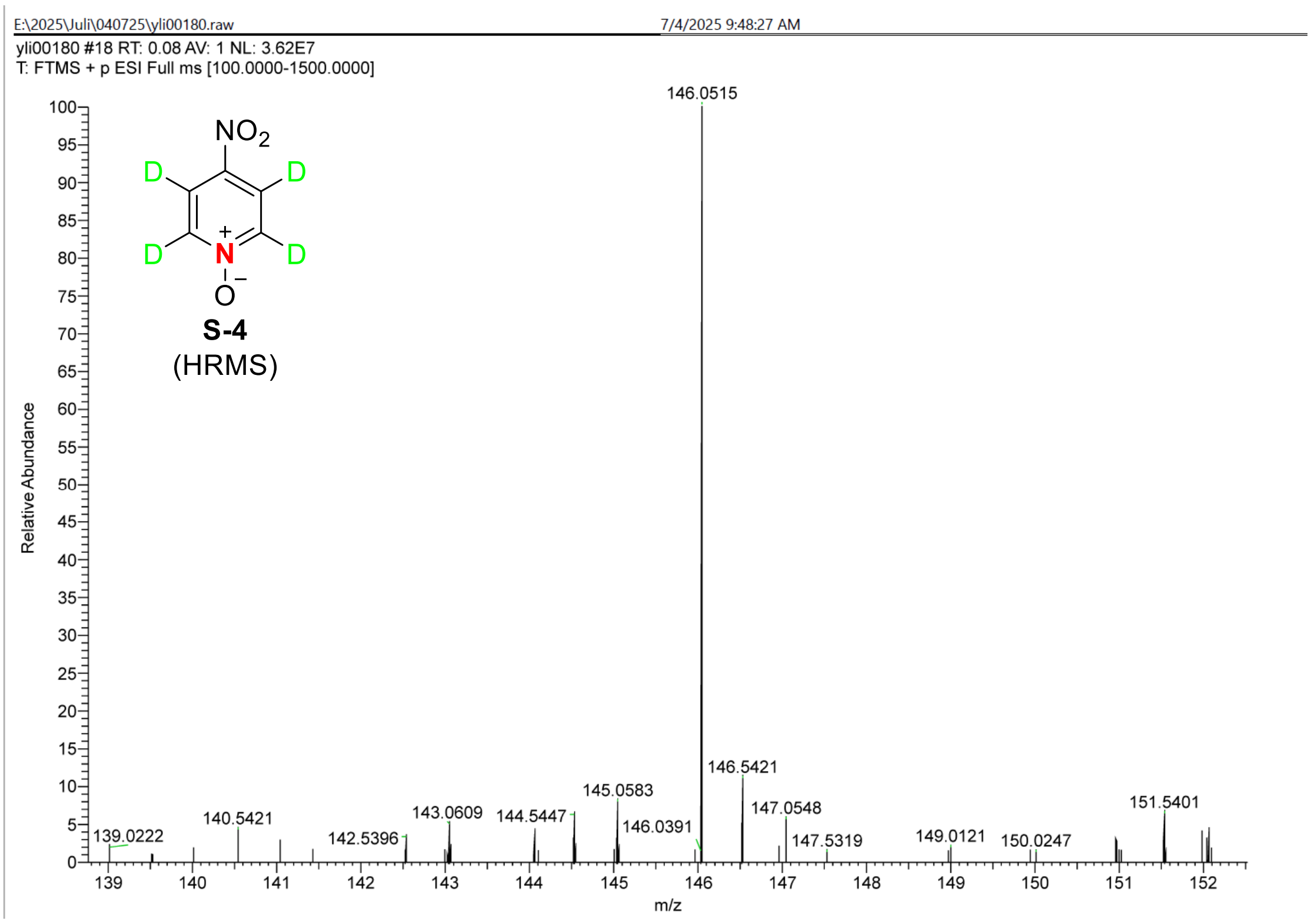
E:\2025\Juli\040725\yli00180.raw
7/4/2025 9:48:27 AM
yli00180 #18 RT: 0.08 AV: 1 NL: 3.62E7
T: FTMS + p ESI Full ms [100.0000-1500.0000]
146.0515
NO2
D
D
D
D
N
O
S-4
(HRMS)
Relative Abundance
146.5421
145.0583
147.0548
151.5401
140.5421
143.0609
144.5447
146.0391
139.0222
142.5396
147.5319
149.0121
150.0247
m/z

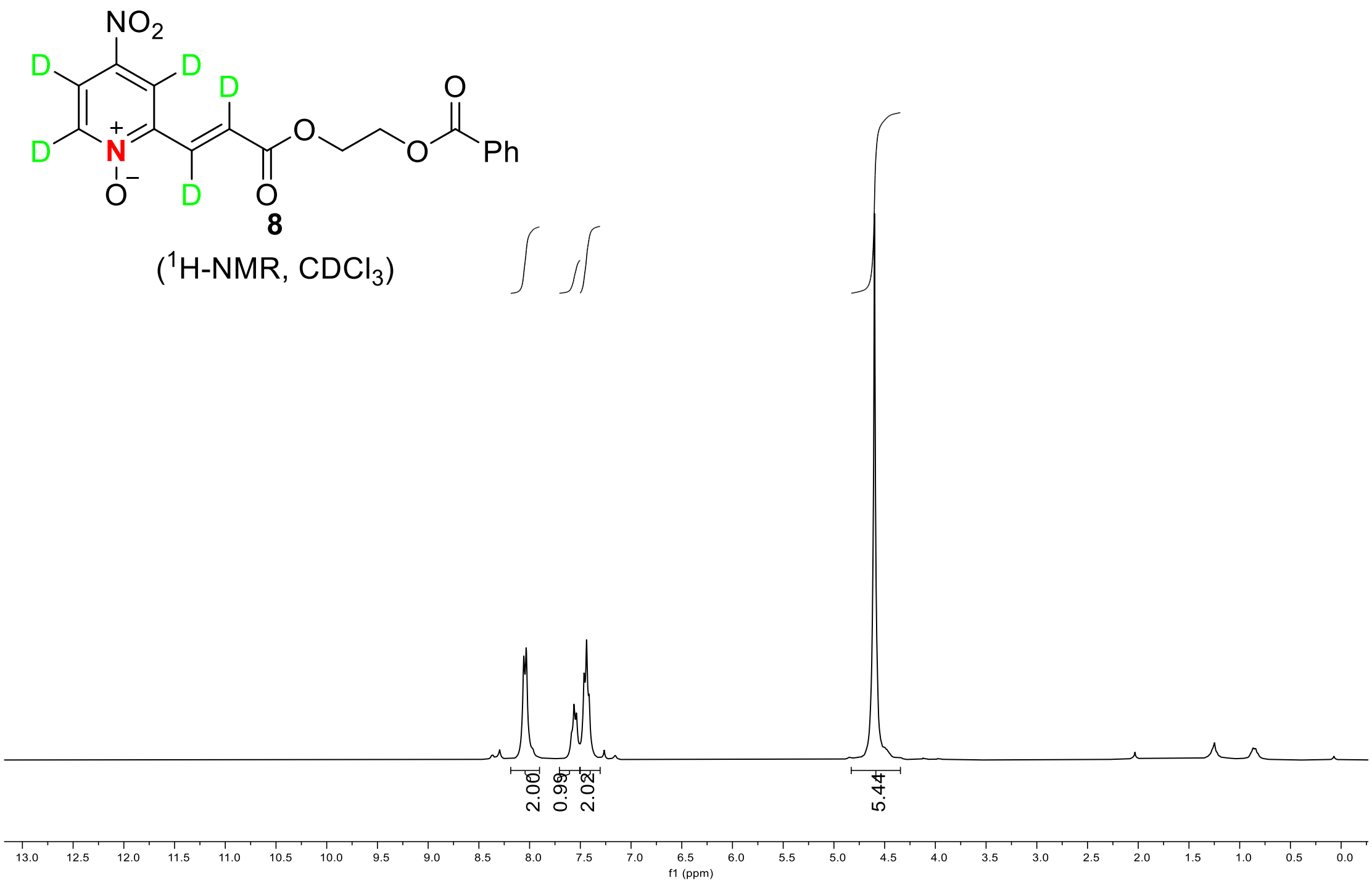

8

(^{1}H-NMR, $CDCl_3$)

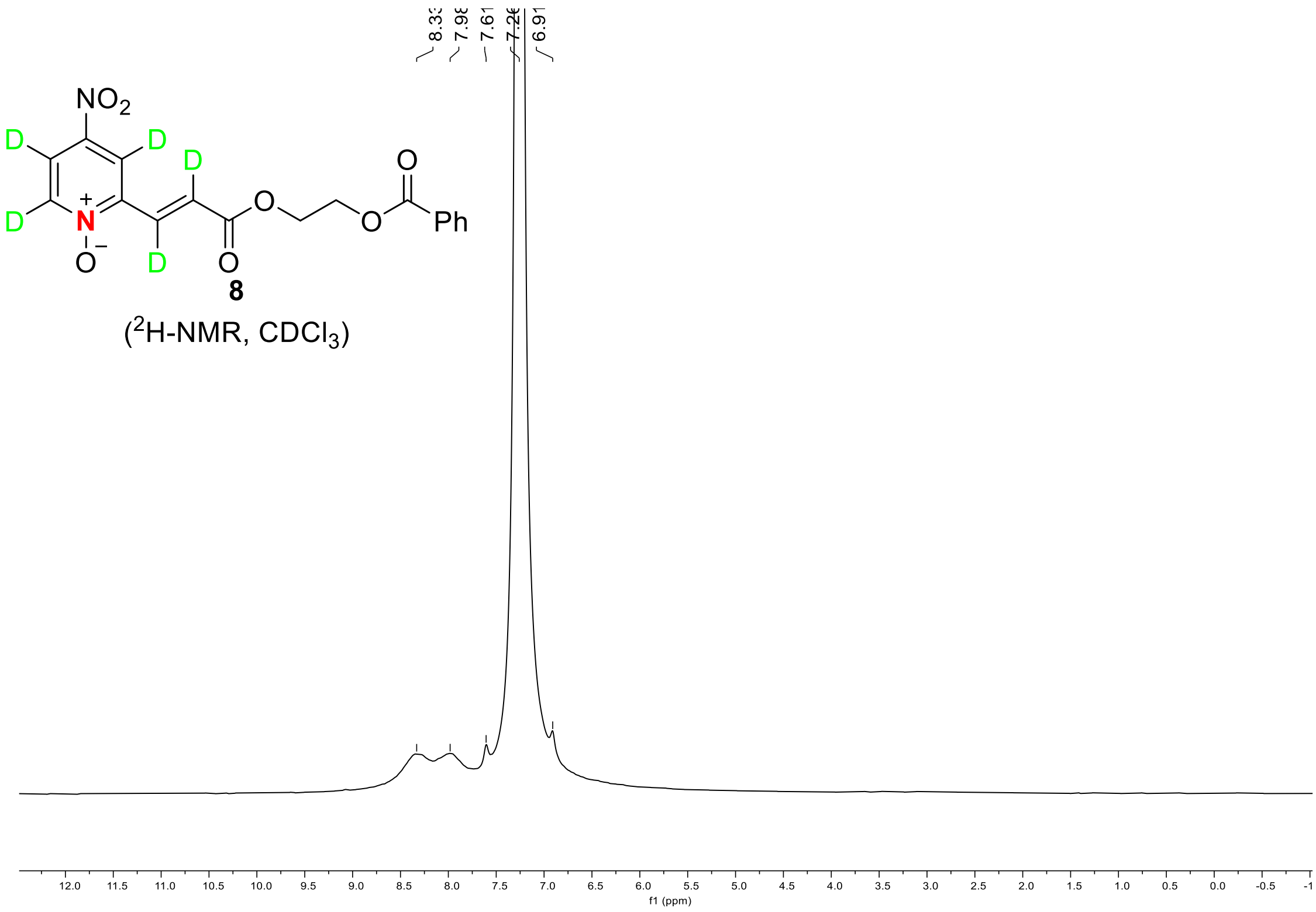

8

(^{2}H-NMR, $CDCl_3$)

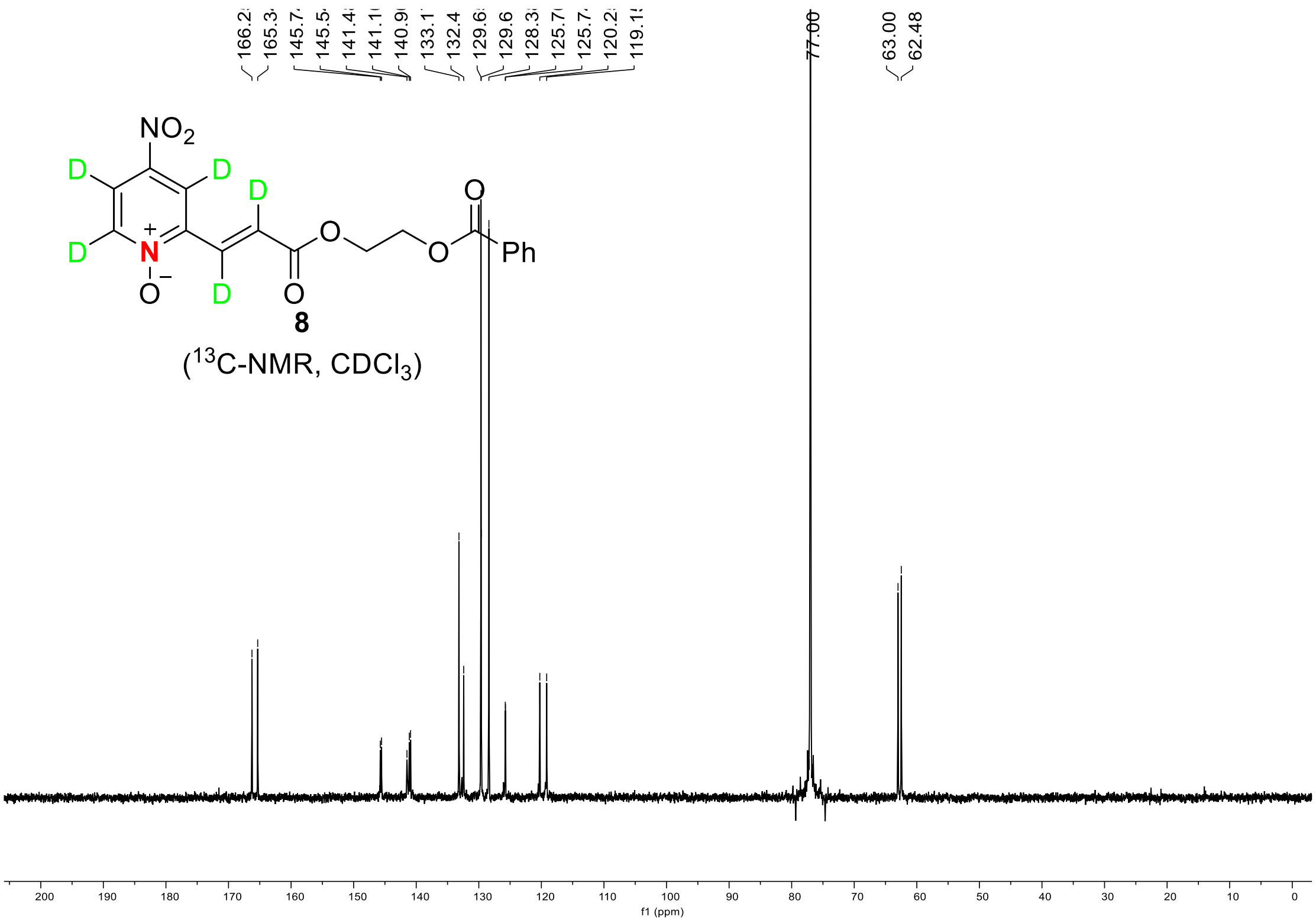

8

(^{13}C-NMR, $CDCl_3$)

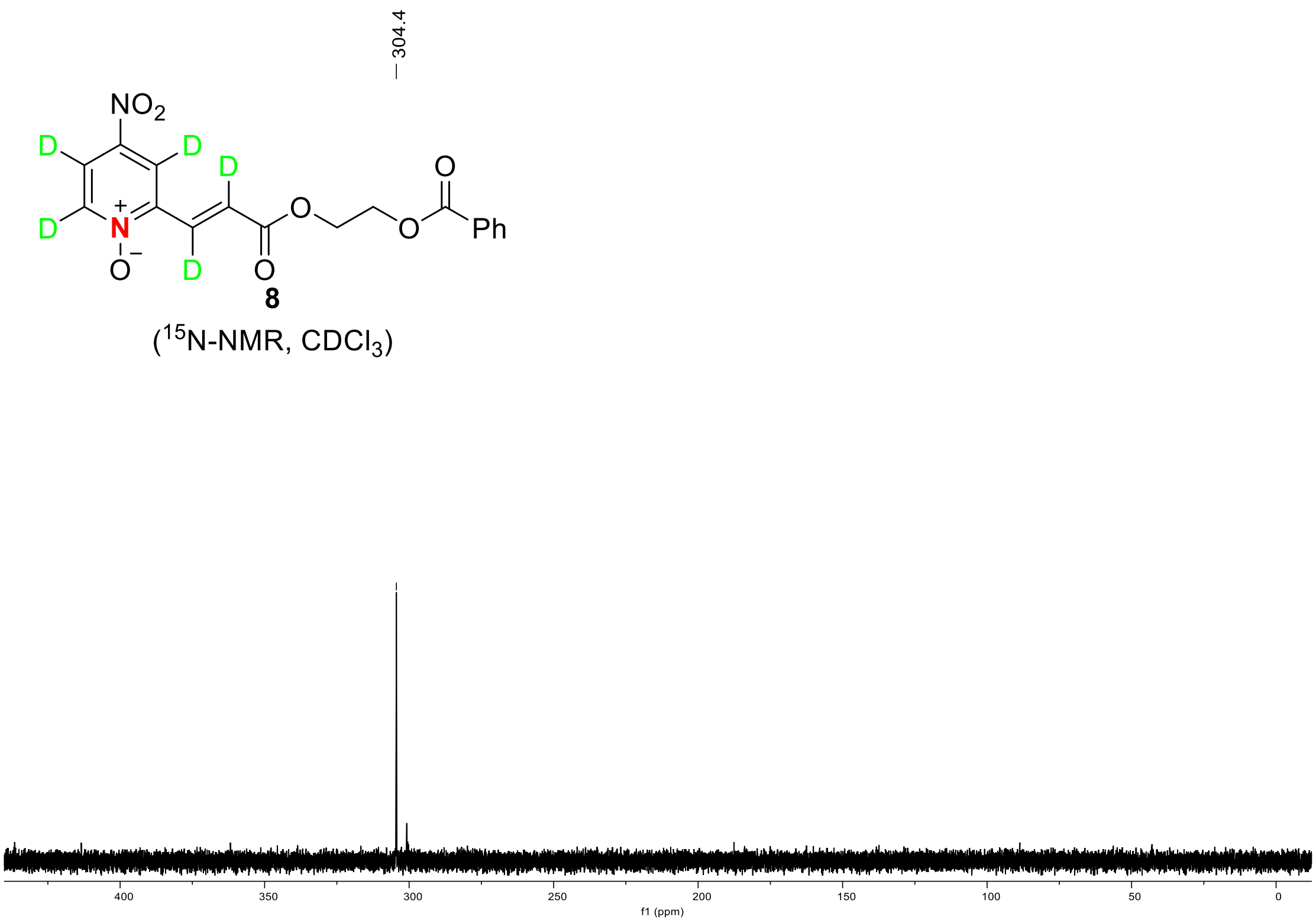

8

(^{15}N-NMR, $CDCl_3$)

Display Report

Analysis Info

Analysis Name	Z:\Data\2025\2503\sam110325\yli00128_5_01_130322.d	Acquisition Date	11.03.2025 15:29:06
Method	hystar_pl.m	Operator	BDAL@DE
Sample Name	yli00128	Instrument / Ser#	micrOTOF 10237
Comment			

Acquisition Parameter

Source Type	ESI	Ion Polarity	Positive	Set Nebulizer	1.2 Bar
Focus	Not active			Set Dry Heater	180 °C
Scan Begin	50 m/z	Set Capillary	4500 V	Set Dry Gas	4.0 l/min
Scan End	1600 m/z	Set End Plate Offset	-500 V	Set Divert Valve	Source

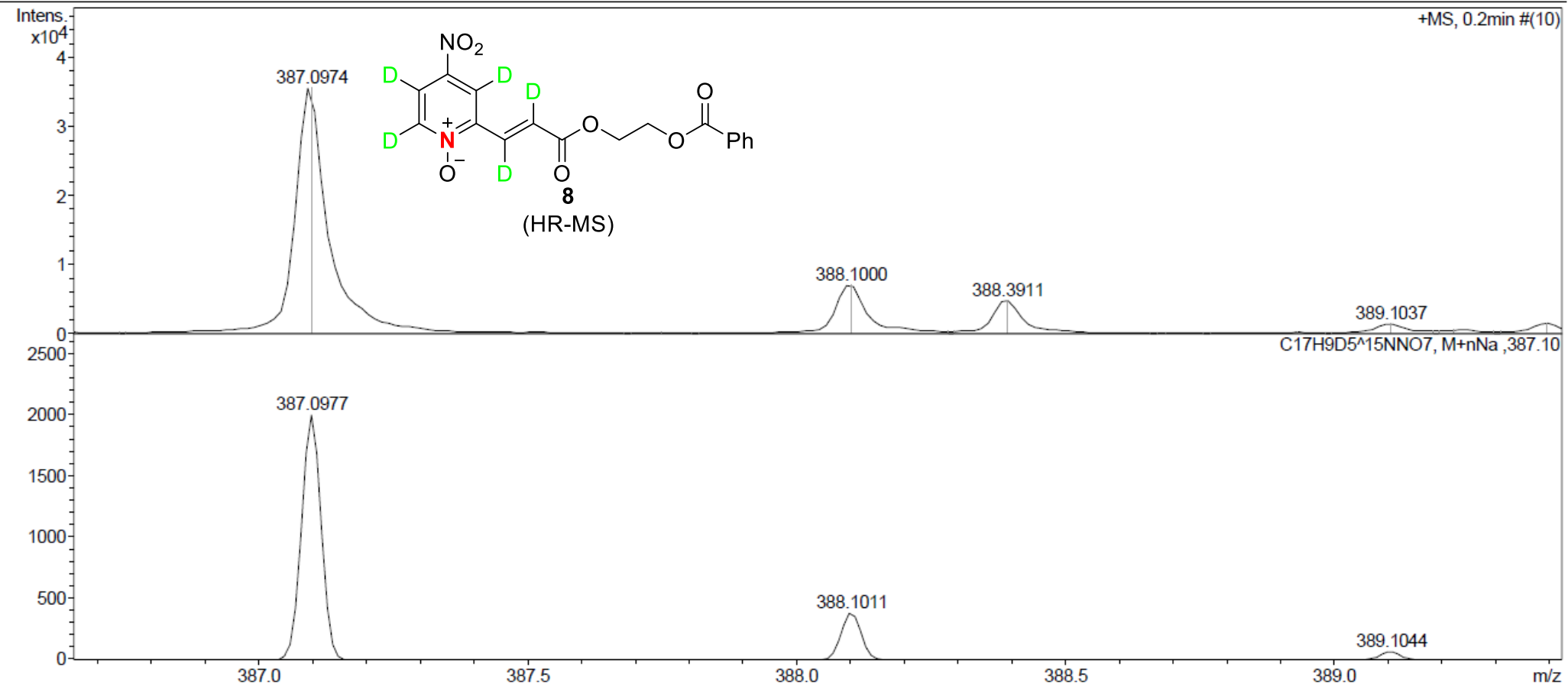

Bruker Compass DataAnalysis 4.0 printed: 11.03.2025 14:16:38 Page 1 of 1

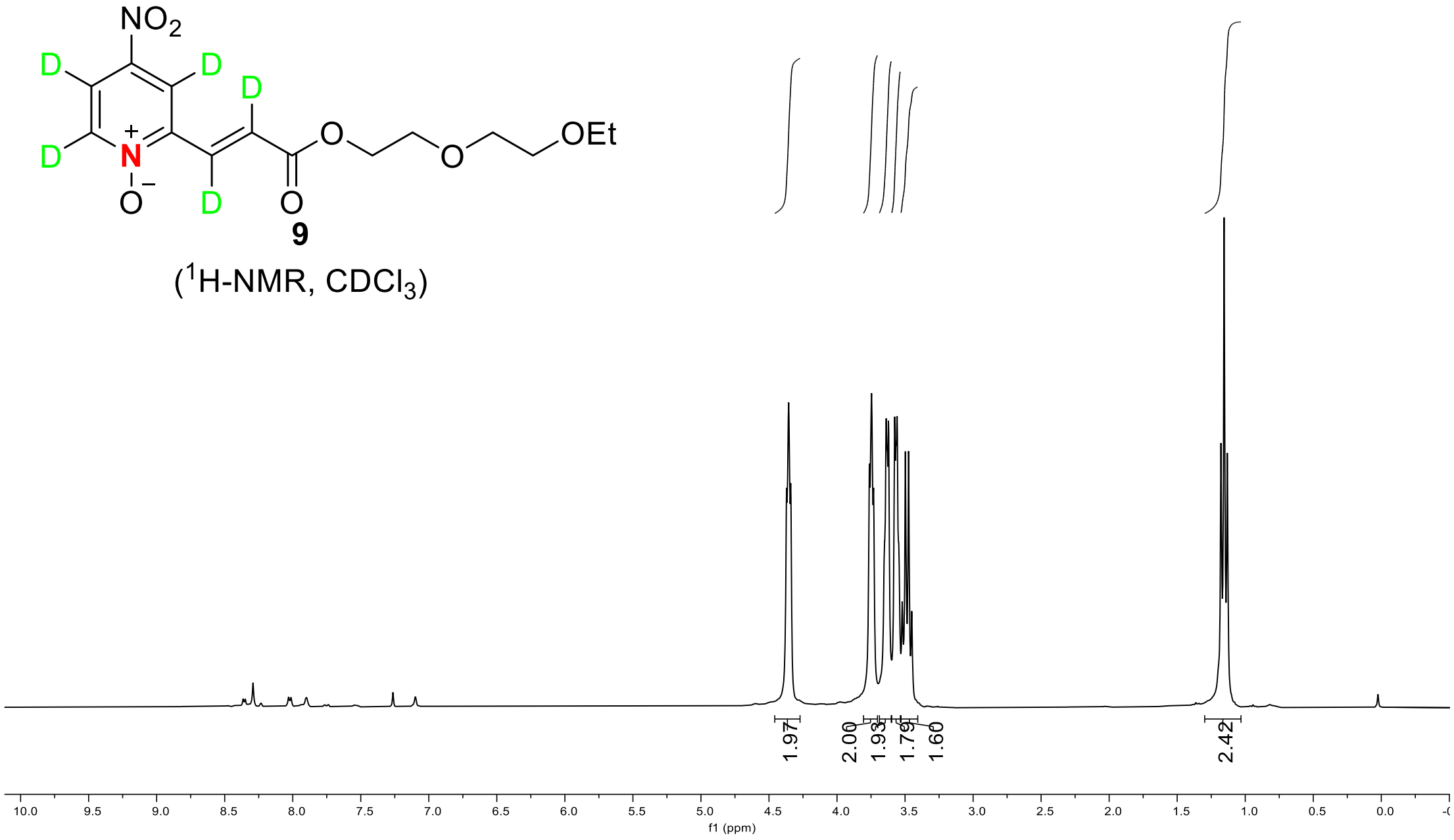

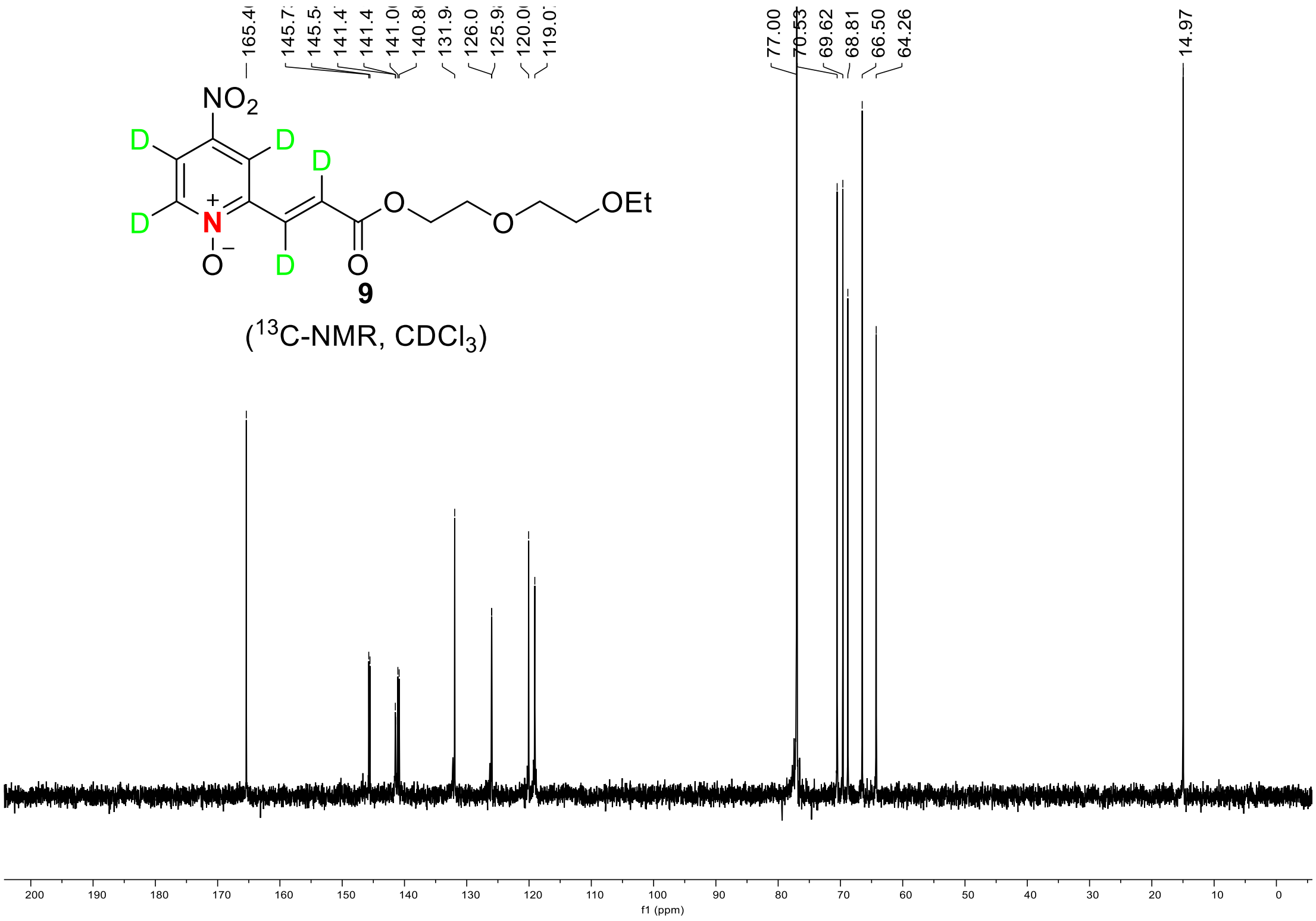
165.4
145.7
145.5
141.4
141.4
141.0
140.8
131.9
126.0
125.9
120.0
119.0
77.00
70.53
69.62
68.81
66.50
64.26
14.97
NO2
D
N
O
OEt
9
(13C-NMR, CDCl3)
f1 (ppm)

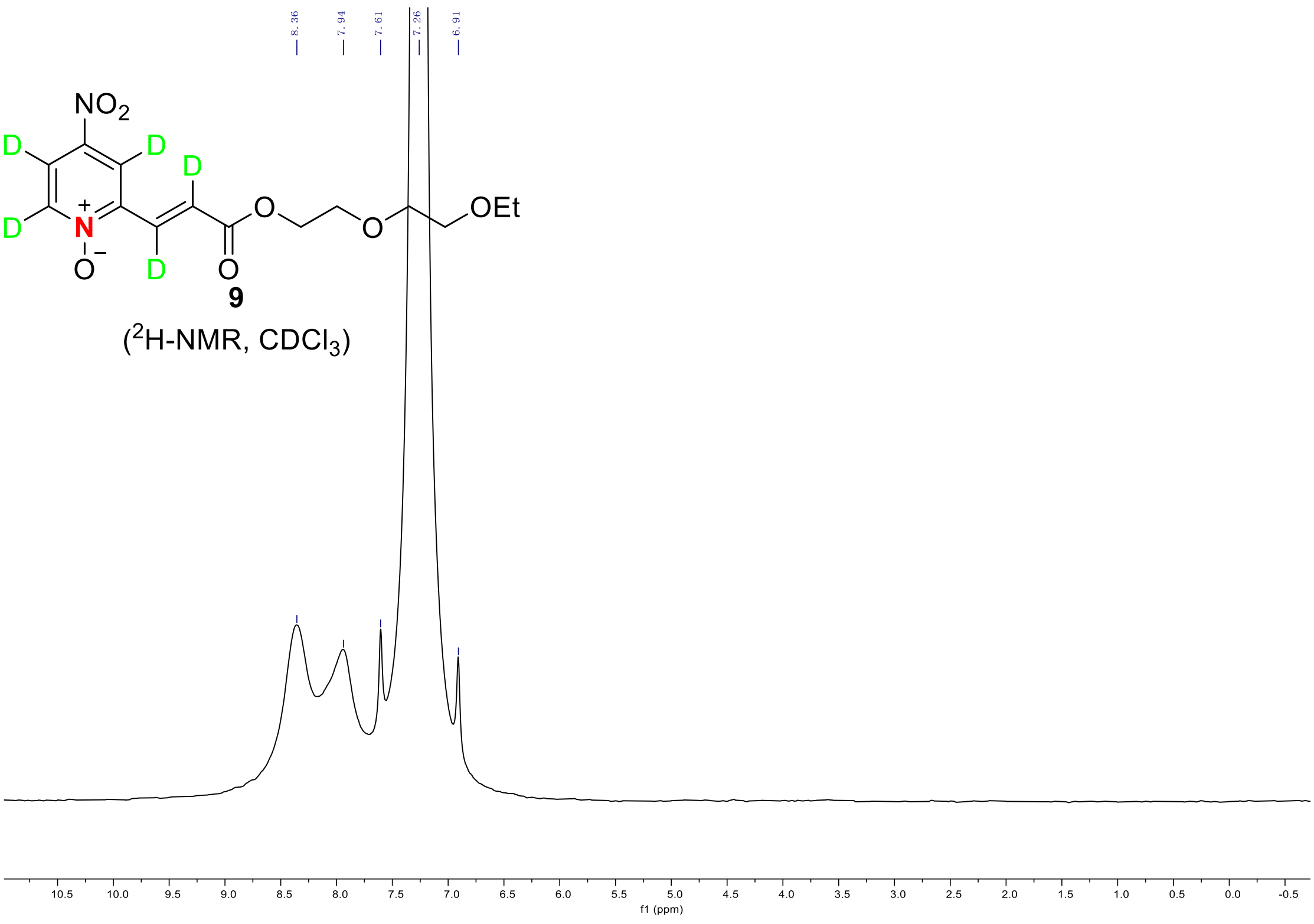
8.36
7.94
7.61
7.26
6.91
NO2
D
N
O
OEt
9
(2H-NMR, CDCl3)
f1 (ppm)

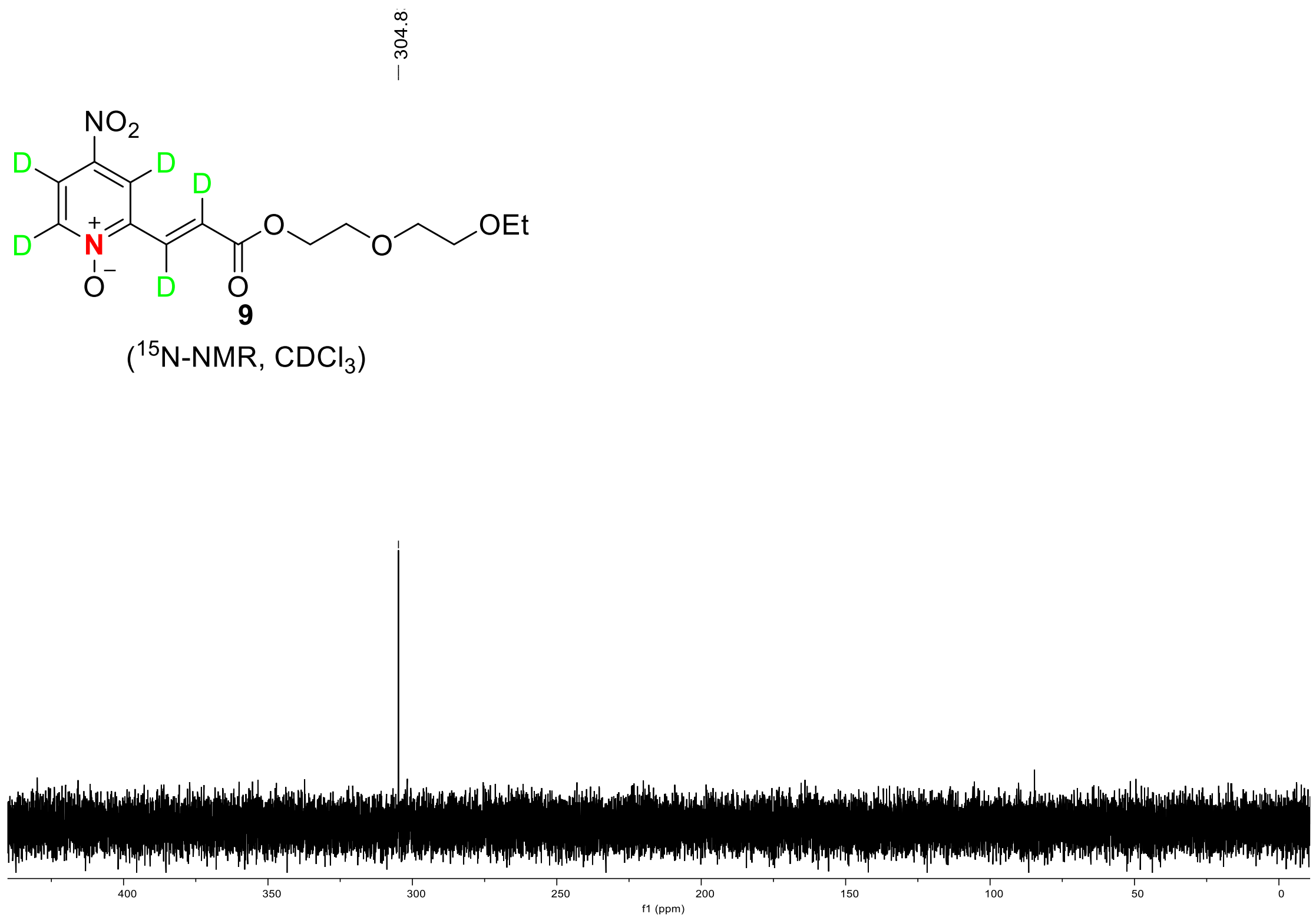

—304.8
NO2
D
D
D
D
D
N
O
O
O
OEt
9
(15N-NMR, CDCl3)
400
350
300
250
200
150
100
50
0
f1 (ppm)

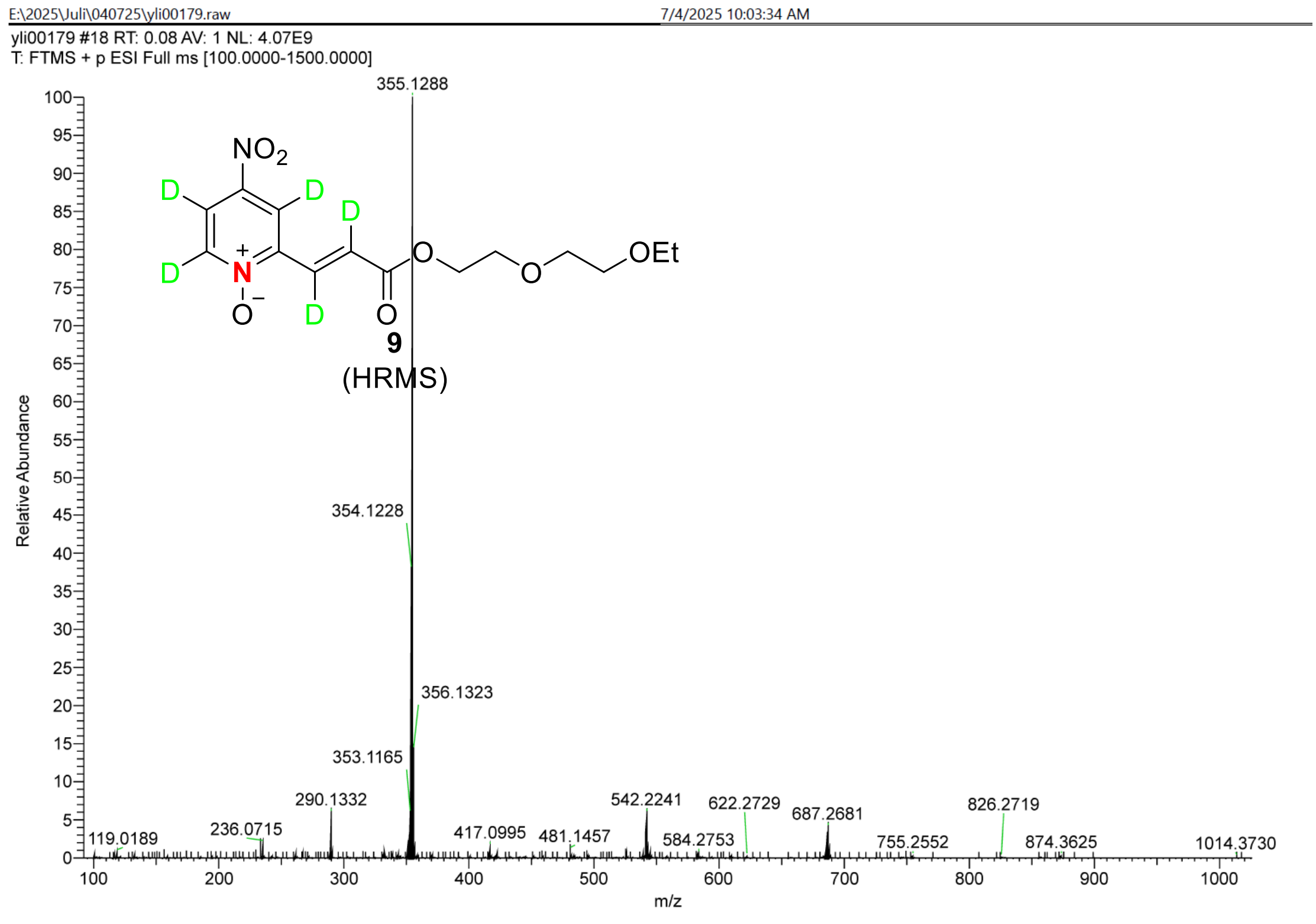

E:\2025\Juli\040725\yli00179.raw
7/4/2025 10:03:34 AM
yli00179 #18 RT: 0.08 AV: 1 NL: 4.07E9
T: FTMS + p ESI Full ms [100.0000-1500.0000]
355.1288
354.1228
356.1323
353.1165
290.1332
542.2241
622.2729
687.2681
826.2719
119.0189
236.0715
417.0995
481.1457
584.2753
755.2552
874.3625
1014.3730
Relative Abundance
m/z
9
(HRMS)

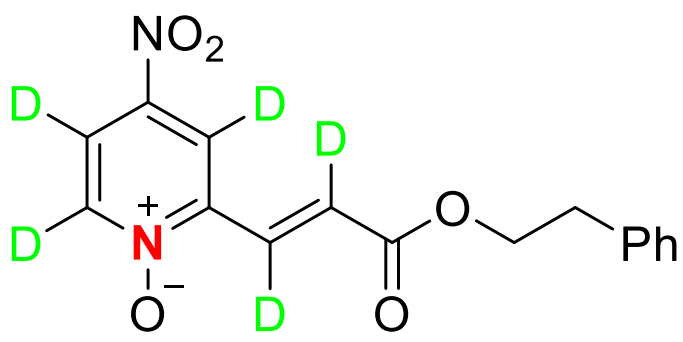

10

(^{1}H-NMR, $CDCl_3$)

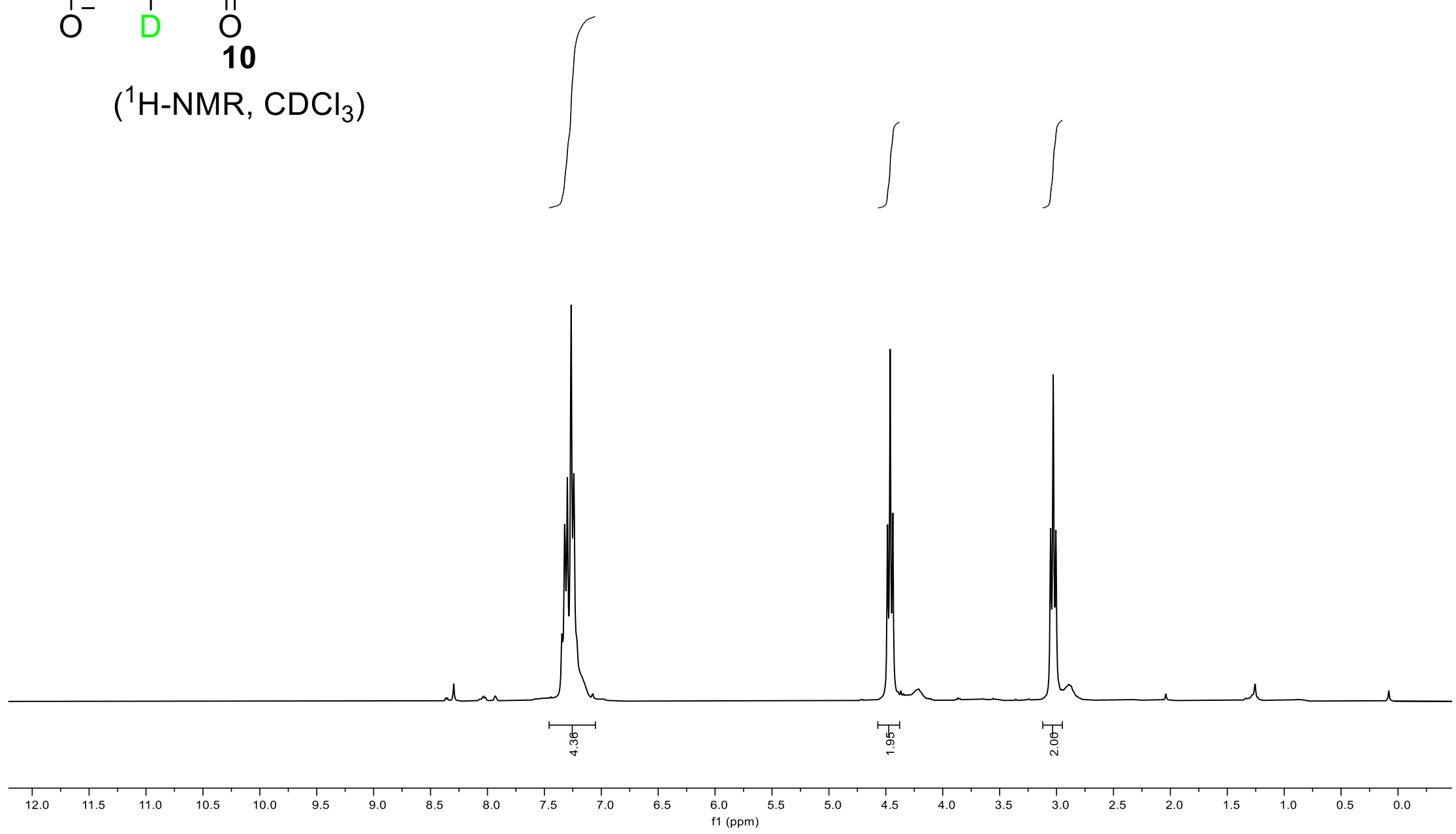

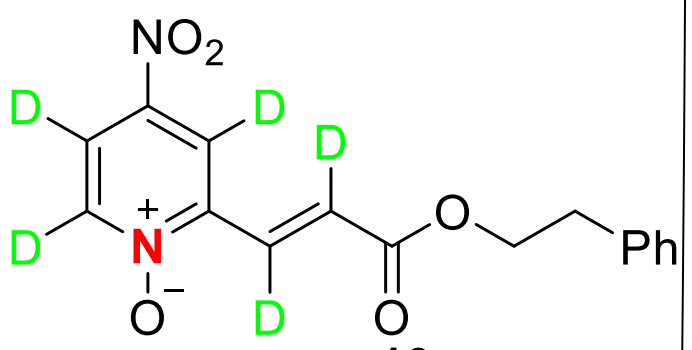

10

(^{2}H-NMR, $CDCl_3$)

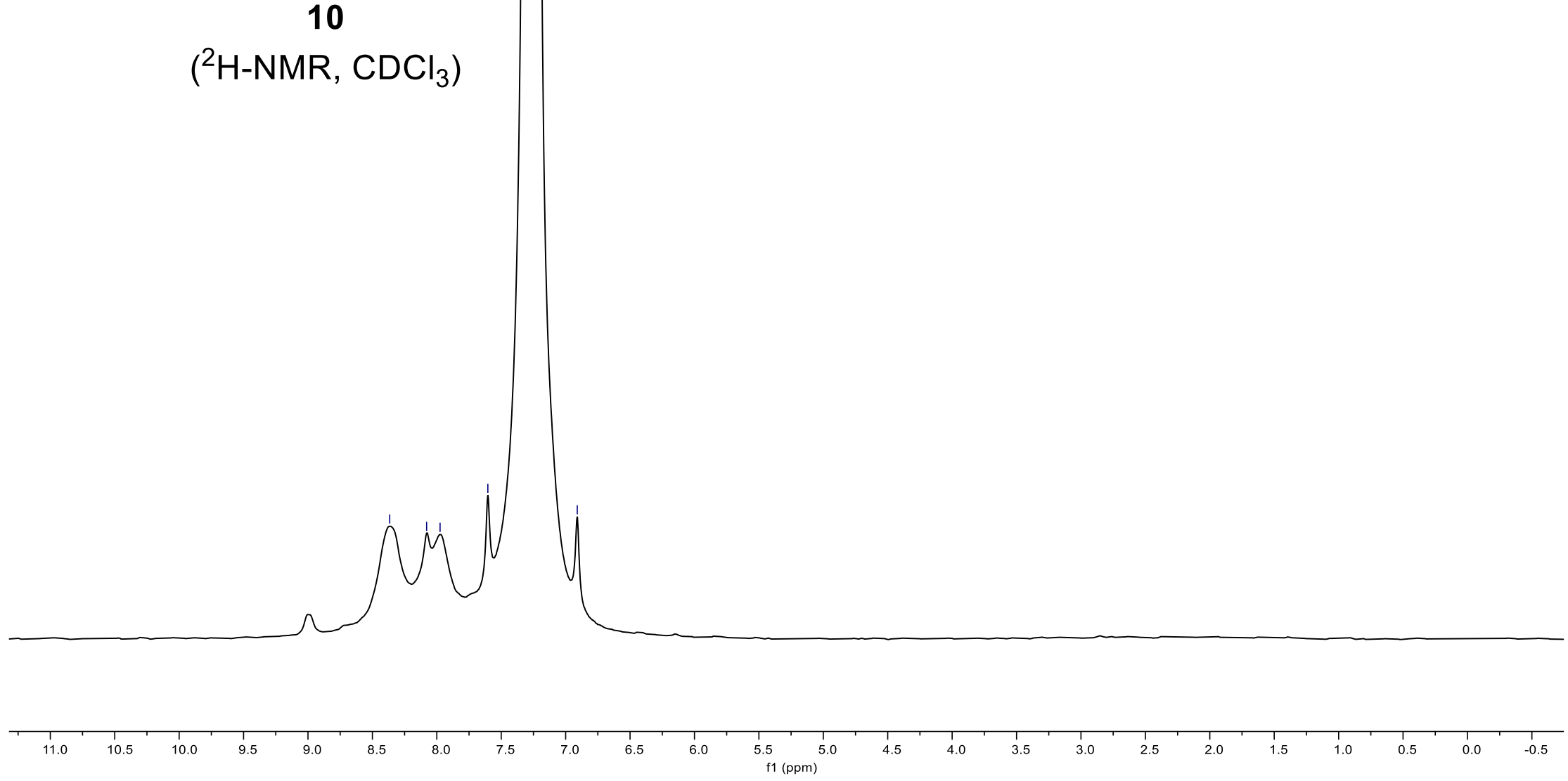

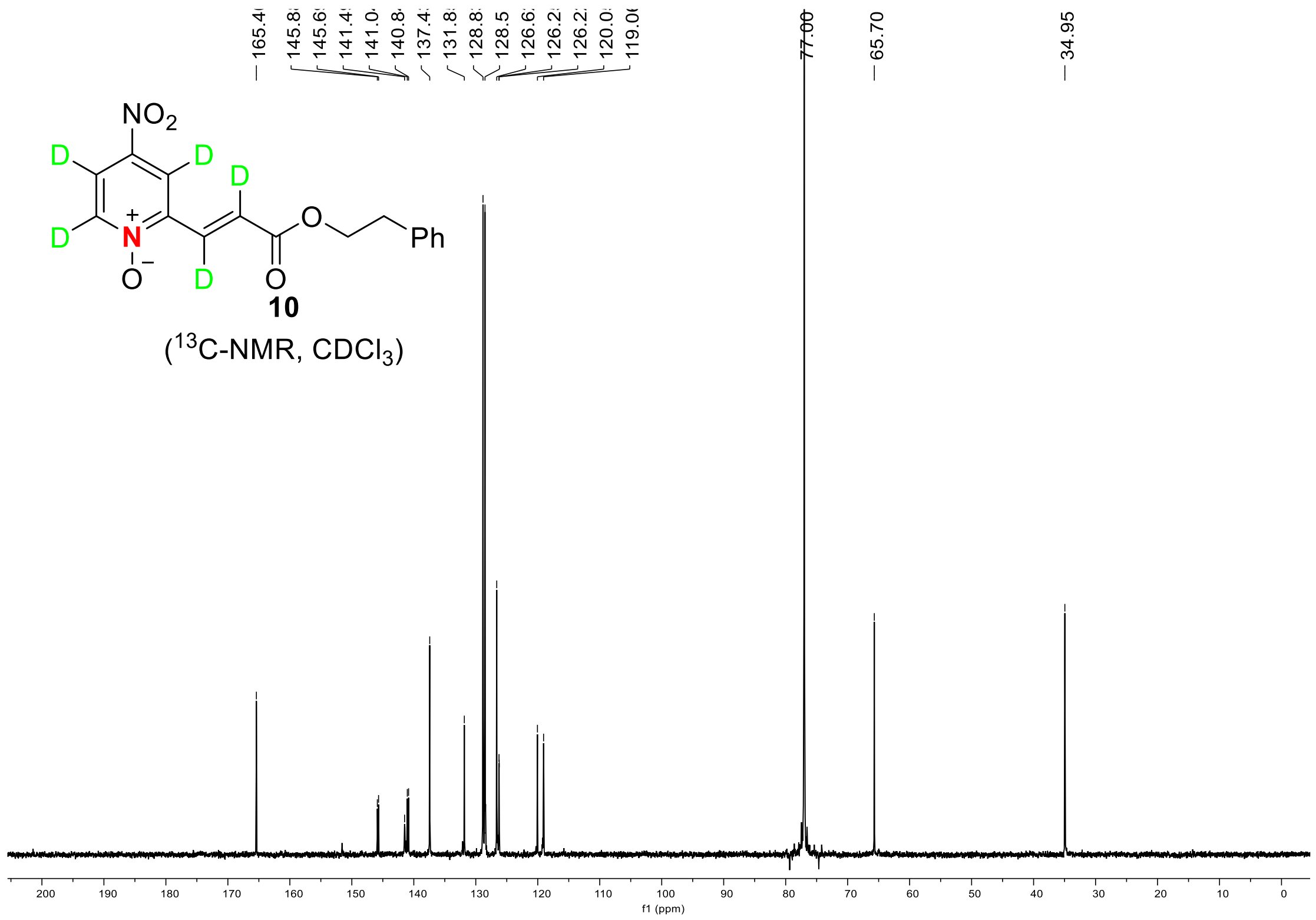

10

(^{13}C-NMR, $CDCl_3$)

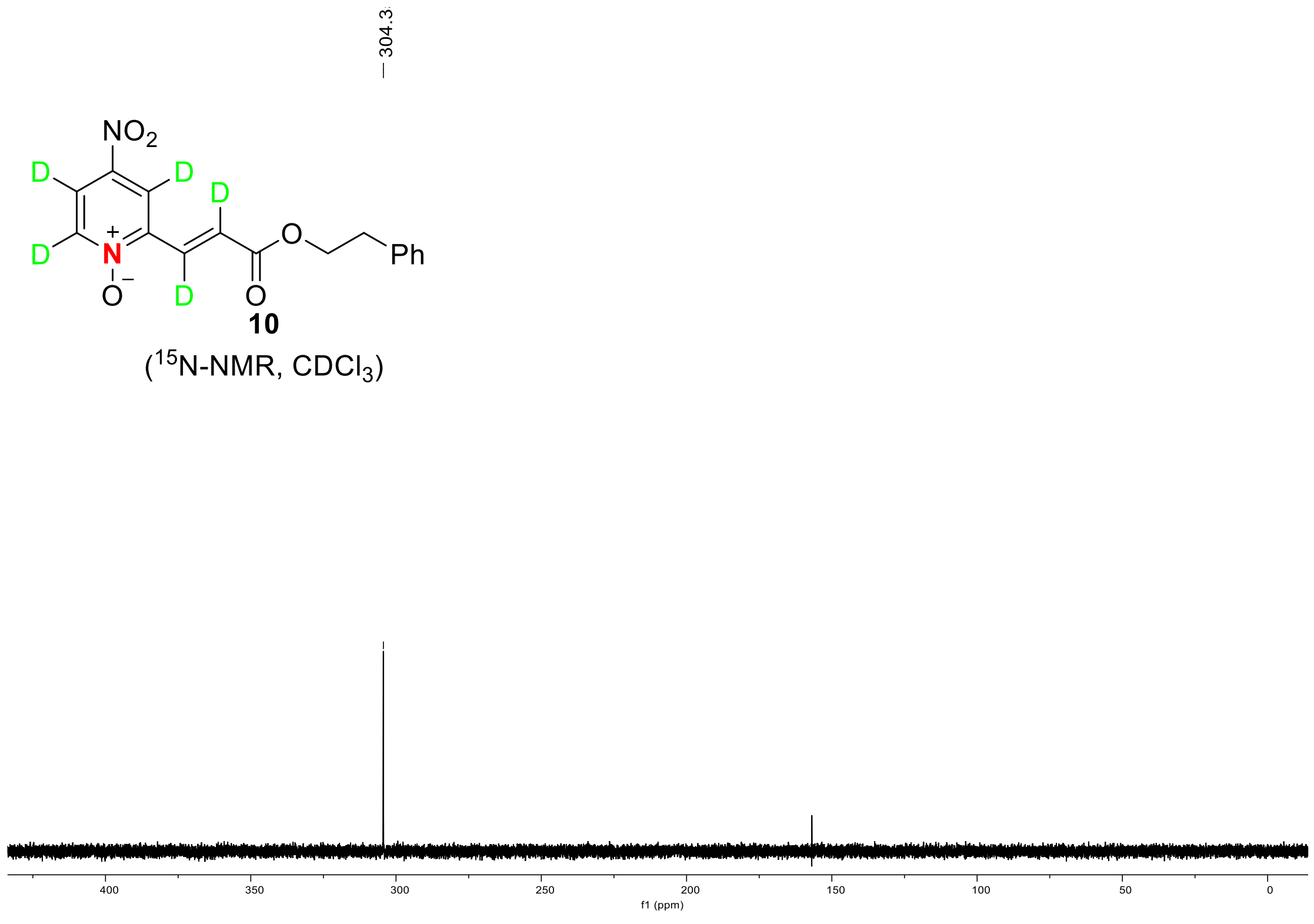

10

(^{15}N-NMR, $CDCl_3$)

\

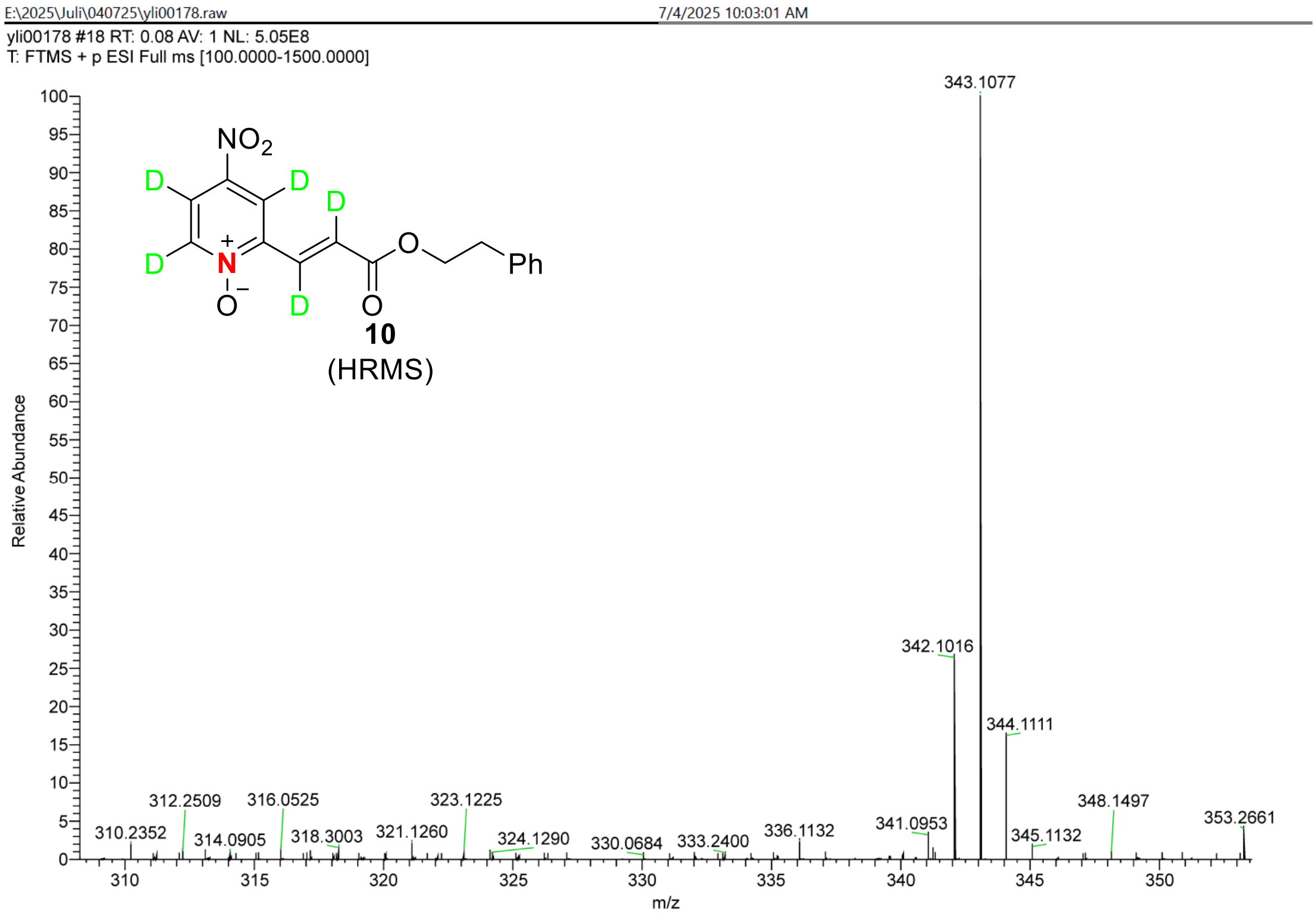

5.0 Reference.

[1] (a) P. Norcott, M. J. Burns, P. J. Rayner, R. E. Mewis, S. B. Duckett, *Magn. Reson. Chem.* **2018**, *56*, 663–671; (b) T. W. Whaley, D. G. Ott, *Journal of Labelled Compounds* **1974**, *10*, 283–286; (c) A. Svyatova, I. V. Skovpin, N. V. Chukanov, K. V. Kovtunov, E. Y. Chekmenev, A. N. Pravdivtsev, J.-B. Hövener, I. V. Koptyug, *Chem. Eur. J.* **2019**, *25*, 8465–8470.

[2] A. Bechtoldt, L. Ackermann, *ChemCatChem* **2019**, *11*, 435–438.

[3] S. H. Cho, S. J. Hwang, S. Chang, *J. Am. Chem. Soc.* **2008**, *130*, 9254–9256.

[4] J. Yin, B. Xiang, M. A. Huffman, C. E. Raab, I. W. Davies, *J. Org. Chem.* **2007**, *72*, 4554–4557.

[5] (a) S. Korchak, S. Yang, S. Mamone, S. Glöggler, *ChemistryOpen* **2018**, *7*, 344–348; (b) S. Korchak, S. Mamone, S. Glöggler, *ChemistryOpen* **2018**, *7*, 672–676.

[6] (a) A. P. Jagtap, L. Kaltschnee, S. Glöggler, *Chem. Sci.* **2019**, *10*, 8577–8582. (b) R. Mei, L. M. Fries, T. L. K. Hune, M. D. Santi, G. G. Rodriguez, S. Sternkopf, S. Glöggler, *Angew. Chem. Int. Ed.* **2024**, *63*, e202403144.
